# Exploring the Catalytic Promiscuity of Phenolic Acid Decarboxylases: Asymmetric, 1,6‐Conjugate Addition of Nucleophiles Across 4‐Hydroxystyrene

**DOI:** 10.1002/adsc.201700247

**Published:** 2017-05-08

**Authors:** Stefan E. Payer, Xiang Sheng, Hannah Pollak, Christiane Wuensch, Georg Steinkellner, Fahmi Himo, Silvia M. Glueck, Kurt Faber

**Affiliations:** ^1^ Austrian Centre of Industrial Biotechnology (ACIB)c/o Department of ChemistryUniversity of GrazHeinrichstrasse 28, A-8010GrazAustria; ^2^ Department of ChemistryUniversity of GrazHeinrichstrasse 28, A-8010GrazAustria; ^3^ Arrhenius LaboratoryDepartment of Organic ChemistryStockholm UniversitySE-106 91StockholmSweden; ^4^ Center for Molecular BiosciencesUniversity of GrazHumboldtstrasse 508010GrazAustria

**Keywords:** biocatalysis, catalytic promiscuity, decarboxylase, hydration, hydroxystyrene, nucleophile addition

## Abstract

The catalytic promiscuity of a ferulic acid decarboxylase from *Enterobacter* sp. (FDC_*E*s) and phenolic acid decarboxylases (PADs) for the asymmetric conjugate addition of water across the C=C bond of hydroxystyrenes was extended to the N‐, C‐ and S‐nucleophiles methoxyamine, cyanide and propanethiol to furnish the corresponding addition products in up to 91% *ee*. The products obtained from the biotransformation employing the most suitable enzyme/nucleophile pairs were isolated and characterized after optimizing the reaction conditions. Finally, a mechanistic rationale supported by quantum mechanical calculations for the highly (*S*)‐selective addition of cyanide is proposed.

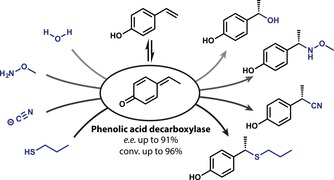

## Introduction

The asymmetric addition of water across C=C bonds was denoted as a “dream reaction”, because it allows one to convert a prochiral alkene with 100% atom efficiency into a non‐racemic *sec*‐alcohol.^1^ However, industrial‐scale production of simple bulk alcohols from olefins *via* hydration in the gas phase suffers from limited productivity^2^ and asymmetric variants are only rarely found.^3^

An attractive alternative to the use of chemo‐catalytic protocols is the use of lyases (EC 4.2.X.X), which catalyze the addition of nucleophiles onto electrophilic acceptor molecules. Besides aminases^4^ and carbolyases^5^ forming C–N and C–C bonds, respectively, hydratases engage water as nucleophile and constitute a lyase‐subgroup (EC 4.2.1.X) of which *ca*. 170 were discovered up to now.^6^ Hydratases catalyze the electrophilic addition of water onto isolated double (e.g., stearate and oleate hydratase)^7^ and triple bonds (e.g., acetylene hydratase).^8^ Alternatively, hydration occurs *via* nucleophilic (conjugate) addition of water onto electron‐deficient α,β‐unsaturated carbonyl substrates (e.g., maleate and aconitate hydratase^9^ or Michael‐type hydratase^10^). Unfortunately, many of these latter enzymes are encountered in primary metabolism and hence show a narrow substrate tolerance, which limits their applicability for synthesis.

Conversely, promiscuous enzymes showing a relaxed specificity for electrophiles and/or nucleophiles, whilst retaining high regio‐ and stereoselectivity or even catalyzing reactions entirely different from the “natural” ones (substrate and catalytic promiscuity, respectively)^11^ are of great interest for the evolution of novel reactivities.

**Scheme 1:**
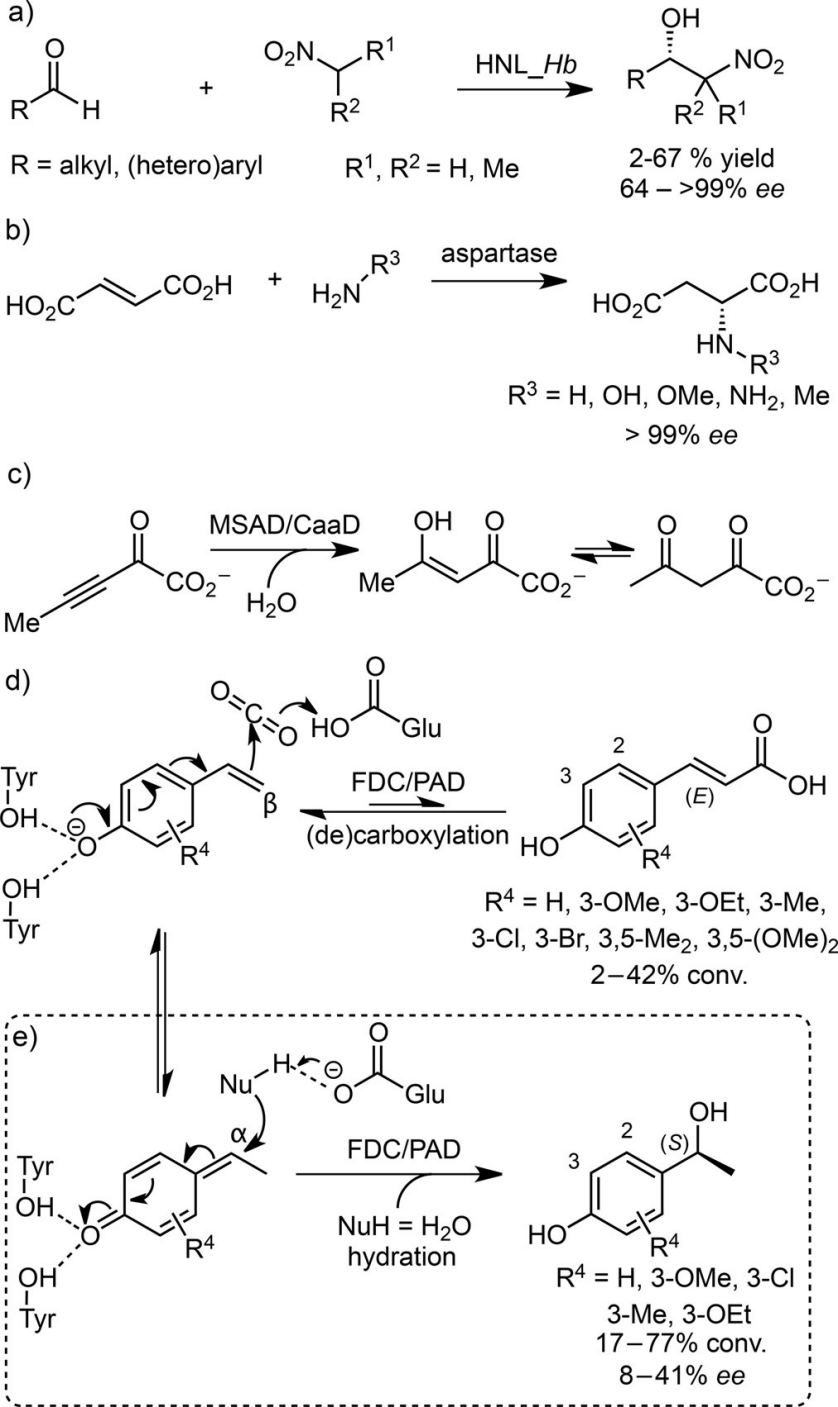
Nucleophile promiscuity of a) hydroxynitrile lyase from *Hevea brasiliensis* (HNL_*Hb*, 1,2‐addition) and b) aspartase (1,4‐addition); c) electrophile promiscuity of malonate semialdehyde decarboxylase (MSAD) and 3‐chloroacrylic acid dehalogenase (CaaD, 1,4‐addition); d) “natural” (*E*)‐selective β‐(de)carboxylation with FDC/PADs; e) 1,6‐nucleophilic α‐hydration with FDC/PADs *via* a quinone‐methide electrophile.

For example, nucleophile promiscuities of lyases include the biocatalytic variant of the Henry‐reaction catalyzed by hydroxynitrile lyases, which accept nitroalkanes (Scheme 1a),^12^ and aspartases add *prim*‐amines stereoselectively across fumaric acid yielding *N*‐substituted aspartic acid derivatives (Scheme 1b).^13^ Related promiscuities were also found in lyases (halohydrin dehalogenases), which catalyze the nucleophilic ring‐opening of epoxides by cyanide, azide, nitrite, (thio)cyanate and formate besides their “natural” co‐substrates – halides.^14^

More recently, dehalogenases (*trans*‐3‐chloroacrylic acid dehalogenase, CaaD) and decarboxylases (malonate semialdehyde decarboxylase, MSAD) were found to exhibit hydration activity on non‐natural 2‐oxo‐3‐pentynoate electrophiles (Scheme 1c)^15^ and tautomerases (4‐oxalocrotonate tautomerase, 4‐OT) turned out as suitable biocatalysts for the stereoselective Michael addition.^16^

We have recently reported on the formal asymmetric addition of water across the C=C bond of *p*‐hydroxystyrenes catalyzed by ferulic acid decarboxylase from *Enterobacter* sp. (FDC_*E*s) and related phenolic acid decarboxylases (PADs) yielding (*S*)‐configured benzylic *sec*‐alcohols (Scheme 1e).17a Hydration thus constitutes a second catalytic mode apart from the β‐(de)carboxylation of hydroxycinnamates (Scheme 1d).^17^

Based on the crystal structure from *Bacillus subtilis* (PAD_*Bs*, PDB‐ID: 4ALB)^18^ density functional theory (DFT) calculations provided a detailed understanding of the mechanism of the carboxylation of 4‐vinylphenol and its asymmetric hydration.^19^ The calculations showed that the substrate's phenolic hydroxy group is deprotonated by two interacting Tyr residues, which is followed by the generation of a quinone‐methide intermediate as a result of the C–C bond formation between Cβ and CO_2_ (Scheme 1d). In the final step, a Glu residue abstracts a proton from Cα to yield the hydroxycinnamic acid product.19b

Alternatively, a proton transfer from Glu to the Cβ position takes place first to generate a different quinone‐methide intermediate (Scheme 1e), which is intercepted by a water molecule (activated by Glu *via* a bicarbonate ion proton relay) in a 1,6‐conjugate addition yielding the (*S*)‐*sec*‐alcohol. Related modes of 1,6‐water addition onto quinone‐methide intermediates were found in vanillyl alcohol oxidase (VAO)^20^ and hydroxycinnamate‐CoA hydratase‐lyase (HCHL).^21^

Quinone‐methides serve as electrophiles for asymmetric 1,6‐conjugate additions using organocatalysts^22^ and can be generated, for example, by acid‐base or photocatalysis.^23^ Furthermore, benzylic C–H functionalization, such as hydroarylation,^24^ and hydroalkoxylations^25^ *via* quinone‐methide intermediates are enabled through palladium catalysis.

The prochiral *p*‐vinylphenol substrates may be derived *via* decarboxylation17d,^26^ or pyrolysis^27^ of coumaric acids from lignin as a renewable feedstock.

In this study, we aimed to extend the asymmetric hydration of phenolic acid decarboxylase towards “non‐natural” C‐, N‐ and S‐nucleophiles. In addition, we performed a detailed quantum mechanical study to investigate the reaction mechanism and the enantioselectivity employing cyanide as a representative nucleophile.

## Results and Discussion

### Screening of Nucleophiles

**Scheme 2:**
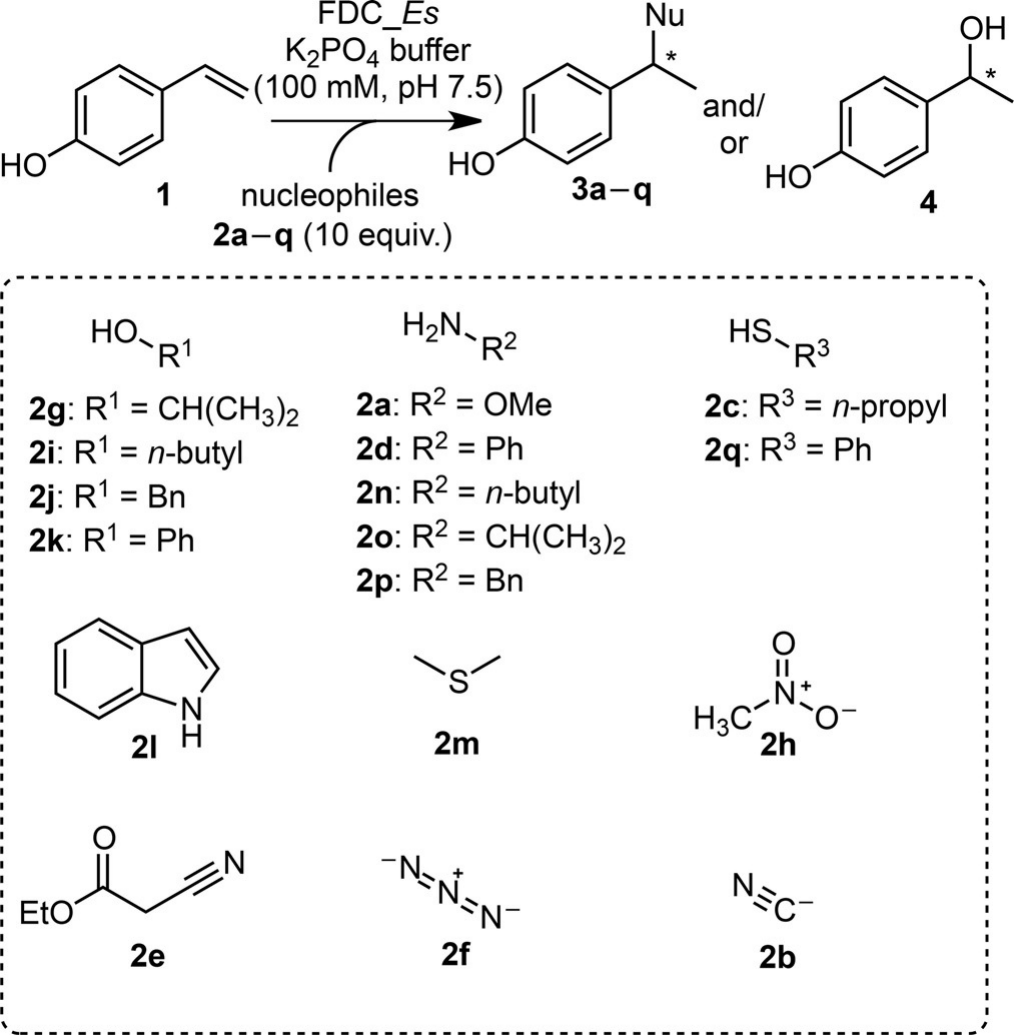
Nucleophile screening with FDC_*Es*.

In a first step, we explored the ability of FDC_*E*s to add a broad variety of nucleophiles (**2a**–**2q**) across 4‐vinylphenol (**1**) as model substrate in aqueous buffer. The screening included primary aliphatic as well as aromatic alcohols, amines, thiols, C–H acidic compounds and nucleophilic anions (Scheme 2). Based on their reactivity, nucleophiles **2a**–**2q** were qualitatively categorized into four groups (Table 1):

**Table 1:** Nucleophile screening.^[a]^

Entry	Nucleophile	Recovery of **1** [%]^[b]^	Hydration **4** [%]^[b]^	Nu‐addition **3a**–**q** [%]^[c]^
1^[d]^	**2a**	9	17	98
2	**2b**	19	<1	91
3	**2c**	14	12	95
4	**2d**	79	<1	10–35^[e]^
5	**2e**	60	30	44
6	**2f–m**	9–85	52–78	<1
7	**2n–p**	76 to >99	<1	<1
8	**2q**	5	<1	>99^[f]^

^[a]^ *Screening conditions*: lyophilized *E. coli* cells (20 mg mL^−1^) containing heterologously expressed FDC_*E*s, **1** (10 mM), **2a**–**2q** (100 mM, 10 equiv.) in KP_i_ buffer (100 mM, pH 7.5) and 1,2‐dimethoxyethane (DME, 10% v/v) as co‐solvent for water‐insoluble nucleophiles; incubation for 24 h at 30 °C with shaking at 700 rpm. Incomplete mass balance due to variations in recovery on analytical scale. ^[b]^ Recovered substrate **1** and hydration product **4** determined by HPLC‐MS using calibration. ^[c]^ Determined by GC‐MS analysis (±5%) of mass ions with *m*/*z* and fragmentation pattern matching the expected Nu‐adducts. ^[d]^ NH_3_ and methylamine were unreactive. ^[e]^ Nu addition product **3d** was formed in varying amounts. ^[f]^ Non‐enzymatic thiol‐ene reaction (32%) in absence of biocatalyst.

- With six out of 17 tested nucleophiles (**2a**–**2e**, entries 1–5, group I) FDC_*E*s yielded single products with a mass spectrum matching the expected 1,6‐addition products, only aniline (**2d**, entry 4) showed variable amounts of the expected amine product. In most cases, the competing hydration activity was largely suppressed (≤17%, entries 1–4) except for **2e** (Table 1, entry 5), which was approximately as reactive as water (44% adduct **3e**, 30% hydration **4**). The identity of the nucleophile adducts **3a**–**3c** and **3e** was confirmed by co‐injection of independently synthesized reference material on both HPLC and GC‐MS (Supporting Information, Sections 4 and 7).
- In contrast to group I, none of the nucleophiles of group II (**2f**–**2m**, entry 6) underwent 1,6‐addition and only hydration was observed.
- Group III lists nucleophiles that abolished any activity (**2n**–**2p**, entry 7).
- Thiophenol (**2q**, entry 8) underwent enzyme‐catalyzed 1,6‐addition yielding **3q**, but also participated in a non‐enzymatic thiol‐ene reaction with **1**, together with spontaneous oxidization to its disulfide.

Control experiments in the absence of biocatalyst or using *E. coli* expression host cells lacking the respective decarboxylase gene proved the requirement of FDC_*E*s for product formation and excluded spontaneous background reactions (except for **2q**).

### Screening of Enzymes

In order to expand the enzyme toolbox for the 1,6‐nucleophile addition, a set of PADs identified by a BLAST‐search17a with 48–75% sequence identity to FDC_*E*s (Supporting Information Figure S1, Table S1) possessing conserved catalytically relevant residues for hydration and carboxylation (Tyr19, Tyr21, Glu72 and Arg49)^19^ was tested.

**Scheme 3:**
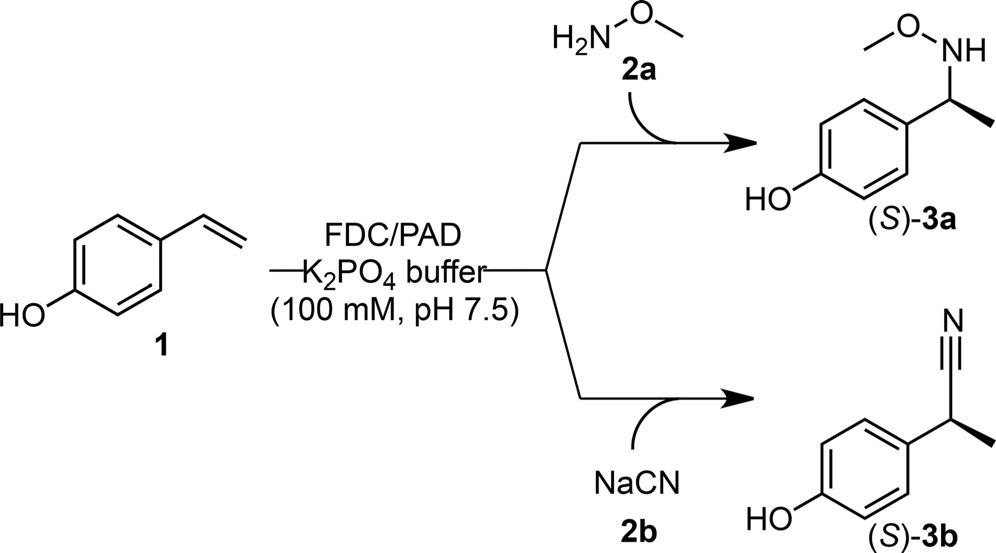
Conjugate 1,6‐addition of methoxyamine **2a** and cyanide **2b**.

Among the first group of nucleophiles (Table 1, entries 1–5), methoxyamine (**2a**), cyanide (**2b**) and *n*‐propanethiol (**2c**) were readily accepted by FDC_*E*s (Table 1), while **2d** and **2e** were less promising due to low reproducibility (**2d**) or due to spontaneous background reactions (**2q**). Hence, the former were chosen for the enzyme screening (Scheme 3, for the screening with **2c** see the Supporting Information, Table S4). All tested decarboxylases catalyzed the formation of nucleophile adducts **3a** and **3b** beside the minor hydration product **4** (Table 2) with the single exception of PAD_*Mc* and **2a** (Table 2, entry 6). However, distinct variations in conversion and optical purity of the products (*S*)‐**3a** and (*S*)‐**3b** were noted.

**Table 2:** Enzyme screening for the addition of **2a** and **2b** onto **1**.^[a]^

Nucleophile		**2a**						**2b**			
Enzyme	Entry	**3a** [%]	*ee* [%]	**4** [%]	*ee* [%]		Entry	**3b** [%]	*ee* [%]	**4** [%]	*ee* [%]
FDC_*E*s	1	93	17	6	39		8	82	64	4	n.d.
PAD_*Lp*	2	48	3	6	10		9	5	68	<1	n.d.
PAD_*Ba*	3	11	*rac*	4	n.d.		10	25	88	1	n.d.
PAD_*Ll*	4	42	5	6	24		11	29	76	<1	n.d.
PAD_*M*s	5	15	8	2	n.d.		12	23	85	<1	n.d.
PAD_*Mc*	6	<1	n.d.	3	n.d.		13	6	71	8	89
PAD_*P*s	7	73	10	8	26		14	57	91	<1	n.d.

^[a]^ Abbreviations: FDC_*Enterobacter* sp. (*E*s), PAD_*Pantoea* sp. (*P*s), PAD_*Mycobacterium columbiense* (*Mc*), PAD_*Methylobacterium* sp. (*M*s), PAD_*Lactobacillus plantarum* (*Lp*), PAD_*Lactococcus lactis* (*Ll*) and PAD_*Bacillus amyloliquefaciens* (*Ba*). *Screening conditions*: lyophilized *E. coli* cells (20 mg mL^−1^) containing the heterologously expressed FDC or PAD, **1** (10 mM), **2a** or **2b** (100 mM) in KP_i_ buffer (50 mM, pH 7.0); incubation for 24 h at 30 °C and 700 rpm; n.d.=not determined due to low conversion.

In the addition of methoxyamine (**2a**), FDC_*E*s showed the best results, both in terms of conversion and enantioselectivity (17% *ee*) (Table 2, entry 1). PAD_*Lp*, PAD_*Ll* and PAD_*Ps* showed moderate conversions of 42–73% (Table 2, entries 2, 4 and 7) and poor optical purity of (*S*)‐**3a** (*ee* ≤10%). Hydration was largely reduced and showed only moderate *ee*s of **4** (max. 39% with FDC_*Es*).

Similarly, addition of cyanide (**2b**) proceeded with moderate to good conversion, but stereoselectivities were generally much better with all enzymes (Table 2, entries 1–7 *vs*. entries 8–14). FDC_*E*s and PAD_*P*s performed best in terms of conversion (Table 2, entries 8 and 14) and the latter enzyme also exhibited superior stereoselectivity in the addition of cyanide (91% *ee*, Table 2, entry 14) thus promoting it as a promising candidate for further investigations. Again, with **2b** hydration was only a minor side reaction (max. 8% with PAD_*Mc*).

The absolute configuration of products **3a**–**3c** was determined by comparison with authentic reference material (**3a**), comparison of optical rotation values (**3b**) and CD spectroscopy (**3c**) as described in the Supporting Information (Table S5). Overall, a strong preference for the formation of the (*S*)‐product is congruent for all enzymes.

Given the comparatively high sequence identity of 73% between PAD_*P*s and FDC_*E*s (other PADs show 48–52% identity, Supporting Information, Table S1) it is plausible that these two enzymes also perform similarly in the addition of nucleophiles.

**Figure 1:**
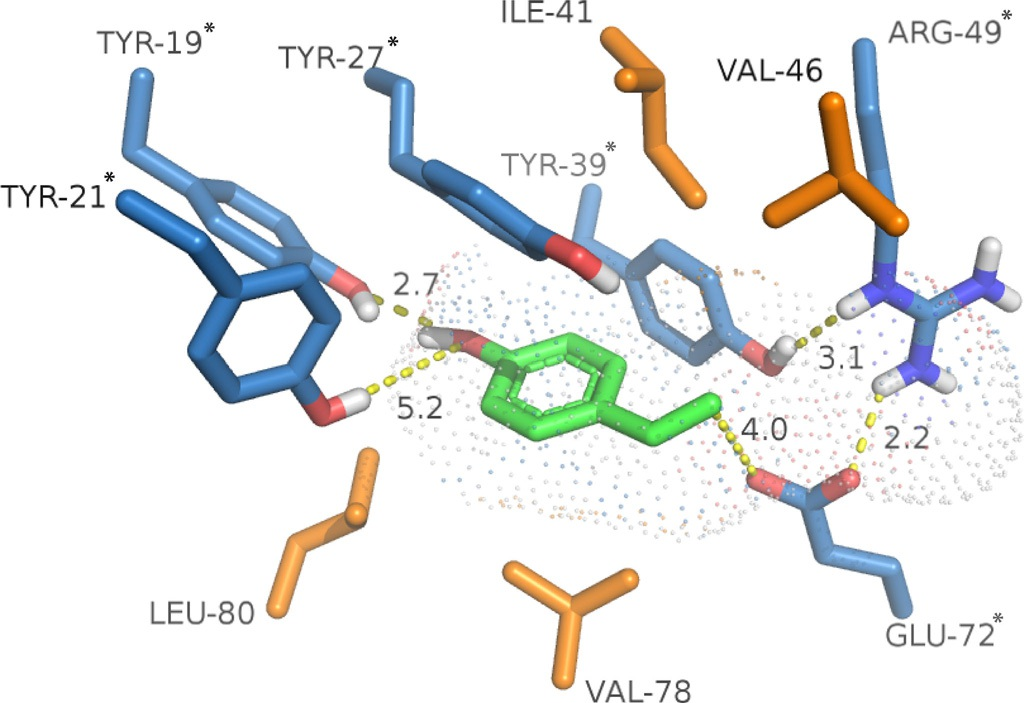
Active‐site of FDC_*E*s (PDB‐ID: 4UU3)^28^ with *p*‐vinylphenol (**1**) docked (green, docking performed with UCSF Chimera);^29^ residues targeted by mutagenesis for improvement of catalysis are highlighted in orange. (Putative) catalytic key residues are marked with an asterisk.

In addition to wild‐type enzymes, FDC_*E*s variants (Figure 1) were tested with nucleophiles **2a** and **2b** (Table 3). Mutants designed to provide more space in the active site led to considerably less conversion (L80A, L80A/V78A; Table 3, entries 3, 4, 12 and 13) or were inactive at all (L80A/V78A/W70V, L80A/V78A/ W70L, L80A/V78A/W70L/V46A; data not shown). However, exchange of Ile to Ala in position 41 appeared to be beneficial (Figure 1) since with both nucleophiles, the conversion was not adversely affected, but the *ee* was significantly enhanced (entries 2 and 11).

**Table 3:** FDC_*E*s mutants in the addition of **2a** and **2b**.^[a]^

Nucleophile	**2a**						**2b**				
FDC_*E*s variant	Entry	**3a** [%]	*ee* [%]	**4** [%]	*ee* [%]		Entry	**3b** [%]	*ee* [%]	**4** [%]	*ee* [%]
wt	1	95	17	5	39		10	82	64	<1	n.d.
I41A	2	96	36	4	*rac*		11	81	81	<1	n.d.
L80A	3	71	*rac*	3	*rac*		12	23	72	<1	n.d.
L80A/V78A	4	10	*rac*	<1	n.d.		13	53	85	<1	n.d.
Y27F	5	94	8	6	*rac*		14	55	82	<1	n.d.
Y39F	6	95	*rac*	5	12		15	16	72	<1	n.d.
Y19F	7	<1	n.d.	<1	n.d.		16	<1	n.d.	<1	n.d.
Y21F	8	<1	n.d.	<1	n.d.		17	3	n.d.	<1	n.d.
E72A	9	8	*rac*	<1	n.d.		18	14	14	<1	n.d.

^[a]^ *Conditions*: see Table 2; n.d.=not determined due to low conversion.

In order to evaluate the relevance of tyrosine residues 27 and 39, which are flanking the substrate, the corresponding Phe‐variants were prepared (Y27F, Y39F). With **2a**, the activity and selectivity were not affected (entries 6 and 7) but with **3b** conversion dropped significantly going in hand with enhanced selectivities (entries 14 and 15).

Changing Glu72 (responsible for CO_2_‐activation in the carboxylation reaction) to alanine or either of Tyr19 and Tyr21 (for deprotonation of the phenolic OH)19b to phenylalanine completely abolished the activity for nucleophile addition as well as hydration (Table 3, entries 7–9 and 16–18), which underlines their crucial role in catalysis.

### Preparative‐Scale Biotransformation

In order to fully characterize products **3a** and **3b** and to evaluate the applicability of this biotransformation for the preparative scale, reactions were performed with 50 to 100 mg substrate after optimization of the reaction conditions. Promising initial results (Table 1) and conversions of up to 73% in an enzyme screening (Supporting Information, Table S4) encouraged us to include also propanethiol (**2c**) in the up‐scales.

Given the heterogeneity of p*K*_a_ values of the nucleophiles and their pH‐dependent reactivity, a detailed pH‐study was performed and the maximum of substrate‐ and nucleophile‐loading was evaluated. Dimethoxyethane (DME) was identified as a suitable co‐solvent for water‐insoluble nucleophile **2c** (Supporting Information, Section 5). Optimal results are listed in Table 4. After isolation and purification of the nucleophile adducts (**3a**–**c**) (Table 4), the absolute configuration of all products was determined to be (*S*) (Supporting Information, Table S5) and hence nicely matched the stereoselectivity of the biocatalytic hydration.17a

**Table 4:** Preparative‐scale biotransformation, isolation and characterization of products.^[a]^

	FDC_*E*s variant	Yield [%]	Yield [mg]	*ee* [%]	[α]20D
**3a**	I41A	71^[b]^	71	22 (*S*)	−6.2°
**3b**	wt	71^[c]^	44	85 (*S*)	−12°
**3c**	wt	56^[d]^	56	81 (*S*)	−156°

^[a]^ *Conditions*: 20 mg mL^−1^ lyophilized *E. coli* whole cells with heterologously expressed FDC_*Es* variants in KP_i_‐buffer (100 mM). ^[b]^ **1** (20 mM), **2a** (5 equiv.), pH 7.0. ^[c]^ **1** (10 mM), **2b** (10 equiv.), pH 6.0. ^[d]^ **1** (10 mM), TAPS‐buffer pH 8.5, DME (10% v/v), **2c** (10 equiv.).

### Quantum Mechanical Mechanistic Investigations

**Figure 2:**
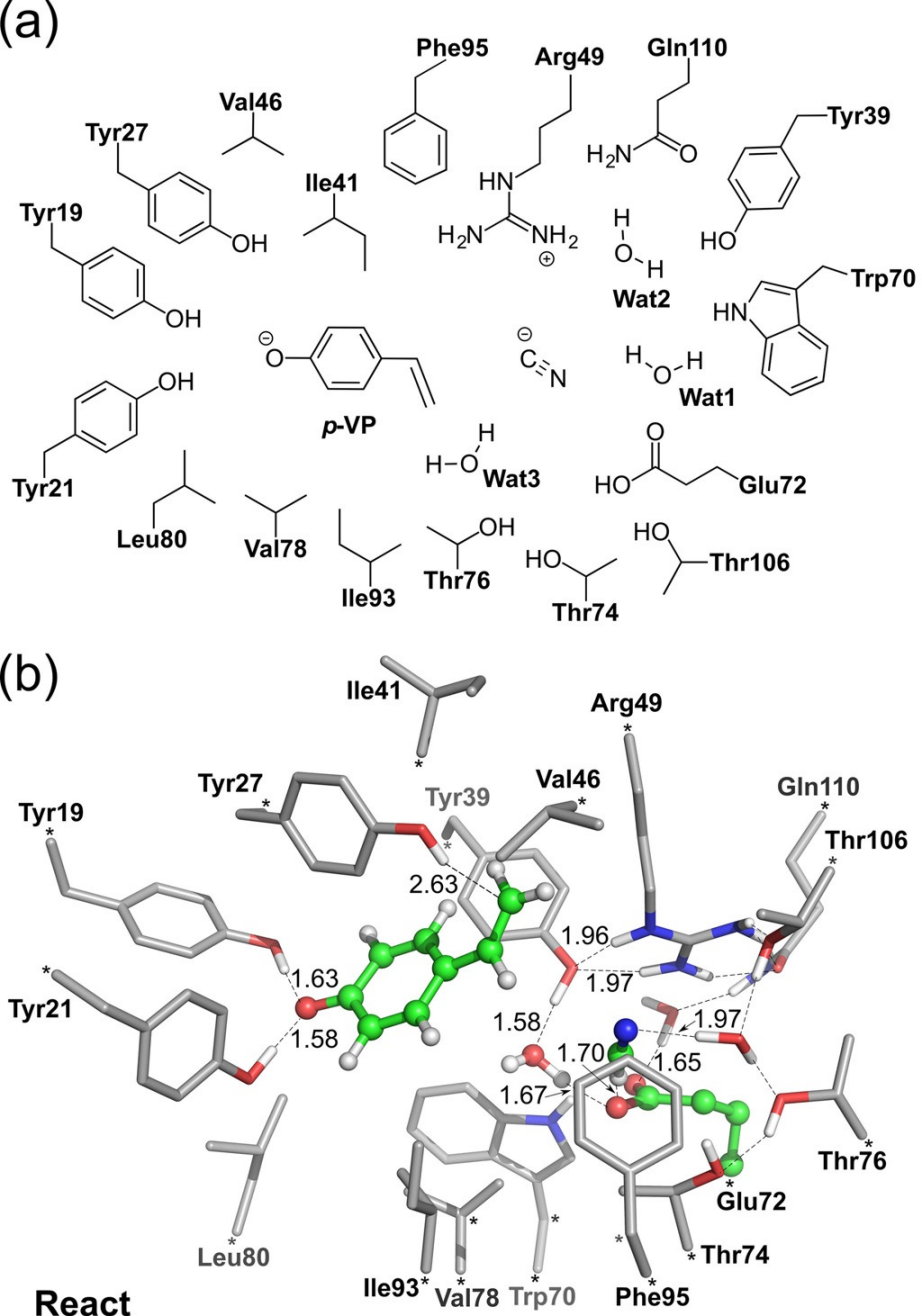
(a) Schematic illustration of the active site model employed in the computational study. (b) Optimized structure of **React**, which corresponds to the lowest energy among the enzyme‐substrate complexes considered. During the geometry optimization of **React**, a proton moves spontaneously from Glu72 to the cyanide anion. Atoms with asterisks were fixed during geometry optimization. Distances are given in Å. For clarity, only substrate hydrogens and polar hydrogens are shown.

To shed more light on the mechanism and the origin of stereoselectivity in the asymmetric nucleophile addition, DFT calculations were undertaken with cyanide as representative nucleophile using the active‐site model based on PAD_*Bs* (Figure 2a, amino acid numbers were adjusted by +8 to fit the sequence of FDC_*E*s).

The total size of the model comprised 309 atoms and the overall charge was −1. In analogy to the previous studies,^19^ the hydroxy group of *p*‐vinylphenol was assumed to be deprotonated upon binding to the tyrosine residues Tyr19 and Tyr21, while Glu72 was modeled in its protonated state. Since the substrates can bind to the active site in many different ways, a large number of structures of the enzyme‐substrate complex (>40) have been optimized. The structure with the lowest energy (called **React**) is shown in Figure 2b.

**Scheme 4:**
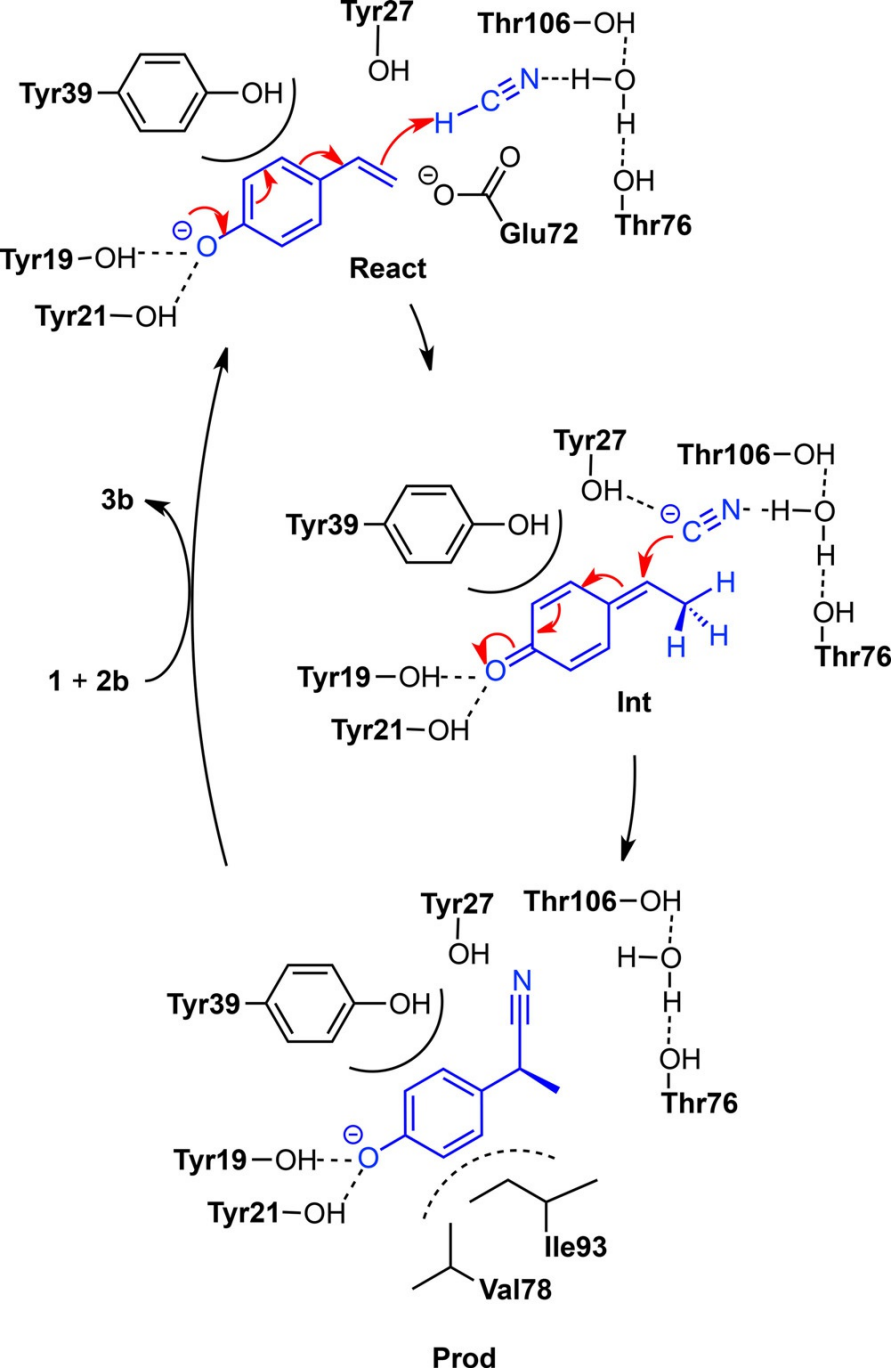
Proposed catalytic mechanism for the stereoselective addition of cyanide (**2b**) across 4‐hydroxystyrene (**1**).

Interestingly, in the geometry optimization of the enzyme‐substrate complex, cyanide was found to spontaneously abstract a proton from Glu72 to form HCN (Figure 2b). The calculations suggest that cyanide addition involves a quinone‐methide intermediate in analogy to that proposed for hydration.19a The reaction starts with a proton transfer from hydrogen cyanide to the β‐carbon of the substrate, forming the quinone‐methide. The second step is a nucleophilic attack of the resulting cyanide at the α‐carbon to generate the product (Scheme 4).

**Figure 3:**
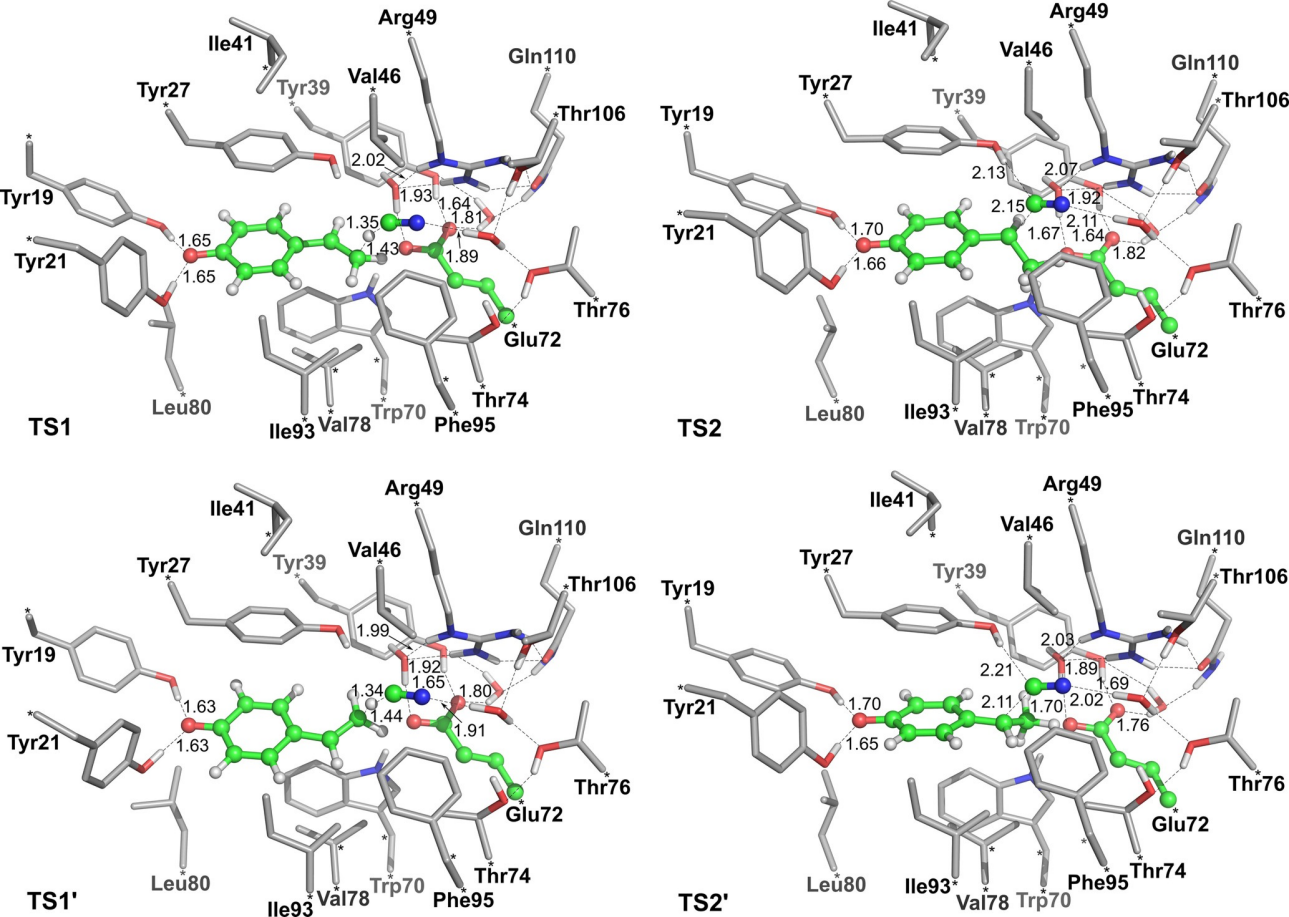
Optimized structures of the transition states involved in the lowest energy pathways leading to (*S*)‐product (**TS1** and **TS2**) and (*R*)‐product (**TS1′** and **TS2′**).

**Figure 4:**
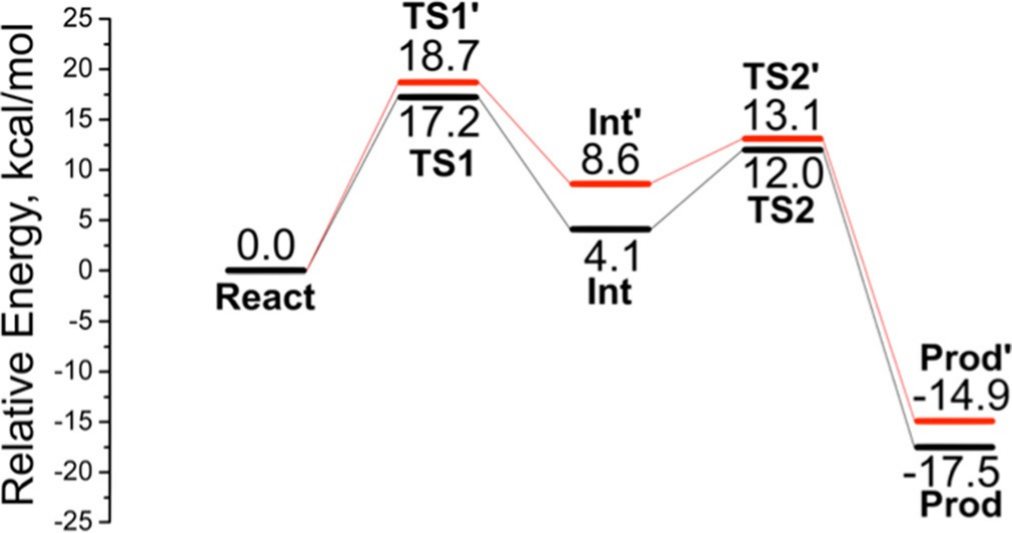
Calculated energy profile for the PAD‐catalyzed addition of cyanide to *p*‐vinylphenol. The lowest‐energy pathways leading to both the (*S*)‐product (black line) and (*R*)‐product (red line) are shown.

The optimized geometries of two transition states are given in Figure 3 (geometries of other stationary points are given in the Supporting Information, Figure S46) and the calculated energy profile is shown in Figure 4. In order to ensure that the lowest energy barrier is obtained, we followed the reaction paths starting from the six lowest‐energy Michaelis complexes.

Proton transfer onto Cβ was calculated to be the rate‐limiting step with a barrier of +17.2 kcal mol^−1^, and the resulting quinone methide (**Int**) is 4.1 kcal mol^−1^ higher than **React**. The subsequent nucleophilic attack has a rather low barrier of +7.9 kcal mol^−1^ relative to **Int**, and the entire reaction is calculated to be exothermic by 17.5 kcal mol^−1^. In the optimized transition state structure for proton transfer (**TS1**), the bond distances of the breaking and the forming H–C bonds are 1.35 Å and 1.43 Å, respectively. Cyanide was located in the vicinity of Tyr27 in **TS1**. A hydrogen bond is formed between cyanide and Tyr27 in **Int**, and this interaction is maintained during the nucleophilic attack. Interestingly, we obtained also optimized structures of stationary points in which the hydroxy group of Tyr27 points away from cyanide (see the Supporting Information, Figure S47 for geometries and relative energies), however, the barriers for the two steps are higher by 2–4 kcal mol^−1^, showing that Tyr27 is important for the reaction (*cf*. Table 3, entry 14). The importance of Tyr27 in the (de)carboxylation has been addressed in previous studies.^19^

Experimentally, the *ee* of (*S*)‐**3b** is 64–91%, which corresponds to an energy difference of 1–2 kcal mol^−1^ between the barriers leading to the enantiomeric products. The reaction pathway with the lowest energy barriers discussed above favors the generation of the (*S*)‐product, which nicely corroborates the experimental data in Table 2 and Table 3.

To investigate the origin of the observed selectivity, we have also optimized the geometries of the transition states for the pathway leading to the (*R*)‐enantiomer (termed **TS1′** and **TS2′**, Figure 4). Indeed, both transition states were found to have higher energies compared to those of the (*S*)‐pathway. The barrier for proton transfer in the lowest‐energy pathway leading to the (*R*)‐enantiomer is 18.7 kcal mol^−1^, which is 1.5 kcal mol^−1^ higher than that for the (*S*)‐enantiomer, while the barrier for the C–C bond formation is calculated to be 13.1 kcal mol^−1^, compared to 12.0 kcal mol^−1^ for the (*S*)‐enantiomer. The calculations confirm the experimental stereoselectivities very well.

Analyzing the geometries of the transition states leading to (*S*)‐ and (*R*)‐enantiomers (Figure 3), we note that the phenoxide group of the substrate is anchored by Tyr19 and Tyr21. On the other hand, the cyanide nucleophile is positioned by Wat1, which in turn forms hydrogen bonds with Thr76 and Thr106. The main difference between the transition states in two pathways is which face of the substrate is exposed to the cyanide nucleophile, that is, where the methylene group of the substrate is pointing. In **TS1** and **TS2** [favoring (*S*)‐**3b**], the methylene group points toward the side‐chains of Val78 and Ile93, while in **TS1′** and **TS2′** [favoring (*R*)‐**3b**] the methylene points toward a more crowded area where the Tyr39 side chain is located. Similar interactions were concluded to be responsible for the stereoinduction in the hydration reaction.19a

## Conclusions

In conclusion, various soft amine‐, thiol‐ and carbon‐nucleophiles were accepted in the enzyme‐catalyzed 1,6‐conjugate addition across 4‐vinylphenol (**1**) to give the corresponding amines **3a** and **3d**, thioether **3c**, nitrile **3b** and cyano‐ester **3e** with conversions up to 95% and moderate to good optical purities (91% *ee*) with a strong preference for (*S*)‐products in case of cyanide and propanethiol. Furthermore, several FDC_*E*s variants and related phenolic acid decarboxylases were identified as suitable biocatalysts for the addition reactions.

Quantum mechanical calculations revealed details on the mechanism and identify steric interactions responsible for the stereochemical outcome.

## Experimental Section

### Materials and Methods

Model substrate 4‐vinylphenol **1** was obtained from Sigma Aldrich as 10% w/w solution in propylene glycol. The actual content was calculated to be 8.6% w/w from ^1^H NMR signals from propylene glycol and 4‐vinylphenol, respectively. Nucleophilic compounds were obtained from commercial sources: **2a** (hydrochloride), **2c**, **2h**, **2i**, **2j**, **2k**, **2l**, **2n**, **2o** were from Sigma Aldrich, **2e**, **2m**, **2p**, **2q** from Fluka, **2d** and **2f** from Lancaster and **2g** from Roth, and were used as received unless otherwise stated. The stock solution containing **2a** hydrochloride was neutralized with equimolar amounts of solid KOH. Compound **2e** and other Michael donors were purified by bulb‐to‐bulb distillation. 1,2‐Dimethoxyethane was from Sigma Aldrich. Buffer salts KH_2_PO_4_ and TAPS were acquired from Aldrich, and K_2_PO_4_ was purchased from Roth. Analytical TLC was performed on aluminum plates (silica gel 60 F_254_) from Merck, compounds were visualized by UV (*λ*=254 nm) and/or by staining with cerium molybdenum solution [phosphomolybdic acid (25 g), CeSO_4_⋅2 H_2_O (10 g), conc. H_2_SO_4_ (60 mL), H_2_O (940 mL)] or potassium permanganate solution [KMnO_4_ (1.5 g), K_2_CO_3_ (10 g), NaOH (aq. 10%, 1.25 mL), H_2_O (200 mL)]. For preparative silica gel column chromatography Merck silica gel 60 was used. Petroleum ether (boiling fraction between 40–60 °C) and ethyl acetate for chromatographic separations were freshly distilled. NMR spectra were recorded on a 300 MHz Bruker Avance III, chemical shifts (*δ*) are given in ppm relative to the solvent signal and coupling constants (*J*) are given in Hz. GC‐MS analytics were performed on an Agilent 7890A gas chromatograph equipped with an Agilent HP‐5 MS column (30 m×0.25 mm×0.25 μm film) using He at 0.55 mL min^−1^ as carrier gas in combination with an Agilent 5975C quadrupole mass detector operated in ESI^+^ mode (70 eV). Standard temperature method: initial hold at 100 °C for 30 sec, 10 °C min^−1^ to 300 °C. Samples from biotransformations diluted with acetonitrile were analyzed on an Agilent 1290 Infinity HPLC equipped with a Phenomenex Luna column (C18, 100A, 250×4.6 mm×5 μm) and a DAD detector at 25 °C using the following method: flow 1 mL min^−1^; mobile phase A: water+0.1% v/v TFA, B: acetonitrile+0.1%v/v TFA; 0–2 min (100% A), 2–15 min (100–60% A), 15–20 min (60–0% A), 20–22 min (0% A), 22–24 min (0%–100% A), 24–25 min (100% A). Quantification of the reaction constituents was performed at 270 nm after calibration with 4‐vinylphenol **1**, hydrate **4** and nucleophile adducts **3a**–**3c** within a range of 10–0.5 mM using anisole as internal standard. Enantiomeric excesses (*ee*) of **3a** and **4** were measured on a Chiralcel OD‐H column (0.46×25 cm, Daicel) with a Shimadzu HPLC System using an isocratic mixture of heptane/2‐propanol 93:7 at a flow of 1 mL min^−1^ at 30 °C column temperature; **3c** was measured with the same system using an isocratic composition of heptane/2‐propanol 98.5:1.5; **3b** was measured after acetylation (acetic anhydride, DMAP) of the phenolic hydroxy group on an Agilent 7890A gas chromatograph equipped with a DEX‐CB column (25 m×0.32 mm, 0.12 μm) and a FID using hydrogen (1.3 mL min^−1^) as carrier gas with the following temperature program: 100 °C (hold 1 min), 10 °C min^−1^ to 160 °C (hold 6 min), 20 °C min^−1^ to 180 °C (hold 1 min). Optical rotation was measured at 20 °C on a Perkin–Elmer Polarimeter 341 (sodium D‐line *λ*=589 nm).

### Preparation of Biocatalysts: Cloning and Heterologous Expression

The genes encoding for the respective PADs and FDC were transformed in *E. coli* BL21(DE3) and heterologously expressed as described previously.17a

### Site‐Directed Mutagenesis

Site‐directed mutagenesis was carried out with the QuikChange PCR mutagenesis kit from Stratagene using the respective primer sequences listed in the Supporting Information, Table S3.

### Nucleophile Screening

Lyophilized *E. coli* whole cells containing heterologously expressed FDC_*E*s (20 mg) were rehydrated for 30 min at 700 rpm shaking in KP_i_ buffer (887 μL; 100 mM, pH 7.6) in 2.0 mL reaction vials. A stock solution containing the respective nucleophile in either buffer or 1,2‐dimethoxyethane (DME), depending on the compound's solubility (100 μL, 1 M), was added to the cell suspension followed by short mixing and addition of the substrate 4‐vinylphenol **1** (13.4 μL of a 8.6% w/w solution in propylene glycol). The mixture was incubated for 24 h at 30 °C and 700 rpm in an Eppendorf Thermoshaker. Then the mixture was split into two equal aliquots á 500 μL. One aliquot was extracted with ethyl acetate (2×500 μL) and after drying over MgSO_4_ the organic phase was subjected to GC‐MS analysis. The other half was diluted with acetonitrile containing anisole as internal standard (10 mM) for quantification of substrate **1** and hydrate **4** using HPLC. Putative adducts were identified by means of MS fragmentation patterns.

### Enzyme Screening

Lyophilized *E. coli* whole cells containing the heterologously expressed PAD (wild‐type or mutants, 20 mg) were rehydrated for 15 min at 700 rpm in KP_i_ buffer (50 mM) in 2.0 mL reaction vials containing 100 mM of methoxyamine **2a** (1 mL, pH 7.0). Substrate 4‐vinylphenol **1** (13.4 μL of a 8.6% w/w solution in propylene glycol) was added and the mixture was incubated for 24 h at 30 °C and 700 rpm in an Eppendorf Thermoshaker. The reaction was stopped by the addition of acetonitrile (1 mL) containing anisole as internal standard (10 mM), vortexing and centrifugation (14000 rpm, 10 min). An aliquot (1 mL) was withdrawn for quantitative aqueous HPLC analytics. The remaining aliquot was extracted with ethyl acetate (2×500 μL) and after drying over MgSO_4_ the combined organic extracts were evaporated to dryness in an air stream, re‐dissolved in 2‐PrOH (100 μL), diluted with *n*‐heptane (900 μL) and subjected to chiral analytics with organic HPLC.

### Preparative‐Scale Biotransformations

**General procedure for the FDC‐catalyzed preparative‐scale addition of 2a**–**2c onto 1**: Lyophilized *E. coli* whole‐cells containing the heterologously expressed FDC_*E*s wild‐type or I41A mutant (20 mg mL^−1^) were rehydrated in KP_i_ buffer for 30 min and 120 rpm at 30 °C in a cultivation shaker in a 50 mL Falcon tube. Nucleophiles **2a**–**2c** were supplemented from a stock solution in reaction buffer or DME (1 M) followed by the addition of substrate **1** (8.6% w/w solution in propylene glycol), the mixture was incubated at 30 °C and 120 rpm for the denoted time. The reaction was stopped by extraction with ethyl acetate (3×20 mL), the combined organic extracts were dried over Na_2_SO_4_ and the solvent was removed under reduced pressure.

**(*S*)‐4‐(1‐Methoxyamino)ethylphenol (3a)**: Substrate **1** (803.4 μL stock solution, 0.6 mmol, 20 mM) was reacted with **2a** (2.99 mL stock solution in buffer, 5 equiv., 100 mM) in KP_i_ buffer (50 mM, pH 7.0, 26.1 mL) for 26 h. Pure product **3a** was obtained after column chromatography on silica gel [petroleum ether/ethyl acetate 7:3, *R*_f_=0.30 (product **3a**)] as an oil; yield: 70.6 mg (71%). ^1^H NMR (300 MHz, CDCl_3_): *δ*=7.24–7.16 (m, 2 H), 6.78–6.73 (m, 2 H), 4.09 (q, ^3^ *J*_1_=6.6 Hz, 1 H), 3.50 (s, 3 H), 1.36 (d, ^3^ *J*_1_=6.6 Hz, 3 H); ^13^C NMR (75 MHz, CDCl_3_): *δ*=155.3, 134.3, 128.6 (2 C), 115.5 (2 C), 62.5, 59.9, 19.6; GC‐MS (ESI^+^, 70 eV): *t_R_*=8.70, *m*/*z=*167.1 [M^+^]; HR‐MS (CI^+^): *m*/*z=*168.10173 [MH^+^] (calcd. 168.10191); [α]20D : −6.2 (*c* 1, CHCl_3_, *ee*=22%, [*S*]).

**(*S*)‐2‐(4‐Hydroxyphenyl)propanenitrile (3b)**: Substrate **1** (457 μL stock solution, 0.33 mmol, 10 mM) was reacted with **2b** (3.4 mL stock solution in buffer, 10 equiv., 100 mM) in KP_i_ buffer (50 mM, pH 6.0, 30 mL) for 40 h. Pure product **3b** was obtained after column chromatography on silica gel [petroleum ether/ethyl acetate 7:3, *R*_f_=0.57 (product **3b**)] as a colorless oil that solidified upon storage at 4 °C; yield: 43.8 mg (89%). ^1^H NMR (300 MHz, CDCl_3_): *δ*=7.22–7.18 (m, 2 H), 6.86–6.81 (m, 2 H), 5.58 (bs, 1 H), 3.85 (q, ^3^ *J*_1_=7.2 Hz, 1 H), 1.61 (d, ^3^ *J*_1_=7.2 Hz, 3 H); ^13^C NMR (75 MHz, CDCl_3_): *δ*=155.8, 128.8, 128.1 (2 C), 122.1, 116.1 (2 C), 30.6, 21.5; GC‐MS (ESI^+^, 70 eV): *t_R_*=8.78 min, *m*/*z=*147.1 [M^+^]; HR‐MS (CI^+^): *m*/*z=*148.07555 [MH^+^] (calcd. 148.07569); [α]20D : −12.5 (*c* 1, CHCl_3_, *ee*=85%, [*S*]).

**(*S*)‐4‐[1‐(Propylthio)ethyl]phenol (3c)**: Substrate **1** (684 μL stock solution, 0.5 mmol, 10 mM) was reacted with **2c** (5.0 mL stock solution in DME, 10 equiv., 100 mM) in TAPS buffer (100 mM, pH 9, 45 mL) for 24 h. Pure product **3c** was obtained after column chromatography on silica gel [petroleum ether/ethyl acetate 7:3, *R*_f_=0.57 (product **3c** co‐elutes with **1**!)] as a colorless oil; yield: 56 mg (58%). ^1^H NMR (300 MHz, CDCl_3_): *δ*=7.23–7.18 (m, 2 H), 6.80–6.75 (m, 2 H), 5.03 (bs, 1 H), 3.91 (q, ^3^ *J*_1_=6.9 Hz, 1 H), 2.36–2.21 (m, 2 H), 1.56–1.45 (m, 5 H), 0.91 (t, ^3^ *J*_1_=7.5 Hz, 3 H); ^13^C NMR (75 MHz, CDCl_3_): *δ*=154.4, 136.5, 128.6 (2 C), 115.4 (2 C), 43.5, 33.3, 22.8, 13.7; GC‐MS (ESI^+^, 70 eV): *t_R_*=10.75 min, *m*/*z=*196.1 [M^+^]; HR‐MS (CI^+^): *m*/*z=*197.09602 [MH^+^] (calcd. 197.09946); [α]20D : −156.2 (*c* 1, CHCl_3_, *ee*=81%, [*S*]).

### QM Calculation Details

All the calculations were performed using the B3LYP density functional method,^30^ as implemented in the Gaussian 09 program.^31^ Geometries were optimized with the 6‐31G(d,p) basis set and more accurate energies were obtained by single‐point calculations on the optimized structures with the larger basis set 6–311+G(2d,2p). Single‐point solvation energies with SMD method^32^ were calculated at the same level as the geometry optimization using *ϵ*=4. Frequency calculations were performed the same level as the geometry optimization to obtain zero‐point energies (ZPE). Dispersion corrections were added using the DFT‐D3(BJ) method.^33^

## Supporting information

As a service to our authors and readers, this journal provides supporting information supplied by the authors. Such materials are peer reviewed and may be re‐organized for online delivery, but are not copy‐edited or typeset. Technical support issues arising from supporting information (other than missing files) should be addressed to the authors.

Supplementary
