# Supplementary material for: Exploring the Catalytic Promiscuity of Phenolic Acid Decarboxylases: Asymmetric, 1,6‐Conjugate Addition of Nucleophiles Across 4‐Hydroxystyrene
Source: Adv Synth Catal. 2017 May 8;359(12):2066–75. doi: 10.1002/adsc.201700247 (PMC5488193; doi:10.1002/adsc.201700247)
Supplement: Supplementary file 1 — Supplementary [file ADSC-359-2066-s001.pdf]

Advanced Synthesis & Catalysis

Supporting Information

Exploring the Catalytic Promiscuity of Phenolic Acid Decarboxylases: Asymmetric, 1,6-Conjugate Addition of Nucleophiles Across 4-Hydroxystyrene

Stefan E. Payer[b], Xiang Sheng[c], Hannah Pollak[c], Christiane Wuensch[a,b], Georg Steinkellner[a,d], Fahmi Himo[c] and Silvia M. Glueck[a,b] and Kurt Faber[b]*

a Austrian Centre of Industrial Biotechnology (ACIB), c/o
b Department of Chemistry, University of Graz, Heinrichstrasse 28, A-8010 Graz, Austria
c Arrhenius Laboratory, Department of Organic Chemistry, Stockholm University, SE-106 91 Stockholm, Sweden
d Center for Molecular Biosciences, University of Graz, Humboldtstrasse 50, 8010 Graz, Austria.

* Corresponding author: ph.: +43 316 380 5332, fax: +43 316 380 9840, e-mail <Kurt.Faber@Uni-Graz.at>

Supporting Information

1. General information
2. Sequence alignment of PADs and FDC_*E*s
3. Cloning and expression of PADs and FDC, primer sequences for site-directed mutagenesis
4. Synthesis of racemic and non-racemic reference material
5. Optimization of biocatalytic process: pH, substrate / nucleophile loading
6. Determination of absolute configuration of products
7. Analytical methods and GC-MS, GC-FID, HPLC, HR-MS chromatograms and NMR spectra.
8. Quantum mechanical calculations
9. References

1. General Information

Solvents and reagents were obtained from commercial sources and used as received unless otherwise stated. 4-Vinylphenol **1** was obtained from Sigma Aldrich as 10% w/w solution in propylene glycol. The actual content was determined as 8.6 % w/w from of ^{1}H-NMR integrals from propylene glycol and 4-vinylphenol, respectively. Nucleophiles were obtained from commercial sources [**2a** (as hydrochloride), **2c**, **2h**, **2i**, **2j**, **2k**, **2l**, **2n**, **2o** from Sigma Aldrich; **2e**, **2m**, **2p**, **2q**, from Fluka; **2d**, **2f** from Lancaster and **2g** from Roth] and were used as received unless otherwise stated. The stock containing **2a** hydrochloride was neutralized with equimolar amounts of solid KOH. Compound **2e** was purified prior to use by bulb-to-bulb distillation. 1,2-Dimethoxyethane and buffer salts (Na citrate, KH_2PO_4, TAPS) were acquired from Sigma Aldrich, and K_2PO_4 was purchased from Roth. Enantiomerically enriched (*S*)- and (*R*)-**5a** was provided by BASF SE.

Preparative chromatographic separations were performed by column chromatography on Merck silica gel 60 (0.063–0.200 mm). Solvents for flash chromatography (petroleum ether bp 40–60 °C/EtOAc) were distilled before use. TLC was carried out with pre-coated aluminium sheets (TLC Silica gel 60 F254, Merck) with detection at UV (254 nm) and/or by staining with ceric molybdic solution ("CAM") [phosphomolybdic acid (25 g), $Ce(SO_4)_2 \cdot H_2O$ (10 g), conc. H_2SO_4 (60 mL), H_2O (940 mL)] or potassium permanganate solution [$KMnO_4$ (1.5 g), K_2CO_3 (10 g), NaOH (aq. 10 %, 1.25 mL), H_2O (200 mL)].

Optical rotation was measured at 20 °C on a Perkin-Elmer Polarimeter 341 against the sodium D-line, solvents ($CHCl_3$, EtOH) were of spectroscopic grade purity. Circular dichroism (CD) spectra of chiral compounds were recorded on a JASCO Spectropolarimeter J-715 using methanol as solvent.

GC-MS spectra were recorded with an Agilent 7890A GC-system, equipped with an Agilent 5975C quadrupole mass selective detector operated in ESI+ mode (70 eV) and a HP-5 MS column (30 m x 0.25 mm x 0.25 µm film) using He as carrier gas (flow = 0.55 mL/min) and the following standard temperature program: 100 °C (0.5 min hold) – [10 °C/min] – 300 °C.

Samples from biotransformations were diluted with acetonitrile and analyzed on an Agilent 1260 Infinity HPLC equipped with a Phenomenex Luna reversed phase column (C18 [2], 100A, 250 m x 4.6 mm x 5 µm) and a DAD detector at 25 °C using the following method: flow = 1 mL/min; mobile phase A: water + 0.1% v/v TFA, B: acetonitrile + 0.1 %v/v TFA; 0–2 min (100% A), 2–15 min (100–60% A), 15–20 min (60–0% A), 20–22 min (0% A), 22–24 min (0%–100% A), 24–25 min (100% A). Quantification of the reaction constituents was performed at 270 nm after calibration of 4-vinylphenol **1**, hydrate **4** and nucleophile adducts **3a** – **c** within the range of 10–0.5 mM using anisole as internal standard. Enantiomeric excess (*e.e.*) of i) **5** and **3a** was measured on a Chiralcel OD-H column (25 m x 0.46 cm, Daicel) with a Shimadzu 20A HPLC System using an isocratic mixture of heptane / 2-propanol (ratio given at chromatograms) with a flow of 1 mL/min at 30°C; ii) **3c** was measured with the same system using isocratic heptane / 2-propanol 98.5:1.5; (iii) **3b** was measured after acetylation of the phenolic OH-group (acetic anhydride, DMAP cat. in EtOAc, 1h, 35 °C) on an Agilent 7890A gas chromatograph equipped with a DEX-CB column (25 m x 0.32 mm x 0.12 µm) and a FID. The following temperature program achieved enantiomer separation using H_2 (1.3 mL/min) as carrier gas: 100 °C (1 min hold) – [10 °C/min] – 160 °C (6 min hold) – [20 °C/min] – 180 °C (1min hold).

High-resolution mass spectra were measured on a Thermo Scientific Q-exactive Orbitrap MS coupled to a Thermo Scientific UltiMate 3000 HPLC with a Phenomenex Luna reversed phase column (C18 [2], 100A, 250 m x 4.6 mm x 5 µm). The same solvent gradient from above was used for separation. Either chemical- (APCI corona discharge) or electrospray ionization (ESI) operated in positive or negative mode (as indicated at spectra in section 8) was employed.

^{1}H and ^{13}C NMR spectra were recorded at 20 °C on a Bruker Avance 300 NMR unit; chemical shifts are given in ppm relative to the solvent (^{1}H: $CDCl_3$ = 7.26 ppm; ^{13}C: $CDCl_3$ = 77.0 ppm). Multiplets were termed as follows: s (singlet), bs (broad singlet), d (doublet), dd (double doublet), t (triplet), dt (double triplet), q (quartet), p (pentet), m (multiplet).

2. Sequence alignment of PADs and FDC_*Es*

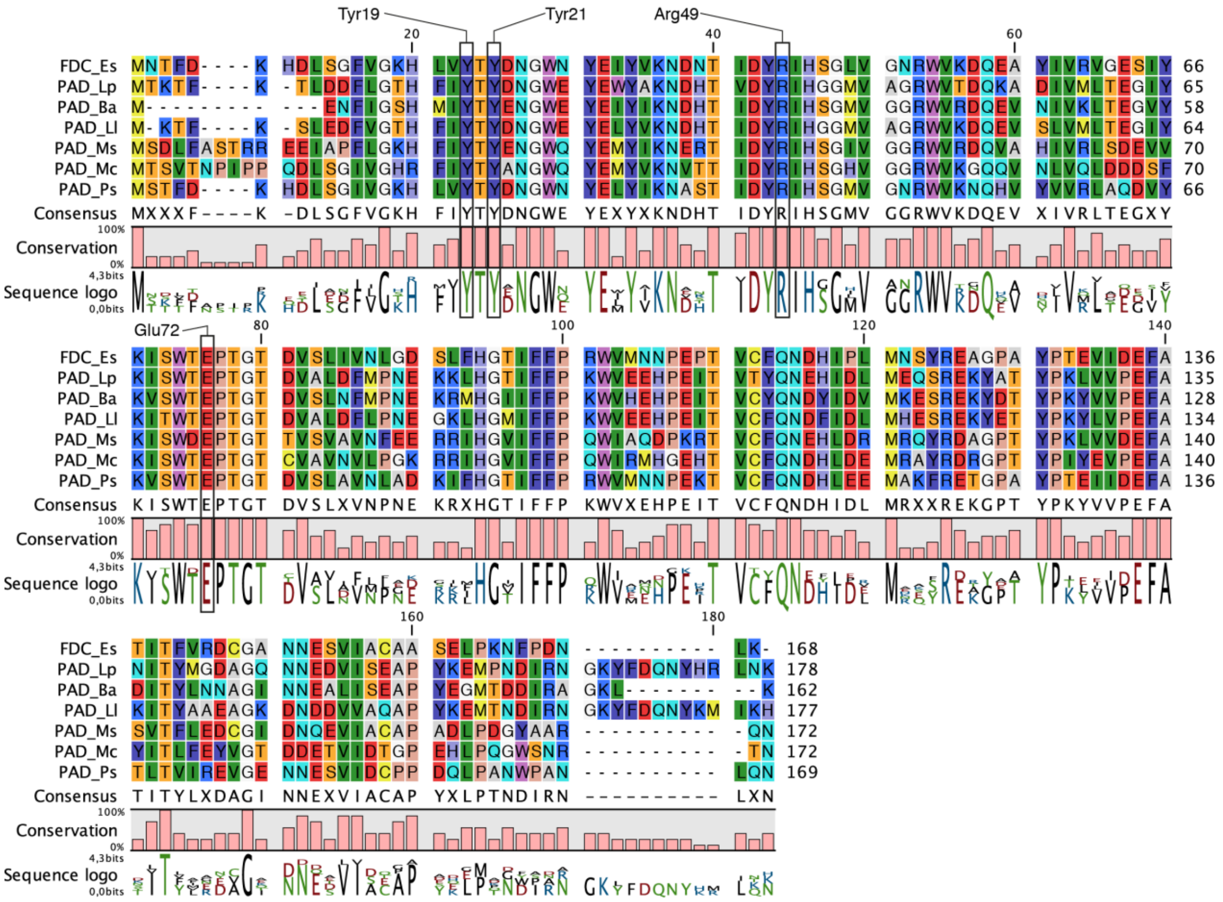

Figure S1. Sequence alignment (CLC Main Workbench 7) of phenolic acid decarboxylases (PADs) from *Lactobacillus plantarum* (*Lp*, GI: 300769086), *Bacillus amyloliquefaciens* (*Ba*, GI: 308175189), *Lactococcus lactis* (*Ll*, GI: 15673912), *Mesorhizobium* sp. (*Ms*, GI: 168197631), *Mycobacterium columbiense* (*Mc*, 342860341) and *Pantoea* sp. (*Ps*, GI: 304396594) with the ferulic acid decarboxylase (FDC) from *Enterobacter* sp (*Es*, GI: 212525355) as lead sequence (all genes allocated in NCBI Genebank). Conserved catalytic key residues responsible for decarboxylation and hydration are highlighted in boxes.

Table S1. Sequence relationships between FDC_*Es* and PADs.

Organism	Abbreviation	Sequence identity(a) (%)	Sequence difference(b) (positions)
Lactobacillus plantarum	PAD_Lp	51	88
Bacillus amyloliquefaciens	PAD_Ba	50	86
Lactococcus lactis	PAD_Ll	50	89
Mycobacterium sp.	PAD_Ms	52	83
Mycobacterium columbiense	PAD_Mc	48	90
Pantoea sp.	PAD_Ps	73	46

(a) Percentage of identical residues in alignment positions to overlapping alignment positions between FDC_*Es* and a given PAD sequence. (b) The number of alignment positions where the sequence is different (including gaps) from FDC_*Es*.

3. Cloning and heterologous expression of PADs and FDC, primer sequences for site-directed mutagenesis

Heterologous expression of genes from commercial and academic sources encoding for the respective biocatalysts was performed as described previously.[1] The biocatalysts were used as lyophilized *E. coli* whole-cell preparation and the activity was determined with HPLC *via* the initial rate of coumaric acid decarboxylation.

Table S2. Decarboxylation activities of FDC_*E*s variants and PADs.

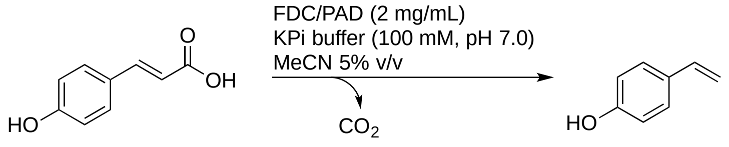

Decarboxylase	*Specific activity (U/mg lyophilisate)*
FDC_Es	*92.7*
PAD_Mc	*0.2*
PAD_Ll	*14.5*
PAD_Lp	*5.8*
PAD_Ps	*42.0*
PAD_Ba	*1.7*
FDC_Es I41A	*6.6*
FDC_Es V46E	*< 0.1*

Conditions: Lyophilized *E. coli* whole cells containing the heterologously expressed PAD or FDC (variant) (2 – 5 mg/mL) were rehydrated in 2.0 mL vials for 5 min at 30 °C and 700 rpm shaking in KP_i (100 mM, pH 7.0, 900 µL). A stock solution containing substrate coumaric acid (500 mM, 100 µL) in MeOH/MeCN 1:1 was added at t = 0 min and after short mixing incubated at 30 °C without shaking. Reactions were stopped after 15, 25 and 35 s by addition of MeCN (1 mL, containing 10 mM anisole as internal standard), the sample was centrifuged for 5 min at 14000 rpm and subjected to HPLC analytics for conversion determination. Activity was calculated from initial decarboxylation velocity.

For mutants of FDC_*E*s wt using the QuikChange Site-Directed Mutagenesis PCR Kit from Stratagene, the following primers were designed and ordered from eurofins. Site directed mutagenesis was performed according to the procedure provided by the kit supplier.

Table S3. Primer sequences for site-directed mutagenesis.

FDC_Es Variant[(a)]	*Forward primer sequence* *Reverse primer sequence*[(b)]
I41A	*5'–CTGGATTATCGCGCGCATAGCGGTCTG–3'* *5'–CAGACCGCTATGCGCGCGATAATCCAG–3'*
L80A	*5'–CACCGATGTTAGCGCGATTGTTAATCTGG–3'* *5'–CCAGATTAACAATCGCGCTAACATCGGTG–3'*
*L80A/**V78A***	*5'–ACCGACCGGCACCGATGCGAGCGCGATTGTTAATC–3'* *5'–GATTAACAATCGCGCTCGCATCGGTGCCGGTCGGT–3'*
*L80A/V78A/**W70L***	*5'–CTATAAAATCAGCCTGACCGAACCGACCG–3'* *5'–CGGTCGGTTCGGTCAGGCTGATTTTATAG–3'*
*L80A/V78A/**W70V***	*5'–CTATAAAATCAGCGTGACCGAACCGACCG–3'* *5'–CGGTCGGTTCGGTCACGCTGATTTTATAG–3'*
*L80A/V78A/W70L/**I41A***	*see primer sequences for I41A single mutant above*
*L80A/V78A/W70L/**V46A***	*5'–CATAGCGGTCTGGCGGGTAATCGTTGG–3'* *5'–CCAACGATTACCCGCCAGACCGCTATG–3'*
Y19F	*5'–GTTGGTAAACATCTGGTGTTTACCTATGATAATGGCTGG–3'* *5'–CCAGCCATTATCATAGGTAAACACCAGATGTTTACCAAC–3'*
Y21F	*5'–CATCTGGTGTATACCTTTGATAATGGCTGGGAG–3'* *5'–CTCCCAGCCATTATCAAAGGTATACACCAGATG–3'*
Y27F	*5'–ACCTATGATAATGGCTGGGAGTTTGAGATCTATGTGAAAAATGAA–3'* *5'–TTCATTTTTCACATAGATCTCAAACTCCCAGCCATTATCATAGGT–3'*
Y39F	*5'–AATGAAAACACCCTGGATTTTCGCATTCATAGCGGTCTG–3'* *5'–CAGACCGCTATGAATGCGAAAATCCAGGGTGTTTTCATT–3'*
E72A	*5'–AATCAGCTGGACCGCGCCGACCGGCACCG–3'* *5'–CGGTGCCGGTCGGCGCGGTCCAGCTGATT–3'*

[(a)] For multiple-round mutations on variants, the primer-targeted position is highlighted in bold. [(b)] Site of mutation is underlined.

4. Synthesis of racemic and non-racemic reference material

rac-4-(1-Hydroxyethyl)phenol (*rac*-**4**)

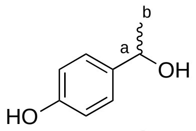

rac-**4**

In a round bottom flask $CeCl_3$·7 H_2O (7.34 mmol, 2,74 g, 1.0 eq) was dissolved in MeOH (25 mL) and 4-hydroxyacetophenone (7.34 mmol, 1.0 g) was added. Sodium borohydride ($NaBH_4$, 7.34 mmol, 277.9 mg, 1.0 eq) was added to the stirred solution at 21 °C in small portions over 5 min. Upon completion of addition, the mixture was stirred for 40 min at 21 °C after which tlc showed full conversion of the ketone [petrolether /EtOAc 1:2, UV and CAM staining, R_f = 0.3 (substrate), 0.17 (product **4**)]. The reaction was quenched by adding saturated aq. NH_4Cl solution (40 mL) and extracted with EtOAc (3 x 30 mL). After drying the combined organic layers over Na_2SO_4, removal of the solvent under reduced pressure and flash-column chromatography (petrolether/EtOAc 1:1), 722.2 mg of pure benzylic alcohol **4** (71.5% yield) was obtained as a colorless solid. ^{1}H NMR (300 MHz, MeOD): δ_H [ppm] 7.17 (m, 2H, C^{ar}–H), 6.83 – 6.66 (m, 2H, C^{ar}–H), 4.73 (m, J = 9.7 Hz, 1H, Cα–H), 1.40 (d, J = 6.5 Hz, 3H, Cβ–H_3). ^{13}C NMR (75 MHz, MeOD) δ_C [ppm] 157.6, 138.3, 127.8 (2C), 115.9 (2C), 70.6, 25.4. GC-MS (EI+, 70 eV): t_R = 7.14 min; *m/z* (%) = 138.1 (31), 123.1 (100), 120.1 (24), 95.1 (50), 91.1 (16), 77.1 (35), 65.1 (12), 51.1 (4), 43.1 (14), 39.1 (9).

(*S*)-4-(1-Hydroxyethyl)phenol [(*S*)-**4**]

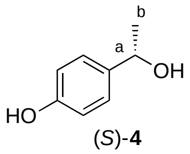

(*S*)-**4**

Lyophilized *E. coli* whole cells containing heterologously expressed ADH from *Rhodococcus ruber* were suspended in a 50 mL Falcon tube in KP_i buffer (50 mM, pH 7.0, 18 mL). To this suspension, NAD^+ sodium salt (13.3 mg, 1 mM) and 2-PrOH (2 mL, 10 % v/v) was added. 4-Hydroxyacetophenone (204.2 mg, 75 mM) was dissolved in 2-PrOH (200 µL), transferred to the enzyme suspension and the mixture was incubated at 30 °C and 120 rpm in a shaker. The reaction progress was followed by tlc [petrolether/EtOAc 2:1, UV and CAM stain, R_f=0.5 (substrate), 0.33 (product **5**)] and after 24 h, a second portion of 2-PrOH (1 mL) was added. The reaction was stopped after 36 h by extraction with EtOAc (3 x 15 mL) and the combined organic extracts were dried over Na_2SO_4 before removing the solvent under reduced pressure. Pure product alcohol (*S*)-**4** was obtained with an *e.e.* of 90% as 82.6 mg of a crystalline white solid (40 %) *via* flash column chromatography (petrolether/EtOAc 2:1). Analytical data match those of *rac*-**4**.

rac-4-[(1-Methoxyamino)ethyl]phenol (*rac*-**3a**)

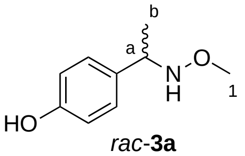

rac-**3a**

Methoxyamine hydrochloride (**2a** · HCl, 3.7 mmol, 306 mg, 5.0 eq) was dissolved in anhydrous MeOH (3 mL). In 1.5 mL vials, KOH (3.7 mmol, 206 mg, 5.0 eq) and 4-hydroxyacetophenone (735 µmol, 100 mg) were dissolved in 500 µL anhydrous MeOH each. The methanolic KOH-solution was added dropwise to the stirred solution of the hydrochloride of **2a** forming a white precipitate, followed by the addition of the dissolved ketone. After stirring for 30 min at 21 °C, full conversion of the ketone to the corresponding intermediate oxime was detected by tlc [petrolether/EtOAc 2:1, UV and CAM staining, R_f = 0.41 (substrate), 0.67 (oxime intermediate)]. $NaBH_3CN$ (441 µmol, 27.7 mg, 0.6 eq.) was dissolved in 1.5 mL anhydrous MeOH and added to the reaction mixture. The pH was subsequently adjusted to 3.0 by dropwise addition of methanolic HCl using methyl orange as pH-indicator. The reaction was stirred at 21 °C overnight after which an oxime to amine conversion of ca. 65% was detected by GC-MS. Thereafter another portion of $NaBH_3CN$ dissolved in methanol was added (27.7 mg, 0.6 eq) and after 4 h of continued stirring the reaction was quenched by adding a mixture of HCl aq. 35%/MeOH 1:1 (500 µL). The solvent was evaporated under reduced pressure to yield a yellowish solid, which was taken up in diluted HCl (10% aq., 10 mL) and washed with EtOAc (2 x 10 mL). Then the pH of the aqueous phase was adjusted with aq. NaOH (10 M) to pH 10 upon which a colorless precipitate formed. The suspension was extracted with EtOAc (3 x 10 mL), the combined organic layers were dried over Na_2SO_4 and the solvent removed under reduced pressure to yield 97.2 mg of **3a** as light brownish oil (79 %) after drying in an vacuum desiccator overnight. ^{1}H NMR (300 MHz, $CDCl_3$): δ_H [ppm] 7.23 – 7.18 (m, 2H, C^{ar}–H), 6.78 – 6.73 (m, 2H, C^{ar}–H), 4.09 (q, J = 6.6 Hz, 1H, C_α–H), 3.50 (s, 3H, C^1–H_3), 1.35 (d, J = 6.6 Hz, 3H, C_β–H_3). ^{13}C NMR (75 MHz, $CDCl_3$) δ_C [ppm] 155.41, 134.2, 128.5 (2C), 115.5 (2C), 62.4, 59.9, 19.6. GC-MS (EI+, 70 eV): t_R = 8.70 min; *m/z* (%) = 167.1 (1), 152.1 (1), 135.1 (2), 121.1 (100), 103.1 (12), 93.1 (7), 91.1 (13), 77.0 (15), 65.1 (10), 53.1 (2), 42.1 (5) 39.1 (7). HR-MS (APCI+): *m/z* = 168.10173 [MH^+] (calcd.: 168.10191).

rac-2-(4-Hydroxyphenyl)propanenitrile (*rac*-**3b**)

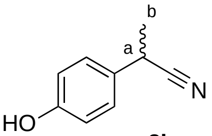

rac-**3b**

Adapted from a literature procedure.[2] All manipulations were carried out under in a well ventilated fumehood in presence of a HCN detector! In a 1.5 mL crimp-cap vial equipped with a stirring bar, *rac*-**4** (362 µmol, 50.0 mg) and NaCN (543 µmol, 26.6 mg, 1.5 eq) were dissolved in anhydrous DMF (from Roth, 400 µL) and the vial was sealed with a crimp cap and a flexible Teflon sealing. The vial was placed in an aluminum heating-block at 130 °C and after stirring for 6 h after, tlc confirmed full consumption of **4** (petrolether/EtOAc 7:3 + 1% v/v acetic acid, UV and CAM staining, R_f = 0.3 (**4**, substrate), 0.55 (**3b**, product). DMF was removed under reduced pressure (50 °C, 22 mbar) and the black-brown residue was taken up in EtOAc (1 mL). The solution was washed with water (1 mL) and after drying over $MgSO_4$ was evaporated in an air stream. The crude product was purified by flash column chromatography (petrolether/EtOAc 7:3) to yield 34.1 mg of **3b** as colorless viscous oil (64 %). ^{1}H NMR (300 MHz, $CDCl_3$): δ_H [ppm] 7.22 – 7.18 (m, 2H, C^{ar}–H), 6.86 – 6.81 (m, 2H, C^{ar}–H), 5.58 (s, 1H, O–H), 3.84 (q, *J* = 7.3 Hz, 1H, Cα–H), 1.61 (d, *J* = 7.3 Hz, 3H, Cβ–H_3). ^{13}C NMR (75 MHz, $CDCl_3$) δ_C [ppm] 155.7, 128.9, 128.1 (2C), 122.1, 116.1 (2C), 30.6, 21.5. GC-MS (EI+, 70 eV): t_R = 8.80 min; *m/z* (%) = 147.1 [M^+] (29), 132.1 (100), 120.1 (2), 104.1 (2), 91.1 (4), 77.1 (8), 65.1 (3), 51.1 (4), 39.1 (3). HR-MS (APCI+): *m/z* = 148.07555 [MH^+] (calcd.: 148.07569).

rac-4-[(1-Propylthio)ethyl]phenol (*rac*-**3c**)

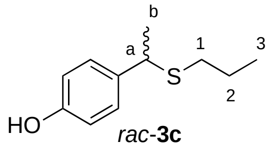

rac-**3c**

Adapted from literature procedure.[3] In a dry 4.0 mL screwtop glass vial equipped with a stirring bar and a PTFE-septum, 4-hydroxyacetophenone (734.5 µmol, 100 mg, 1.0 eq) was dissolved in anhydrous CH_2Cl_2 (2 mL). Propanethiol (955 µmol, 87 µL, 1.3 eq), triethylsilane (808 µmol, 129 µL, 1.1 eq.) and a solution of triflic acid in nitromethane (1 M solution, 7.34 µmol, 7.4 µL, 0.01 eq) were added in this sequence with a syringe through the septum. The reaction progress was monitored by tlc [petrolether/EtOAc 7:3, UV and CAM staining, R_f = 0.28 (educt), 0.68 (product)]. The bright-yellow mixture was stirred at 21 °C overnight (20 h) and then aliquoted in 2 x 1 mL portions before quenching with sat. aq. $NaHCO_3$ solution (500 µL each). Phases were separated by centrifugation (5 min, 14000 rpm), the combined CH_2Cl_2 phases were dried over $MgSO_4$ and the solvent removed *in vacuo* at ambient temperature. Flash column chromatography (pethrolether/EtOAc 9:1) yielded 78.5 mg of **3c** (54 % yield) as colorless oil after drying *in vacuo* (1 mbar, 1 h). ^{1}H NMR (300 MHz, $CDCl_3$): δ_H [ppm] 7.24 – 7.17 (m, 2H, C^{ar}–H), 6.81 – 6.74 (m, 2H, C^{ar}–H), 4.90 (bs, 1H, C^{ar}–OH), 3.91 (q, *J* = 7.0 Hz, 1H, Cα–H), 2.36 – 2.21 (m, 2H, C^2H_2), 1.60 – 1.41 (m, 5H, C^1H_2, CβH_3), 0.91 (t, *J* = 7.3 Hz, 3H, C^3H_3). ^{13}C NMR (75 MHz, $CDCl_3$): δ_C [ppm] 154.5, 136.5, 128.6 (2C), 115.4 (2C), 43.5 (d), 33.4, 22.8, 13.7. GC-MS (EI+, 70 eV): t_R = 10.75 min; *m/z* (%) = 196.1 [M^+] (7), 153.1 (1), 121.1 (100), 103.1 (7), 91.1 (15), 77.1 (10), 65.1 (5), 39.1 (5). HR-MS (APCI+): *m/z* = 197.09602 [MH^+] (calcd.: 197.09946).

rac-Ethyl 2-cyano-3-(4-hydroxyphenyl)butanoate (*rac*-**3e**)

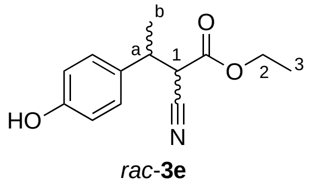

rac-**3e**

4-Hydroxyacetophenone (300 mg, 2.2 mmol), ethyl cyanoacetate (**2e**; 235 µL, 2.2 mmol, 1.0 eq), ammonium acetate (34 mg, 441 µmol, 0.2 eq) and acetic acid (98 µL, 1.76 mmol, 0.8 eq) were mixed with toluene (2 mL) in a 5 mL microwave reaction vessel equipped with a stirring bar. The mixture was iteratively irradiated in a microwave reactor

(Monowave, Anton Paar) for 4 x 5 min at 160 °C, condensed water was intermittently removed of from the vessel rim and the reaction mixture was dried over Na_2SO_4. The reaction was monitored after each irradiation cycle with GC-MS and tlc [petrolether / EtOAc 2:1, UV and $KMnO_4$ staining, R_f = 0.46 (4-hydroxyacetophenone), 0.51 (Knoevenagel adduct), 0.65 (**2e**)]. After the 4th cycle, a conversion of 57% was reached as determined by GC-MS. For workup, the mixture was diluted with toluene (10 mL), washed with water (15 mL), dried over Na_2SO_4 and evaporated under reduced pressure. Remaining ketone was removed *via* flash column chromatography using petrolether/EtOAc 2:1 to yield 131 mg of a mixture of the respective Knoevenagel adduct and **2e** (molar ratio 1:0.32 intermediate / **2e**, ^{1}H-NMR). Catalytic reduction with hydrogen (1 atm) of the crude intermediate (20 mg) over Pd on C (10 % w/w) in EtOAc (0.86 mL) at 21 °C was complete within 90 min (GC-MS). The suspension was filtered through celite and the solvent evaporated under reduced pressure to give 19.8 mg of a mixture of **3e** and **2e** as a colorless oil after drying *in vacuo* (molar ratio 1:0.12 **3e** / **2e** ^{1}H-NMR).

^{1}H NMR (300 MHz, $CDCl_3$): δ_H [ppm] 7.19 – 7.13 (m, 2H, C^{ar}–H), 6.81 – 6.75 (m, 2H, C^{ar}–H), 5.45 (bs, ca. 1H, C^{ar}–OH), 4.23 – 4.11 (m, 2H, C^2–H_2), 3.65 (dd, J_1 = 6.6 Hz, J_2 = 15.9 Hz, 1H, C^1–H), 3.49 (p, J = 6.6 Hz, 1H, C_α–H), 1.74 (dd, J_1 = 2.7 Hz, J_2 = 7.2 Hz, 3H, C_β–H_3), 1.21 (dt, J_1 = 7.2 Hz, J_2 = 15.9, 3H, C^3–H_3). ^{13}C NMR (75 MHz, $CDCl_3$): δ_C [ppm] 165.6 (d), 155.5 (d), 132.8, 131.88, 128.6 (d), 115.7 (d), 62.9 (d), 46.4 (d), 39.8 (d), 19.7, 17.7, 14.0 (d). GC-MS (EI+, 70 eV): t_R = 13.32 min; *m/z* (%) = 233.1 [M^+] (6), 160.1 (1), 145.1 (2), 132.1 (1), 121.1 (100), 103.1 (5), 91.1 (7), 77.1 (7), 65.1 (3), 51.1 (1), 39.1 (2). HR-MS (ESI–): *m/z* = 232.0976 (isomer 1); 232.0977 (isomer 2) [M^-] (calcd.: 232.0979).

5. Optimization of the biocatalytic process

Table S4. Enzyme screening for the addition of **2c** onto **1**.

Entry	Enzyme	**3c** (*e.e.*) [%]	**4** (*e.e.*) [%]
1	FDC_*E*s	73 (41)	13 (n.d.)
2	PAD_*Lp*	9 (23)	< 1 (n.d.)
3	PAD_*Ba*	13 (23)	< 1 (n.d.)
4	PAD_*Ll*	29 (25)	< 1 (n.d.)
5	PAD_*M*s	16 (17)	1 (n.d.)
6	PAD_*Mc*	8 (n.d.)	1 (n.d.)
7	PAD_*P*s	52 (16)	1 (n.d.)

Screening conditions: lyophilized *E. coli* cells (20 mg/mL) containing the heterologously expressed FDC or PAD, **1** (10 mM), **2c** (100 mM) in KP_i buffer (50 mM, pH 7.0) and DME (10% v/v); incubation for 24 h at 30 °C and 700 rpm; n.d. = not determined due to low conversion.

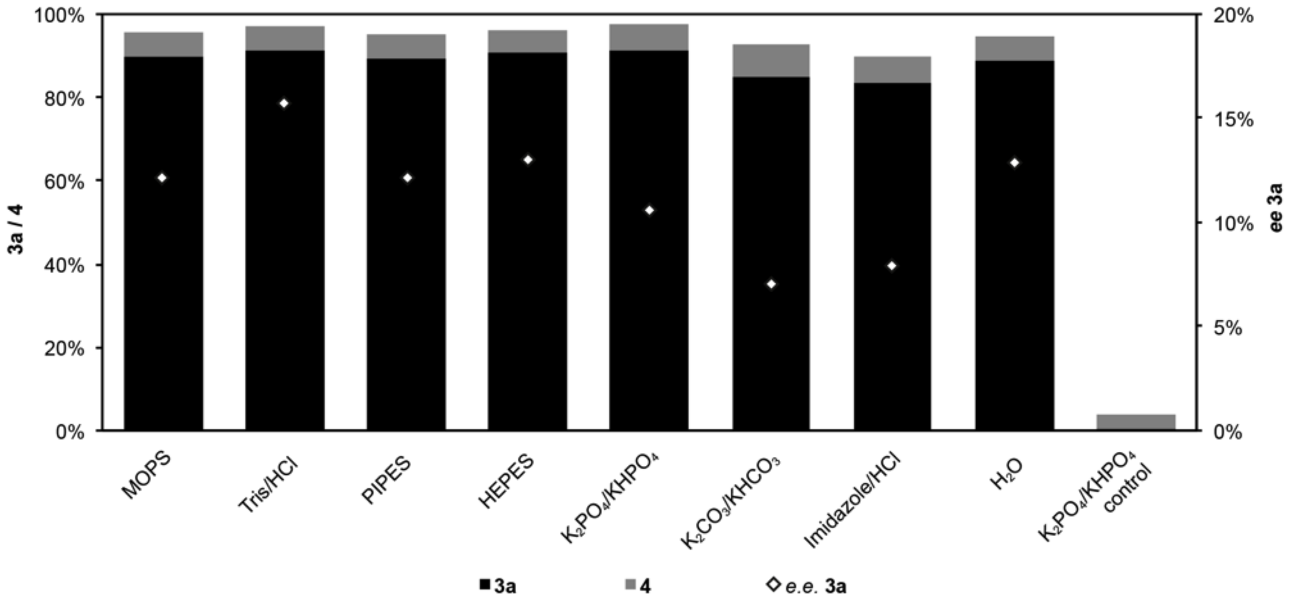

Figure S2. Influence of the buffer system on the addition of methoxyamine (**2a**) onto 4-vinylphenol (**1**). Reaction conditions: lyophilized *E. coli* cells containing the overexpressed FDC_*E*s (20 mg/mL) rehydrated in respective buffer (900 µL, 100 mM); **1** (10 mM supplied as 13.44 µL of a 8.6 % w/w stock solution in propylene glycol); methoxyamine (**2a**; 100 mM supplied as 100 µL of a 1 M stock solution in water) were incubated at 30 °C, 700 rpm for 24 h. For FDC_*E*s control, an *E. coli* preparation lacking the FDC gene was used. Conversion and *e.e.* was analyzed with HPLC after quenching with MeCN and further workup (see analytics section).

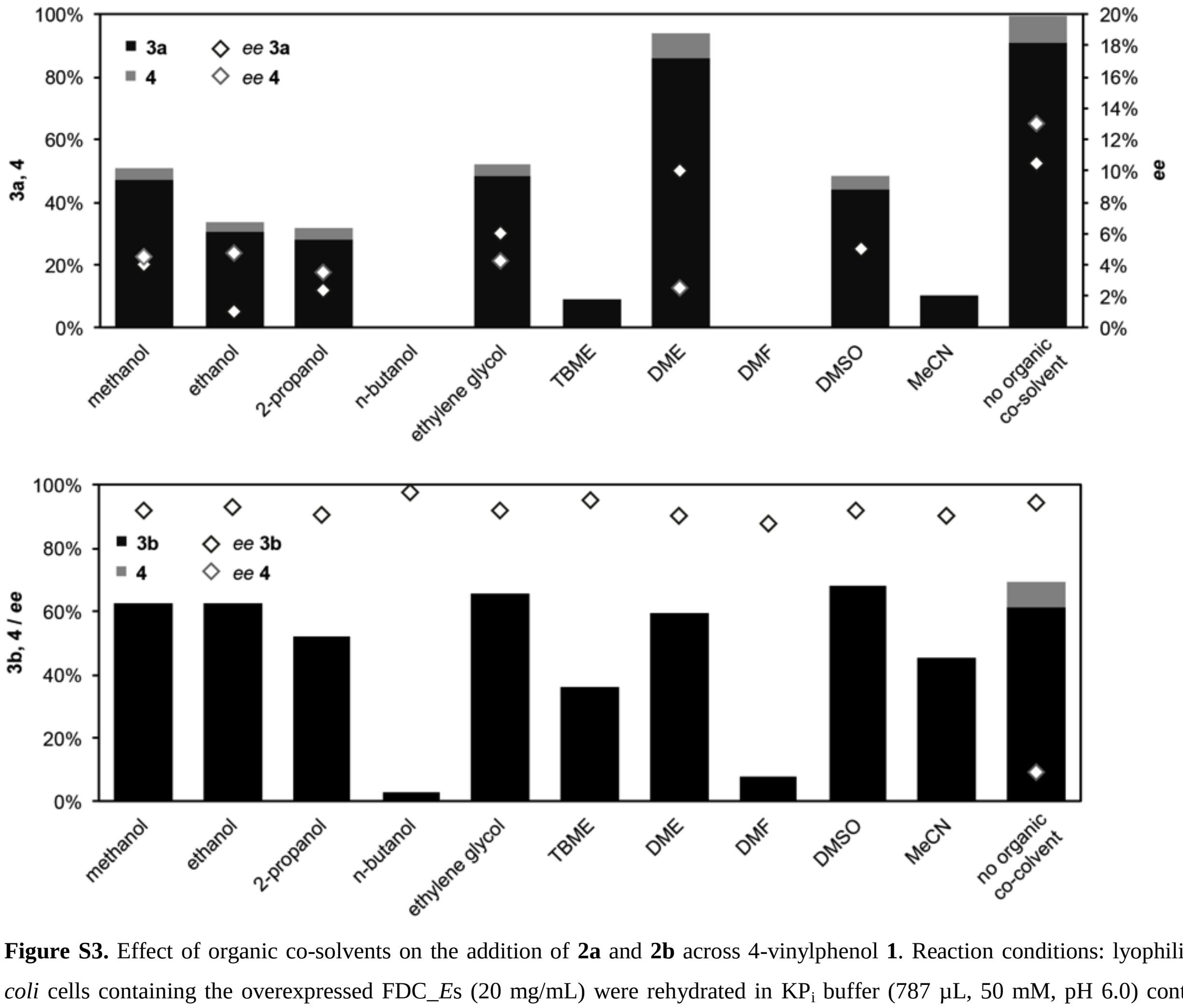

Figure S3. Effect of organic co-solvents on the addition of **2a** and **2b** across 4-vinylphenol **1**. Reaction conditions: lyophilized *E. coli* cells containing the overexpressed FDC_*E*s (20 mg/mL) were rehydrated in KP_i buffer (787 µL, 50 mM, pH 6.0) containing methoxyamine or KCN (**2a** or **2b**, resp.; 100 mM supplied as 100 µL of a 1 M stock solution in buffer) and organic co-solvent (100

µL, 10% v/v; buffer was used for control run) before addition of **1** (10 mM supplied as 13.44 µL of a 8.6 % w/w stock solution in propylene glycol) and incubation at 30 °C, 700 rpm for 24 h. Conversion and *e.e.* was analyzed with HPLC after quenching with MeCN and further workup (see analytics section).

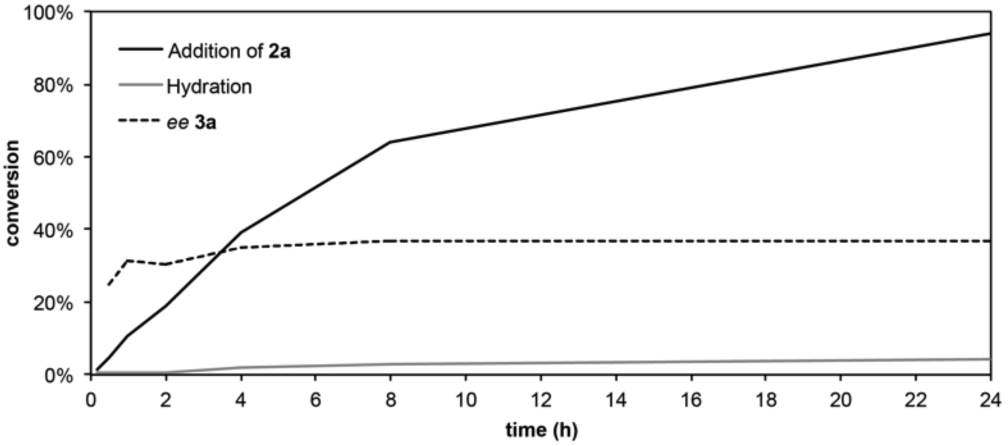

Figure S4. Time course of the addition of methoxyamine (**2a**) across 4-vinylphenol (**1**). Reaction conditions: lyophilized *E. coli* cells containing the overexpressed FDC_*E*s I41A variant (20 mg/mL) rehydrated in Na/KPO$_4$-buffer (940 µL, 100 mM); **1** (10 mM supplied as 13.44 µL of a 8.6 % w/w stock solution in propylene glycol); methoxyamine (**2a**; 100 mM supplied as 50 µL of a 2 M stock solution in water) were incubated at 30 °C, 700 rpm for the indicated time intervals. Conversion and *e.e.* was analyzed with HPLC after quenching with MeCN and further workup (see analytics section).

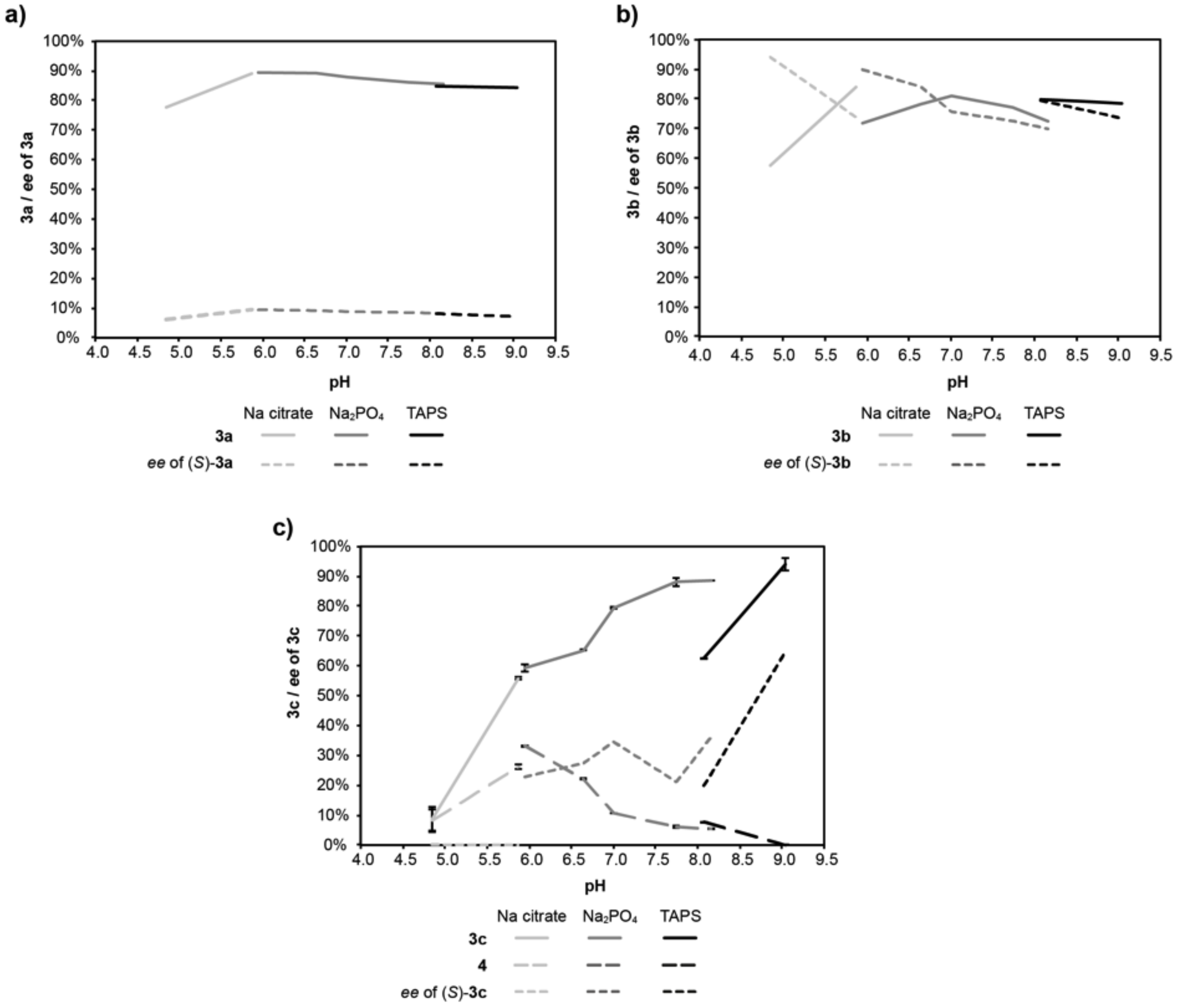

Figure S5. pH profile of 4-vinylphenol **1** in the addition reaction with a) methoxyamine **2a,** b) cyanide **2b** and c) propanethiol **2c** with varying pH-value of the reaction mixture. For pH-range 5–6 sodium citrate, for pH 6–8 NaP_i and for pH 8–9 TAPS/NaOH buffer was used. Reaction conditions: lyophilized *E. coli* cells containing the overexpressed FDC_*E*s (20 mg/mL) rehydrated in buffer (900 µL, 100 mM); **1** (10 mM supplied as 13.44 µL of a 8.6 % w/w stock solution in propylene glycol); NaCN and methoxyamine (**2a** or **2b**, resp.; 100 mM supplied as 100 µL of a 1 M stock solution in water) or propanethiol (**2c**; 100 mM supplied as 100 µL of a 1 M stock in DME) were incubated at 30 °C, 700 rpm for 24 h. Conversion and *e.e.* was analyzed with HPLC and GC-FID after quenching with MeCN and further workup (see analytics section).

a)

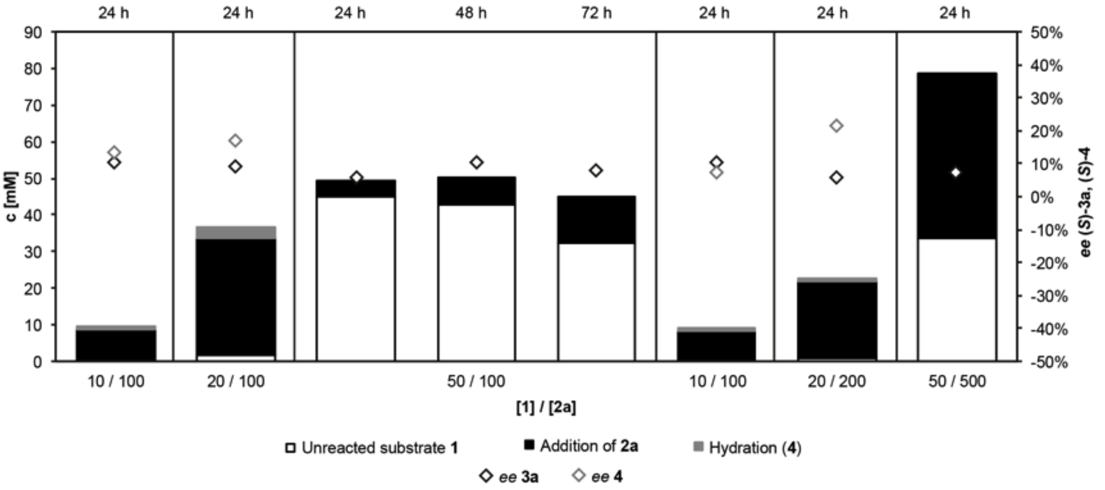

b)

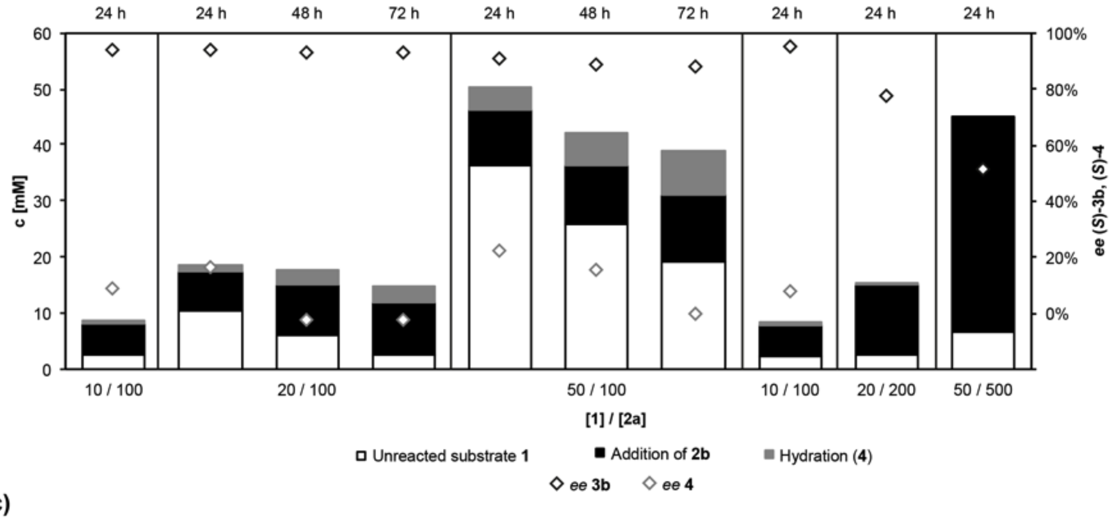

c)

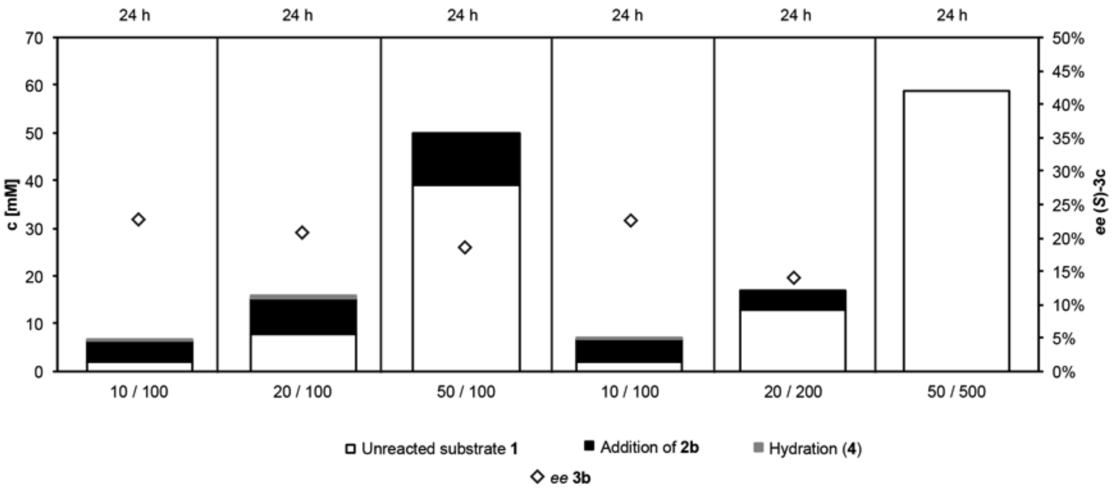

Figure S6. Optimization of substrate **1** / nucleophile **2a**–**c** loading (a–c); for samples showing incomplete conversion after 24 h, incubation time was prolonged as indicated. Reaction conditions: lyophilized *E. coli* cells containing overexpressed FDC_*E*s (20 mg/mL) rehydrated in buffer (pH 6.0, 900 µL, 100 mM); **1** (10, 20 or 50 mM supplied as aliquot of a 8.6 % w/w stock solution in propylene glycol); NaCN and methoxyamine (**2a** or **2b**, resp.; 100, 200 or 500 mM supplied as aliquot of a 1 M stock solution in buffer) or propanethiol (**2c**; 100, 200 r 500 mM supplied as 100 µL of a 1, 2 or 5 M stock in DME; 10% v/v DME) were incubated at 30 °C, 700 rpm for 24 – 72 h. Conversion and *e.e.* was analyzed with HPLC and GC-FID after quenching with MeCN and further workup (see analytics section).

6. Determination of the absolute configuration of products

Table S5. Determination of absolute configuration of products.

Entry	Compound	Derivative	Absolute configuration determined *via*	Ref.
1	(*S*)-**4**	–	optical rotation, comparison to literature	[4]
2	(*S*)-**3a**	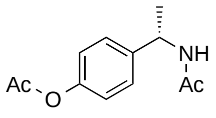 (*S*)-**6a**	comparison of GC-FID elution order with enantiomerically pure reference material of known absolute configuration[(a)]	–
3	(*S*)-**3b**	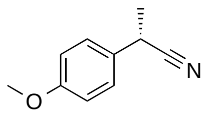 (*S*)-**5b**	optical rotation, comparison to literature	[5]
4	(*S*)-**3c**	–	Comparison of CD spectrum with compound (*S*)-**3b** of known absolute configuration.	–

[(a)] Both enantiomers of amine **5a** were obtained from BASF and were acetylated in the same fashion as the biotransformation product. [(b)] Compound **3c** shows the same (negative) Cotton-effect as sample (*S*)-**3b**, indicating same stereoconfiguration.

Ad entry 2:

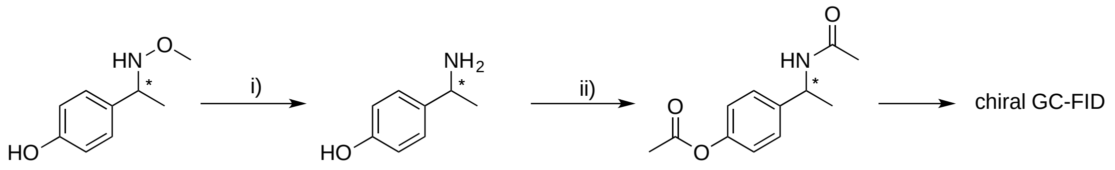

3a **5a** **6a**

Scheme S1. Derivatization of **3a** by hydrogenation and per-acetylation. i) **3a** (10 mg), acetic acid (10 µL) and Pd on C (1.1 mg 10 % w/w) in MeOH (500 µL) subjected to 1 atm H_2-pressure (balloon) at 21 °C for 1.5 (tlc [petrolether / EtOAc 7:3] showed full conversion). Work-up by filtering through celite and evaporation yielded **5a** (9.8 mg). ii) **5a** (1.7 mg, 10 mM) was acetylated with acetic anhydride (10 µL) and DMAP (0.2 mg) in EtOAc (1 mL) at 50 °C and 700 rpm for 1 h (full conv. of **5a** on tlc [$CHCl_3$ / MeOH 9:1 + 1% v/v triethylamine]). Quenching with sat. aq. NH_4Cl solution (500 µL) for 30 min at 50 °C, 700 rpm, dry EtOAc phase over $MgSO_4$ and subject to GC-FID.

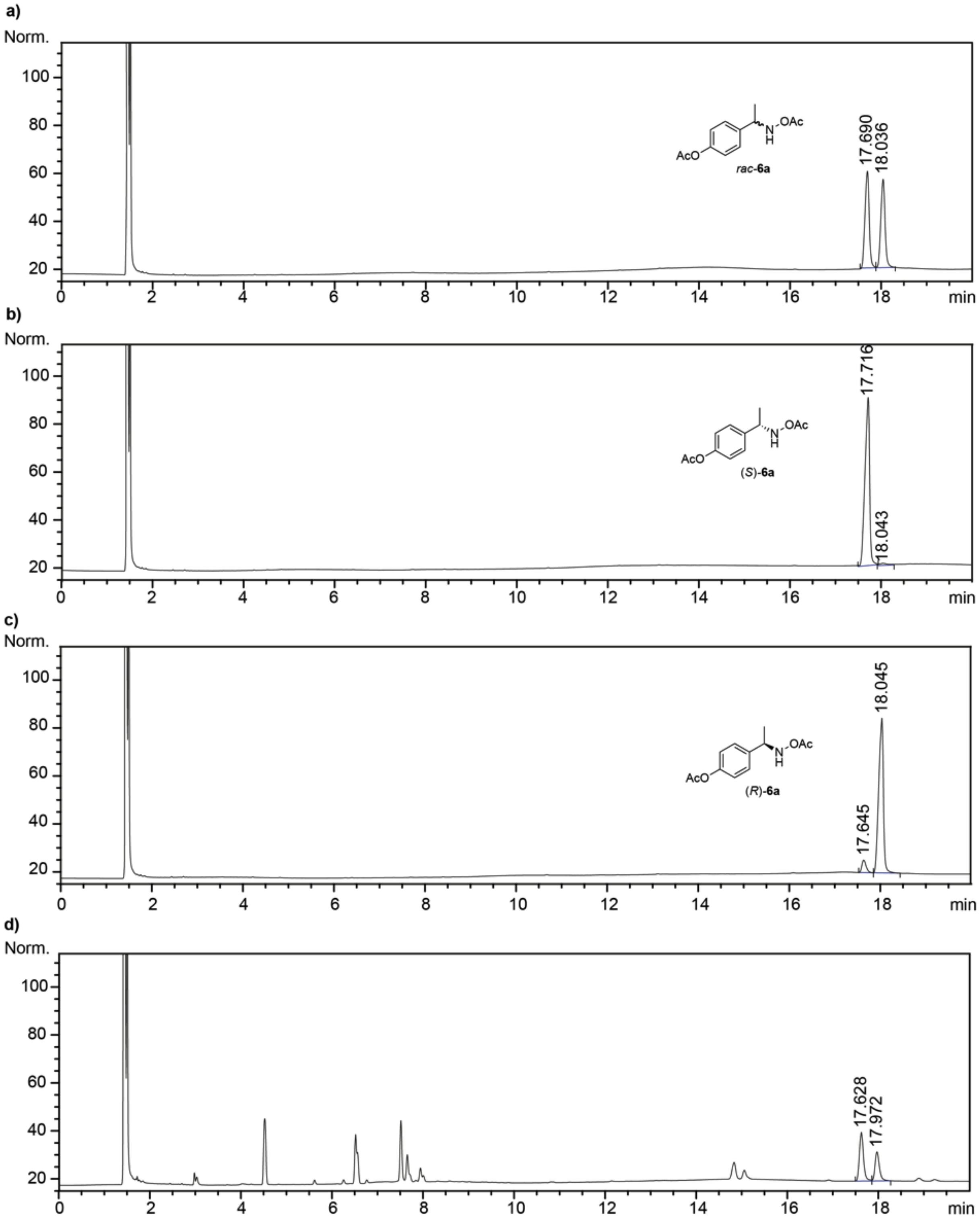

Figure S7. Determination of the absolute configuration of **3a** by comparison of the elution order from a chiral Agilent DEX-CB GC (250 m x 320 µm x 0.25 µm film) column. a) Racemic reference after peracetylation (*rac*-**6a**). b) Reference sample with (*S*)-configuration after peracetylation [(*S*)-**6a**]. c) Reference sample with (*R*)-configuration after per-acetylation [(*R*)-**6a**]. d) Sample from biotransformation (**3a**, *e.e.* = 23%) after catalytic reduction and peracetylation (**6a**). The (*S*)-enantiomer elutes first and is in excess in the biotransformation sample. Temperature program: 150 °C (3 min)–[3 °C/min]–180 °C (7 min), 1.3 mL/min H_2.

Ad entry 3:

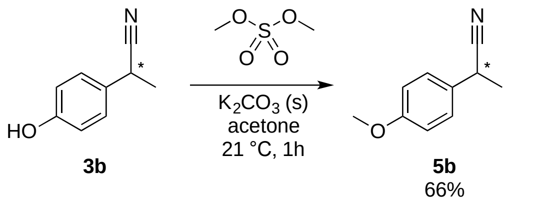

Scheme S2. Derivatization of **3b** by *O*-methylation. **3b** (20 mg, 136 µmol), dimethylsulfate (64.3 µL, 680 µmol, 5.0 eq) and powdered K_2CO_3 (94 mg, 680 µmol, 5.0 eq) were stirred in acetone (2.7 mL) for 1 h at 21 °C upon which tlc showed full conversion of **3b** (petrolether / EtOAc 9:1). Acetone was evaporated; the product was taken up in EtOAc and washed with sat. aq. Na_2CO_3 solution (1 mL). After drying over $MgSO_4$, evaporation of the solvent and passing through a small silica column (petrolether / EtOAc 9:1) yielded 14.5 mg of the pure product **5b** as colorless oil with a coconut-like smell (66% yield). Optical rotation: $[\alpha]_D^{20}$: –13.2±0.4 (c 1, $CHCl_3$, *e.e.* = 85%); $[\alpha]_D^{20}{}_{lit}$: –16 (c 0.85, CH_2Cl_2, *e.e.* = 99% [*S*]).[5]

Ad entry 4:

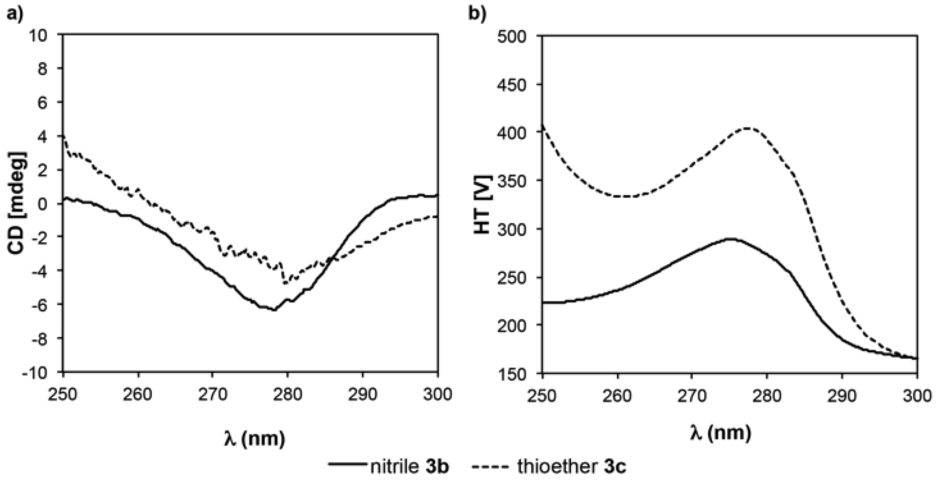

Figure S8. a) CD spectra of enantiomer-enriched **3c** (0.17 mg/mL, 5% *e.e.*) and (*S*)-configured nitrile **3b** (0.17 mg/mL, 85 % *e.e.*); both samples show negative Cotton effects suggesting (*S*)-configuration of **3c**. b) The Cotton-effect was measured at an UV-absorption maximum between 270 and 280 nm.

7. Analytical methods, example chromatograms and spectra

GC-MS chromatograms (nucleophile screening)

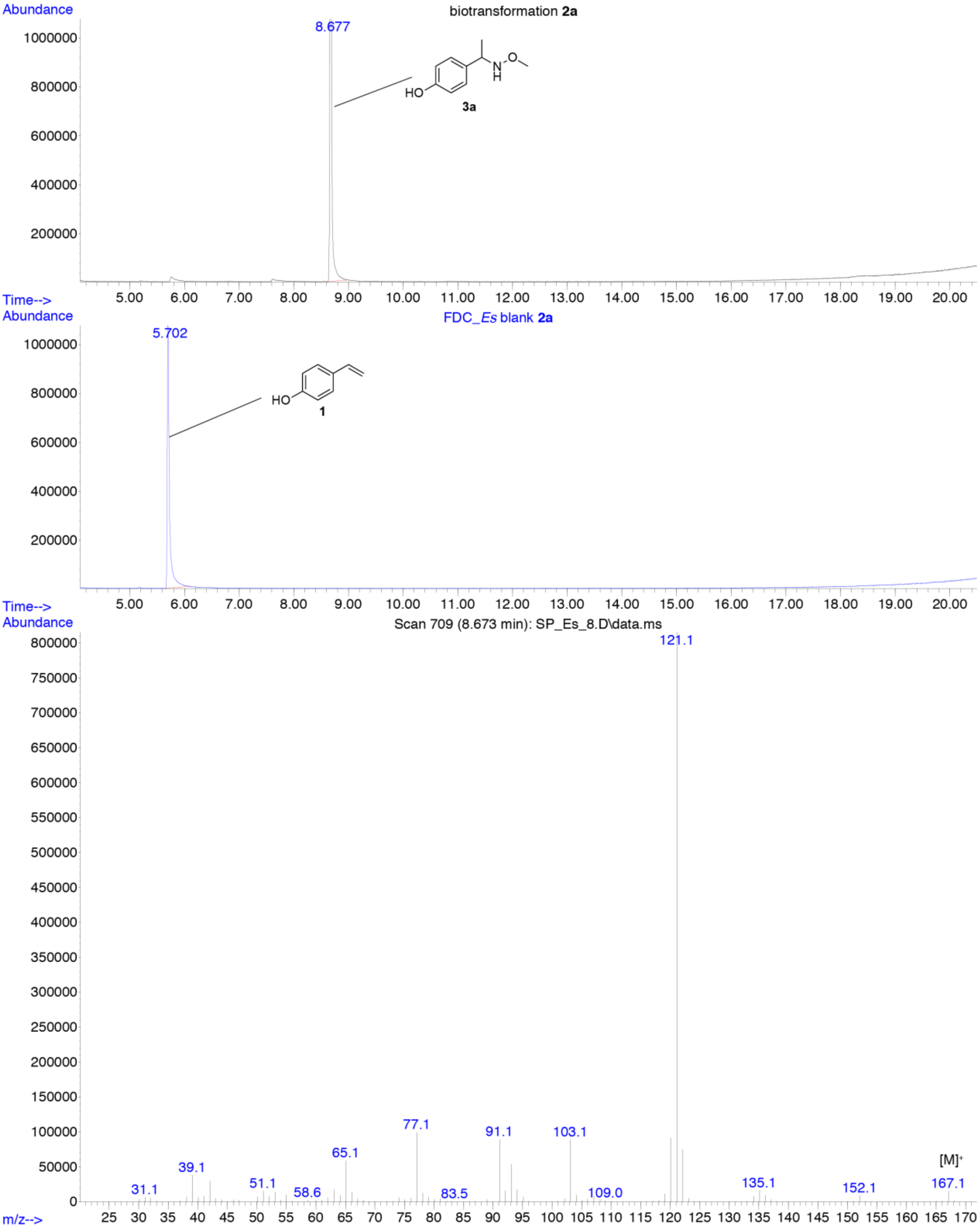

Figure S9. Biotransformation of **1** with methoxyamine (**2a**) and FDC_*E*s as biocatalyst. Upper two panels: gas-chromatograms of the biotransformation and control run without biocatalyst. Lower panel: mass spectrum of putative adduct at 8.67 min.

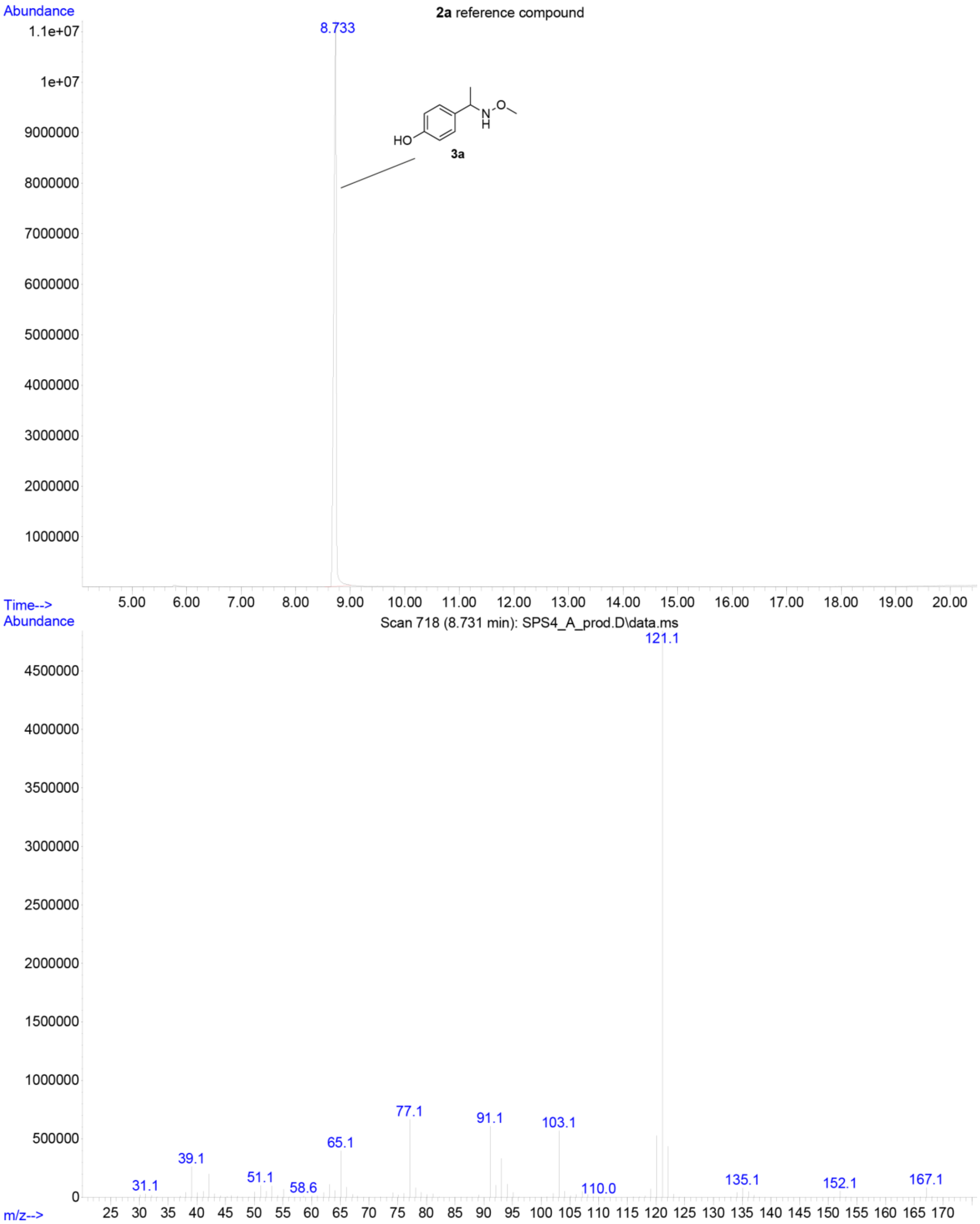

Figure S10. GC-trace and mass spectrum of reference compound **3a**.

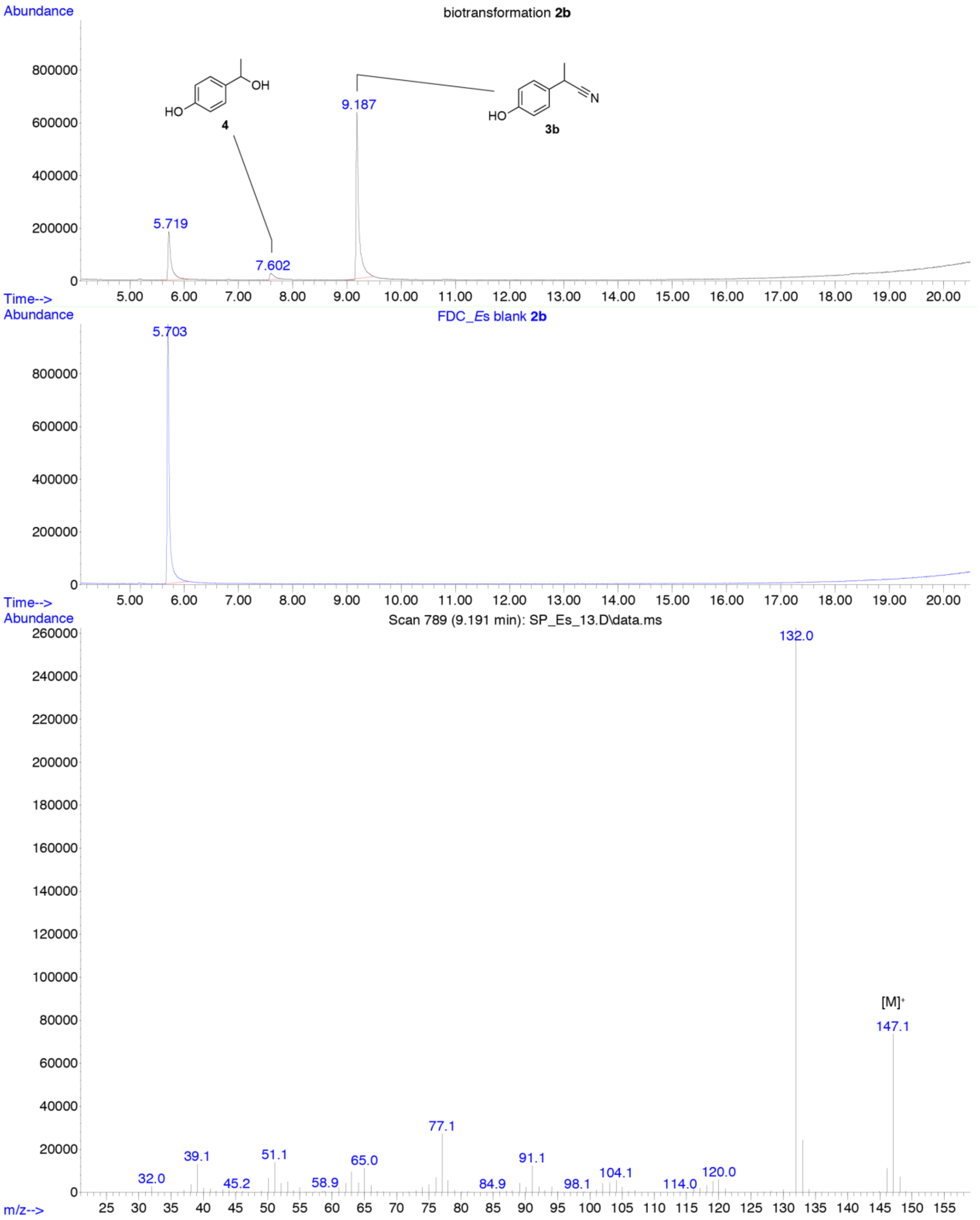

Figure S11. Biotransformation of **1** with cyanide (**2b**) and FDC_*E*s as biocatalyst. Upper two panels: gas-chromatograms of the biotransformation and control run without biocatalyst. Lower panel: mass spectrum of putative adduct at 9.19 min.

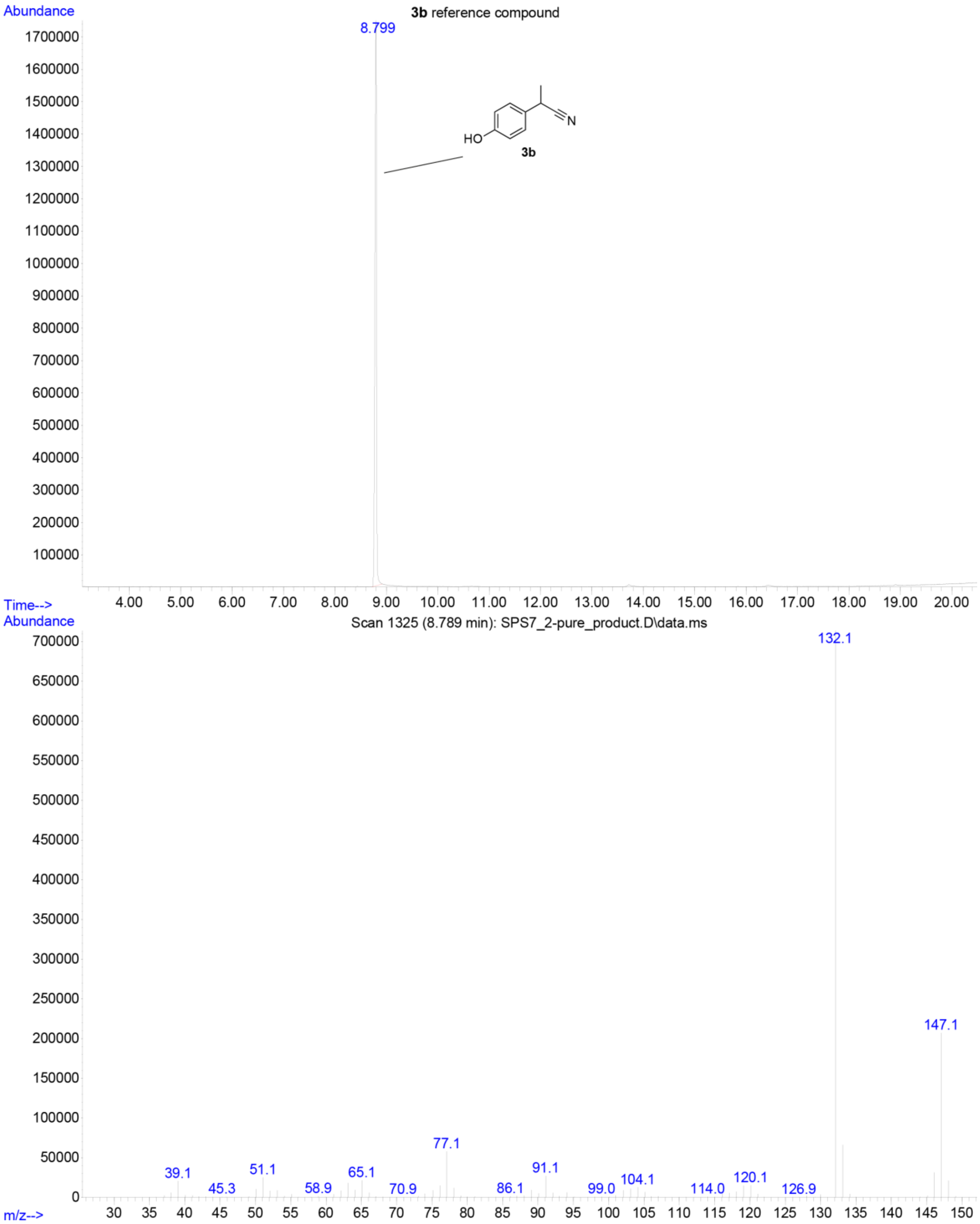

Figure S12. GC-trace and mass spectrum of reference compound **3b**.

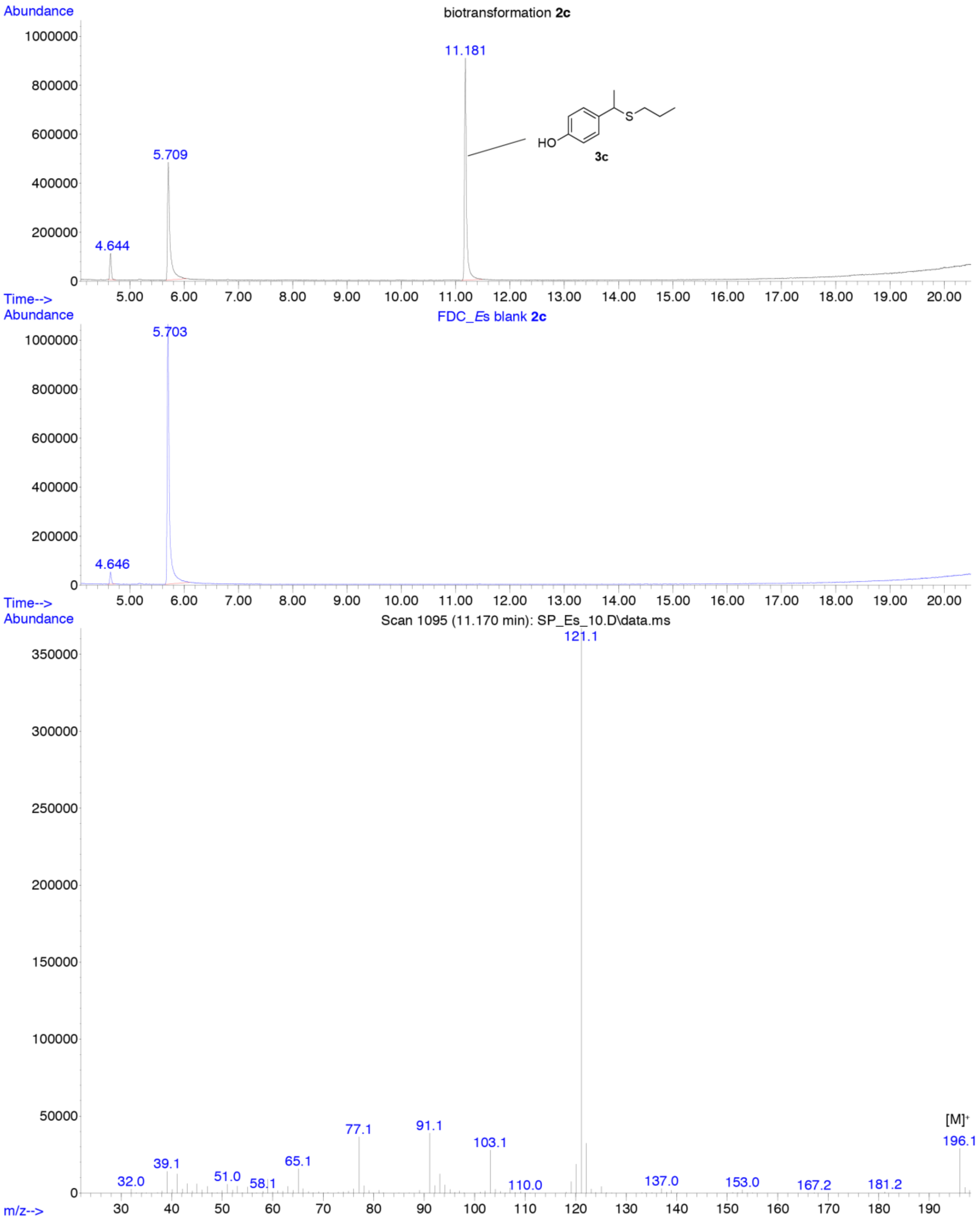

Figure S13. Biotransformation of **1** with propanethiol (**2c**) and FDC_*E*s as biocatalyst. Upper two panels: gas-chromatograms of the biotransformation and control run without biocatalyst. Lower panel: mass spectrum of putative adduct at 11.17 min.

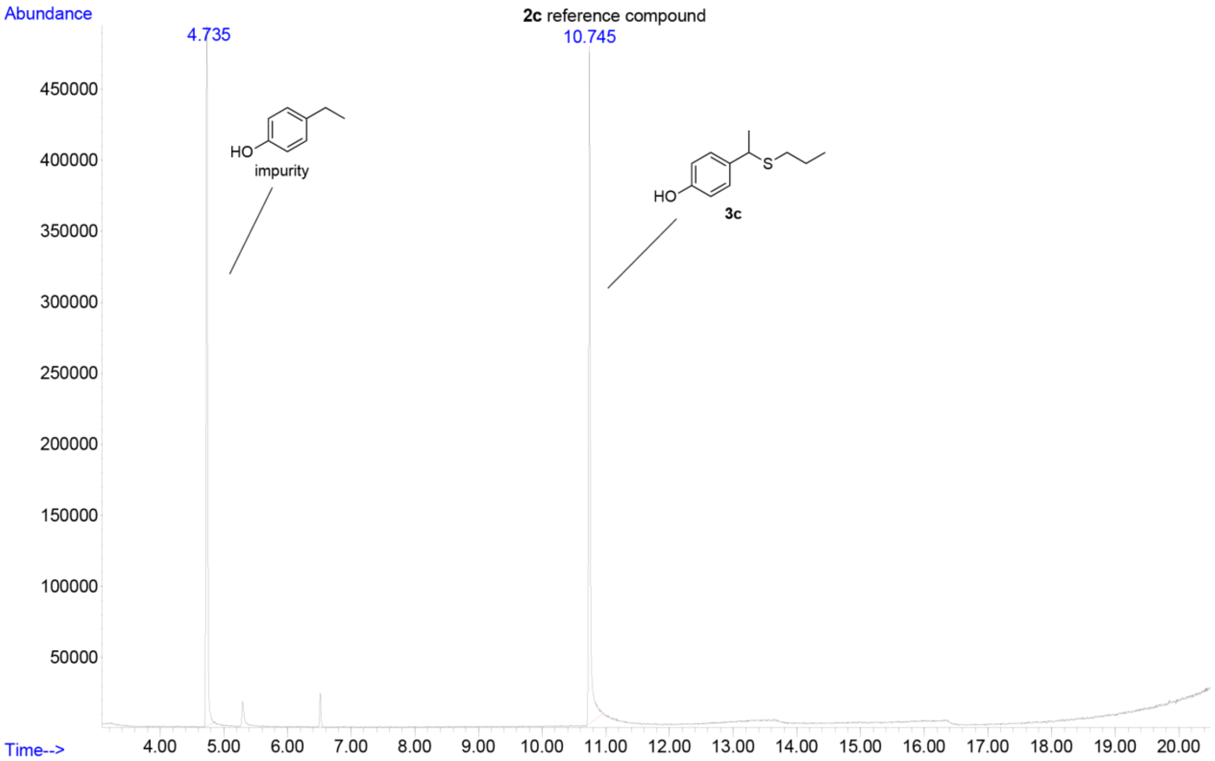

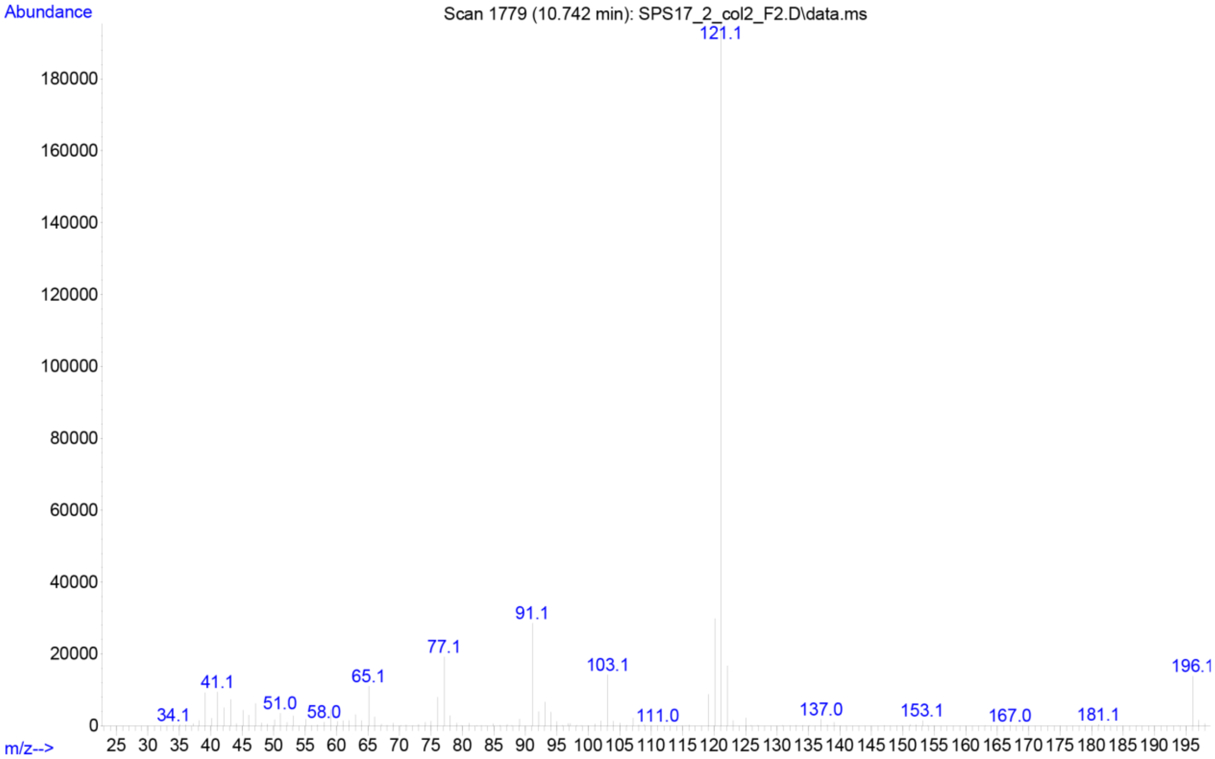

Figure S14. GC-trace and mass spectrum of reference compound **3c**. Retention-time shift due to column exchange.

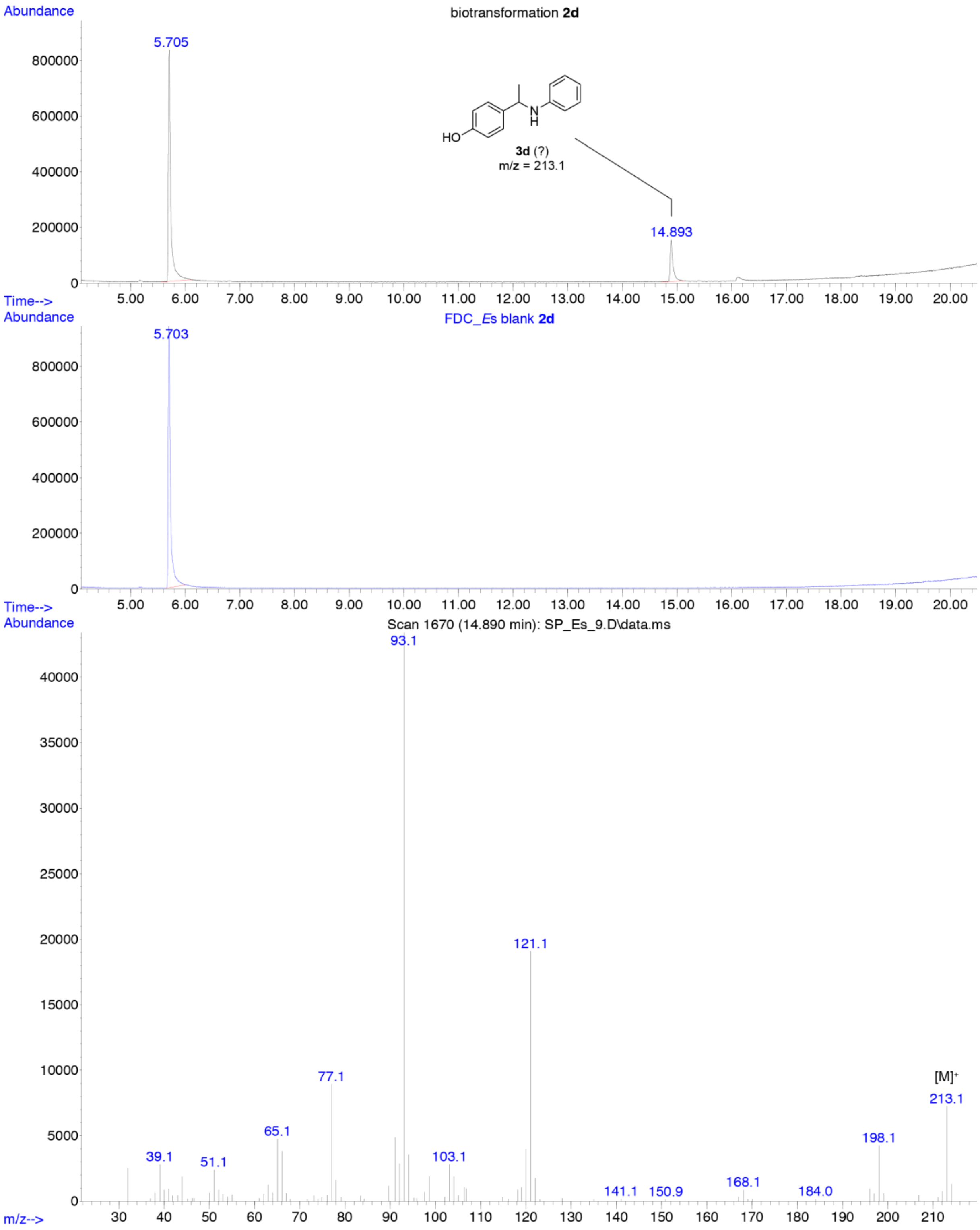

Figure S15. Biotransformation of **1** with aniline (**2d**) and FDC_*E*s as biocatalyst. Upper two panels: gas-chromatograms of the biotransformation and control run without biocatalyst. Lower panel: mass spectrum of putative adduct at 14.89 min.

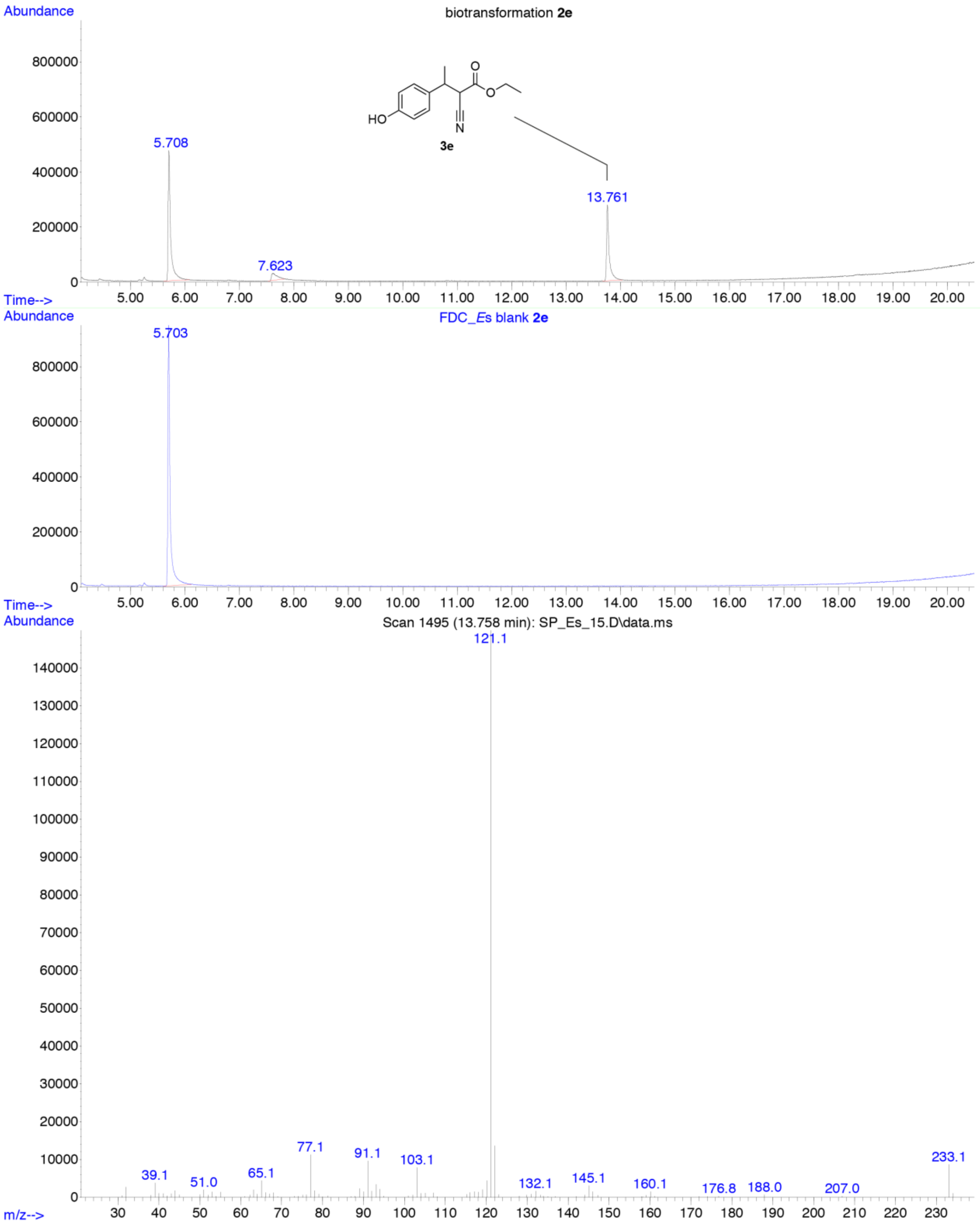

Figure S16. Biotransformation of **1** with ethyl cyanoacetate (**2e**) and FDC_*E*s as biocatalyst. Upper two panels: gas-chromatograms of the biotransformation and control run without biocatalyst. Lower panel: mass spectrum of putative adduct at 13.76 min.

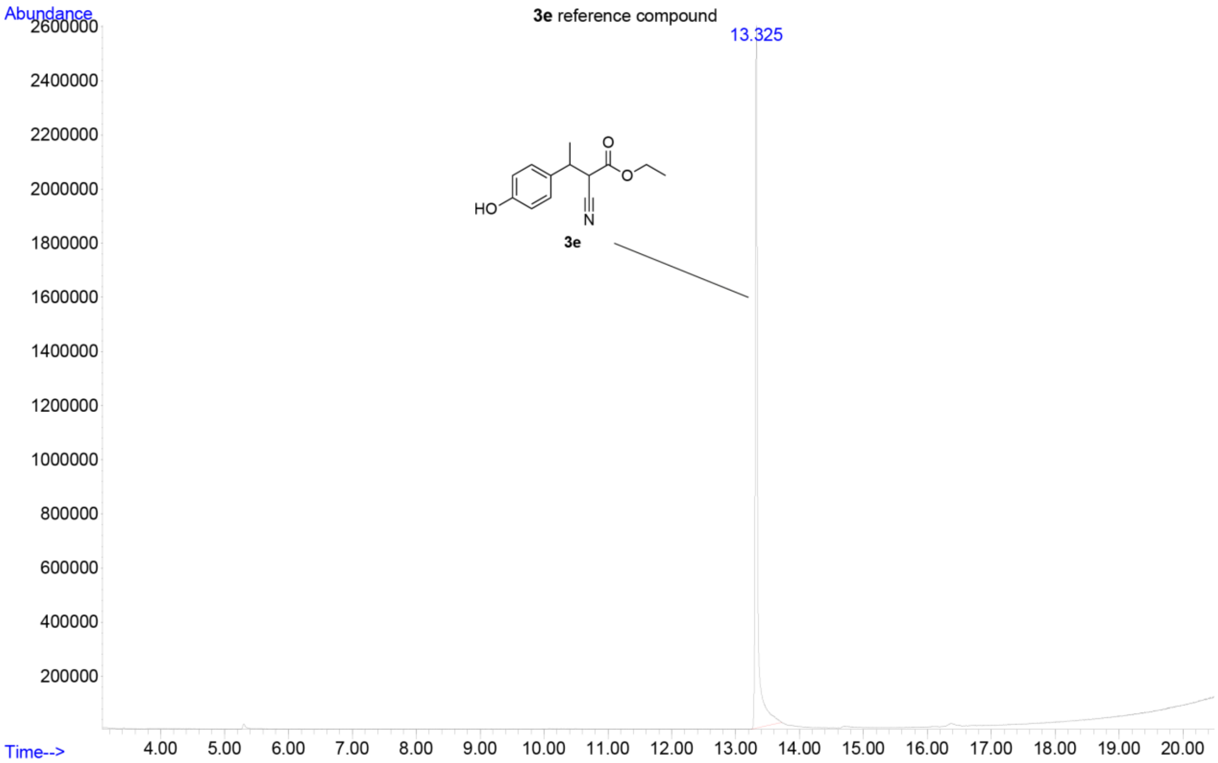

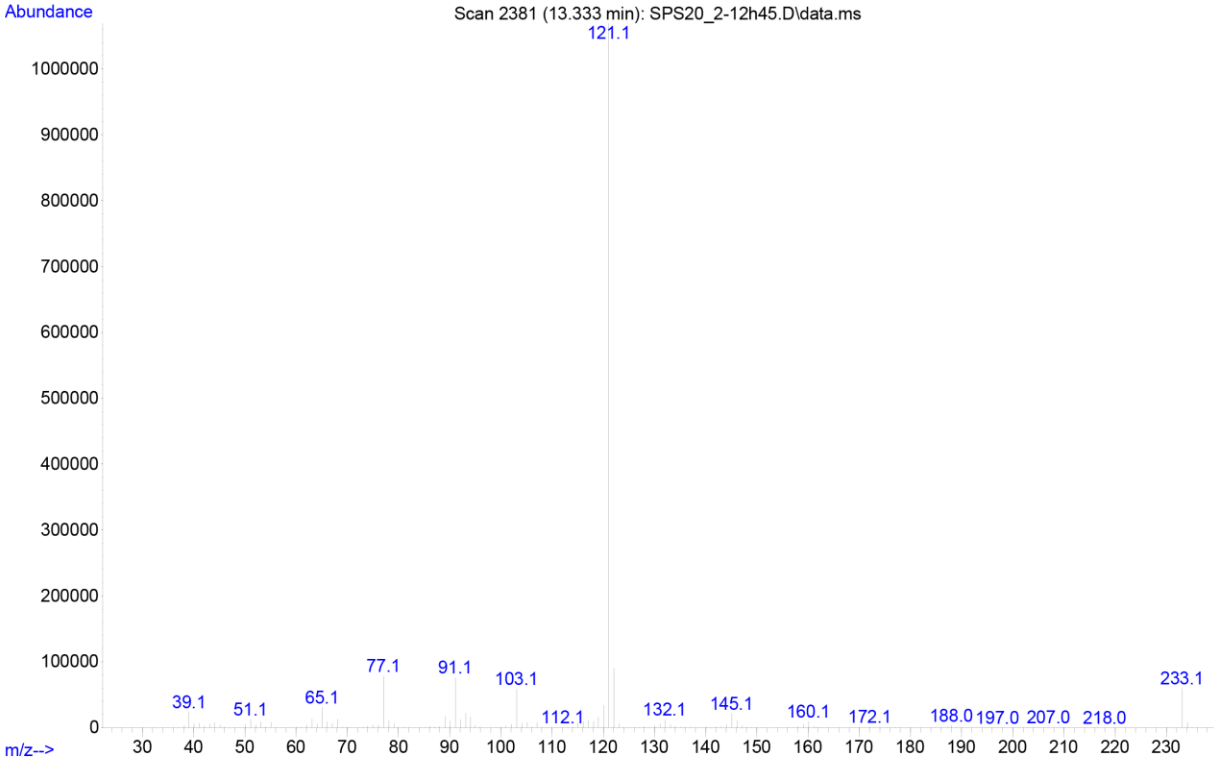

Figure S17. GC-trace and mass spectrum of reference compound **3e**. Retention-time shift due to column exchange.

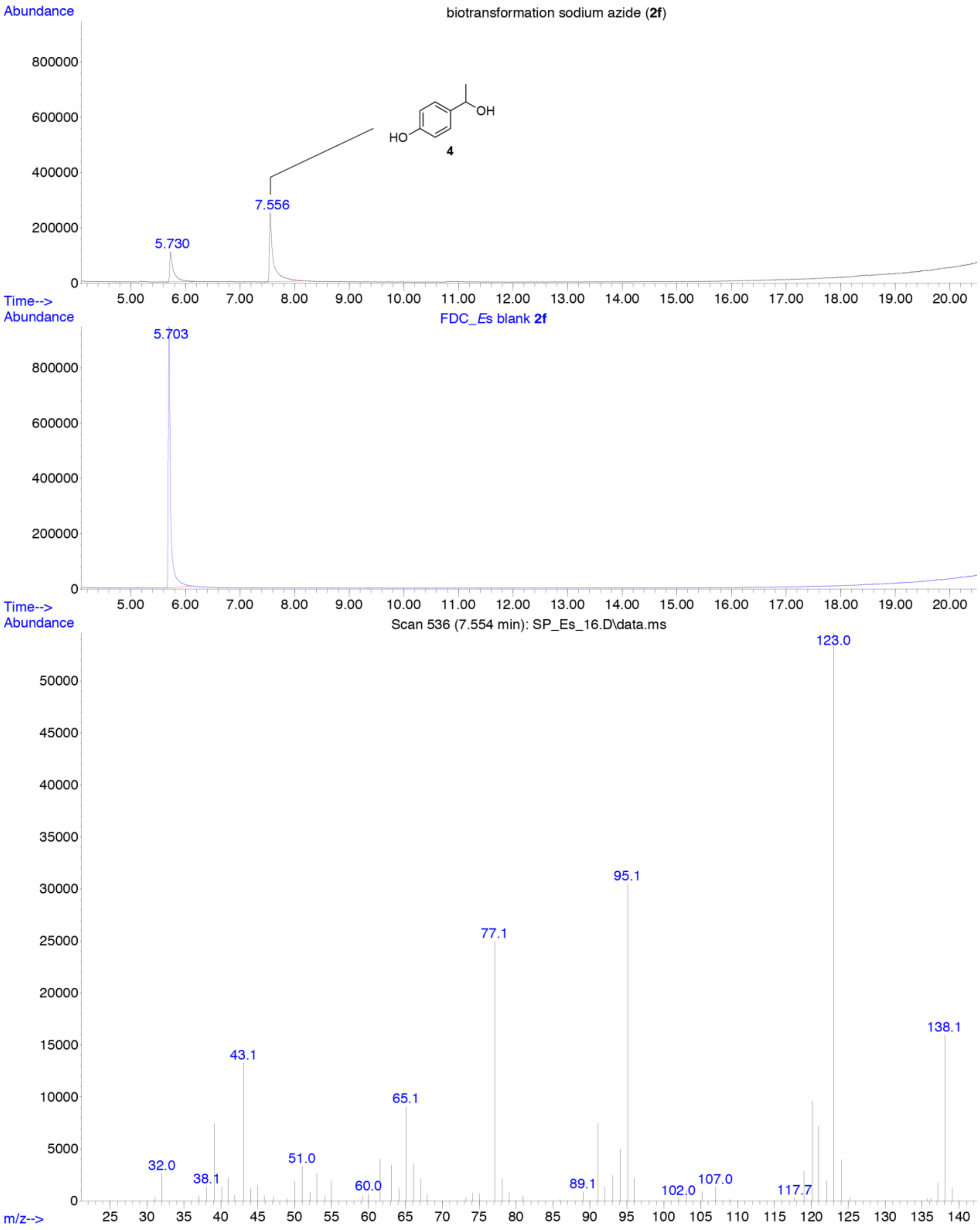

Figure S18. Biotransformation of **1** with sodium azide (**2f**) and FDC_*E*s as biocatalyst. Upper two panels: gas-chromatograms of the biotransformation and control run without biocatalyst. Lower panel: mass spectrum of hydrate **4** at 7.55 min.

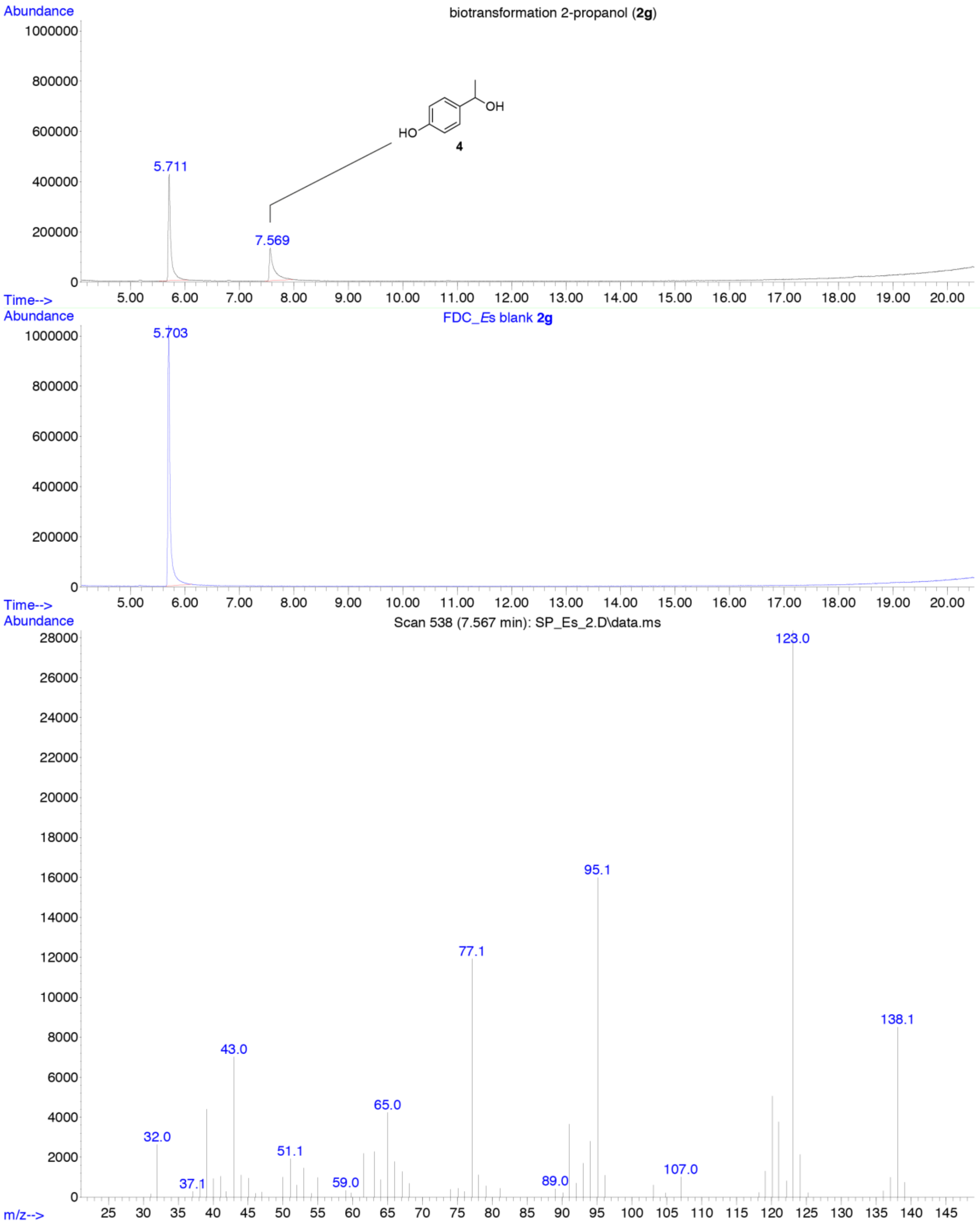

Figure S19. Biotransformation of **1** with 2-propanol (**2g**) and FDC_*E*s as biocatalyst. Upper two panels: gas-chromatograms of the biotransformation and control run without biocatalyst. Lower panel: mass spectrum of hydrate **4** at 7.55 min.

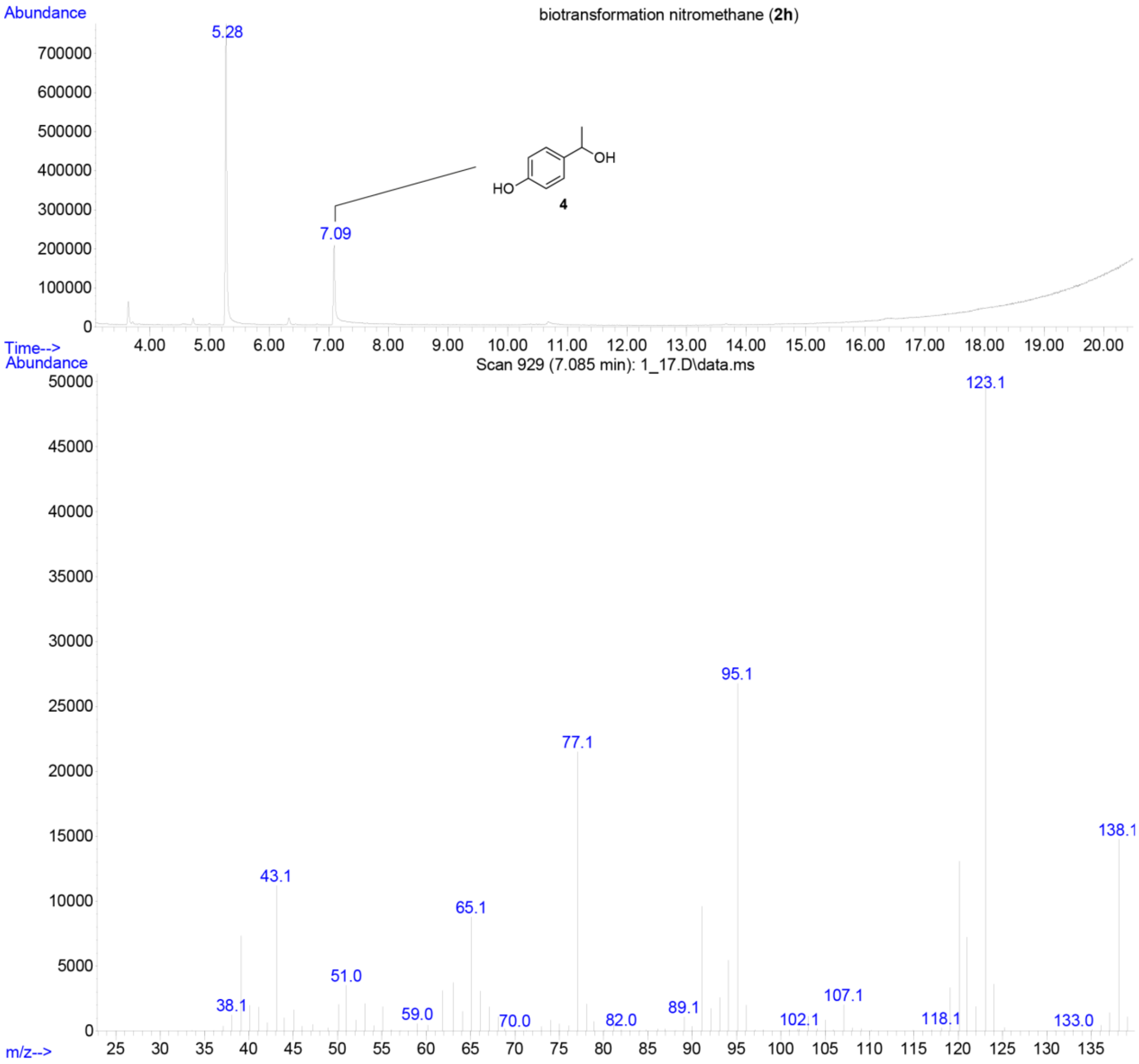

Figure S20. Biotransformation of **1** with nitromethane (**2h**) and FDC_*E*s as biocatalyst. Upper two panels: gas-chromatograms of the biotransformation and control run without biocatalyst. Lower panel: mass spectrum of hydrate **4** at 7.55 min.

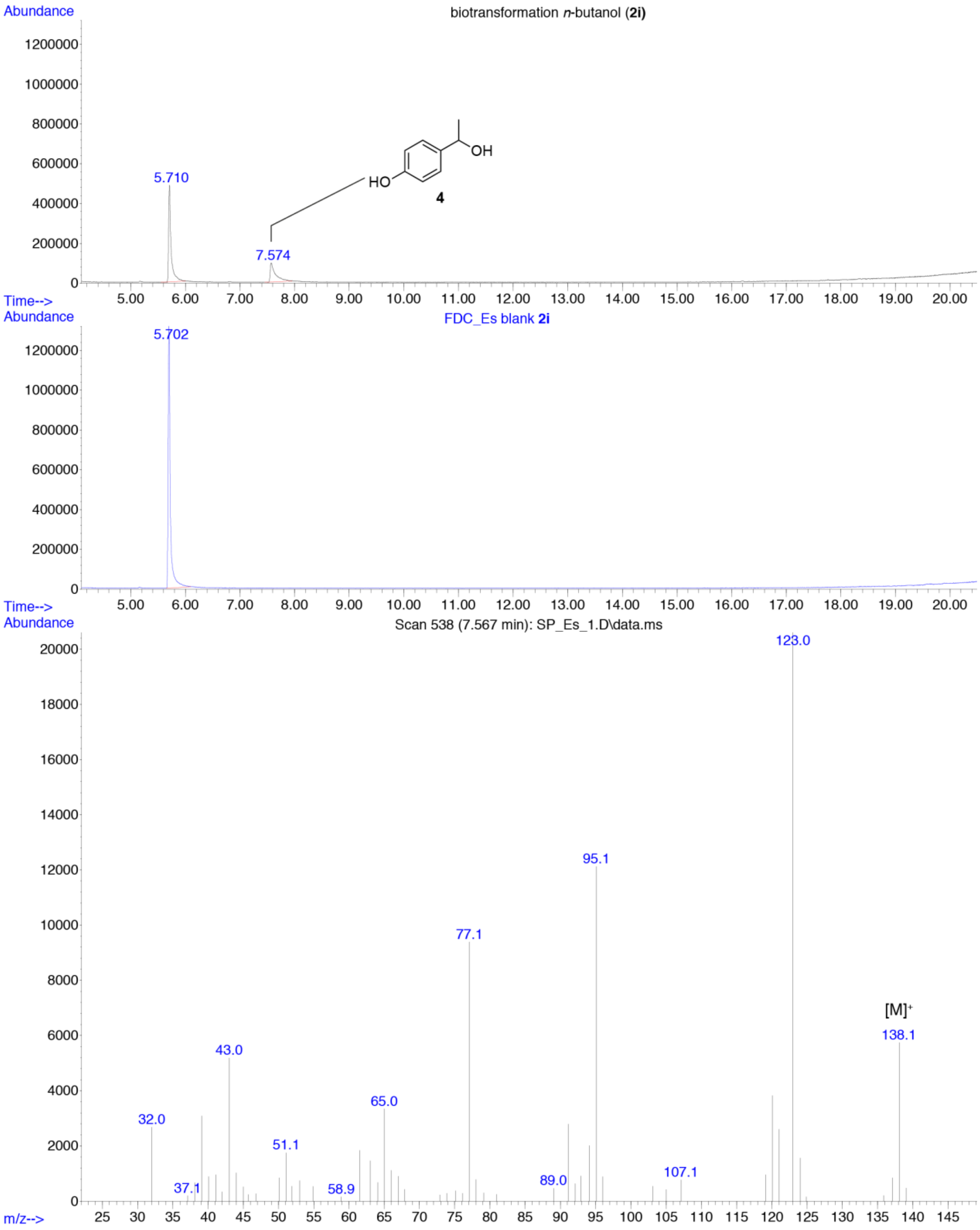

Figure S21. Biotransformation of **1** with *n*-butanol (**2i**) and FDC_*E*s as biocatalyst. Upper two panels: gas-chromatograms of the biotransformation and control run without biocatalyst. Lower panel: mass spectrum of hydrate **4** at 7.57 min.

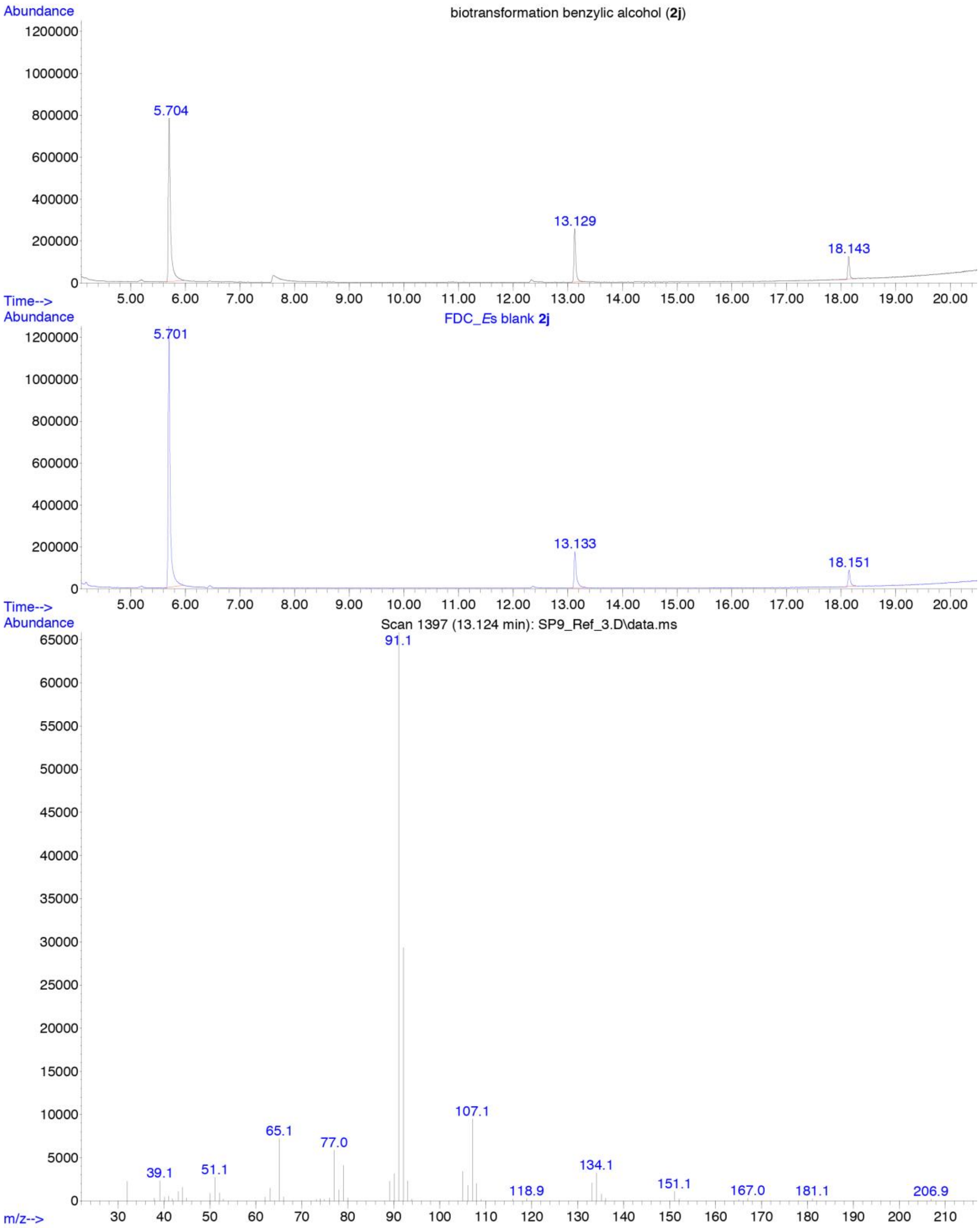

Figure S22. Biotransformation of **1** with benzyl alcohol (**2j**) and FDC_*E*s as biocatalyst. Upper two panels: gas-chromatograms of the biotransformation and control run without biocatalyst. Lower panel: mass spectrum of the unidentified peak at 13.12 min.

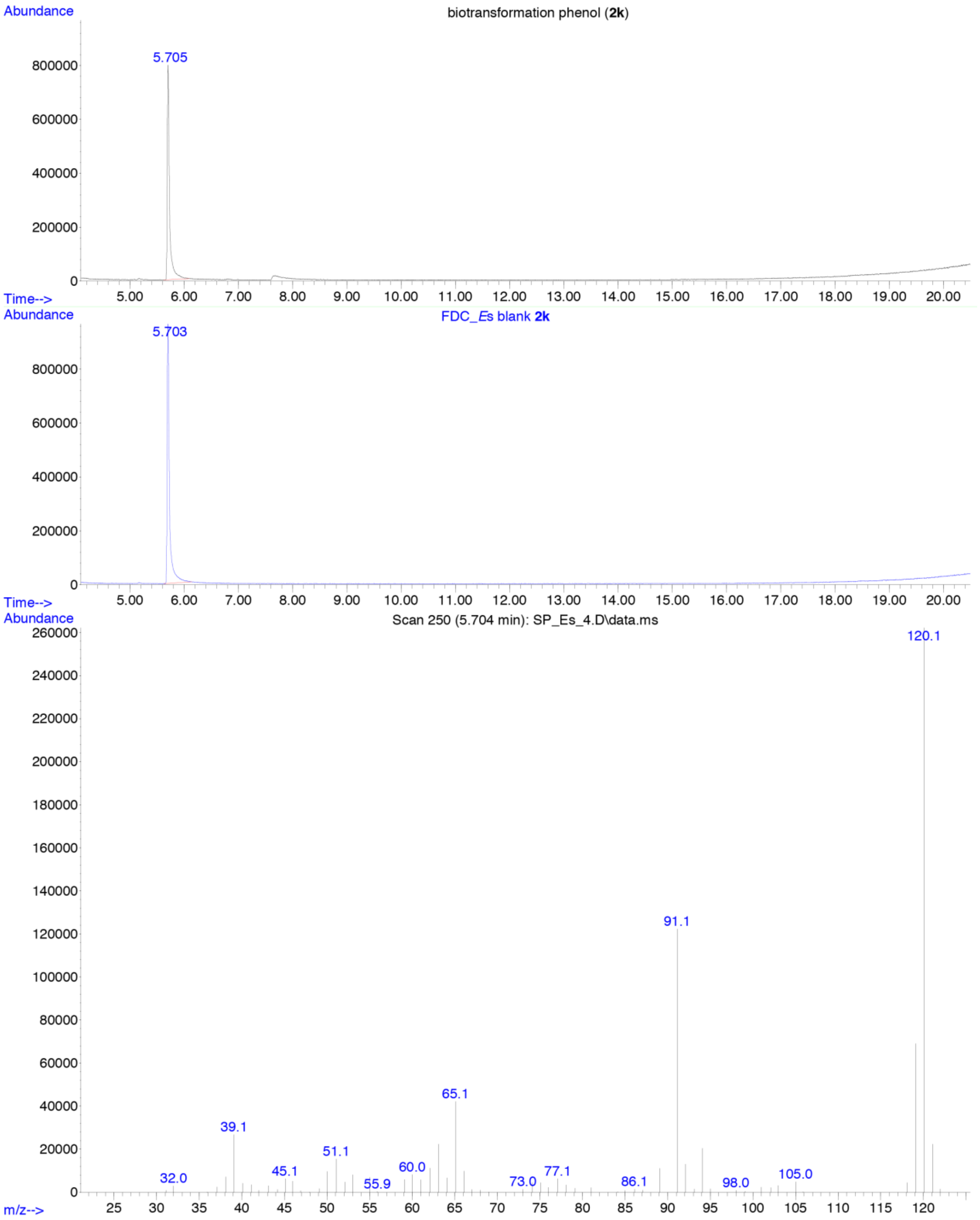

Figure S23. Biotransformation of **1** with phenol (**2k**) and FDC_*E*s as biocatalyst. Upper two panels: gas-chromatograms of the biotransformation and control run without biocatalyst. Lower panel: mass spectrum of substrate **1** at 5.70 min.

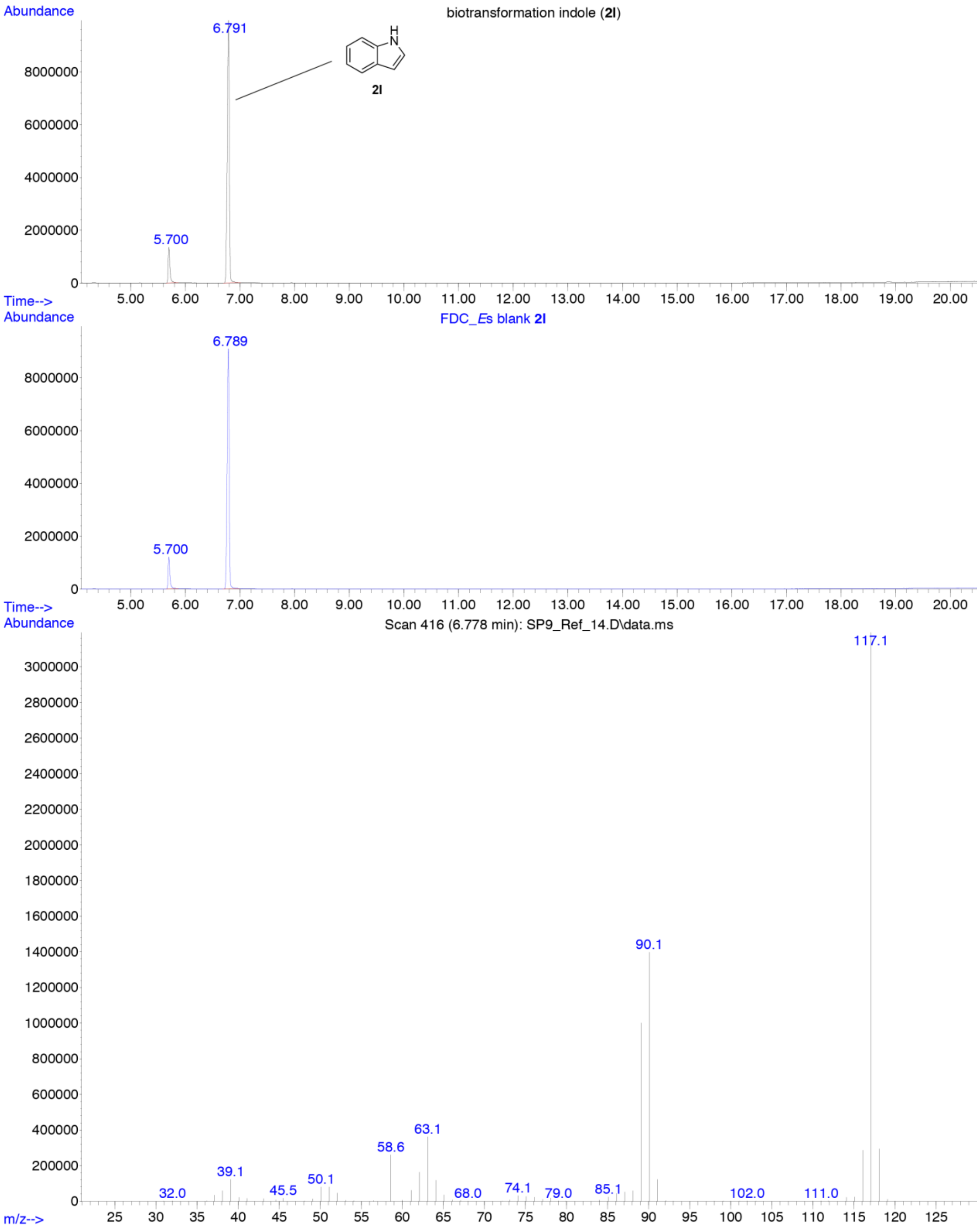

Figure S24. Biotransformation of **1** with indole (**2l**) and FDC_*E*s as biocatalyst. Upper two panels: gas-chromatograms of the biotransformation and control run without biocatalyst. Lower panel: mass spectrum of nucleophile **2l** at 6.78 min.

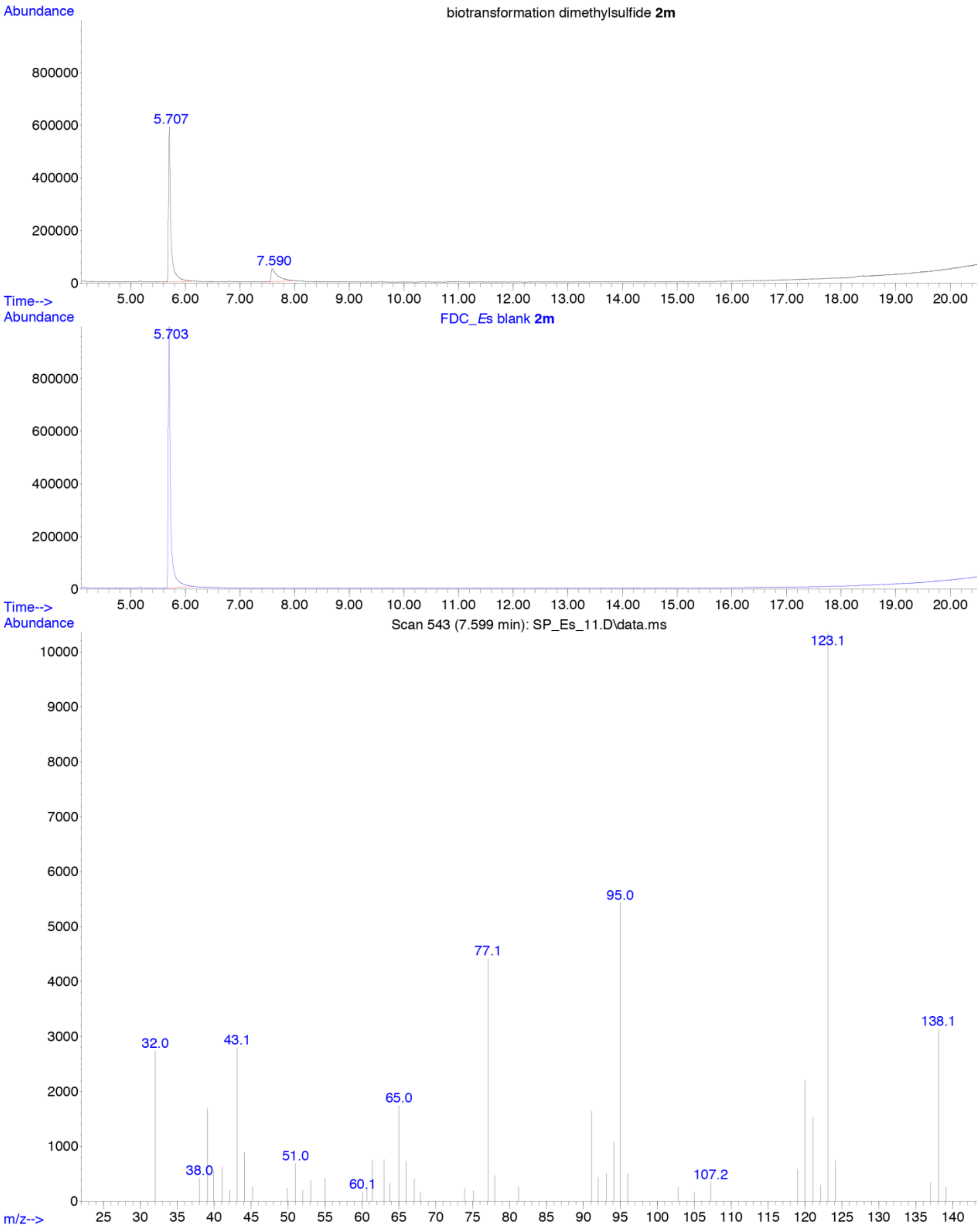

Figure S25. Biotransformation of **1** with dimethylsulfide (**2m**) and FDC_*E*s as biocatalyst. Upper two panels: gas-chromatograms of the biotransformation and control run without biocatalyst. Lower panel: mass spectrum of hydrate **4** at 7.60 min.

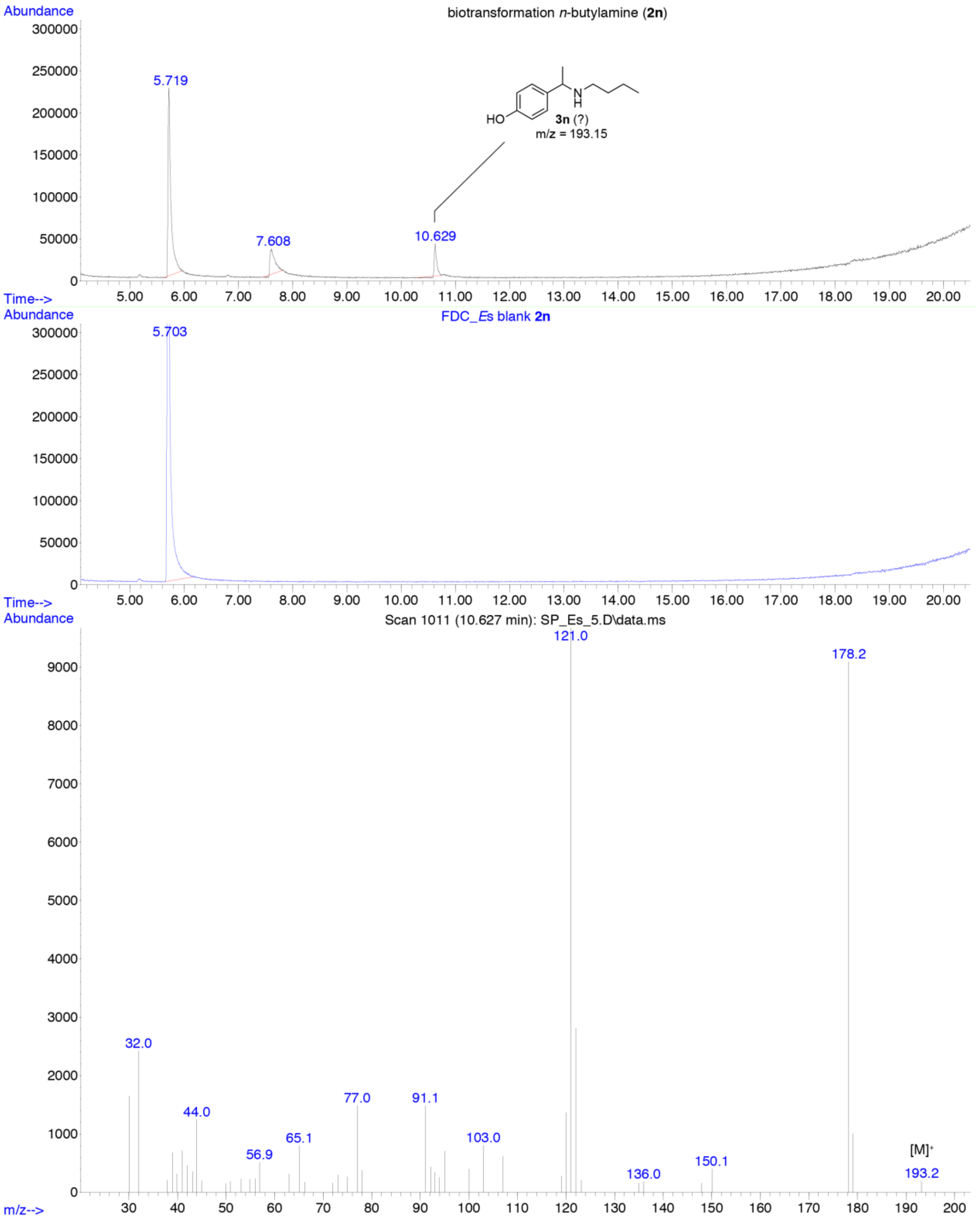

Figure S26. Biotransformation of **1** with *n*-butylamine (**2n**) and FDC_*E*s as biocatalyst. Upper two panels: gas-chromatograms of the biotransformation and control run without biocatalyst. Lower panel: mass spectrum of putative adduct **3n** at 10.63 min, which could not be reproduced in subsequent experiments.

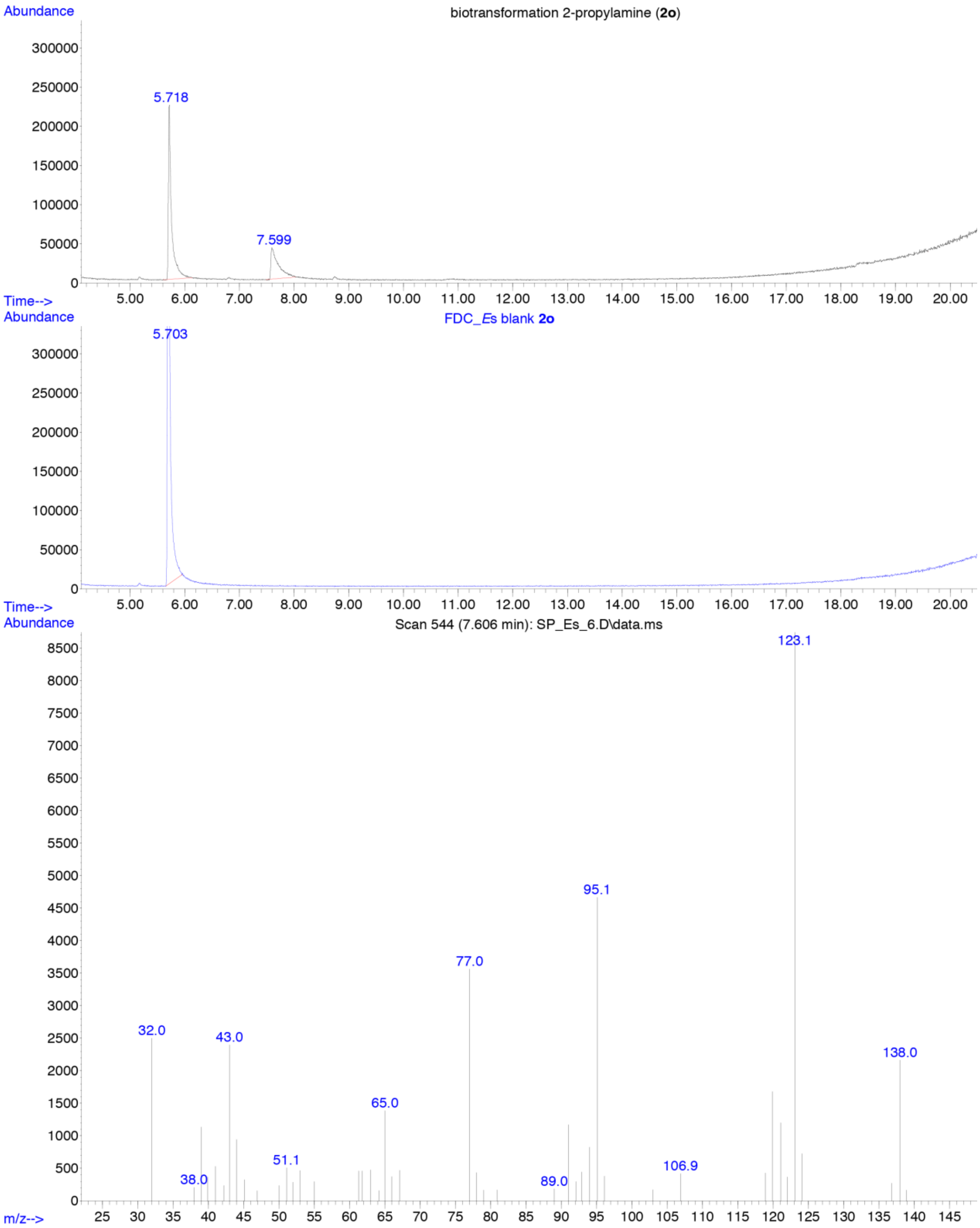

Figure S27. Biotransformation of **1** with 2-propylamine (**2o**) and FDC_*E*s as biocatalyst. Upper two panels: gas-chromatograms of the biotransformation and control run without biocatalyst. Lower panel: mass spectrum of hydrate **4** at 7.61 min.

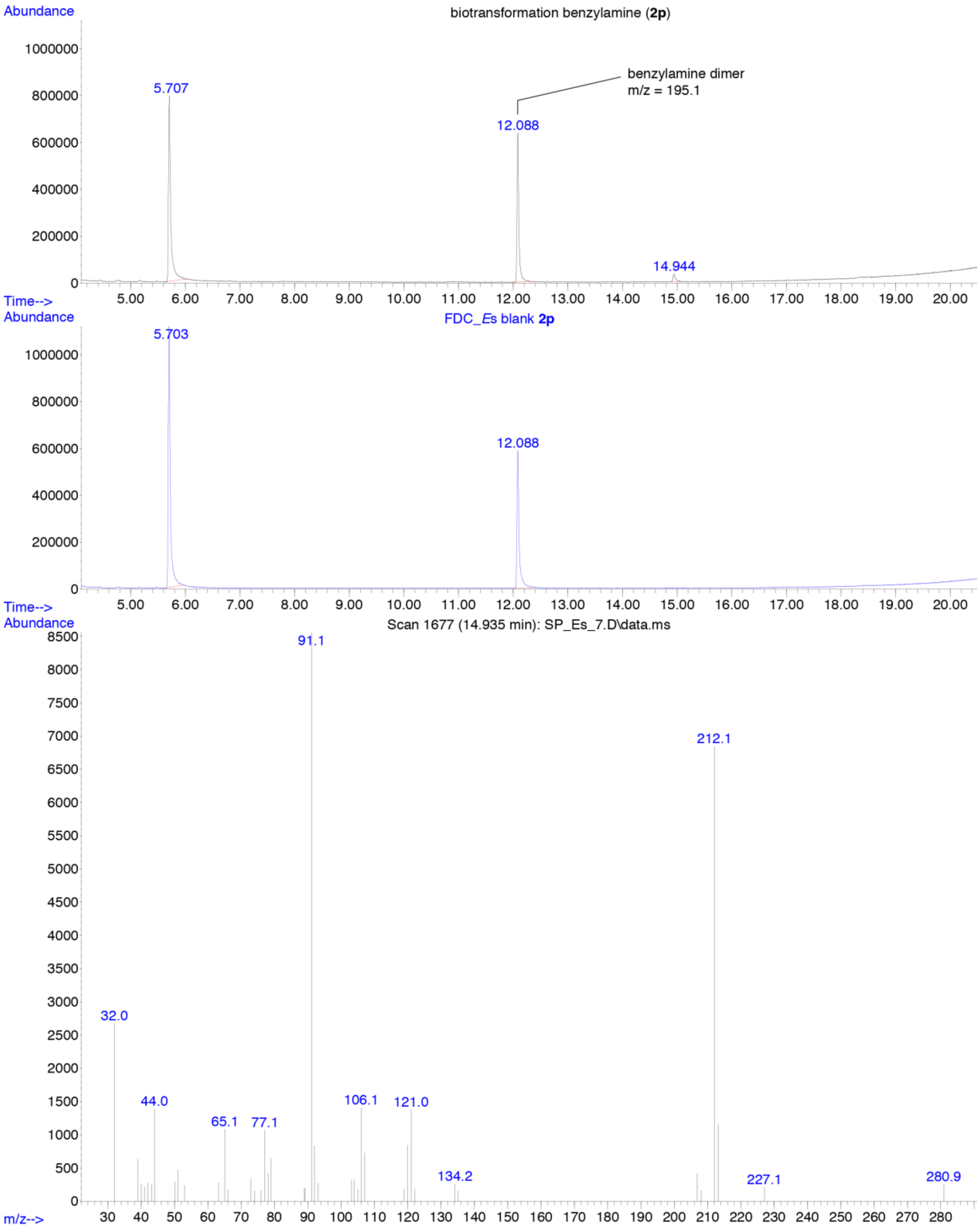

Figure S28. Biotransformation of **1** with benzylamine (**2p**) and FDC_*E*s as biocatalyst. Upper two panels: gas-chromatograms of the biotransformation and control run without biocatalyst. Lower panel: mass spectrum of an unidentified peak at 14.93 min.

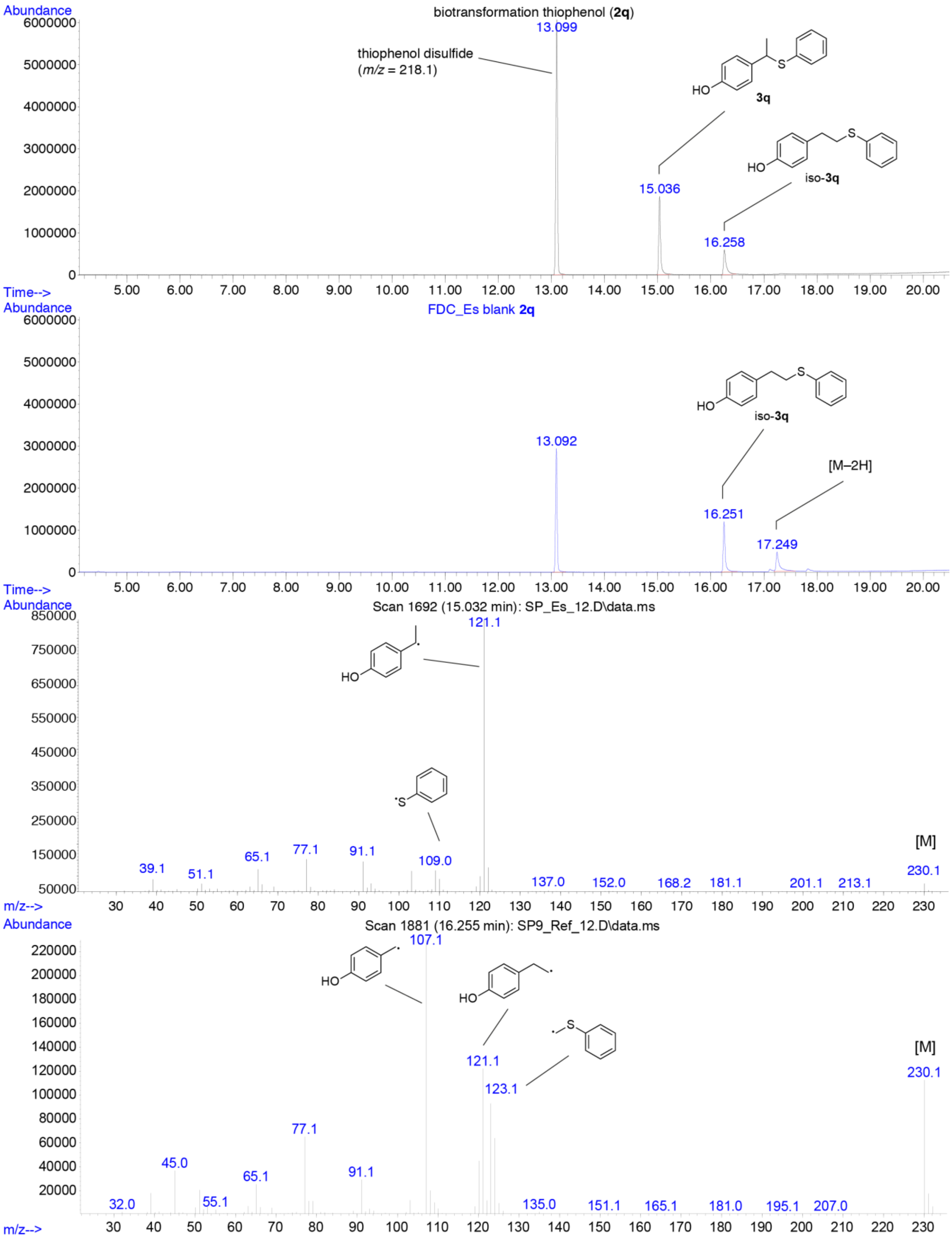

Figure S29. Biotransformation of **1** with thiophenol (**2q**) and FDC_*E*s as biocatalyst. Upper two panels: gas-chromatograms of the biotransformation and control run without biocatalyst. Lower two panels: mass spectra of adduct **3q** at 15.03 min and spontaneous adduct at 16.25 min. Main fragments hint at 1,6-addition product and thiol-ene reaction product respectively.

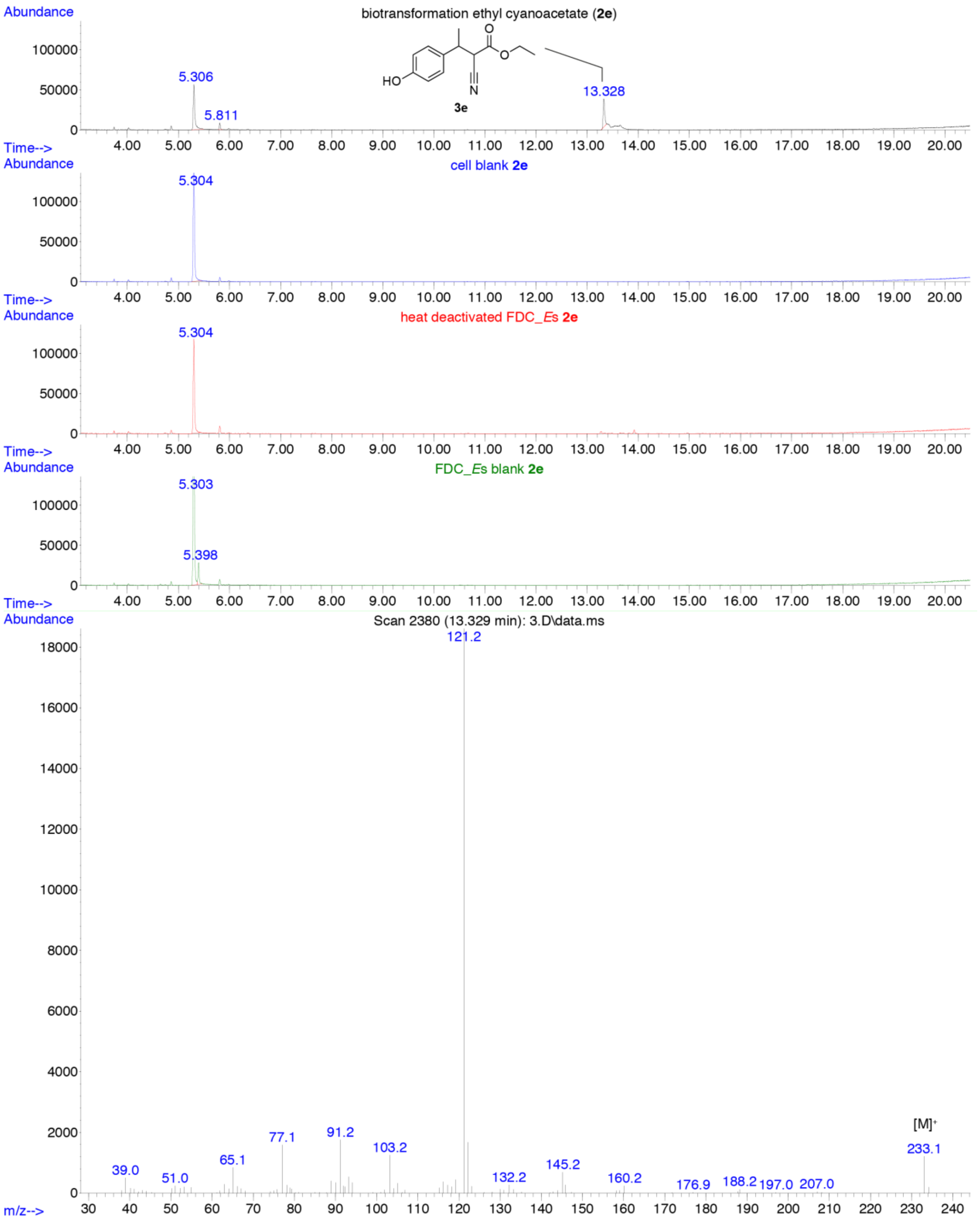

Figure S30. Biotransformation of **1** with ethyl cyanoacetate (**2e**) and FDC_*E*s as biocatalyst (10% v/v DME as co-solvent). Upper four panels: gas-chromatograms of the biotransformation and control runs with buffer only, heat-deactivated FDC_*E*s preparation and empty *E. coli* host cells, respectively. Lower panel: mass spectrum of putative adduct **3e** at 13.33 min.

HPLC (aqueous, achiral)

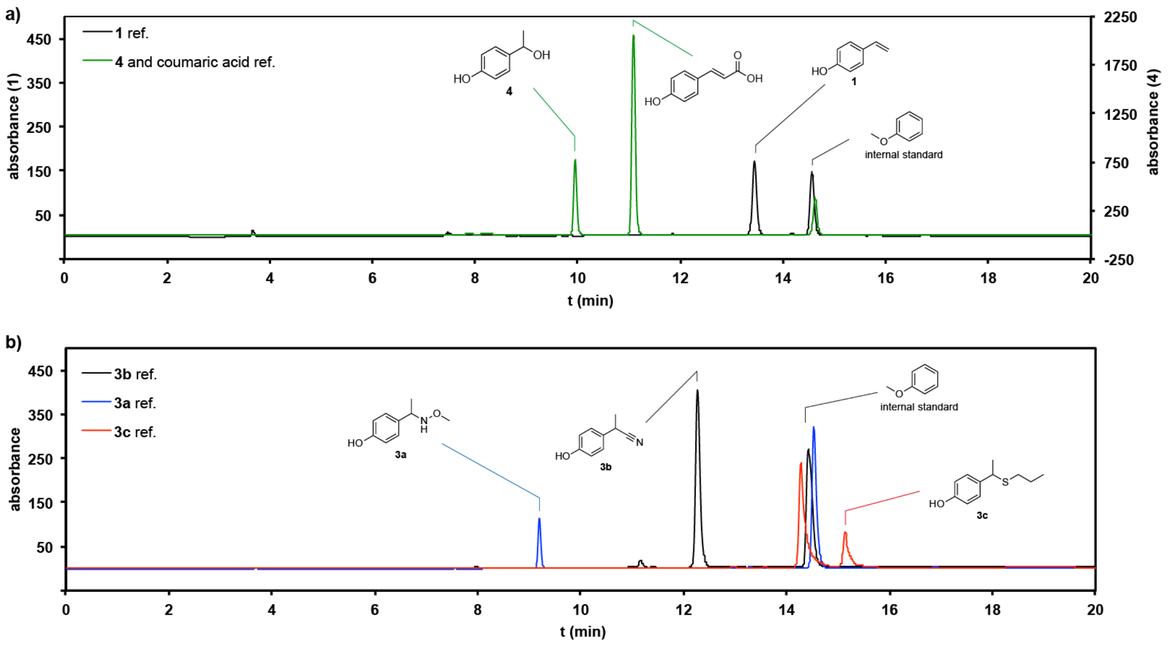

Figure S31. HPLC traces of a) 4-vinylphenol **1**, hydrate **4** and *p*-coumaric acid; b) nucleophile adducts **3a** – **3c** with anisole as internal standard in all samples (UV traces at 270 nm).

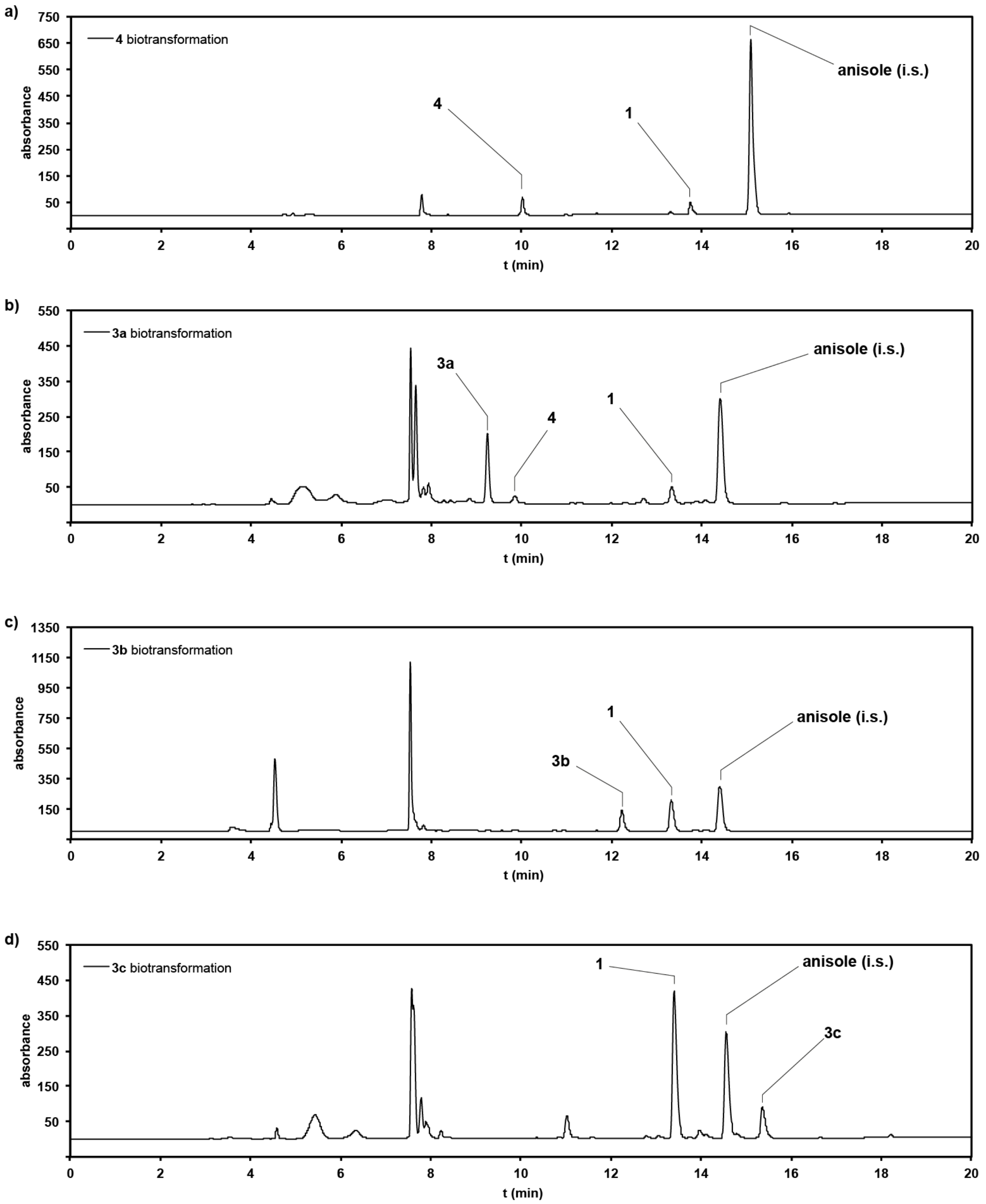

Figure S32. Representative chromatograms of the biocatalytic addition of a) water, b) methoxyamine (**2a**), c) cyanide (**2b**) and d) propanethiol (**2c**) across 4-vinylphenol **1** (UV traces at 270 nm).

Table S6. Summary of HPLC calibration data for biotransformation components.

Compound	*t_R [min]*	*k*[(a)]	*R^2*	*Range [mM]*
1	*13.0*	*5.37*	*0.99933*	*0.2 – 10*
3a	*9.2*	*0.555*	*0.99999*	*0.2 – 20*
3b	*12.3*	*0.672*	*0.99998*	*0.2 – 20*
3c	*15.1*	*0.672*	*0.99869*	*0.5 – 30*
4	*9.9*	*0.719*	*0.99995*	*0.5 – 20*
2w	*12.4*[(b)]	*1.07*[(c)]	*0.99975*	*0.5 – 25*
***2u** isomer 1*	*20.4*[(d)]	*1.35* [(c)]	*0.99979*	*0.25 – 5*
***2u** isomer 2*	*20.9*[(d)]	*1.01* [(c)]	*0.99973*	*0.25 – 5*

[(a)] Response factor derived from a calibration curve of the form: area(analyte)/area(i.s.) = k · [analyte]/[i.s] where "i.s." is the internal standard anisole with const. concentration (10 mM). [(c)] Integration at 220 nm. (d) Modified HPLC-method: 0 – 2 min (100% A), 2 – 20 min (100 – 40% A), 20 – 23 min (40% A), 23 – 24 min (40 – 0% A), 24 – 26 min (0% A), 26 – 28 min (0 – 100% A), 28 – 30 min (100% A).

HPLC (organic, chiral)

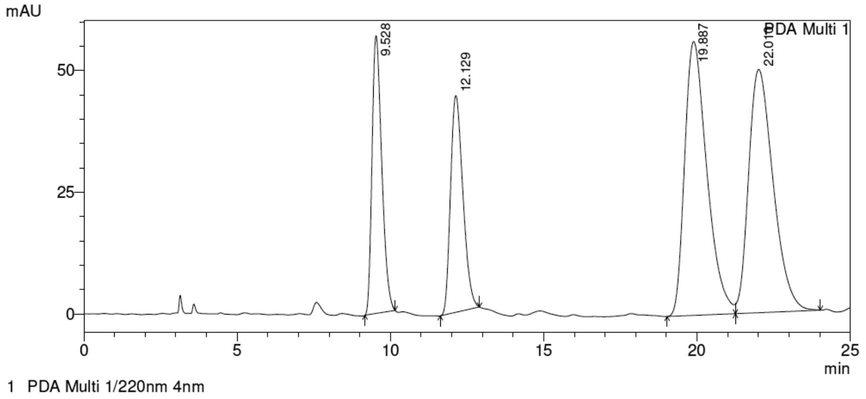

Figure S33. HPLC-trace of a mixture of racemic reference compounds **3a** and **4** (Chiralcel OD-H; heptane/2-PrOH = 93:7).

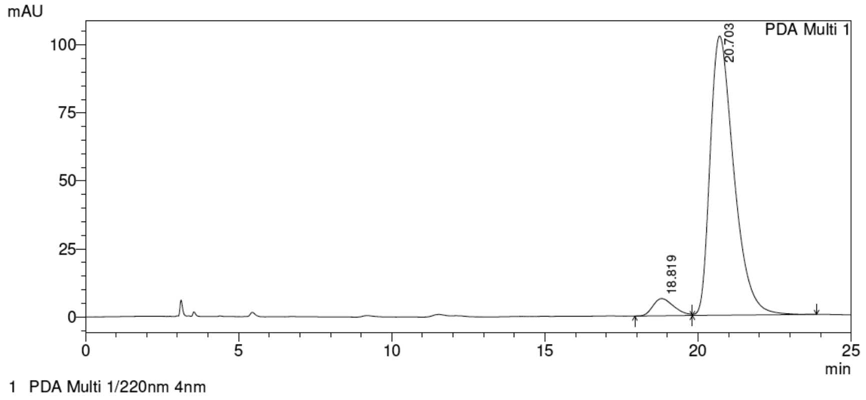

Figure S34. HPLC-trace of enantiomer-enriched reference sample of (*S*)-**4** (Chiralcel OD-H; heptane/2-PrOH = 93:7).

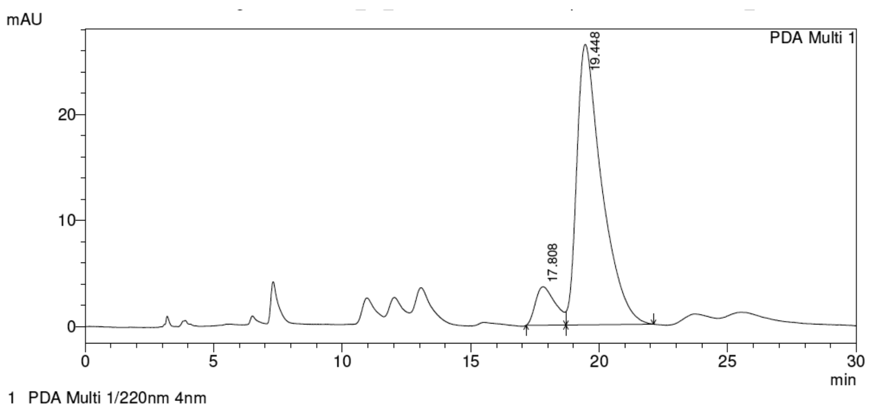

Figure S35. HPLC-trace of a biotransformation containing enantiomer-enriched (*S*)-**4** as product (Chiralcel OD-H; heptane/2-PrOH = 93:7).

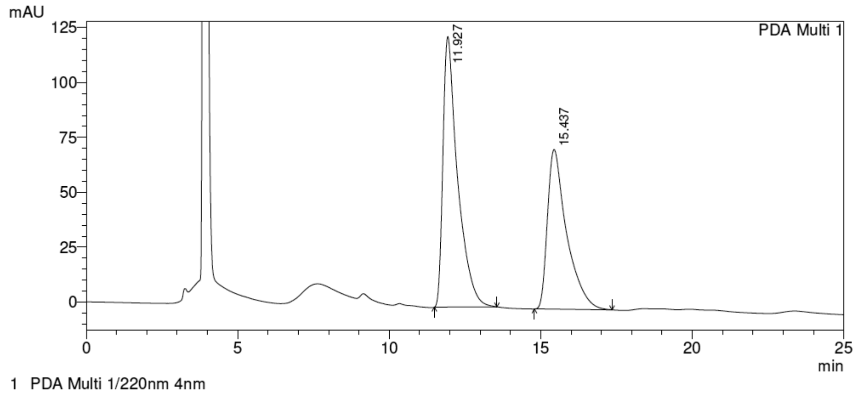

Figure S36. HPLC-trace of a racemic reference sample of **3a** (Chiralcel OD-H; heptane/2-PrOH = 95:5).

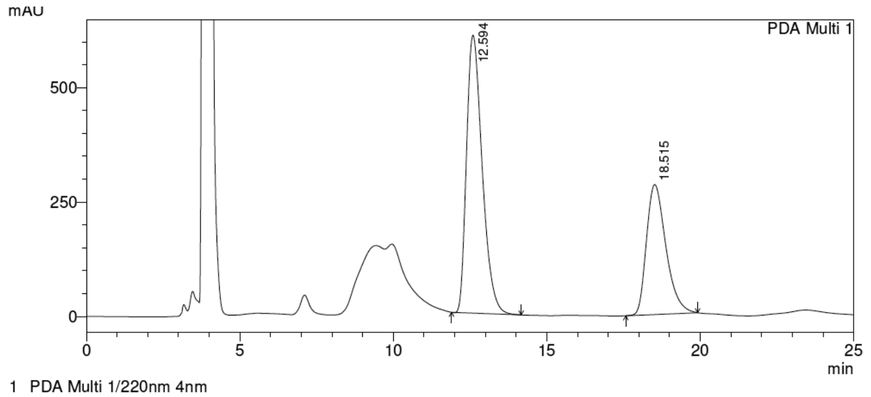

Figure S37. HPLC-trace of a biotransformation containing enantiomer-enriched (*S*)-**3a** as product (Chiralcel OD-H; heptane/2-PrOH = 95:5).

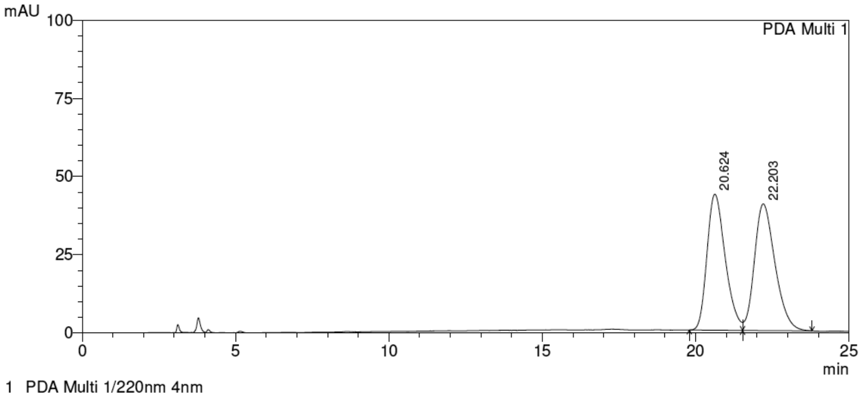

Figure S38. HPLC-trace of a racemic reference sample of **3c** (Chiralcel OD-H; heptane/2-PrOH = 98.5:1.5).

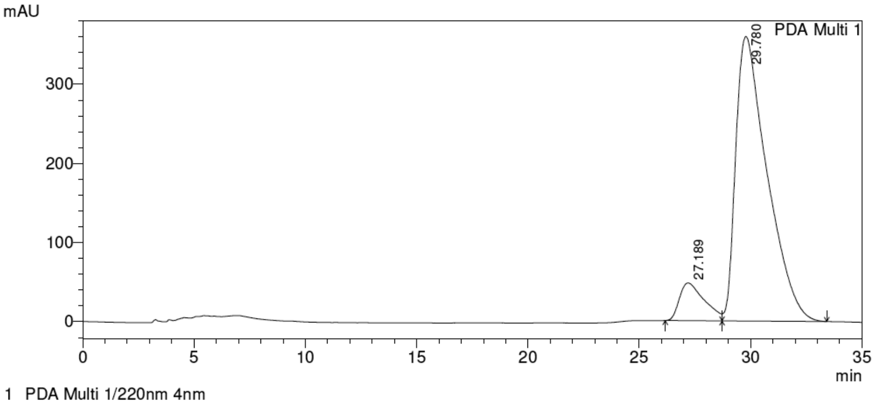

Figure S39. HPLC-trace of a biotransformation containing enantiomer-enriched (*S*)-**3c** as product (Chiralcel OD-H; heptane/2-PrOH = 99:1).

GC-FID (chiral)

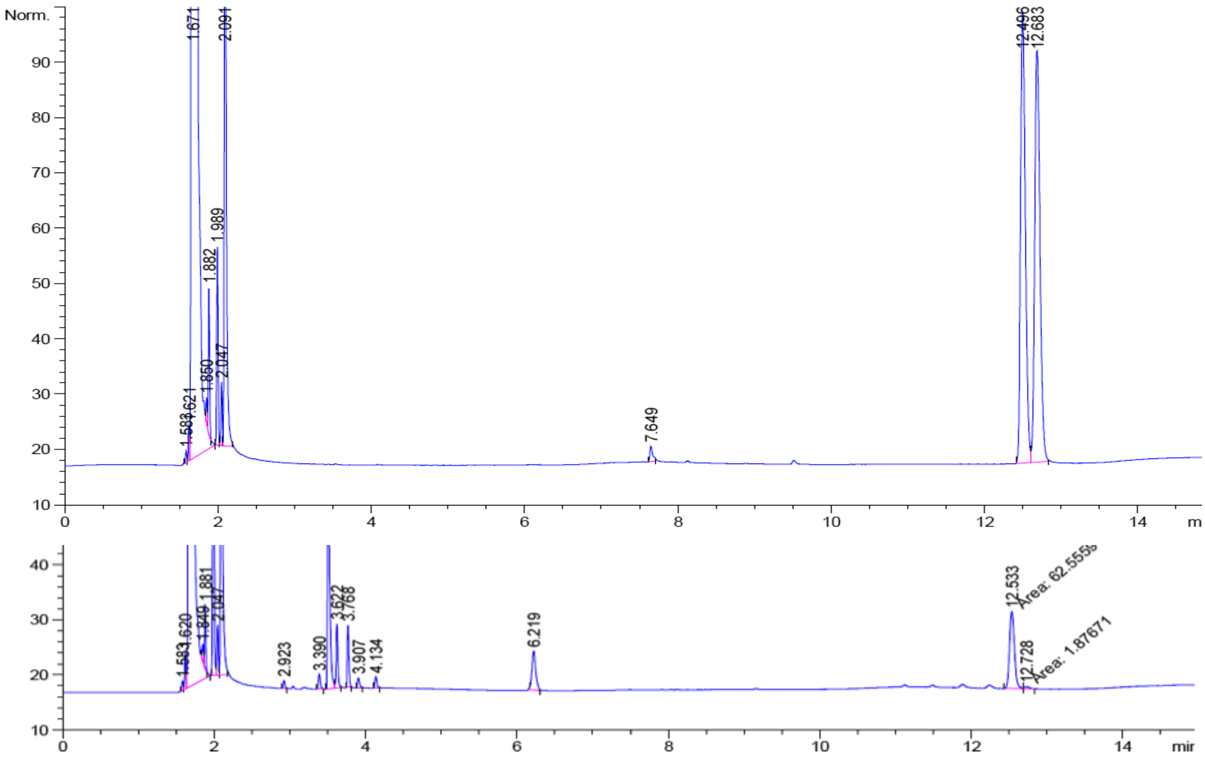

Figure S40. Upper panel: GC-FID-trace of a racemic reference sample of **3b** after derivatization *via O*-acetylation (*cf*. general information) as product. Lower panel: GC-FID trace of a biotransformation containing (*S*)-**3b** as product (after derivatization)

^{1}H-NMR spectra

rac-**4** reference compound

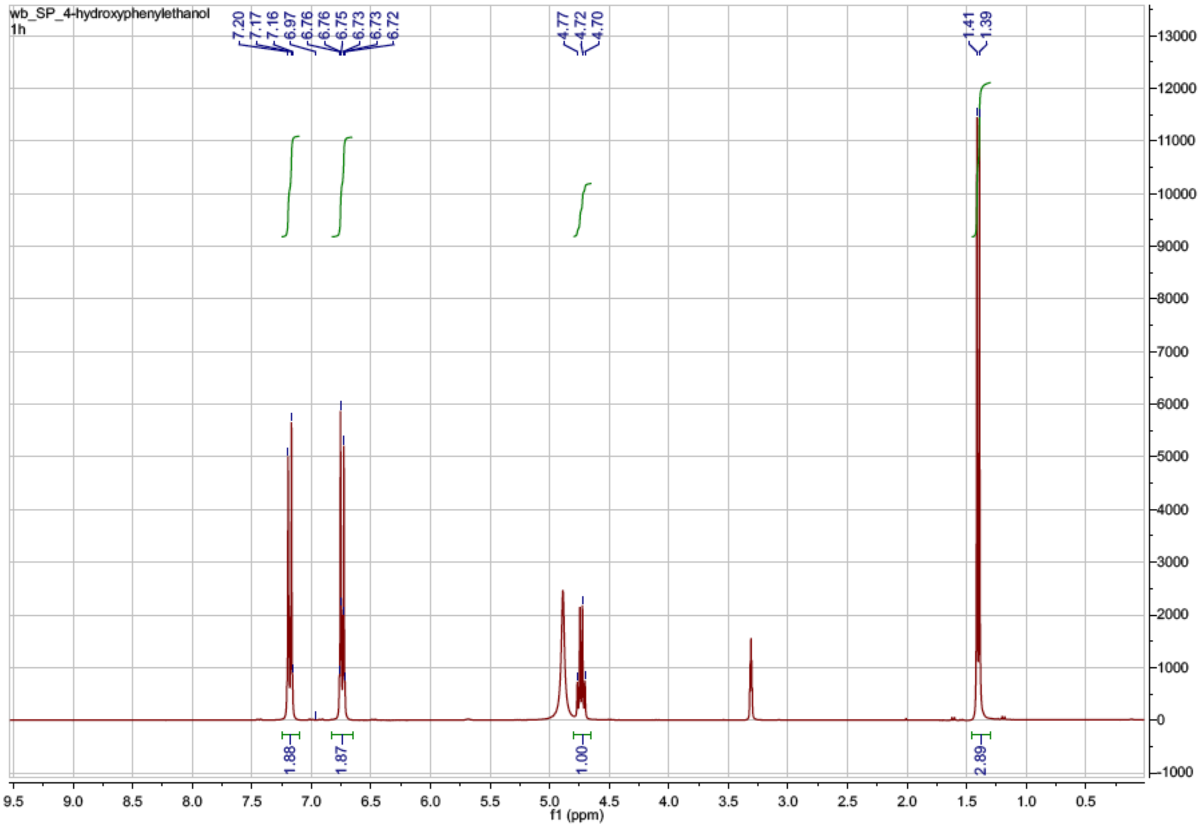

rac-**3a** reference compound

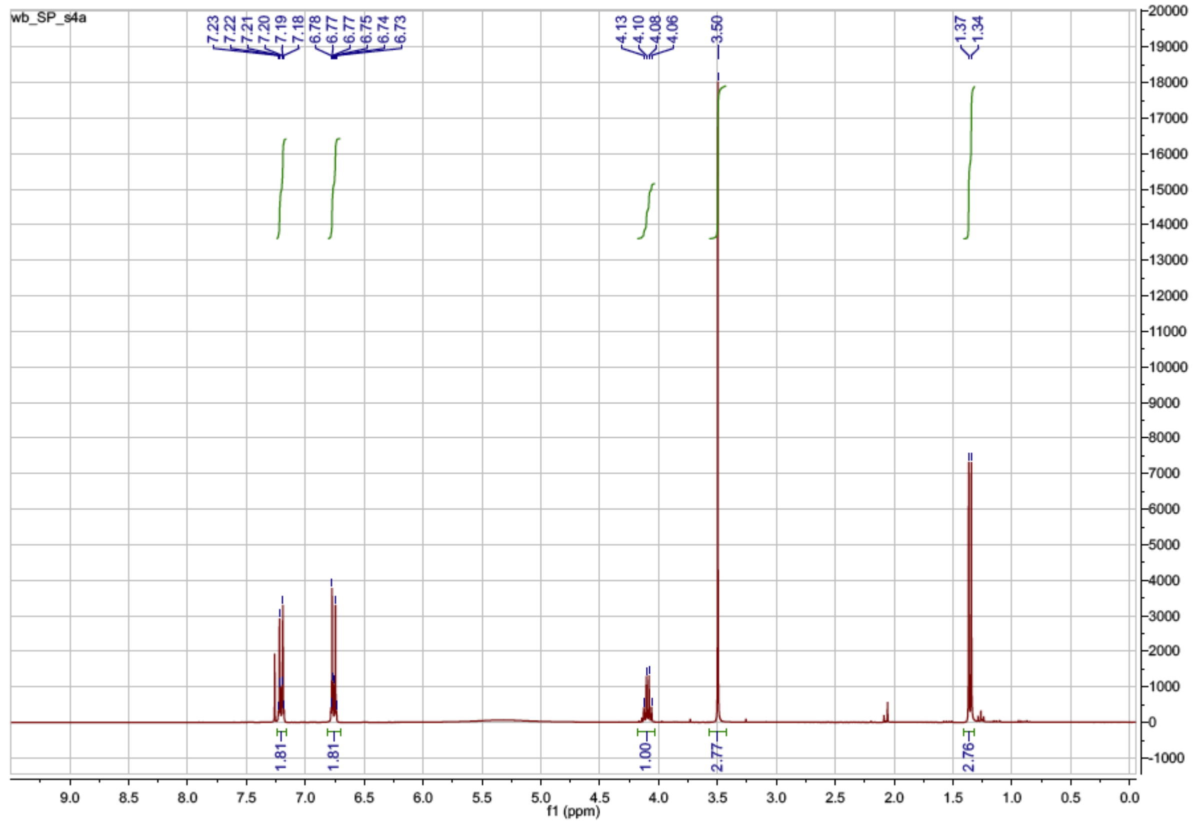

(*S*)-**3a** from biotransformation

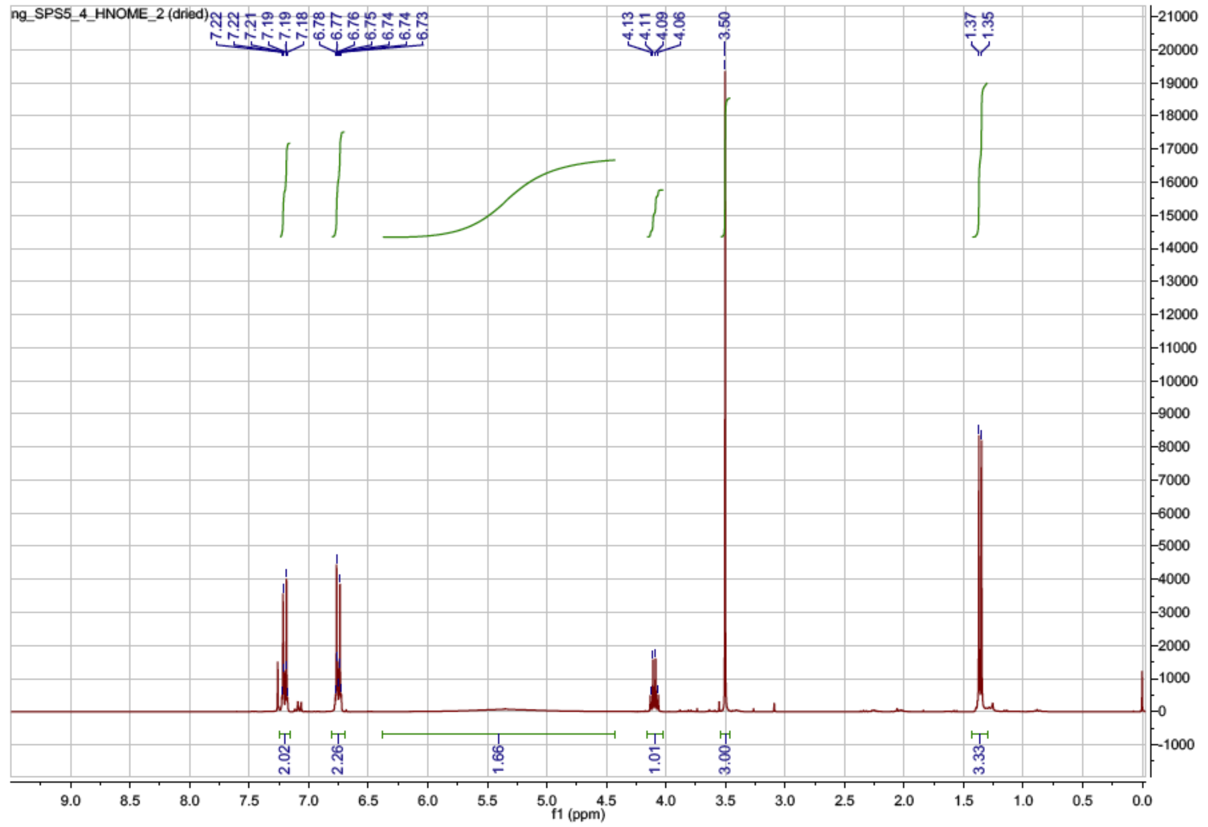

rac-**3b** reference compound

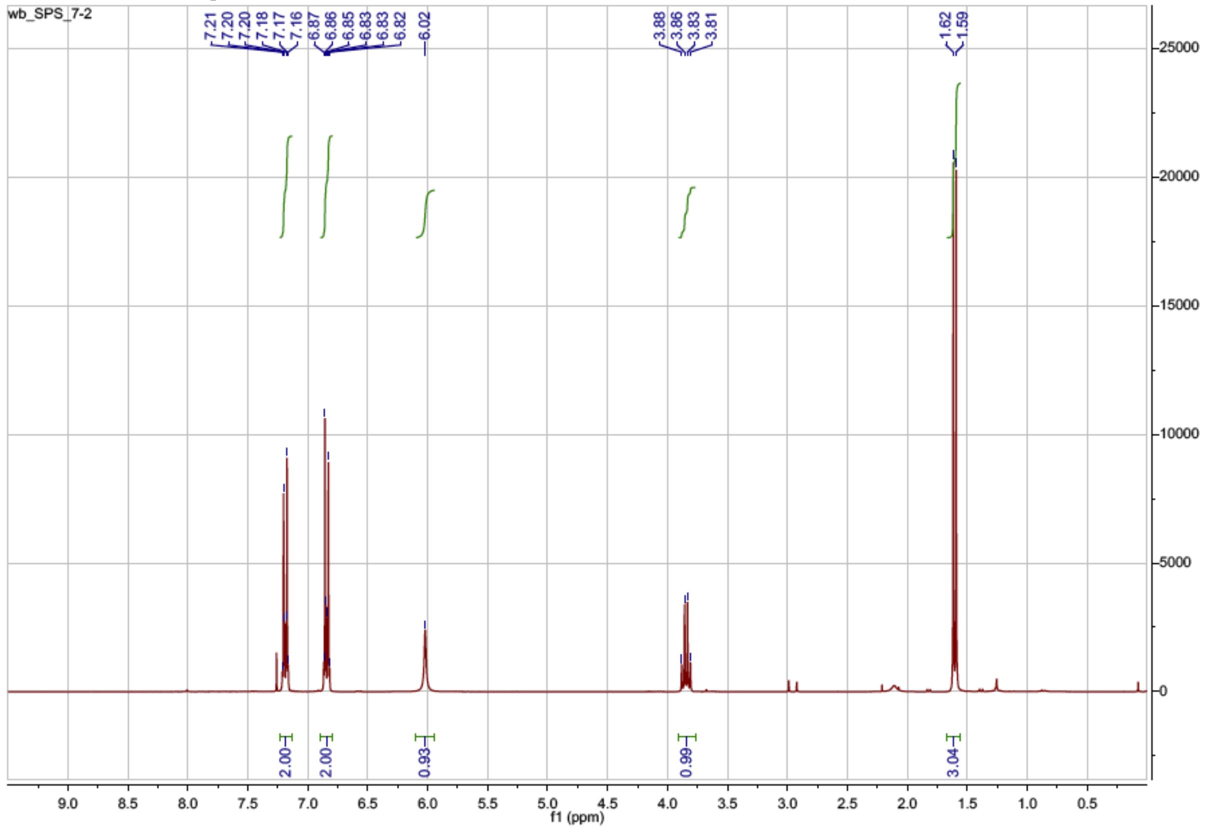

(*S*)-**3b** reference compound

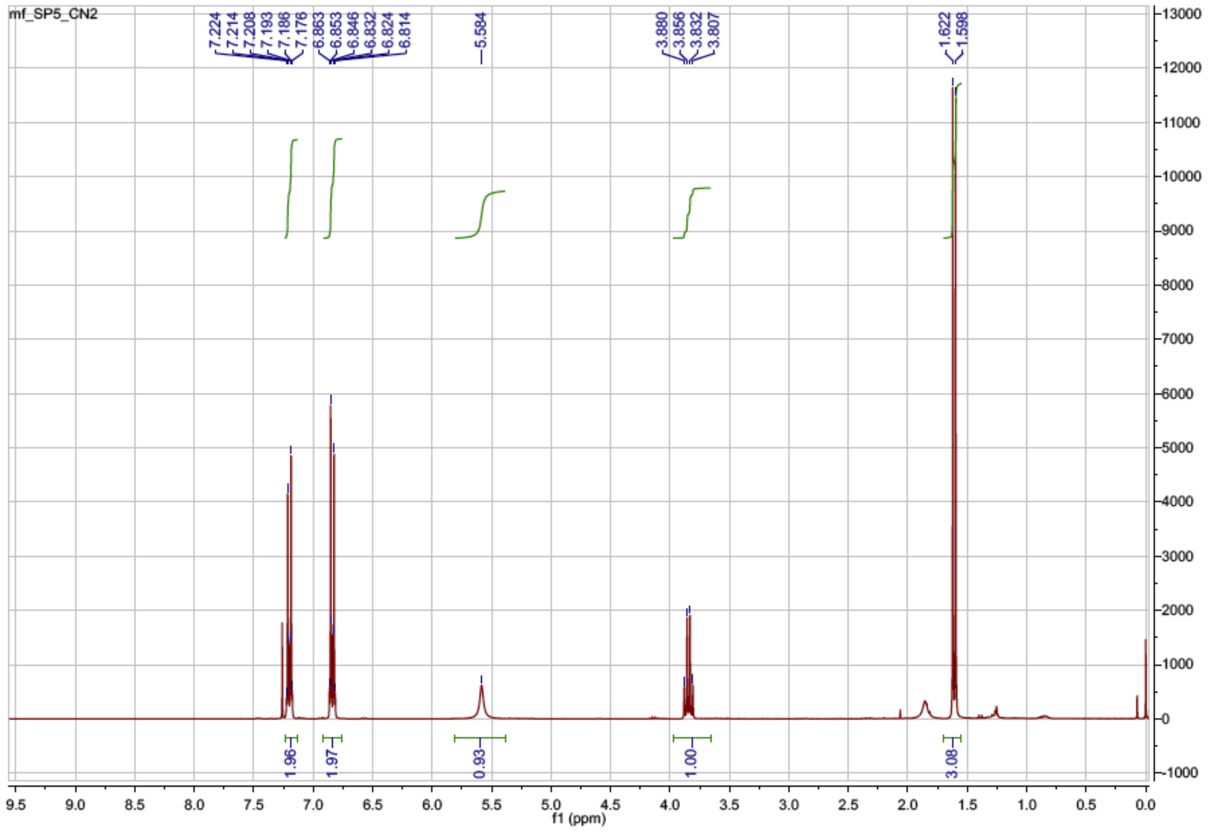

rac-**3c** reference compound

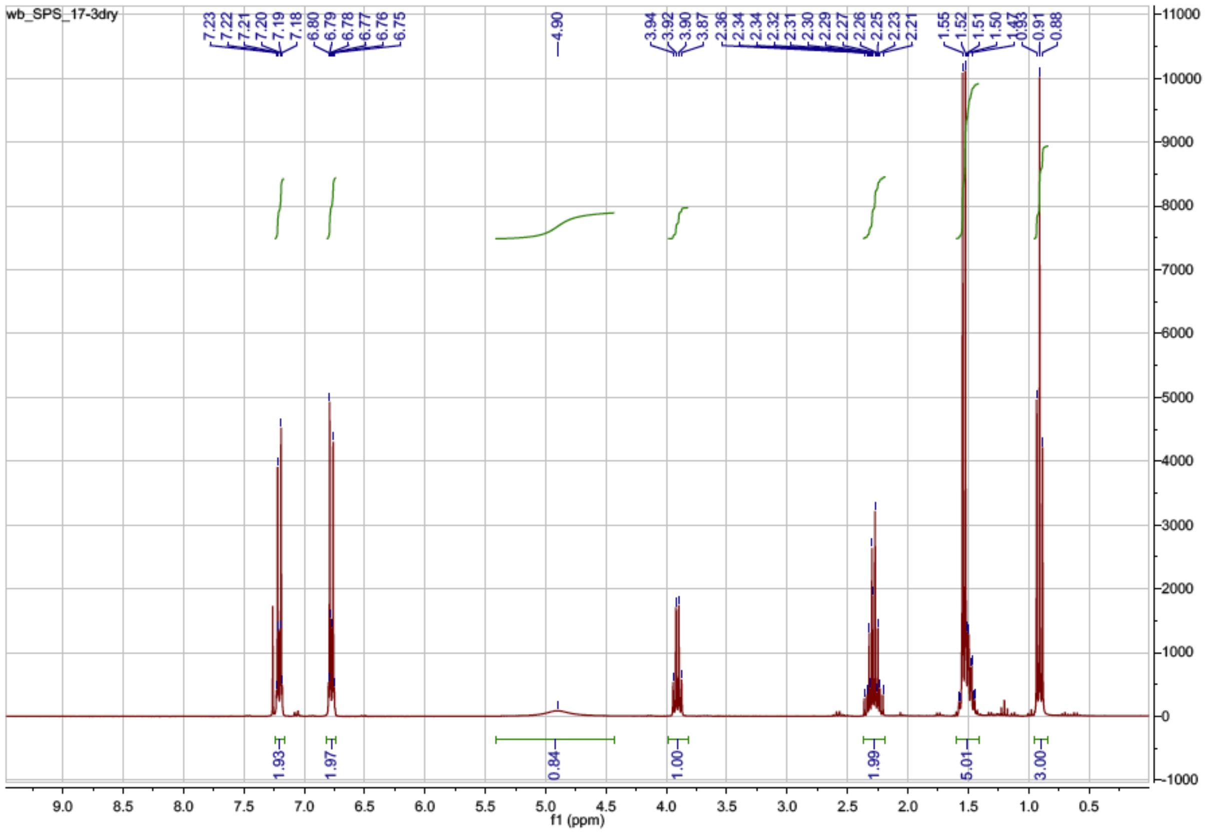

(*S*)-**3c** from biotransformation

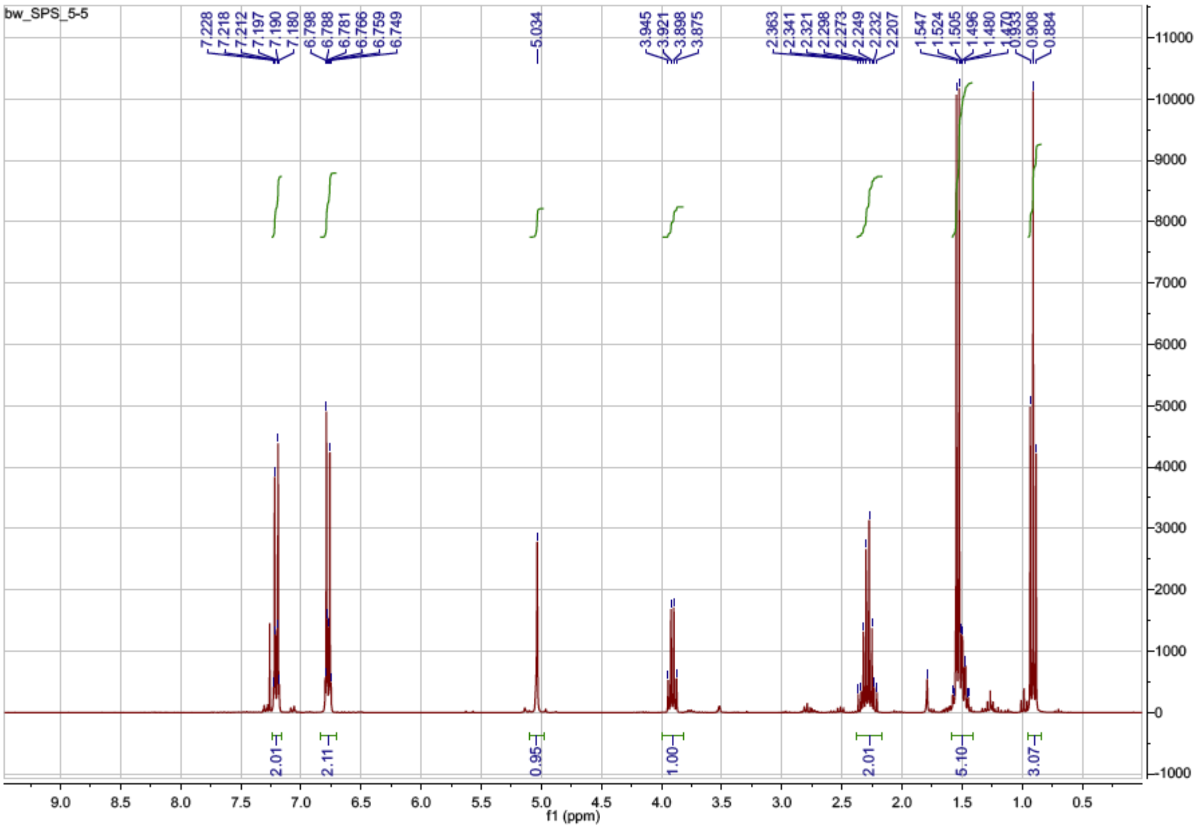

all-*rac*-**3e** reference compound (and traces of **2e**) (mixture of 2 diastereoisomers)

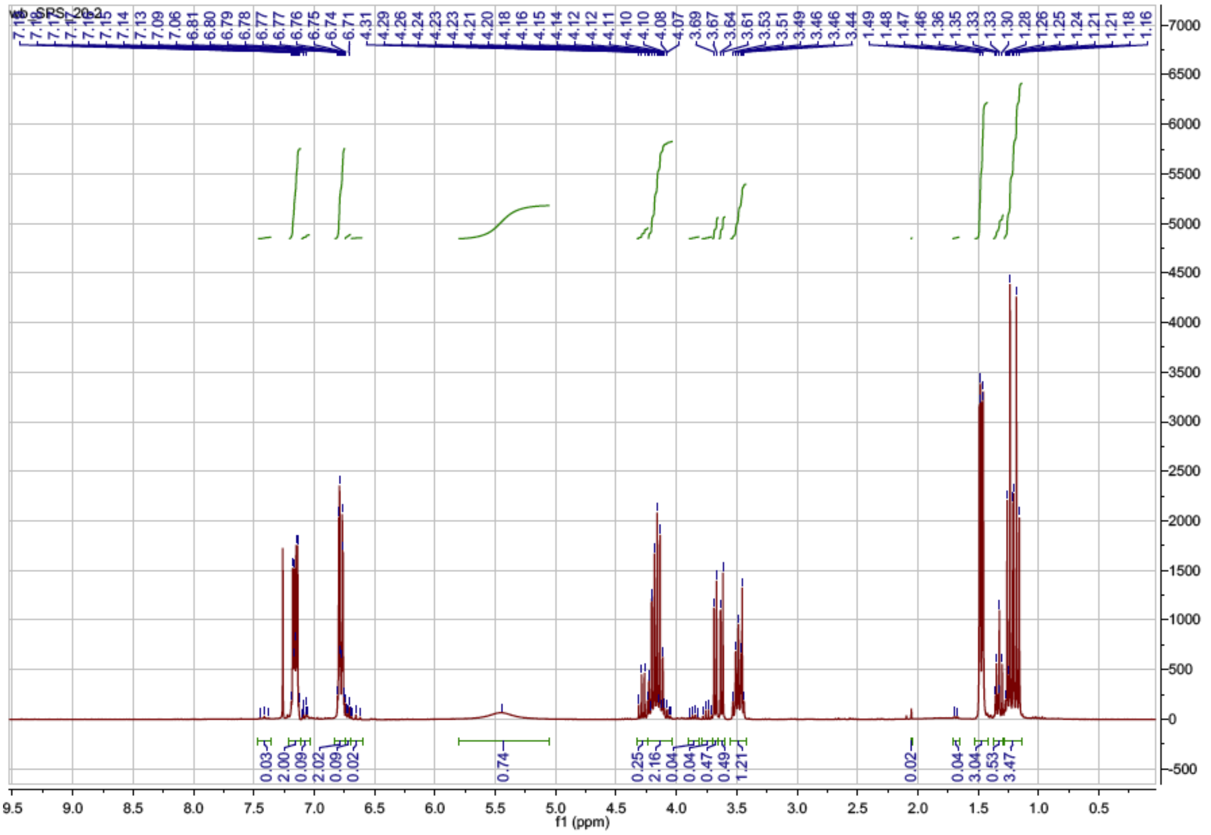

^{13}C-NMR Spectra

4 (reference compound)

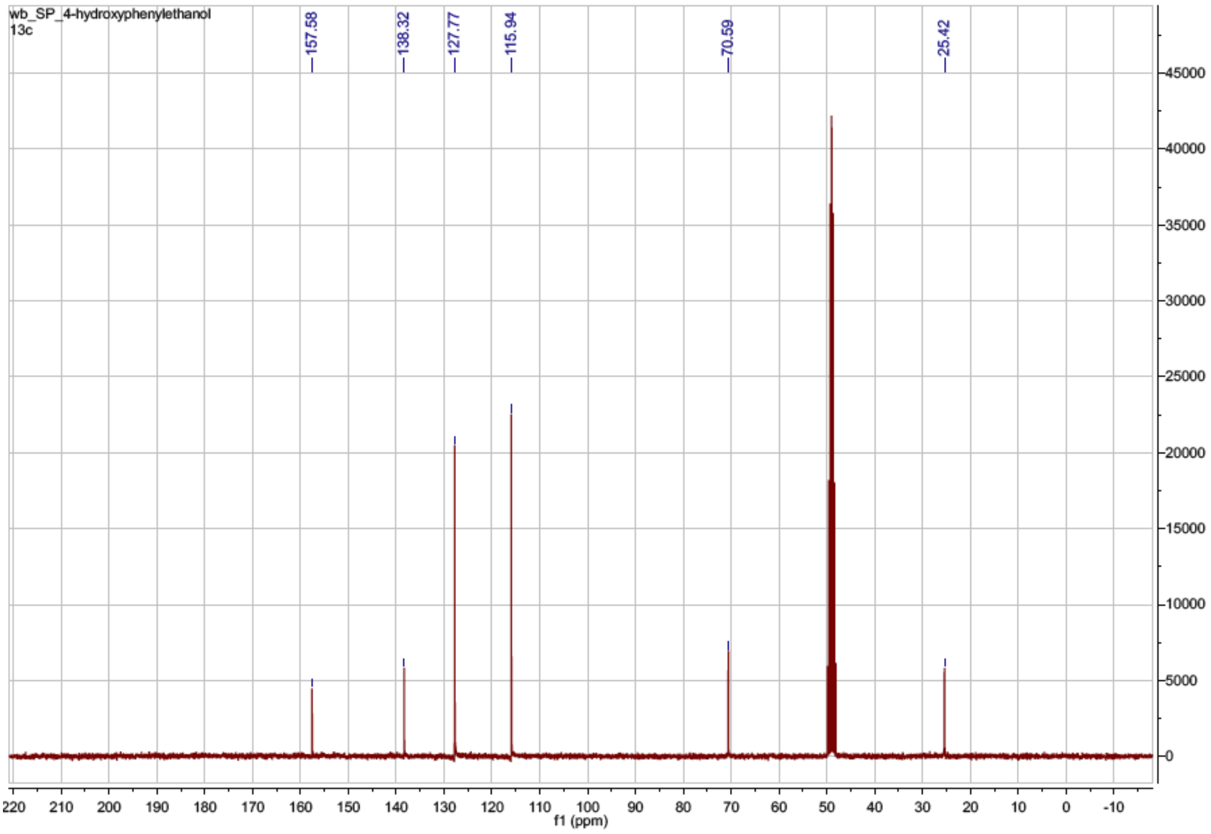

3a (biotransformation product)

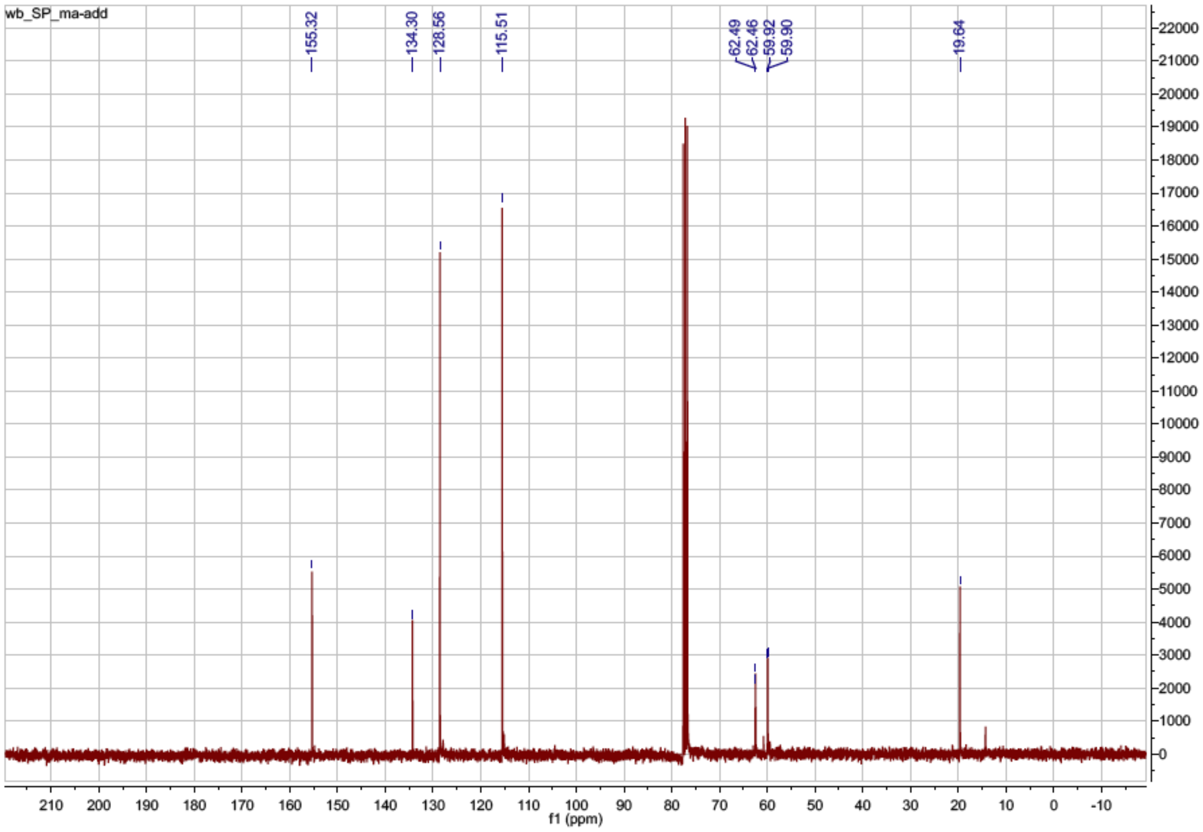

3b (biotransformation product)

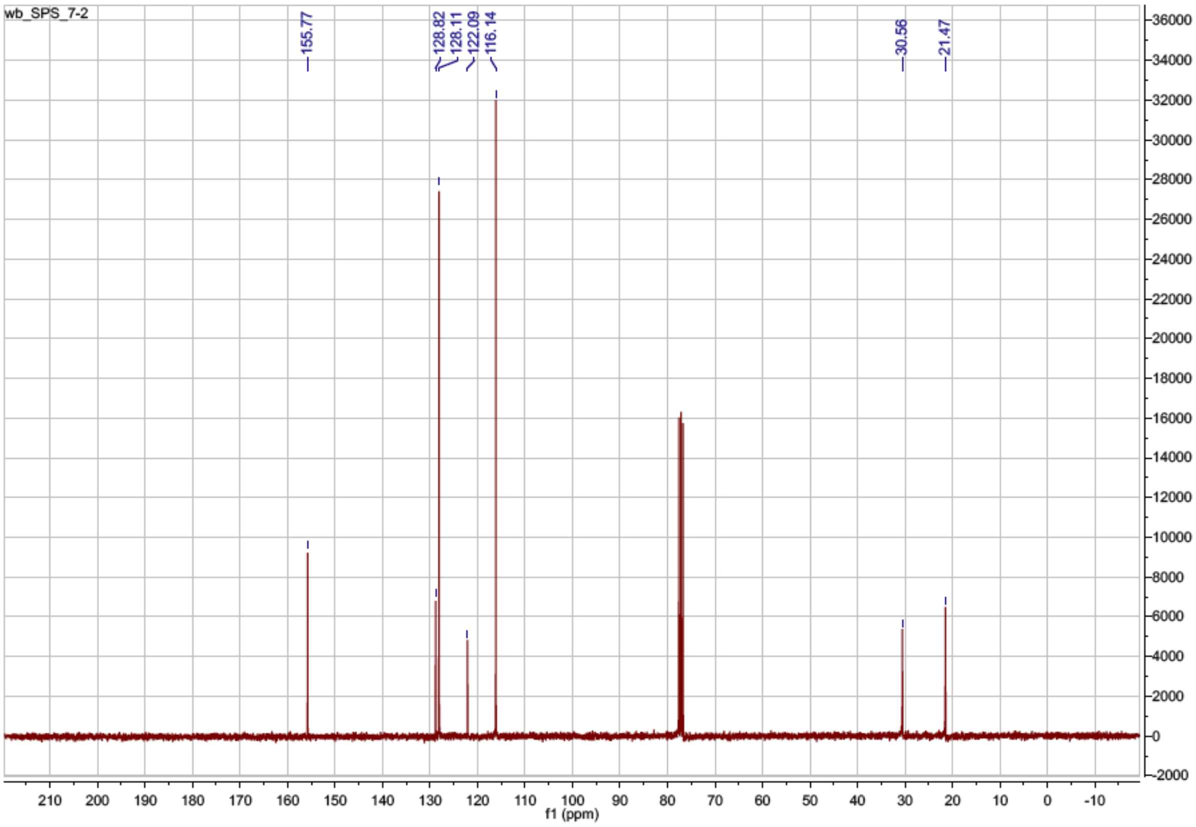

3c (biotransformation product)

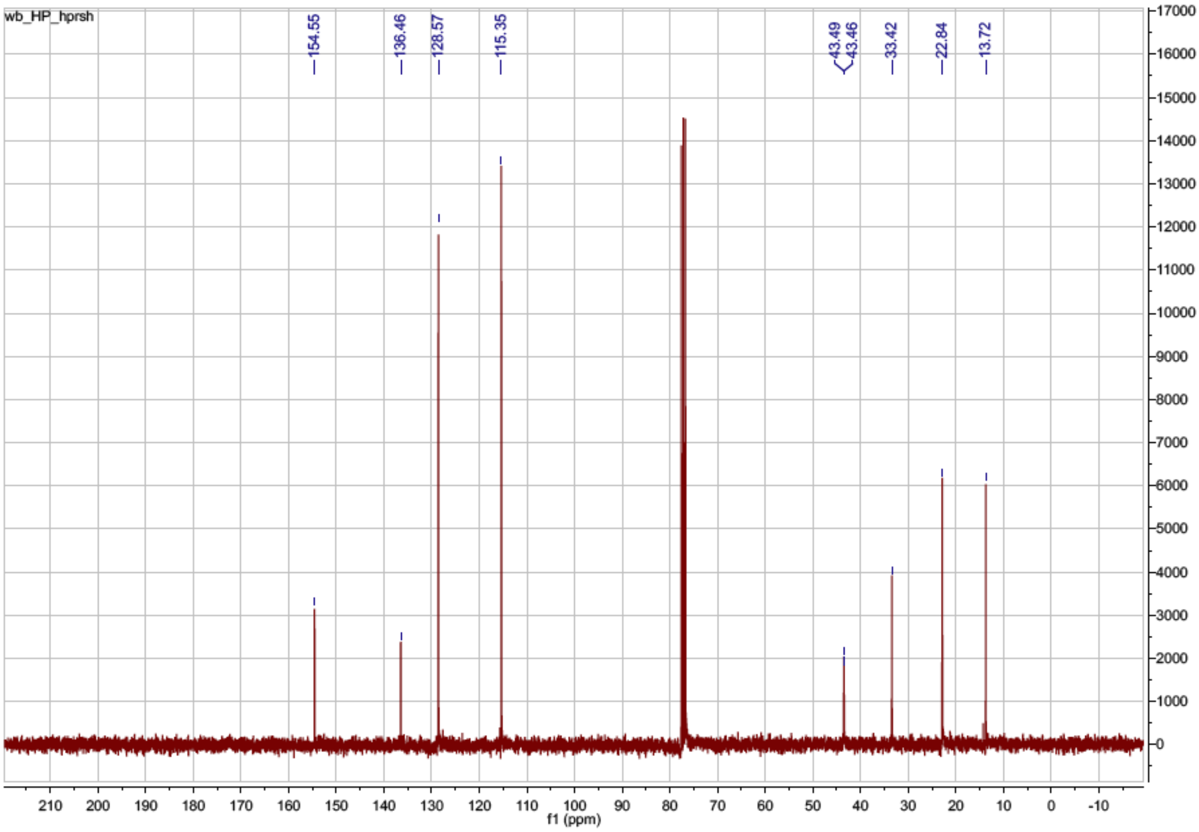

3e (reference compound, mixture of diastereoisomers)

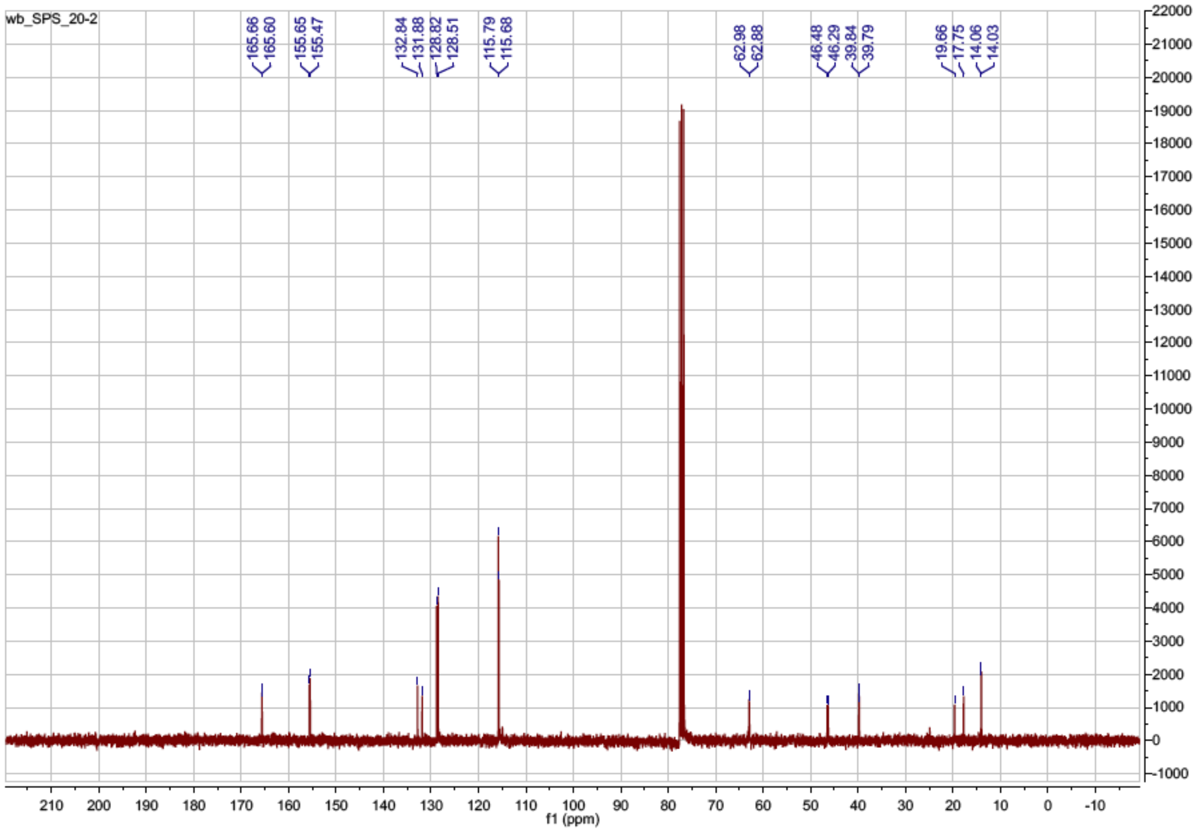

HR-MS Spectra

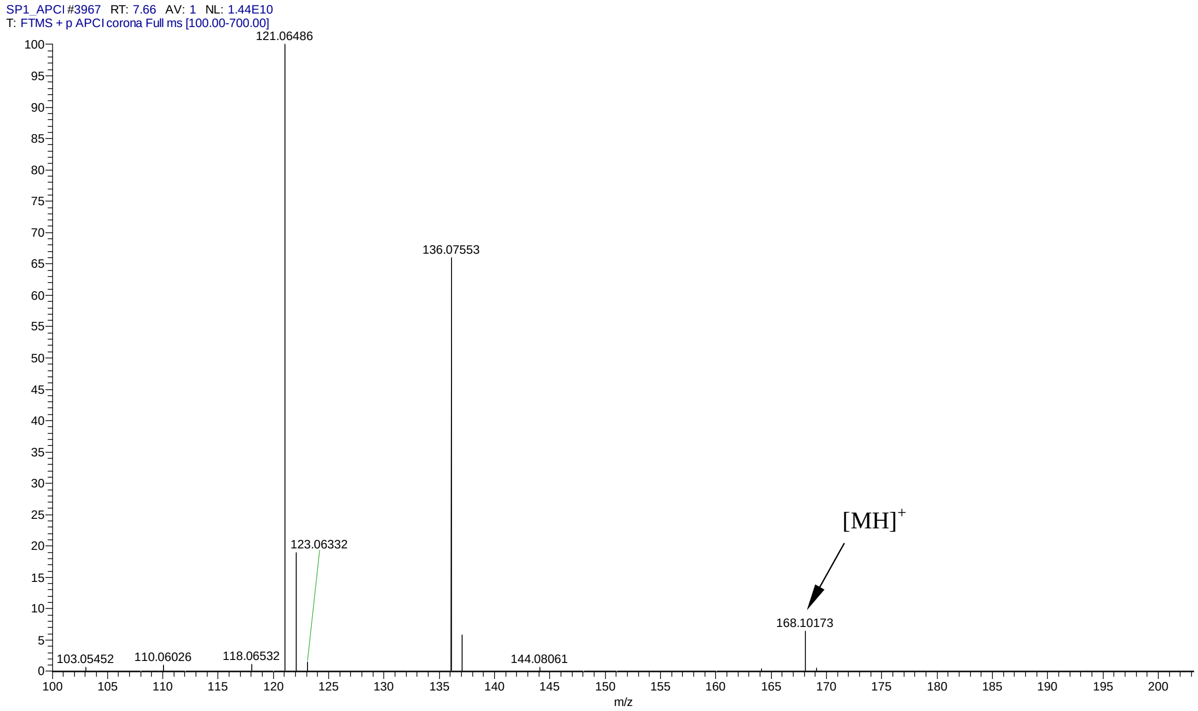

Figure S41. Biotransformation product**3a** (APCI+) full scan spectrum.

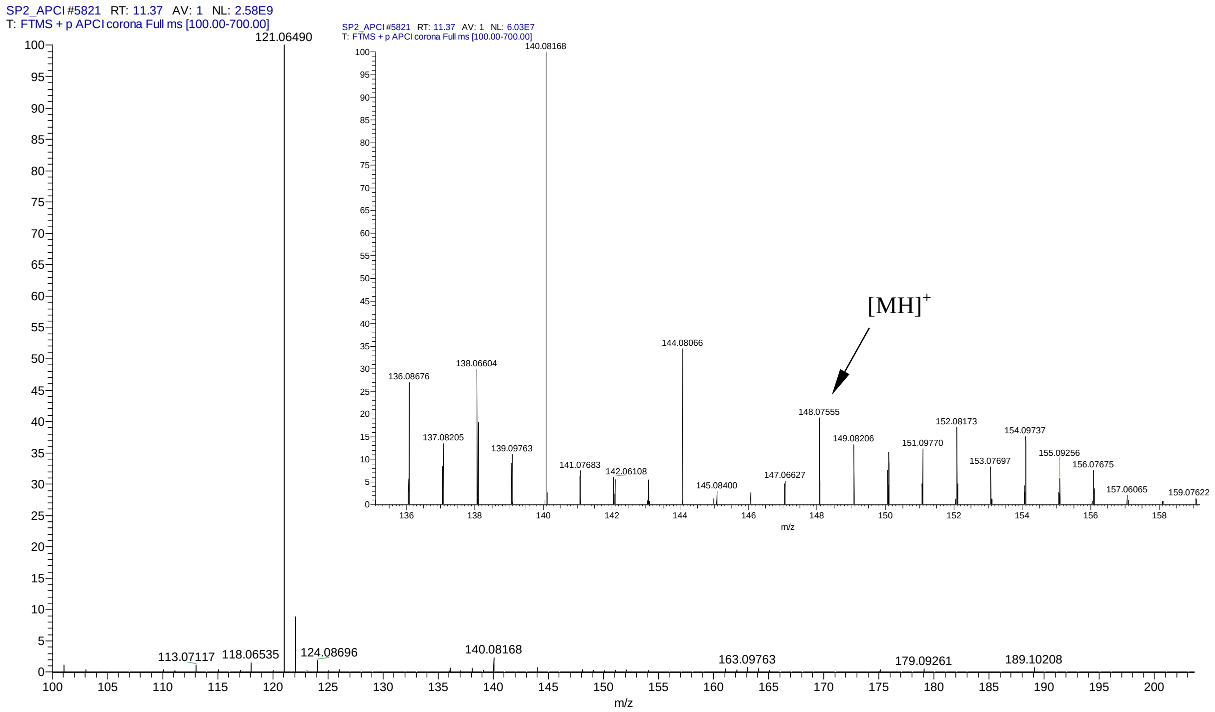

Figure S42. Biotransformation product **3b** (APCI+) full scan; inset: zoom on $[MH]^{+}$.

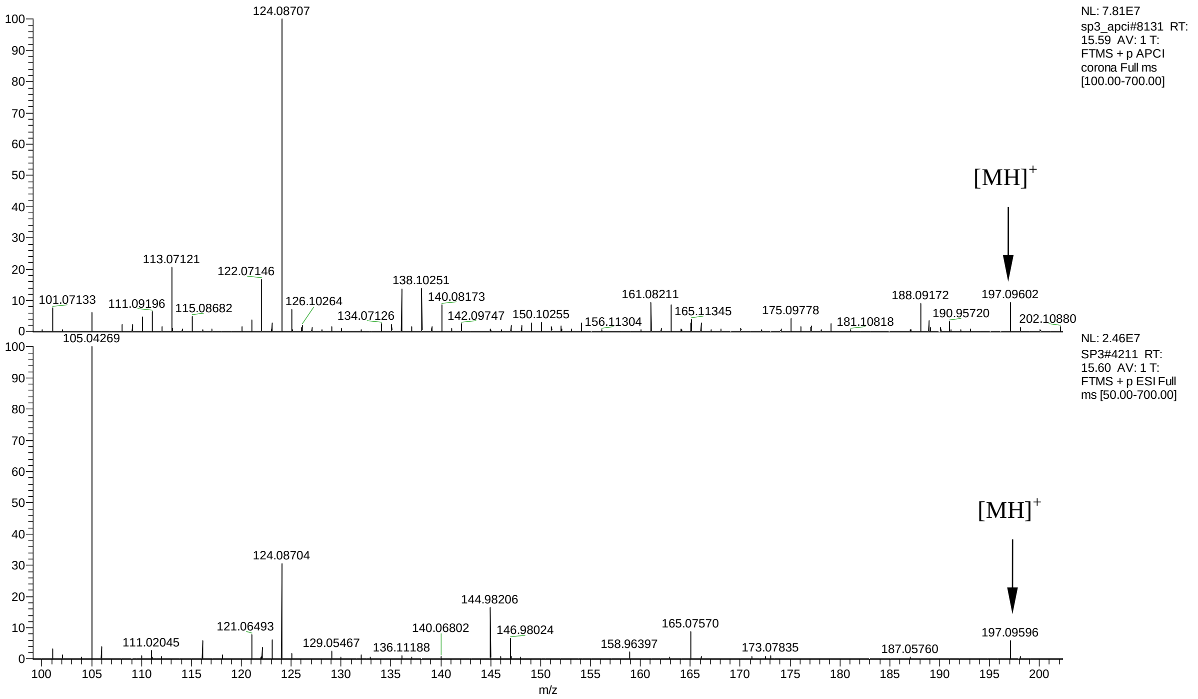

Figure S43. Biotransformation product **3c** (upper panel: APCI+; lower panel: ESI+).

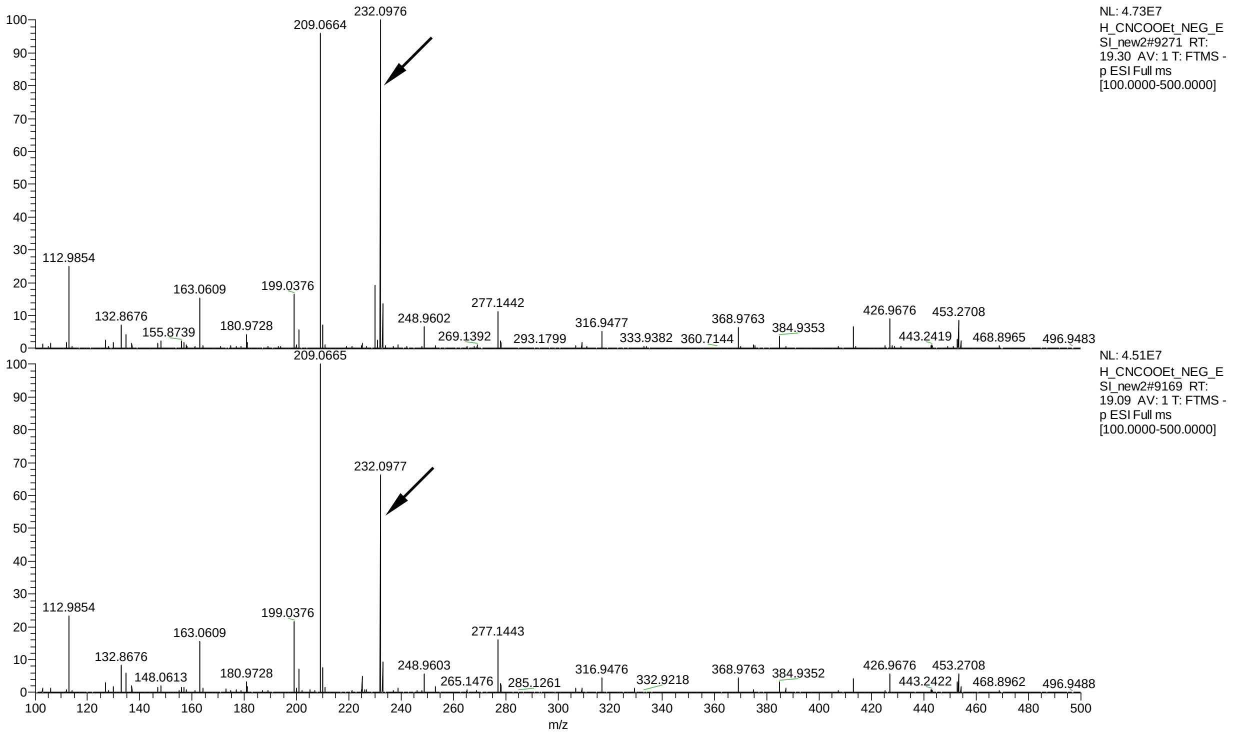

Figure S44. Reference compound **3e** (ESI –) full scan spectra of both diastereosiomers.

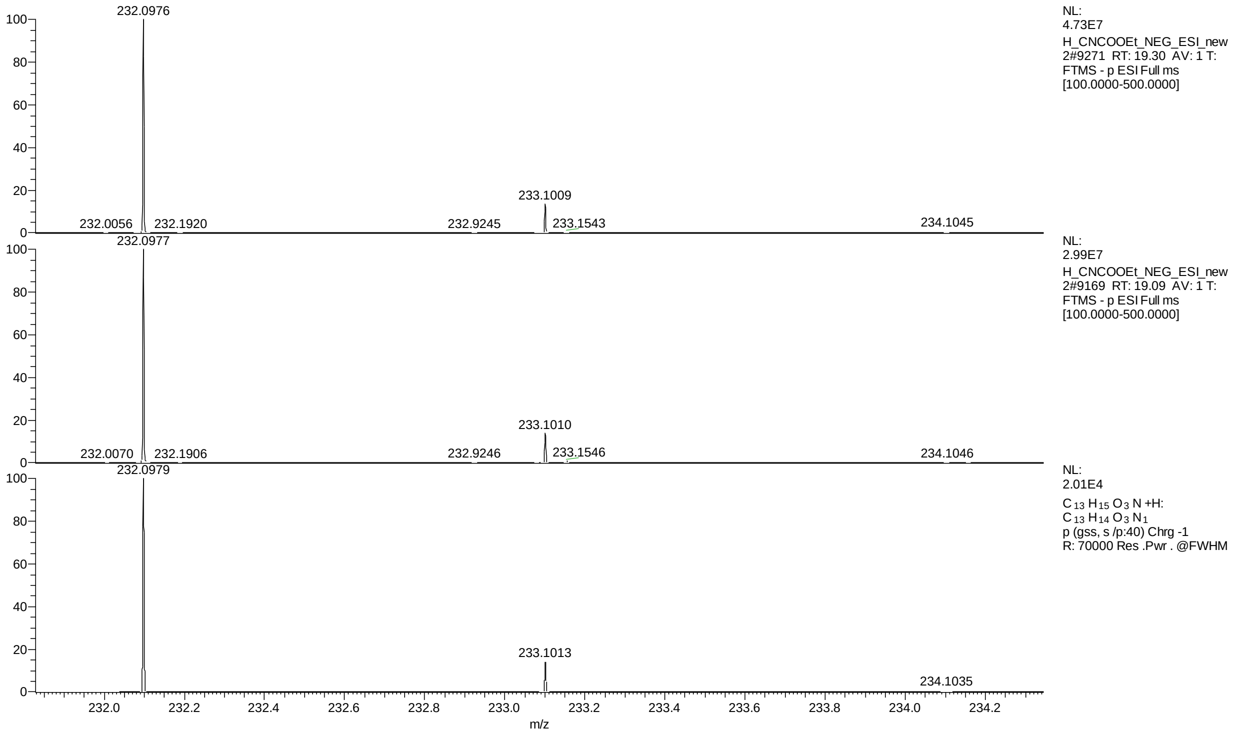

Figure S45. Reference compound **3e** (APCI –) upper panel: isomer 1 $[M]^-$ zoom; middle panel: isomer 2 $[M]^-$; lower panel: calculated mass spectrum of $[M]^-$.

8. Quantum mechanical calculations

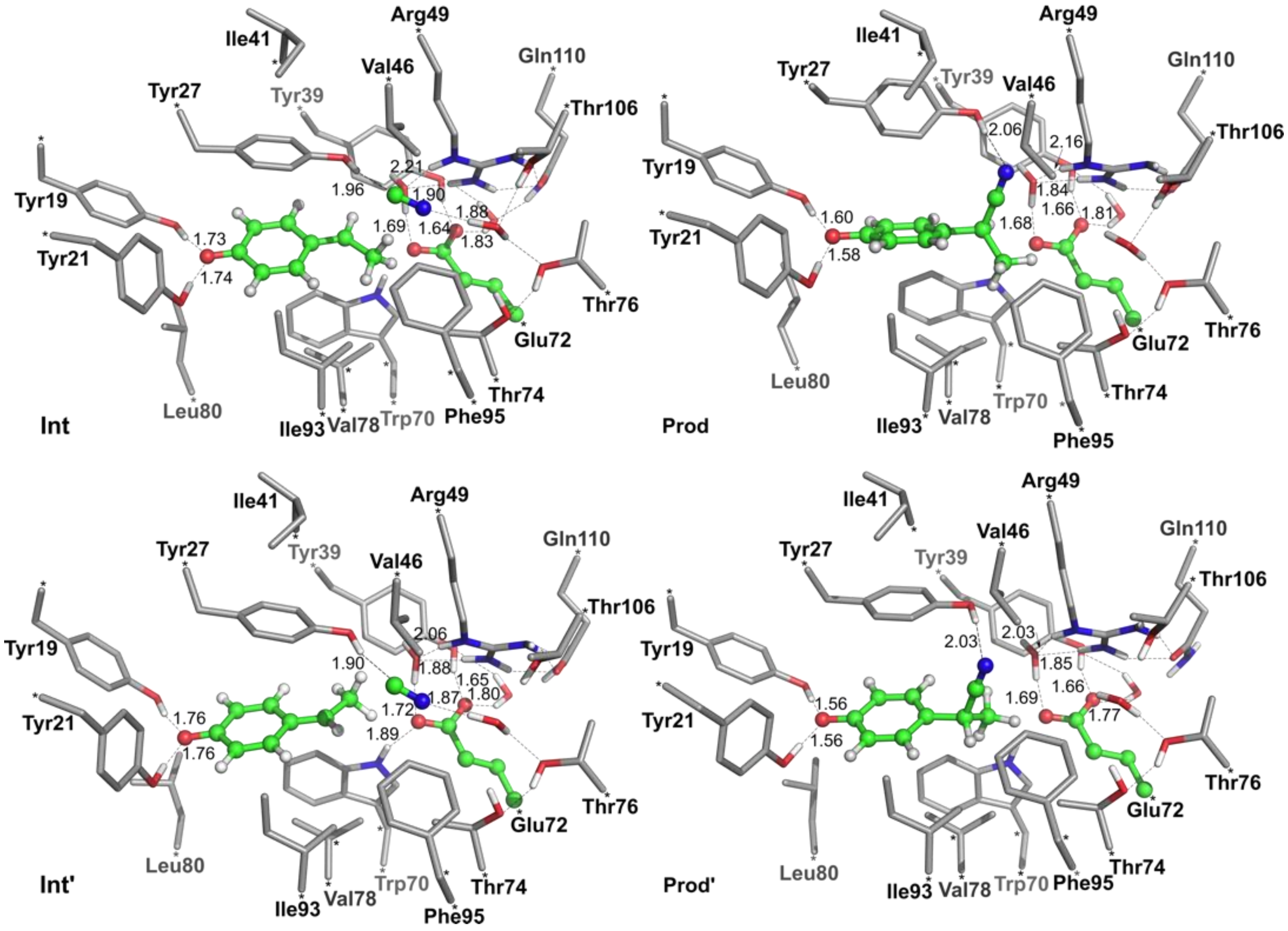

Figure S46. Optimized structures of the intermediates involved in the lowest energy pathways leading to (*S*)-product (**Int1** and **Prod**) and (*R*)-product (**Int'** and **Prod'**). For the sake of clarity, only polar hydrogen atoms and the hydrogen atoms on the substrate are shown in the figure. Distances are given in Å.

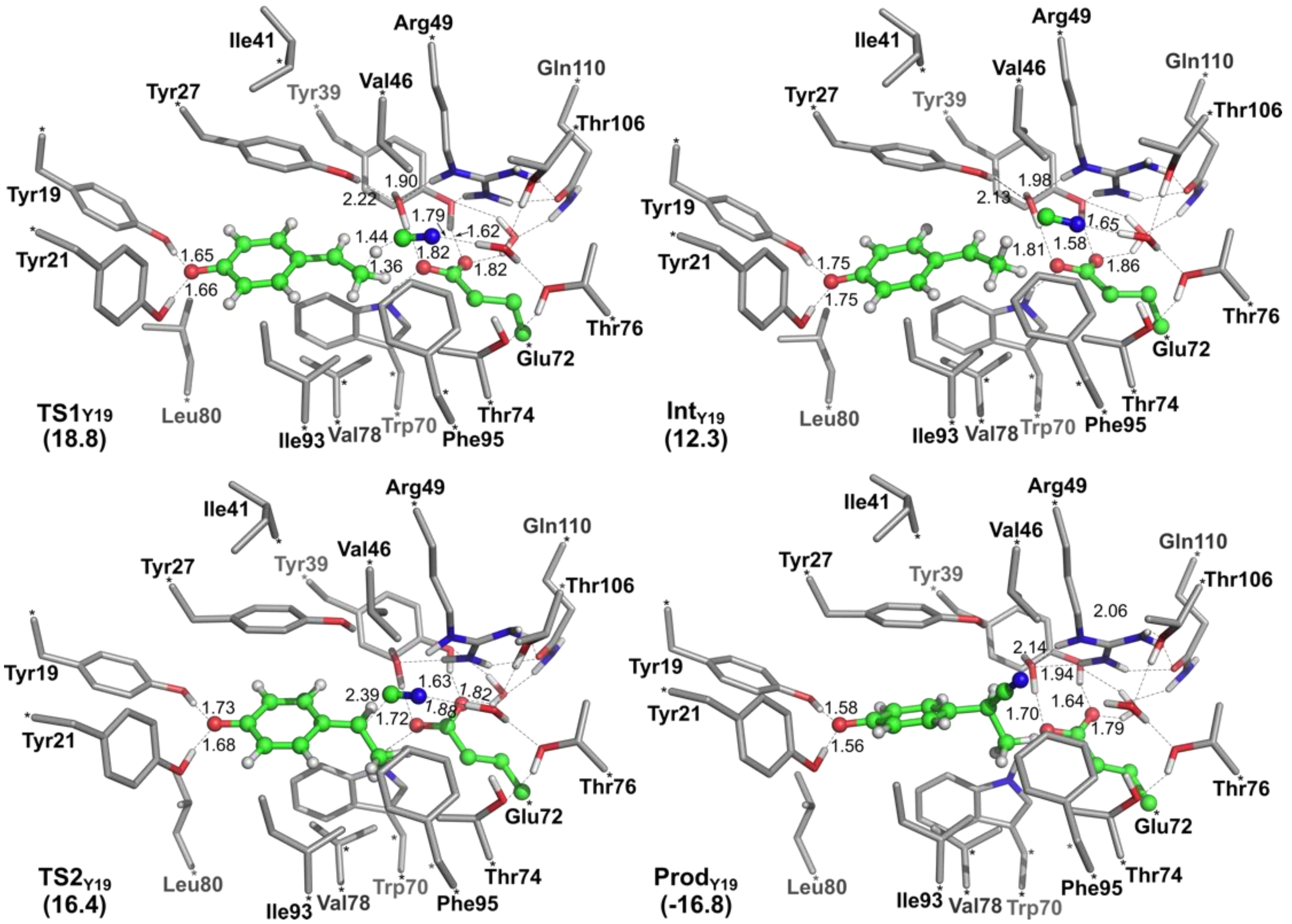

Figure S47. Optimized structures of the intermediate and transition states with Tyr19 pointing away cyanide. The energies relative to **React** are also given (in kcal/mol). For the sake of clarity, only polar hydrogen atoms and the hydrogen atoms on the substrate are shown in the figure. Distances are given in Å.

Cartesian Coordinates

React (0.0 kcal/mol)

N	-2.52932100	5.23410400	-2.43577100
N	-3.69884100	-2.11952300	-1.39663500
N	-5.99686900	-2.41278400	-1.76875700
N	-4.91727400	-0.47837200	-2.46208900
N	-7.73579200	0.85570500	-5.00023500
C	-3.01623200	9.63163800	-1.35161200
C	-3.07009400	8.90217200	-2.68927000
C	-2.56931000	7.48664300	-2.65361700
C	-3.31784200	6.36066600	-2.40278200
C	-1.21825700	7.02603700	-2.85636500
C	-1.23337100	5.60684800	-2.71012300
C	-0.00128700	7.66174800	-3.16523300
C	-0.07726700	4.82979400	-2.85838600
C	1.14485000	6.89255700	-3.31972500
C	1.10714800	5.48982000	-3.16551300
C	-0.34934300	6.98428500	2.46072300
C	-0.18853100	6.11382000	1.21437400
C	1.29226900	5.85070300	0.90888500
C	-0.97200700	4.80217900	1.34141100
C	0.99061300	4.26888300	5.95728500
C	1.10888400	2.84545100	5.40957300
C	1.73825100	1.91489100	6.46560700
C	1.89170400	2.84296800	4.08984500
C	1.84764400	0.44405100	6.05022700
C	-3.98721400	1.59946200	8.15660500
C	-3.31580500	0.25622000	8.42906900
C	-3.13542300	-0.62069000	7.20776800
C	-4.24777200	-1.09659700	6.49739800
C	-1.86038100	-0.99334900	6.76868300
C	-4.09026200	-1.91122100	5.37633100
C	-1.69482300	-1.81148000	5.64859400
C	-2.80870400	-2.26853800	4.94603300
C	12.09490200	-0.88075400	-2.91431600
C	11.56211500	0.56452000	-2.94417900
C	10.05516400	0.67391200	-2.81926800
C	9.22401500	0.63329800	-3.94859800
C	9.43254600	0.81093800	-1.57135600
C	7.83854100	0.72050000	-3.84126800
C	8.04696300	0.90039900	-1.44206200
C	7.22592600	0.85459200	-2.58246800
C	11.15136600	-1.24816900	3.63519400
C	9.87332200	-1.09293400	4.47891400
C	8.82751400	-0.22082400	3.81745300
C	8.72710300	1.14793700	4.10248700
C	7.95154800	-0.74410700	2.85739600
C	7.80419900	1.96406900	3.45365900
C	7.02106400	0.05523200	2.19774400
C	6.93942600	1.43027300	2.48369800
C	6.75856600	-2.82914800	-2.87263600
C	7.31738100	-3.47258900	-1.58816000
C	6.22942200	-4.05500800	-0.70950100
C	5.29066100	-3.20736400	-0.10737100
C	6.07235500	-5.43366500	-0.51757700
C	4.99175300	-5.95737900	0.19844800
C	4.03558700	-5.09035600	0.73393800
C	4.21074500	-3.70937700	0.61184900
C	1.49003500	-0.26848000	-7.62224100
C	1.47565100	-1.38709100	-6.56909700
C	0.54951800	-1.05265600	-5.42353300
C	-0.79355800	-1.44535200	-5.44040000
C	0.98928800	-0.26354400	-4.35320300
C	-1.67531500	-1.05684400	-4.43359300
C	0.12552700	0.13001400	-3.33403400
C	-1.21577100	-0.26245800	-3.37894800
C	2.96505600	-5.27002300	-3.10390800
C	2.88389900	-6.63833300	-3.79759300
C	1.85094200	-7.57890600	-3.13604400
C	4.27422200	-7.28271300	-3.91125200
C	2.14552600	-8.00174500	-1.69077700
C	-0.40355700	-7.55002300	2.40507800
C	0.26619700	-8.05610800	3.69072700
C	1.20496200	-6.99326200	4.27907900
C	-0.78087100	-8.50639000	4.71933000
C	-2.25659800	-6.50962600	-3.39190600
C	-2.60282900	-5.75314700	-2.10414900
C	-3.04696600	-4.31124300	-2.37468900
C	-3.36873200	-3.51601800	-1.10077900
C	-4.87135500	-1.67480900	-1.86672700
C	-7.39508100	4.51169600	-0.69377700

C	-7.24418400	3.03810800	-1.07787000
C	-6.09689400	2.33573700	-0.34435400
C	-4.68176300	2.76531800	-0.73821800
C	-9.87676700	0.99200800	1.93255400
C	-8.89627000	-0.18122200	1.97903000
C	-9.09128500	-1.14048000	0.80875200
C	-5.72977600	3.73224000	4.16892700
C	-5.09140500	2.44270400	3.66744200
C	-3.71567000	2.19675200	4.27568900
C	5.62414600	8.50837200	0.35613400
C	5.41928300	7.37854800	-0.66090000
C	5.38605200	5.95700900	-0.06328400
C	4.98201400	4.93021600	-1.13226300
C	6.71684100	5.55732100	0.59128500
C	-7.36248200	-5.88101200	0.62026700
C	-6.77295600	-4.85552500	1.57414800
C	-5.60893500	-5.41458700	2.39387600
C	-8.41009900	-3.52994900	-6.38920300
C	-7.76130200	-2.40248400	-5.58878000
C	-8.77714100	-1.35362200	-5.08742400
C	-8.03825200	-0.26770500	-4.32474400
C	0.28608700	-3.47532700	0.50202700
C	0.61146100	-2.33400100	1.14808000
C	1.57912100	-1.31979900	0.74544000
C	2.15368600	-0.47909600	1.72449400
C	3.21177200	0.36779100	1.43901100
C	3.77229600	0.43566800	0.12980100
C	3.12278300	-0.33964500	-0.87820300
C	2.07786400	-1.19509700	-0.57067800
O	5.88133100	0.93345600	-2.53382500
O	6.06260100	2.25745700	1.87906300
O	2.93025900	-5.62627100	1.35504500
O	-3.76266800	2.60966200	0.13389100
O	-4.46438400	3.20808400	-1.89890900
O	-7.53604600	0.27965400	1.91003100
O	-6.00851300	1.36653000	4.00122600
O	-6.35364200	-3.73333800	0.79156600
O	-5.41535100	2.11631700	-4.09808400
O	-2.10535600	0.08422200	-2.39309900
O	-5.86886800	-1.79495900	2.57672100
O	-7.67480300	-0.45368300	-3.15022200

O	-1.39820400	2.12134400	-0.96529700
O	4.83287700	1.15505300	-0.12737900
H	11.88641700	1.04050400	-3.87906800
H	12.03595400	1.13185200	-2.13297200
H	9.67214500	0.53992100	-4.93681500
H	10.04233000	0.85725900	-0.67105600
H	7.20523900	0.69626900	-4.72308200
H	7.59632000	1.02215300	-0.46321100
H	5.53160800	1.04321600	-1.59491600
H	13.18723100	-0.90497000	-3.0075950
H	10.13856800	-0.67260700	5.45759500
H	9.45475300	-2.08867900	4.67519300
H	7.99184700	-1.80515900	2.61767300
H	9.38820800	1.58629100	4.84852300
H	6.34982500	-0.37439000	1.46278400
H	7.73575100	3.02383800	3.67997700
H	5.57842100	1.80588400	1.11002500
H	11.61529000	-0.27370800	3.44913900
H	7.87811600	-2.70889800	-1.03433300
H	8.03680800	-4.25803400	-1.85129000
H	5.38160200	-2.13185900	-0.22599600
H	6.79603200	-6.11855400	-0.95460800
H	3.49173000	-3.02689600	1.05145700
H	4.86367400	-7.02861000	0.31893000
H	2.26695500	-4.92236900	1.45356600
H	6.07510500	-2.00865800	-2.63884700
H	2.52175000	-6.46633700	-4.82280600
H	2.49306900	-1.54581000	-6.19307900
H	4.22464900	-8.26123100	-4.40428700
H	4.73191100	-7.42227100	-2.92664800
H	1.16462800	-2.32850300	-7.03846900
H	4.95030000	-6.64781600	-4.49428000
H	-1.16077000	-2.05958600	-6.25983300
H	0.86953100	-7.08650700	-3.16784200
H	2.02938800	0.04967200	-4.30872000
H	1.75596000	-8.48080300	-3.75766800
H	-2.71901100	-1.35505100	-4.46104400
H	1.35280300	-8.65890600	-1.31581200
H	0.49488600	0.72037700	-2.50273800
H	-1.75316700	0.83764700	-1.82208100
H	2.21136600	-7.14709600	-1.01122700

H	2.16421700	-0.50895800	-8.45225500
H	3.08748800	-8.55509700	-1.61511200
H	3.36006600	-5.34999200	-2.08821900
H	0.87709400	-8.93179800	3.42584800
H	-0.30848700	-8.89428700	5.62889400
H	-1.42240900	-7.66551800	5.01099600
H	-1.42863400	-9.29321500	4.31552000
H	1.71372200	-7.36589300	5.17558100
H	0.64328800	-6.09605900	4.56912100
H	1.96880600	-6.69218300	3.55528100
H	-1.03889600	-8.32071400	1.95335500
H	-1.72964400	-5.74699800	-1.43921900
H	-3.39585200	-6.28825400	-1.56365900
H	-2.25736600	-3.78313200	-2.92455900
H	-3.93250100	-4.31358100	-3.02454500
H	-4.18588100	-3.96965500	-0.53354700
H	-2.49953100	-3.48699300	-0.43724300
H	-2.92024900	-1.47540800	-1.53299700
H	-6.84017700	-1.95452500	-2.10546100
H	-6.10129400	-3.05124200	-0.97692500
H	-5.81155100	-0.09298900	-2.75401900
H	-4.05268900	0.02169300	-2.64947000
H	-3.11973800	-6.55924000	-4.06617200
H	-8.17405300	2.50223700	-0.85012800
H	-7.09792700	2.95430300	-2.15904000
H	-6.20701200	2.45348300	0.73696800
H	-6.15270700	1.25447800	-0.52394500
H	-6.48514600	5.07129700	-0.93309900
H	-9.04275000	-0.73165300	2.92209900
H	-8.94744800	-0.61434200	-0.14125400
H	-8.37639200	-1.96717900	0.85238100
H	-10.10332100	-1.55799900	0.82256900
H	-7.29701000	0.82192600	2.69206100
H	-10.91030400	0.63481600	1.99410400
H	-4.98460200	2.49474200	2.57559900
H	-3.02683200	2.99546200	3.98334800
H	-3.28897000	1.24963700	3.93001300
H	-3.77325500	2.16500000	5.36600900
H	-5.50478900	0.53930700	4.03702300
H	-5.82141300	3.71431700	5.25979200
H	4.47567700	7.55350000	-1.19555100
H	6.21202300	7.42456300	-1.42227600
H	4.61064200	5.94749000	0.71827100
H	4.01311500	5.18556400	-1.57720500
H	4.90557300	3.92049200	-0.71677500
H	5.72250400	4.89753200	-1.94162500
H	7.52685500	5.55856500	-0.15011200
H	7.00241900	6.24374100	1.39632400
H	6.65095200	4.55141400	1.01656800
H	6.59414400	8.43585900	0.85859600
H	-7.56196500	-4.52499700	2.26820500
H	-5.19803400	-4.64397000	3.05252200
H	-5.93504800	-6.25448500	3.01747000
H	-4.80827900	-5.76557100	1.73398000
H	-6.12926300	-3.00513700	1.42673300
H	-7.73886300	-6.75271200	1.16514900
H	-7.00636300	-1.89973100	-6.20655600
H	-7.22825900	-2.81154700	-4.72341700
H	-9.33680000	-0.93513400	-5.93278800
H	-9.49494100	-1.82245100	-4.40742300
H	-9.14929200	-4.07109600	-5.78796400
H	-4.70428700	2.30589400	-4.72127100
H	-5.15507900	2.59178300	-3.26320600
H	-4.94103200	-1.56772100	2.38823000
H	-6.41063500	-1.03677700	2.25091500
H	-8.92384400	-3.14519900	-7.27764000
H	-7.66131900	-4.25389300	-6.72540500
H	-8.18866200	-5.44602500	0.04968400
H	-6.60064400	-6.22430000	-0.08860700
H	-1.93883600	-7.53534400	-3.17996300
H	-1.44277400	-6.01408500	-3.93210700
H	-9.75624600	1.55089500	0.99910100
H	-9.70869500	1.68029400	2.76812300
H	-8.22729700	4.98213500	-1.22865100
H	-7.58270100	4.62465700	0.38059700
H	4.84872800	8.48443900	1.13102200
H	5.57747500	9.49045100	-0.12819100
H	-5.12129000	4.59445100	3.88057800
H	-6.72962000	3.86238600	3.74259300
H	11.82189600	-1.37684900	-1.9771530
H	11.67010500	-1.47073400	-3.7335590
H	10.92231600	-1.69323800	2.66152500

H	11.88834100	-1.88719800	4.13658300
H	1.97808500	-4.79551800	-3.04977700
H	3.62588100	-4.59184900	-3.65442200
H	6.21187600	-3.56751200	-3.47018300
H	7.56607800	-2.41481300	-3.48395800
H	-7.00143000	1.47903400	-4.63274200
H	-7.98721300	0.93245200	-5.97317900
H	-0.98577300	-0.64052300	7.30911000
H	-0.69479800	-2.08689100	5.32579600
H	-2.68226100	-2.88814800	4.06439300
H	-4.96253500	-2.25156600	4.82703000
H	-1.03720200	-6.67902500	2.61531100
H	-5.24929700	-0.82995000	6.82818000
H	-2.33948400	0.42622400	8.89739500
H	0.34496500	-7.24854000	1.66507000
H	1.82138600	0.67661000	-7.18089100
H	-3.91369800	-0.29078500	9.17188500
H	0.48798700	-0.10700000	-8.03383800
H	-3.36166900	2.23103400	7.51864200
H	-4.16362500	2.14418700	9.09085900
H	-4.95304000	1.47503600	7.65676000
H	0.87261200	0.04940400	5.74129600
H	2.54101500	0.30697600	5.21539100
H	2.21120200	-0.17326200	6.87901600
H	1.46373100	3.55584100	3.37815500
H	2.93815100	3.12984300	4.25122200
H	1.88659600	1.86434800	3.60531200
H	0.09258800	2.47376900	5.20397200
H	1.98203900	4.69178300	6.16160800
H	0.41990000	4.29656600	6.89344800
H	0.49187000	4.93191900	5.24229800
H	1.14484400	1.98466900	7.38833000
H	2.73810000	2.29551200	6.71925900
H	0.06036700	6.48281200	3.34718000
H	-0.60800200	6.66436100	0.36093900
H	1.84288900	6.78944300	0.78267100
H	1.77204600	5.29188700	1.72178100
H	1.40802000	5.27360800	-0.01297500
H	-0.60234000	4.20204200	2.18347500
H	-2.04151800	4.97976100	1.49893300
H	-0.87198900	4.20963200	0.42856800

H	-3.38216100	10.66174200	-1.4418700
H	-2.49432300	9.47137600	-3.43181900
H	-4.10627600	8.90810100	-3.05386800
H	0.04186500	8.74197800	-3.28269200
H	2.08935200	7.37380000	-3.55813400
H	2.02124800	4.91601600	-3.28721200
H	-0.11268800	3.75357200	-2.72514400
H	-2.89664200	4.30484900	-2.24026100
H	-4.37759100	6.26925100	-2.20759200
H	0.17773700	7.93933200	2.35252200
H	-1.40364700	7.20630100	2.66425500
H	-1.99309600	9.67077100	-0.96303000
H	-3.62835200	9.11951900	-0.60163100
H	-3.45757600	1.11249400	0.87744300
H	-2.24385500	2.45407700	-0.54490100
H	-0.75202400	2.01956900	-0.25453000
H	1.78048100	-0.53543900	2.74641800
H	3.65922400	0.97846500	2.21668000
H	3.50275000	-0.27570500	-1.89388300
H	1.63618700	-1.79083400	-1.36543700
H	0.13901600	-2.15615300	2.11605100
H	-0.44971800	-4.15886800	0.91543300
H	0.72961400	-3.74787500	-0.45178400
C	-3.28294600	0.10476000	1.30253800
N	-3.17563500	-0.96150200	1.74472100

TS1 (17.2 kcal/mol)

C	-1.64259900	8.87041000	2.64379100
C	-1.83898900	8.67424800	1.14642700
C	-1.29176400	7.37820600	0.62008400
C	-2.02053900	6.26712300	0.27193700
C	0.09054800	7.02899800	0.40105800
C	0.11252200	5.68559000	-0.07735200
C	1.30979500	7.71610500	0.54753500
N	-1.19161800	5.24921400	-0.14914800
C	1.30841200	5.03043800	-0.39746600
C	2.49622000	7.06837700	0.22680300
C	2.49517400	5.73662100	-0.23849600
C	0.74931500	4.74986800	5.08222900
C	0.75033300	4.36099200	3.60125400
C	1.99842500	3.54556500	3.24626700
C	-0.54128400	3.61802700	3.23687300
N	-4.18241400	-1.52643100	-1.97331100
N	-6.53730000	-1.44861400	-1.90339700
N	-5.29030800	0.44796600	-2.35688400
N	-8.67848100	2.83991700	-3.59234600
C	1.81013600	0.83255500	7.30196800
C	1.71434900	-0.30775600	6.28752600
C	3.04791600	-0.47538200	5.53126400
C	0.51987800	-0.10619000	5.34648500
C	3.13924400	-1.72149500	4.64402300
C	-3.41849800	-1.83502700	8.81857300
C	-2.93035200	-3.25560900	8.55233200
C	-2.65123800	-3.49717700	7.08060200
C	-3.69822300	-3.47877100	6.14563100
C	-1.34665300	-3.68349300	6.60907000
C	-3.44767100	-3.61505000	4.78060100
C	-1.08971600	-3.83078500	5.24342100
C	-2.13746500	-3.78598900	4.32353100
C	11.81974700	-2.11136300	-3.63796500
C	12.02942100	-0.64168700	-3.23709300
C	10.73532700	0.05277000	-2.87400600
C	10.07996200	0.90506800	-3.77194500
C	10.12061400	-0.18008700	-1.63659900
C	8.85740100	1.49481200	-3.45865200
C	8.90004500	0.39971100	-1.30404700
C	8.24748000	1.24113100	-2.22133200
C	11.03669900	-4.66229200	2.42909700
C	9.58404700	-4.75114100	2.93090100
C	8.83573100	-3.43840600	2.83006700
C	8.84916300	-2.50933600	3.88042100
C	8.14108200	-3.08399700	1.66659100
C	8.21110500	-1.27706200	3.77475800
C	7.49228600	-1.85639100	1.54152800
C	7.52895700	-0.92866600	2.59740800
C	6.26756900	-3.30341700	-3.86232000
C	5.67915100	-1.97775100	-3.34269100
C	4.20302700	-2.04153900	-3.00014400
C	3.22292700	-1.60157800	-3.90106000
C	3.76421600	-2.52673400	-1.76041500
C	2.41005300	-2.56797700	-1.42546200
C	1.45201700	-2.10748700	-2.33489900
C	1.86612600	-1.62911300	-3.58227800
C	1.25885800	1.37669800	-7.00349800
C	0.69591700	2.80856900	-7.07214300
C	-0.64037300	2.96388800	-6.37619400
C	-1.84652300	2.74283600	-7.05683300
C	-0.71961500	3.30112800	-5.01925500
C	-3.07832800	2.83517200	-6.41427000
C	-1.94464000	3.40472000	-4.35645300
C	-3.14109000	3.15902500	-5.05156700
C	2.17063400	-5.03728500	-4.63064900
C	2.17041600	-6.57339400	-4.60897600
C	1.74714800	-7.14546100	-3.23655400
C	3.51965300	-7.13372300	-5.08518900
C	2.66425300	-6.80590200	-2.05454500
C	-1.30139200	-8.70487300	-0.01422600
C	-1.25983100	-7.43834600	0.85326300
C	-1.70002600	-6.20753300	0.04889500
C	-2.11009100	-7.60585100	2.12050000
C	-3.18008900	-5.46879000	-4.92574200
C	-3.52014300	-4.99316100	-3.51078200
C	-3.72034200	-3.47636100	-3.43447800
C	-4.01730000	-2.97755000	-2.01474600
C	-5.33576200	-0.85249800	-2.06333300
C	-6.66307400	4.40543900	1.82046400
C	-6.01337500	4.22564700	0.44833700
C	-4.67258100	3.49361400	0.52401500

C	-3.98863100	3.24697100	-0.82015700
C	-9.52555600	0.50375200	3.24654200
C	-8.60479000	-0.51555600	2.56864100
C	-9.06886700	-0.87919500	1.16204100
C	-4.97223000	1.76272800	5.96464200
C	-4.65268400	0.91231100	4.73594500
C	-3.23801300	0.34617100	4.75990800
C	6.80773400	6.20304000	3.17953000
C	7.27780200	6.27700400	1.72321800
C	6.98342800	5.01893200	0.88331700
C	7.32775700	5.25901300	-0.59410900
C	7.71274700	3.77432000	1.40883300
C	-8.02374700	-5.70457500	-0.56225100
C	-7.33547300	-4.95261100	0.57611800
C	-6.21303300	-5.77334900	1.21376000
C	-8.95382100	-0.91535900	-6.21536900
C	-8.33401600	0.00236900	-5.16577300
C	-9.37251600	0.58430000	-4.18313400
C	-8.66713200	1.54294700	-3.23774700
O	7.06071800	1.83043800	-1.95328300
O	6.93585900	0.28229900	2.53916600
O	0.10798700	-2.10085900	-2.06750500
O	-4.36001300	3.22204000	-4.45857200
O	-2.77185700	2.89014200	-0.80411100
O	-4.68290100	3.36764900	-1.87408600
O	-7.27031700	0.00189400	2.44883900
O	-5.63112000	-0.15521700	4.68599100
O	-6.82384400	-3.71321300	0.06555800
O	-6.66308400	4.72567400	-3.14628600
O	-5.60704500	-2.27069000	2.10028400
O	-8.05571100	1.11679600	-2.24162700
H	12.51673700	-0.10720700	-4.06217900
H	12.72621400	-0.60044000	-2.38932600
H	10.53501500	1.11132700	-4.73913700
H	10.60554600	-0.82861100	-0.90947000
H	8.35484200	2.15436200	-4.15951100
H	8.45253600	0.21264700	-0.33430000
H	6.65467100	1.44587100	-1.12238800
H	12.76931200	-2.59897500	-3.88938300
H	9.58710100	-5.09437300	3.97350700
H	9.05504200	-5.52182500	2.35581000
H	8.10230400	-3.78528000	0.83491100
H	9.37286700	-2.75623200	4.80241300
H	6.95530300	-1.60167900	0.63321000
H	8.22909400	-0.56219800	4.59163300
H	6.54271700	0.45479300	1.63519000
H	11.60321500	-3.92315800	3.00523500
H	5.83950400	-1.19752600	-4.09559100
H	6.24316000	-1.65765400	-2.45902900
H	3.52809500	-1.21045600	-4.86904900
H	4.49519300	-2.85891800	-1.02724200
H	1.11684300	-1.27114500	-4.28153000
H	2.10193600	-2.92702200	-0.44642200
H	-0.04673100	-2.27968300	-1.12808000
H	5.75874800	-3.62943300	-4.77601200
H	0.59609300	3.10307400	-8.12450800
H	1.42245000	3.49890400	-6.62694800
H	-1.82076600	2.48852300	-8.11464200
H	0.19413100	3.50206500	-4.46410100
H	-4.00572900	2.65517500	-6.94933200
H	-1.98042300	3.68827100	-3.30851500
H	-4.28172500	3.26944300	-3.46321200
H	2.22115200	1.30583600	-7.52222900
H	1.40811900	-6.91296300	-5.32661300
H	3.52522900	-8.23013700	-5.07699700
H	4.34440400	-6.78998300	-4.45141700
H	3.73876700	-6.80614500	-6.10755800
H	0.73338200	-6.78542100	-3.01330500
H	1.66804300	-8.23821900	-3.32504500
H	2.28338500	-7.25624600	-1.13146600
H	2.73282500	-5.72650600	-1.88988800
H	3.68042900	-7.18519100	-2.20541000
H	2.94297300	-4.61801100	-3.98047400
H	-0.21743300	-7.27987500	1.16639900
H	-2.05734000	-6.71858800	2.76023300
H	-3.16480000	-7.76552100	1.86305100
H	-1.77943900	-8.46678000	2.71274600
H	-1.66462300	-5.29270000	0.64942200
H	-2.73115000	-6.32638100	-0.30697900
H	-1.06007400	-6.05890600	-0.82843700
H	-0.95171700	-9.58254400	0.54106000
H	-2.71834800	-5.28970900	-2.82270900

H	-4.42872500	-5.50110800	-3.15869200
H	-2.81693800	-2.97070000	-3.80022600
H	-4.54114700	-3.17621300	-4.09975500
H	-4.90481800	-3.46016800	-1.59744400
H	-3.19460300	-3.22263100	-1.33845600
H	-3.34606900	-0.95043100	-2.07837900
H	-7.32933900	-0.80986300	-1.90257500
H	-6.62571200	-2.30372400	-1.35695200
H	-6.11592500	1.02580000	-2.23389300
H	-4.38266300	0.86864600	-2.54746200
H	-3.97591700	-5.20908700	-5.63349100
H	-6.68741300	3.66362800	-0.20668600
H	-5.86996900	5.20483200	-0.02672600
H	-3.96360100	4.01635800	1.17643500
H	-4.81339800	2.50263600	0.97943600
H	-6.01305400	4.96449000	2.50388900
H	-8.58345800	-1.43085800	3.18233900
H	-9.00439100	-0.00612000	0.50372400
H	-8.44761000	-1.67674800	0.74385100
H	-10.10675400	-1.22838300	1.17823800
H	-6.88853800	0.10308900	3.34712400
H	-10.53906200	0.10224000	3.35497200
H	-4.77190500	1.53401200	3.83507500
H	-2.50322900	1.15640800	4.78486700
H	-3.03597200	-0.25629800	3.86746600
H	-3.09120400	-0.28921300	5.63893600
H	-5.25346000	-0.89582400	4.18322400
H	-4.83733900	1.17601200	6.87901600
H	6.80018000	7.14092300	1.24009500
H	8.35976100	6.47574300	1.69595500
H	5.90148800	4.82523500	0.94908400
H	6.76460900	6.10643000	-1.00352700
H	7.10954700	4.37396500	-1.19955100
H	8.39602500	5.48398800	-0.71125800
H	8.80077700	3.91726800	1.37283100
H	7.43929700	3.53259600	2.44002700
H	7.46860300	2.90045700	0.80047100
H	7.32092600	5.41164800	3.73442000
H	-8.08961000	-4.73092300	1.34893900
H	-5.72867300	-5.20772400	2.01489000
H	-6.60161900	-6.70510200	1.63993300

H	-5.45311200	-6.02412800	0.46596300
H	-6.37482100	-3.24202600	0.81160900
H	-8.46953800	-6.63322300	-0.19289200
H	-7.81111500	0.82976700	-5.66131000
H	-7.57565500	-0.53936100	-4.59074000
H	-10.17268500	1.09060600	-4.73727100
H	-9.82759600	-0.21717800	-3.59345000
H	-9.46142600	-1.76814800	-5.75030200
H	-6.12932000	4.55881900	-3.93942000
H	-6.07339400	4.37447900	-2.44473200
H	-4.68778900	-2.17042400	1.76390000
H	-6.06389400	-1.40698100	2.01562500
H	-9.69321000	-0.38360800	-6.82561600
H	-8.18965100	-1.31285700	-6.89080100
H	-8.81414000	-5.09302800	-1.00807900
H	-7.30048600	-5.95479000	-1.34608500
H	-3.04355700	-6.55465200	-4.96208100
H	-2.25471700	-5.00501900	-5.28464000
H	-9.57570500	1.42271500	2.65329600
H	-9.15566100	0.76004100	4.24532600
H	-7.61069700	4.94932800	1.74444900
H	-6.87458600	3.43303600	2.28146900
H	5.73185900	6.00012700	3.23528400
H	6.99386600	7.14691800	3.70453600
H	-4.31185000	2.63411500	6.00493700
H	-6.00748900	2.11574800	5.93149600
H	11.35577600	-2.67525900	-2.82235700
H	11.15773100	-2.18683100	-4.50708700
H	11.06692800	-4.35267200	1.37929500
H	11.54951900	-5.62763000	2.51477100
H	0.56965500	0.66169900	-7.46535700
H	1.41174200	1.06603100	-5.96463300
H	1.20874300	-4.63110500	-4.30178500
H	2.36042500	-4.66289600	-5.64290500
H	6.15750700	-4.10161000	-3.12014500
H	7.33414400	-3.19348600	-4.08463200
H	-2.32498400	-8.91135800	-0.35153100
H	-0.67318100	-8.59918200	-0.90558700
H	-7.98036100	3.51315900	-3.22952400
H	-9.13235900	3.09745600	-4.45541000
H	-0.52139900	-3.70367100	7.31732300

H	-0.06783500	-3.96558100	4.89982000
H	-1.94408200	-3.85456000	3.25820700
H	-4.26656900	-3.56414000	4.06829900
H	-4.72063200	-3.33995400	6.49033300
H	-2.02153500	-3.45139300	9.13302900
H	-3.68500300	-3.97005000	8.90854700
H	-2.66991600	-1.09974800	8.50530900
H	-3.62076500	-1.67579300	9.88398400
H	-4.34036800	-1.62033700	8.26891400
H	2.96208000	-2.63401000	5.22673600
H	2.40750700	-1.70107000	3.83019100
H	4.13117700	-1.80551500	4.18939000
H	-0.40809700	0.01406700	5.91496200
H	0.65446700	0.79369500	4.73470800
H	0.37619800	-0.95602900	4.67311500
H	1.53803500	-1.23934000	6.84920400
H	1.99938400	1.78984900	6.80092100
H	2.62662100	0.66755900	8.01423200
H	0.88178600	0.93849700	7.87495500
H	3.86276900	-0.50720600	6.26680200
H	3.22964200	0.42206000	4.92328700
H	0.70738700	3.85891300	5.72227700
H	0.77810700	5.28467500	3.00746500
H	2.91376200	4.10268700	3.47504900
H	2.03270400	2.60851400	3.81605600
H	2.02227100	3.29216700	2.18351600
H	-0.62948600	2.68538700	3.80935700
H	-1.42380300	4.22955600	3.45838100
H	-0.57128000	3.36253500	2.17468400
H	-2.04216000	9.83576400	2.97706100
H	-1.37306800	9.51432900	0.61153000
H	-2.90970900	8.73044600	0.91158200
H	1.32383600	8.74262800	0.90559300
H	3.44256900	7.59041400	0.33746300
H	3.43715500	5.25139100	-0.47655900
H	1.31370700	4.00297900	-0.74827900
H	-1.52576100	4.31917000	-0.39681200
H	-3.09286100	6.12869400	0.28025000
H	1.65385900	5.30708700	5.35173900
H	-0.11506300	5.37625200	5.33270600
H	-0.58213200	8.83516800	2.91401500

H	-2.14742900	8.08103700	3.21078100
H	-1.12232300	-0.61925600	0.67315500
O	-2.45202900	0.82125800	-2.45645900
H	-2.05220100	1.13778500	-3.27842600
H	-2.47225900	1.61582600	-1.85641600
C	-0.66791200	0.73069800	0.82424400
C	0.30724300	0.93765400	-0.15479500
C	1.71435200	0.84850600	-0.04819900
C	2.39349500	0.51943500	1.16293400
C	3.76173100	0.48919900	1.23550400
C	4.57551200	0.81124900	0.09417200
C	3.89511200	1.14846200	-1.12241500
C	2.52461000	1.14831400	-1.18317800
O	5.86393900	0.79414500	0.16620600
H	1.81096700	0.28835100	2.04999300
H	4.26910600	0.24314300	2.16302500
H	4.49701900	1.38839900	-1.99252900
H	2.02886900	1.38707700	-2.12134700
H	-0.07163700	1.15538900	-1.15369500
H	-0.36632700	0.70978900	1.87047300
H	-1.62982600	1.20587500	0.63715200
C	-1.88283400	-1.71954400	0.81283100
N	-2.88805600	-2.17994100	1.19706900

Int (4.1 kcal/mol)

C	-1.69989800	9.01502600	2.17319200
C	-1.91236100	8.73651700	0.69116100
C	-1.34361500	7.42805500	0.22263800
C	-2.05670500	6.29132600	-0.06948600
C	0.04236400	7.09165100	0.00644200
C	0.08113600	5.72864700	-0.41378600
C	1.25215300	7.80259400	0.10999300
N	-1.21546700	5.26938700	-0.45284900
C	1.28396000	5.07985700	-0.72059900
C	2.44549500	7.16044900	-0.19669500
C	2.46116800	5.81064000	-0.60618200
C	0.77125900	5.05864000	4.79755000
C	0.75867600	4.59115500	3.33968700
C	2.02775500	3.80166600	3.00188400
C	-0.51488100	3.79686900	3.02903900
N	-4.28838500	-1.72477300	-1.77854700
N	-6.63114300	-1.53640700	-1.75031600
N	-5.29012400	0.30308900	-2.18924600
N	-8.64907700	2.65380200	-3.74370600
C	1.90631100	1.27846700	7.20992900
C	1.81166300	0.08437400	6.25928200
C	3.16590200	-0.15607100	5.56099200
C	0.65759400	0.26142300	5.26563800
C	3.26390900	-1.45459200	4.75346500
C	-3.27071600	-1.36270000	8.93497200
C	-2.76914100	-2.79000100	8.73903700
C	-2.46366500	-3.07450000	7.27836400
C	-3.49763200	-3.10409300	6.32893100
C	-1.14866300	-3.23457600	6.82587700
C	-3.22483200	-3.25376200	4.96905900
C	-0.87025300	-3.39573800	5.46540000
C	-1.90464200	-3.39108300	4.52855800
C	11.81439500	-2.13539200	-3.68582700
C	11.96223900	-0.64565700	-3.33256600
C	10.63674300	0.00991200	-3.01299400
C	9.92346200	0.73044000	-3.98005400
C	10.05069100	-0.13449700	-1.74910800
C	8.67188200	1.27685700	-3.70719800
C	8.80070900	0.40465900	-1.45622800
C	8.09388500	1.11007300	-2.44176300

C	11.13707600	-4.36549300	2.51881400
C	9.69477900	-4.47111300	3.04704400
C	8.90688900	-3.18471000	2.90809800
C	8.90526500	-2.21748000	3.92352200
C	8.18060600	-2.89867000	1.74547300
C	8.22022600	-1.01359800	3.78586800
C	7.48463100	-1.70026000	1.58896000
C	7.50478100	-0.73789800	2.61106600
C	6.27448200	-3.40039500	-3.77380300
C	5.73803200	-2.55587900	-2.59788400
C	4.22709300	-2.52988000	-2.41220400
C	3.36954800	-2.00559100	-3.39405100
C	3.62827800	-2.96271100	-1.21979200
C	2.24987400	-2.87445200	-0.99775500
C	1.40966200	-2.33616000	-1.98500300
C	1.99527800	-1.91131700	-3.19335800
C	1.17165200	1.04730500	-7.09581800
C	0.59699000	2.46879700	-7.23765900
C	-0.72895800	2.65208800	-6.52953800
C	-1.94424200	2.39351300	-7.17989200
C	-0.78858300	3.05069800	-5.18850100
C	-3.16599200	2.50899700	-6.52242400
C	-2.00312000	3.17798200	-4.51122600
C	-3.20862800	2.89459700	-5.17520900
C	2.18949100	-5.21943400	-4.39504100
C	2.05605800	-6.74789600	-4.46825600
C	1.58817500	-7.36339900	-3.12937400
C	3.35000300	-7.39390000	-4.98798600
C	2.53139000	-7.17057600	-1.93490100
C	-1.18097400	-8.67265800	0.45605800
C	-1.17918600	-7.30989000	1.16042900
C	-1.73285400	-6.22096600	0.23432700
C	-1.95766300	-7.36757300	2.48206300
C	-3.15892700	-5.72649200	-4.59692300
C	-3.67850600	-5.21457000	-3.25352300
C	-3.68942100	-3.68516000	-3.17036000
C	-4.19969300	-3.18400600	-1.81572800
C	-5.40079300	-0.99282100	-1.89392300
C	-6.67628600	4.45586400	1.65550100
C	-6.01739400	4.23717400	0.29355000
C	-4.67544200	3.51023900	0.39872800

C	-3.98872500	3.22048200	-0.93498700
C	-9.47388600	0.60424000	3.32555300
C	-8.51949300	-0.41714200	2.68987400
C	-9.00189800	-0.88280100	1.31979800
C	-4.90272800	2.05870400	5.91288700
C	-4.53522600	1.17560900	4.71950700
C	-3.09883700	0.66936100	4.78385900
C	6.78782900	6.47598800	2.74116700
C	7.19411400	6.41143900	1.26496100
C	6.87670300	5.07877800	0.55900500
C	7.16242900	5.18348300	-0.94677900
C	7.63687100	3.89225000	1.16869000
C	-7.94516200	-5.78239800	-0.16450400
C	-7.19547500	-4.97825000	0.90018600
C	-5.97877400	-5.73229800	1.43841100
C	-9.00232500	-1.31440500	-6.05338900
C	-8.35553700	-0.34185200	-5.07013400
C	-9.38220300	0.37203600	-4.16586800
C	-8.65905700	1.38653200	-3.29388200
O	6.86940300	1.65243000	-2.21833700
O	6.85927000	0.45099600	2.52411100
O	0.07261500	-2.18857200	-1.85516700
O	-4.41790300	2.97979600	-4.56602800
O	-2.76333700	2.88718200	-0.90829600
O	-4.68630400	3.28301800	-1.98985600
O	-7.20913200	0.13651700	2.50742100
O	-5.46571400	0.06807500	4.69838000
O	-6.79947100	-3.72112900	0.33357100
O	-6.65652400	4.58476800	-3.36126700
O	-5.28192600	-2.16321000	2.11627200
O	-8.06090200	1.02762900	-2.26459400
H	12.44200000	-0.12158200	-4.16860000
H	12.64164600	-0.54749400	-2.47601200
H	10.35559100	0.86581600	-4.96969200
H	10.57947000	-0.68111100	-0.97099400
H	8.12382000	1.83350600	-4.46093400
H	8.37364400	0.28405000	-0.46617900
H	6.51757300	1.36647800	-1.33899500
H	12.78539100	-2.59245800	-3.91013400
H	9.72221500	-4.77214000	4.10202000
H	9.17759400	-5.27719300	2.51159100
H	8.15109800	-3.63119600	0.94123000
H	9.45290000	-2.41133500	4.84393700
H	6.92002500	-1.50615800	0.68209700
H	8.22565000	-0.26929800	4.57600700
H	6.46003100	0.57457400	1.62841200
H	11.69441600	-3.59144900	3.05682500
H	6.10086900	-1.52667200	-2.72127800
H	6.19482000	-2.92020100	-1.66969100
H	3.78632000	-1.65023700	-4.33423800
H	4.25675200	-3.36641100	-0.42855800
H	1.34591400	-1.50217100	-3.96214100
H	1.82311300	-3.20914200	-0.05656500
H	-0.30212800	-2.42845600	-0.96084200
H	5.87117300	-3.05482000	-4.73144300
H	0.47768500	2.70165300	-8.30331900
H	1.32470100	3.18929800	-6.84480900
H	-1.93370100	2.09019500	-8.22491000
H	0.13303700	3.28190000	-4.65846400
H	-4.10070300	2.29915900	-7.03326300
H	-2.02512100	3.50944700	-3.47718400
H	-4.32269900	3.07268900	-3.57530200
H	2.12785500	0.95289400	-7.62201100
H	1.26581200	-6.97588400	-5.19972400
H	3.25804400	-8.48484900	-5.05064500
H	4.20242800	-7.16802800	-4.33762700
H	3.59802000	-7.02121400	-5.98814700
H	0.60886500	-6.93448700	-2.87834200
H	1.41893400	-8.43882300	-3.28184500
H	2.12015500	-7.65162100	-1.04085400
H	2.67697900	-6.11231500	-1.69905900
H	3.51682400	-7.61237200	-2.11820700
H	2.98636500	-4.91048200	-3.71358100
H	-0.13573500	-7.05394100	1.39617700
H	-1.93238000	-6.40509200	3.00328700
H	-3.01072800	-7.61884600	2.30176600
H	-1.54629500	-8.12763300	3.15667300
H	-1.71831000	-5.23132500	0.69818000
H	-2.77210500	-6.44564700	-0.03994500
H	-1.15028500	-6.15906600	-0.69226300
H	-0.75867200	-9.45834300	1.09370800
H	-3.06106800	-5.61892200	-2.44233500

H	-4.69579900	-5.59473400	-3.08332900
H	-2.67450400	-3.29847300	-3.33281500
H	-4.31949200	-3.27547000	-3.97180100
H	-5.17630200	-3.60980300	-1.58209800
H	-3.52387400	-3.48405200	-1.01280700
H	-3.41467500	-1.20432500	-1.81904300
H	-7.39640300	-0.86678300	-1.78860500
H	-6.75105900	-2.35575600	-1.15664800
H	-6.10112200	0.91023300	-2.13580800
H	-4.36855500	0.66992100	-2.42588100
H	-3.77282900	-5.35750400	-5.42705000
H	-6.68559500	3.65373700	-0.34834100
H	-5.87285600	5.20193200	-0.21030200
H	-3.96967700	4.05537000	1.03633000
H	-4.81845000	2.53392100	0.88412700
H	-6.03236100	5.03693700	2.32638600
H	-8.45458000	-1.29272400	3.35630800
H	-8.97422400	-0.05045300	0.60790000
H	-8.36740400	-1.68925000	0.94129800
H	-10.03012200	-1.25582700	1.37874900
H	-6.79068400	0.24824800	3.38573400
H	-10.46683600	0.16716900	3.47866000
H	-4.66628500	1.75991200	3.79524000
H	-2.39780700	1.50950000	4.78242400
H	-2.86111600	0.03587700	3.92224100
H	-2.93925900	0.07824500	5.69096400
H	-5.06899000	-0.65685900	4.18674300
H	-4.75991000	1.51027900	6.84953400
H	6.68811100	7.22210100	0.72259700
H	8.27169900	6.61269500	1.17181500
H	5.79954900	4.88802100	0.68439100
H	6.58017100	5.98961800	-1.40885000
H	6.92477900	4.24877900	-1.46438400
H	8.22403900	5.39805100	-1.12579200
H	8.72130500	4.04512300	1.09386700
H	7.39162700	3.73492900	2.22314500
H	7.39390400	2.96604200	0.64132000
H	7.33673600	5.74981700	3.34873200
H	-7.88762600	-4.78612000	1.73679200
H	-5.44578300	-5.13008900	2.17980600
H	-6.28054300	-6.67242000	1.91381700
H	-5.28194900	-5.96214600	0.62565000
H	-6.24628400	-3.23914600	0.99747700
H	-8.31182000	-6.72350300	0.25682400
H	-7.78162600	0.41317000	-5.62180400
H	-7.63798900	-0.87016300	-4.43377500
H	-10.15193400	0.85614100	-0.7798510
H	-9.88096800	-0.35304000	-3.51541800
H	-9.56420000	-2.09645500	-5.53015600
H	-6.10873600	4.35893000	-4.13040000
H	-6.08471700	4.27638000	-2.62718700
H	-4.36257500	-2.13592700	1.74309100
H	-5.71573100	-1.30506300	1.96094600
H	-9.69927600	-0.79994100	-6.72530400
H	-8.24665200	-1.80794500	-6.67272700
H	-8.80021800	-5.21600700	-0.54632800
H	-7.28243200	-6.01354700	-1.00549800
H	-3.16418200	-6.82093300	-4.64022400
H	-2.13070900	-5.39230400	-4.77407200
H	-9.57453800	1.48227400	2.67887400
H	-9.09737800	0.93759900	4.29890000
H	-7.62471200	4.99464500	1.55690000
H	-6.88826700	3.49641700	2.14268700
H	5.71846000	6.26824400	2.86416000
H	6.98221500	7.46921200	3.16103600
H	-4.27376200	2.95383200	5.92950600
H	-5.94922600	2.37300200	5.85364500
H	11.36273900	-2.68922800	-2.8564680
H	11.16668700	-2.26606600	-4.5590290
H	11.14412400	-4.09735200	1.45726200
H	11.67370700	-5.31431800	2.63406300
H	0.48190200	0.30247700	-7.50635600
H	1.33922800	0.79615700	-6.04329400
H	1.26335000	-4.75261200	-4.04619100
H	2.42296300	-4.80029400	-5.38032900
H	6.00103200	-4.45500300	-3.66125500
H	7.36571200	-3.33052000	-3.82628700
H	-2.20367600	-8.97303800	0.19411700
H	-0.59762900	-8.64366200	-0.47109300
H	-7.95841400	3.34896200	-3.41107200
H	-9.09563200	2.85685200	-4.62462900
H	-0.33217200	-3.22138300	7.54489800

H	0.15947700	-3.50989900	5.13760500
H	-1.69876700	-3.46245100	3.46487100
H	-4.03389000	-3.24058900	4.24361000
H	-4.52827300	-2.98817100	6.65767300
H	-1.86917100	-2.95672400	9.34222200
H	-3.52634400	-3.49537300	9.10725000
H	-2.51889000	-0.63698000	8.60728100
H	-3.49926100	-1.15806100	9.98757800
H	-4.17983700	-1.17855000	8.35407800
H	3.05681100	-2.32732100	5.38459900
H	2.55715400	-1.47738200	3.91791000
H	4.26788900	-1.57916200	4.33547900
H	-0.28694200	0.43219500	5.79126400
H	0.83652500	1.12542100	4.61416500
H	0.51768600	-0.62030200	4.63403800
H	1.59087400	-0.80875700	6.86499200
H	2.13783700	2.20032700	6.66197000
H	2.69433500	1.13399900	7.95790800
H	0.96343900	1.44143600	7.74436800
H	3.95527500	-0.15749300	6.32459100
H	3.38597900	0.70126500	4.90844900
H	0.77168800	4.20382400	5.48626500
H	0.74994400	5.48512500	2.70108100
H	2.92666700	4.39881600	3.19151000
H	2.10233000	2.89199500	3.61060700
H	2.04449000	3.50732500	1.94924600
H	-0.56271800	2.87804600	3.62689500
H	-1.41117200	4.38670700	3.25255800
H	-0.56024800	3.51718900	1.97335900
H	-2.11713600	9.98731300	2.46104700
H	-1.47405700	9.55832700	0.10679400
H	-2.98705700	8.75598600	0.46977200
H	1.25406200	8.84360200	0.42352000
H	3.38423800	7.70191300	-0.11995600
H	3.40879500	5.33389400	-0.83945600
H	1.29836400	4.04005100	-1.03410000
H	-1.54351900	4.32635600	-0.65940300
H	-3.12640000	6.13586700	-0.04534600
H	1.66089700	5.65969500	5.01737800
H	-0.10931300	5.66867700	5.03022000
H	-0.63528400	9.01814700	2.42921600

H	-2.17877200	8.24585100	2.78837200
H	-1.35232800	-0.21698900	0.82340200
O	-2.47366000	0.69783200	-2.42162500
H	-2.12227200	0.94409900	-3.28863800
H	-2.48287100	1.54231400	-1.89774000
C	-0.81446200	0.74007800	0.87975100
C	0.19472500	0.78789400	-0.20002700
C	1.56177200	0.79166100	-0.09020000
C	2.26431100	0.65811200	1.16831500
C	3.62031800	0.66335100	1.22916500
C	4.42376100	0.83763500	0.03151500
C	3.72825300	0.97916300	-1.23147800
C	2.37221800	0.93385800	-1.28173900
O	5.69056500	0.86335700	0.09453300
H	1.68769800	0.53502500	2.07805800
H	4.14982400	0.55071900	2.16981300
H	4.32978700	1.09627100	-2.12617700
H	1.85916200	1.01171400	-2.23555700
H	-0.22282600	0.81199300	-1.20551800
H	-0.40112600	0.84837500	1.88279900
H	-1.56356100	1.52140300	0.69852100
C	-1.62451700	-2.54526300	0.48211700
N	-2.61357700	-2.39530200	1.10433800

TS2 (12.0 kcal/mol)

C	-1.99212100	9.16507800	1.45123700
C	-2.20865800	8.76238900	-0.00142900
C	-1.60619200	7.43753700	-0.37585600
C	-2.29040800	6.27061900	-0.61495400
C	-0.20900900	7.11763000	-0.54354400
C	-0.13363800	5.73345500	-0.87917200
C	0.98429900	7.85776900	-0.45402400
N	-1.42076100	5.24603300	-0.92410100
C	1.08819400	5.08800900	-1.10879000
C	2.19688600	7.22024000	-0.68629500
C	2.24829100	5.84732500	-1.00691900
C	0.68083300	5.53718400	4.33900000
C	0.67261700	4.95652700	2.92256200
C	1.78596400	3.92585100	2.71752700
C	-0.71535200	4.39477700	2.59498600
N	-4.13877100	-1.90781600	-1.67844700
N	-6.49211700	-1.88961100	-1.59863000
N	-5.30497100	-0.02529100	-2.28978000
N	-8.76959400	2.04823100	-3.81099500
C	2.00895300	2.01036300	7.02155200
C	1.95649600	0.74239500	6.16817500
C	3.27080500	0.54702900	5.38581400
C	0.71074900	0.71896700	5.27670100
C	3.48425000	-0.86815800	4.83818000
C	-3.02662500	-0.70863200	9.02263400
C	-2.46452600	-2.12386900	8.93098400
C	-2.21079100	-2.52450800	7.48700600
C	-3.28438700	-2.70114300	6.59917800
C	-0.91101400	-2.65033200	6.98239700
C	-3.06645800	-2.95874000	5.24539800
C	-0.68620000	-2.91902700	5.62909500
C	-1.76198700	-3.05903200	4.75131200
C	11.93531400	-1.81614100	-3.71935800
C	11.81587800	-0.37438300	-3.18868300
C	10.39197400	0.07337300	-2.92160600
C	9.59925500	0.62738200	-3.93808800
C	9.80887800	-0.05786900	-1.65475000
C	8.28479600	1.02298600	-3.70729900
C	8.49530400	0.33745400	-1.40186000
C	7.71184900	0.87842900	-2.43301400

C	11.42693800	-3.58002500	2.64930400
C	10.01114700	-3.87318400	3.17971700
C	9.03832100	-2.72945600	2.97775100
C	8.93264900	-1.69068200	3.91466400
C	8.23106700	-2.64916200	1.83614500
C	8.07287000	-0.61441000	3.71952800
C	7.35807600	-1.58200500	1.62450100
C	7.27636100	-0.54067900	2.56457900
C	6.45613900	-3.32230000	-3.62745500
C	5.81900800	-2.40778000	-2.56584000
C	4.30576500	-2.46754900	-2.47196600
C	3.49307600	-2.03728600	-3.53326900
C	3.66057200	-2.88786300	-1.30188400
C	2.26927800	-2.86539000	-1.17706600
C	1.48216300	-2.40239400	-2.23597800
C	2.10518300	-2.00839400	-3.42739400
C	1.12466500	0.62662000	-7.21049900
C	0.49136000	2.01020200	-7.44570500
C	-0.83355000	2.19069200	-6.73493600
C	-2.04582900	1.85906700	-7.35647200
C	-0.89316400	2.66279400	-5.41762100
C	-3.26480700	1.97370400	-6.69293000
C	-2.10490200	2.79104900	-4.73609800
C	-3.30671700	2.43306000	-5.36955600
C	2.44886000	-5.35929400	-4.04530200
C	2.52792600	-6.89352000	-4.06653100
C	2.01843000	-7.53056000	-2.75345800
C	3.94141200	-7.37042200	-4.43576800
C	2.80793800	-7.17828500	-1.48608200
C	-0.71098800	-8.56374400	1.10878700
C	-0.70434100	-7.16804500	1.75061400
C	-1.18970400	-6.10389000	0.75684000
C	-1.53915900	-7.14876200	3.03883700
C	-2.87386500	-6.11111700	-4.12873800
C	-3.31308800	-5.48889200	-2.80203900
C	-3.46625300	-3.96699400	-2.88618600
C	-3.92658800	-3.34950000	-1.56042600
C	-5.30938900	-1.28133800	-1.84164000
C	-6.76756900	4.36867000	1.36295000
C	-6.12812500	4.03281800	0.01575200
C	-4.73597400	3.41590500	0.16072200

C	-4.06491000	3.01090100	-1.15178000
C	-9.37328700	0.54262500	3.36857000
C	-8.39327000	-0.51167700	2.82966800
C	-8.85936600	-1.10760900	1.50515300
C	-4.84208900	2.39157000	5.76757900
C	-4.40968700	1.40572100	4.68490200
C	-2.96468200	0.95476400	4.85617100
C	6.60540500	7.04740600	2.09069500
C	7.18674600	6.47578500	0.79272400
C	6.54478800	5.15854000	0.31127300
C	7.07564600	4.77922700	-1.07926000
C	6.75324000	4.00321300	1.30080000
C	-7.60281000	-6.02595500	0.36425700
C	-6.84033300	-5.11802700	1.33074200
C	-5.58216800	-5.78975600	1.88243700
C	-8.92260200	-2.08338700	-5.83847600
C	-8.33438000	-0.98503900	-4.95739000
C	-9.39052500	-0.29028200	-4.07216200
C	-8.71627800	0.81573200	-3.27661500
O	6.42618000	1.27069800	-2.26843100
O	6.46211700	0.52689100	2.42143300
O	0.11979900	-2.30261300	-2.16647800
O	-4.51285100	2.51421600	-4.75191000
O	-2.83091100	2.71922500	-1.11505900
O	-4.78505300	2.94676300	-2.19293200
O	-7.09065500	0.04588400	2.59854700
O	-5.30662100	0.27133000	4.74813000
O	-6.50418900	-3.90167500	0.64660800
O	-6.83377100	4.05502000	-3.60521000
O	-5.02777900	-2.09882500	2.22992900
O	-8.09288900	0.55546600	-2.23179300
H	12.28269100	0.30705900	-3.91196000
H	12.40230200	-0.28748800	-2.26542200
H	10.02186200	0.75654600	-4.93310900
H	10.39281200	-0.47387400	-0.83612400
H	7.68026300	1.45183000	-4.50066200
H	8.07908300	0.23378700	-0.40577000
H	6.06268800	1.04904700	-1.36639800
H	12.98208400	-2.08757600	-3.89932600
H	10.07601200	-4.11526600	4.24856300
H	9.62516700	-4.77338200	2.68499300

H	8.27829200	-3.44288700	1.09286200
H	9.53827000	-1.72564600	4.81874900
H	6.73282400	-1.54502900	0.73767100
H	8.00088900	0.18735000	4.44797000
H	6.01805900	0.54816800	1.52362500
H	11.85586900	-2.70290100	3.14524500
H	6.11453000	-1.37252700	-2.76827300
H	6.24407000	-2.65331100	-1.58564000
H	3.95689400	-1.69399700	-4.45526100
H	4.25759100	-3.21743400	-0.45482800
H	1.48673600	-1.65358200	-4.24654200
H	1.79667300	-3.16820100	-0.24659300
H	-0.17390100	-2.25371200	-1.23476000
H	6.07666900	-3.09580900	-4.62967600
H	0.35144000	2.16170100	-8.52348200
H	1.19347500	2.78489200	-7.11432700
H	-2.03574400	1.49892500	-8.38333500
H	0.02549400	2.95080000	-4.91108300
H	-4.19707100	1.70679000	-7.18122800
H	-2.12748600	3.18071000	-3.72256700
H	-4.41123800	2.67287000	-3.77086800
H	2.07714300	0.53198700	-7.74332300
H	1.85082900	-7.24913800	-4.85825700
H	4.00354100	-8.46504700	-4.46117400
H	4.68729200	-7.01017600	-3.71865300
H	4.23422700	-6.99547300	-5.42267700
H	0.97058600	-7.23290000	-2.61102200
H	2.00880900	-8.62291500	-2.87677600
H	2.37210100	-7.67327800	-0.61128900
H	2.80301800	-6.10174200	-1.29166300
H	3.85266000	-7.49991600	-1.55379000
H	3.12842500	-4.92418800	-3.30968800
H	0.33451100	-6.92752100	2.01971200
H	-1.51167600	-6.16674900	3.52281200
H	-2.58983500	-7.38223000	2.82445100
H	-1.17578000	-7.88879200	3.76113800
H	-1.17569300	-5.09824300	1.18914800
H	-2.22079300	-6.31185300	0.44371000
H	-0.56538500	-6.08855800	-0.14386100
H	-0.33269600	-9.32489300	1.80070100
H	-2.58466900	-5.73881500	-2.02075300

H	-4.26714600	-5.93420500	-2.48600700
H	-2.50788800	-3.51566300	-3.17506100
H	-4.19016200	-3.71103700	-3.67177100
H	-4.84321700	-3.82259800	-1.20319100
H	-3.17540600	-3.49845400	-0.78059300
H	-3.31704400	-1.32213000	-1.82498500
H	-7.30380500	-1.27991300	-1.66922000
H	-6.53999100	-2.66423600	-0.93955100
H	-6.14763100	0.53758400	-2.23138900
H	-4.41463100	0.39133500	-2.55754100
H	-3.59814200	-5.90082900	-4.92439400
H	-6.76946800	3.33385600	-0.53155800
H	-6.06484200	4.93798500	-0.60209200
H	-4.05281100	4.08175400	0.70105700
H	-4.79404000	2.50089300	0.76785800
H	-6.15026900	5.06799800	1.93926200
H	-8.31425100	-1.32187400	3.57318800
H	-8.86118200	-0.33772800	0.72576000
H	-8.19666200	-1.91947000	1.19123800
H	-9.87366400	-1.51017300	1.59844200
H	-6.67741600	0.26636500	3.45854900
H	-10.35424900	0.09536400	3.56228400
H	-4.52249400	1.88701200	3.70101200
H	-2.28699100	1.81156500	4.79796900
H	-2.67227600	0.24676700	4.07396200
H	-2.82810400	0.46371900	5.82448300
H	-4.87221200	-0.48002800	4.31281900
H	-4.71478600	1.94660600	6.75969500
H	7.08121400	7.22662100	-0.00278600
H	8.26829400	6.31641500	0.91543100
H	5.46088300	5.33379800	0.22269000
H	6.88395400	5.57368300	-1.81060100
H	6.61660100	3.85741300	-1.44972300
H	8.15999300	4.61308300	-1.04816000
H	7.82242900	3.79980300	1.44206500
H	6.32359000	4.21767400	2.28445000
H	6.28882100	3.08300800	0.93600000
H	6.76744100	6.37680600	2.94030200
H	-7.50712500	-4.87477500	2.17419400
H	-5.04605800	-5.11599800	2.55744100
H	-5.83592700	-6.69846800	2.43952200

H	-4.90520200	-6.06145500	1.06575700
H	-5.96360700	-3.33847400	1.25110100
H	-7.92878500	-6.93711400	0.87509300
H	-7.84435300	-0.23016700	-5.58475600
H	-7.55428300	-1.40040100	-4.31085200
H	-10.20194900	0.10694000	-4.6947710
H	-9.82724600	-1.00744000	-3.37037500
H	-9.39305600	-2.86857100	-5.23582600
H	-6.29288200	3.81388600	-4.37451000
H	-6.23087700	3.81452400	-2.86950400
H	-4.15324500	-1.97724400	1.80264300
H	-5.56287500	-1.29361700	2.09357500
H	-9.68542600	-1.68723300	-6.51892600
H	-8.14606000	-2.55526300	-6.44879000
H	-8.48551100	-5.51429300	-0.03113700
H	-6.96389200	-6.31080000	-0.47874700
H	-2.77382000	-7.19877500	-4.04852500
H	-1.90580500	-5.71123900	-4.44981200
H	-9.49553700	1.35375900	2.64330000
H	-9.00671500	0.97378900	4.30656300
H	-7.75396800	4.82639400	1.23250900
H	-6.89926300	3.46313000	1.96755500
H	5.52484100	7.20945600	2.00033000
H	7.06559700	8.01035500	2.33949000
H	-4.23995900	3.30317000	5.71085100
H	-5.89440600	2.66591800	5.64532400
H	11.51618700	-2.53117000	-3.0037360
H	11.38697500	-1.93305200	-4.6601240
H	11.40451900	-3.37130400	1.57465800
H	12.09950500	-4.42930800	2.81768400
H	0.46002400	-0.17238200	-7.55567500
H	1.31635800	0.46060900	-6.14537000
H	1.43798200	-5.01481700	-3.80632500
H	2.71792900	-4.94363500	-5.02231300
H	6.24177300	-4.37753300	-3.42490500
H	7.54255000	-3.18874300	-3.64473400
H	-1.72908800	-8.85416800	0.81982400
H	-0.09029900	-8.59181800	0.20643700
H	-8.09969100	2.79131000	-3.54283800
H	-9.23123200	2.16717600	-4.69970200
H	-0.06404700	-2.52329000	7.65293200

H	0.33203600	-3.00252100	5.25973400
H	-1.59546300	-3.21819600	3.68989700
H	-3.90727500	-3.06078800	4.56426300
H	-4.30363800	-2.61527100	6.96976700
H	-1.53182700	-2.19501500	9.50193300
H	-3.16948100	-2.82674600	9.39477200
H	-2.32251700	0.01863000	8.60512000
H	-3.22759000	-0.42292000	10.06170600
H	-3.96331000	-0.61839900	8.46372600
H	3.47625500	-1.60620500	5.64964700
H	2.70966300	-1.15680700	4.12035200
H	4.44819100	-0.94560400	4.32676900
H	-0.19853700	0.80692800	5.87944800
H	0.72108200	1.55047400	4.56362800
H	0.63057700	-0.21397200	4.71066300
H	1.87064700	-0.10885500	6.86371100
H	2.12491500	2.90437200	6.39617400
H	2.85341600	1.98523200	7.71929500
H	1.09203500	2.13716400	7.60898700
H	4.11061400	0.79041700	6.05005400
H	3.31965500	1.27601900	4.56568600
H	0.47385300	4.75930800	5.08584500
H	0.85964600	5.78309300	2.22346800
H	2.77064200	4.36384300	2.91487800
H	1.66562300	3.06953900	3.39007600
H	1.79391100	3.54505800	1.69246600
H	-0.95829600	3.54078800	3.24044400
H	-1.49041200	5.15556900	2.74164700
H	-0.77672000	4.06792900	1.55415000
H	-2.43191900	10.14689800	1.66296600
H	-1.79967500	9.54843100	-0.65282000
H	-3.28529800	8.73423500	-0.21249500
H	0.95925300	8.91643600	-0.20783500
H	3.12341400	7.78361500	-0.61913600
H	3.21050100	5.37454400	-1.18014100
H	1.13184800	4.03016700	-1.35215700
H	-1.71369700	4.27614100	-1.04034100
H	-3.35726200	6.09453200	-0.60329800
H	1.65227100	5.98021900	4.58739300
H	-0.08080700	6.31692100	4.45447400
H	-0.92615400	9.21507600	1.69590300
H	-2.44664500	8.43681500	2.13143300
H	-2.19824500	0.69501700	1.25698300
O	-2.50187900	0.45916200	-2.48281200
H	-2.05472400	0.62487300	-3.32407800
H	-2.52876700	1.34058200	-2.01730100
C	-1.13579600	0.90720900	1.36216400
C	-0.34040300	0.30676700	0.23374700
C	1.07847000	0.35568200	0.21902500
C	1.87545200	0.30809200	1.40022000
C	3.24058700	0.42894200	1.35567200
C	3.93828200	0.62816700	0.11234900
C	3.13343300	0.71080400	-1.07310700
C	1.77153600	0.53949400	-1.01582900
O	5.22413800	0.71976200	0.07779700
H	1.39018100	0.15537800	2.35928400
H	3.83517500	0.37526000	2.26081400
H	3.63843500	0.86675800	-2.02048800
H	1.18939800	0.56733300	-1.93321000
H	-0.81481700	0.38450400	-0.74053700
H	-0.79682700	0.56965200	2.34296800
H	-0.99611600	1.99076800	1.31061700
C	-1.24397100	-1.62283500	0.49301600
N	-2.18940900	-2.06064400	1.03875400

Prod (-17.5 kcal/mol)

C	-2.06553300	9.06122600	1.99047600
C	-2.28324900	8.74218700	0.51733900
C	-1.65787600	7.44996200	0.07563300
C	-2.31213100	6.27059700	-0.18307900
C	-0.25574600	7.17808800	-0.12603900
C	-0.14439500	5.80724700	-0.50138200
C	0.91478600	7.95344400	-0.03456500
N	-1.41752200	5.28136000	-0.53600100
C	1.09306100	5.20984500	-0.77102100
C	2.14240400	7.36225600	-0.30499400
C	2.23065100	6.00158200	-0.66602300
C	0.61209400	5.27541600	4.66344500
C	0.60274700	4.77707300	3.21599500
C	1.85319800	3.94619100	2.90252500
C	-0.68816200	3.99961900	2.92715200
N	-4.08343800	-1.91307100	-1.52373300
N	-6.43645600	-1.88458800	-1.65160600
N	-5.19695400	-0.00828300	-2.19206700
N	-8.73156200	2.20889600	-3.56841700
C	1.94456500	1.60170400	7.13847200
C	1.89194700	0.38463300	6.21399200
C	3.23975700	0.18455200	5.49045900
C	0.70749200	0.46531800	5.24590200
C	3.44726700	-1.21173800	4.89850800
C	-3.08768600	-1.23028500	8.98523000
C	-2.52495600	-2.63763100	8.81224500
C	-2.24156300	-2.96939000	7.35782400
C	-3.29477100	-3.12128000	6.44200100
C	-0.93002400	-3.08486000	6.88149900
C	-3.04526000	-3.36692900	5.09154700
C	-0.67255100	-3.33918300	5.53217000
C	-1.72959800	-3.47561000	4.63185400
C	11.86277900	-1.59797900	-3.81291200
C	11.81467400	-0.24824500	-3.07231200
C	10.41534900	0.19541700	-2.69623100
C	9.60957700	0.90524800	-3.59888600
C	9.86328200	-0.10490100	-1.44430200
C	8.31233300	1.29003000	-3.27407600
C	8.56720900	0.27507200	-1.09744400
C	7.76658300	0.97492000	-2.01643500
C	11.36135100	-3.72379900	2.44480800
C	10.03537600	-4.17100600	3.08396200
C	8.98512200	-3.08340000	3.04830500
C	8.78688000	-2.21911700	4.13343100
C	8.22652200	-2.85383100	1.89290500
C	7.88813500	-1.15792300	4.06734700
C	7.32017300	-1.80086200	1.80703900
C	7.15170000	-0.92144600	2.89398900
C	6.38447400	-3.11009200	-3.80236400
C	5.75857800	-1.75207200	-4.17537900
C	4.24900100	-1.74475000	-4.06084600
C	3.41574400	-1.75750000	-5.18627300
C	3.63339300	-1.75678700	-2.80152800
C	2.24874000	-1.81778200	-2.66505200
C	1.43434000	-1.85579100	-3.80355500
C	2.02587100	-1.80953800	-5.06959300
C	1.04754800	1.03438800	-7.14865300
C	0.39998700	2.42222300	-7.30649900
C	-0.91389500	2.54890300	-6.56492700
C	-2.13277500	2.23574000	-7.18238900
C	-0.95345800	2.93916900	-5.22044100
C	-3.33978600	2.28659000	-6.48909600
C	-2.15284200	3.00331000	-4.50923700
C	-3.35953300	2.66086400	-5.13996300
C	2.37791000	-5.12222800	-4.33247100
C	2.62239600	-6.57696100	-3.90250900
C	2.30948900	-6.81092800	-2.40665800
C	4.04044200	-7.03389200	-4.27878300
C	3.15350400	-6.00541700	-1.41042800
C	-0.77541300	-8.61820600	0.63264000
C	-0.66552400	-7.35833100	1.50353300
C	-1.02483000	-6.10001600	0.70189400
C	-1.53272400	-7.47952200	2.76463900
C	-2.94452100	-5.87105700	-4.45393800
C	-3.31582700	-5.37393300	-3.05328600
C	-3.57627000	-3.86314900	-3.00658500
C	-3.88498500	-3.36700400	-1.58816900
C	-5.24241100	-1.27326400	-1.78892900
C	-6.83854600	4.27495800	1.63215000
C	-6.15252700	3.96925700	0.30045200
C	-4.75088100	3.37726000	0.46608300

C	-4.05381300	2.99851800	-0.84410500
C	-9.44036500	0.33888400	3.41786700
C	-8.49895700	-0.69091100	2.79532500
C	-8.98192300	-1.18343200	1.43539600
C	-4.90786500	2.05009900	5.91463200
C	-4.48287200	1.15392300	4.75540600
C	-3.05354000	0.65017900	4.90592200
C	6.53369800	6.91538500	2.49982200
C	7.13990400	6.39922700	1.19032600
C	6.54657300	5.07501400	0.66866400
C	7.09145000	4.76145200	-0.73276100
C	6.79678100	3.89647100	1.62143500
C	-7.66927200	-6.04595800	0.04088000
C	-6.98004400	-5.18689200	1.09865600
C	-5.74271600	-5.86738600	1.68498500
C	-8.99698700	-1.75556900	-5.92480200
C	-8.36089000	-0.74569000	-4.97480400
C	-9.38226700	-0.09274500	-4.02030800
C	-8.66255100	0.93212300	-3.16040600
O	6.50341500	1.36626100	-1.75812700
O	6.30508500	0.12334300	2.86270000
O	0.06872000	-1.94469200	-3.73273900
O	-4.55490500	2.67896100	-4.48991900
O	-2.82120500	2.70579700	-0.79370100
O	-4.76005000	2.95858700	-1.89587100
O	-7.19212100	-0.12197500	2.59235800
O	-5.40998200	0.03998500	4.70347800
O	-6.63162300	-3.93249400	0.49322800
O	-6.79312900	4.17751800	-3.24097400
O	-5.32861000	-2.03192800	2.09581600
O	-7.98928100	0.56804100	-2.17734900
H	12.28568600	0.51731400	-3.70321900
H	12.43054400	-0.31877300	-2.16668700
H	10.00852700	1.16638500	-4.57800000
H	10.46046300	-0.64588800	-0.71233000
H	7.69758100	1.84171700	-3.97879900
H	8.17286100	0.03652200	-0.11570400
H	6.15569800	1.04771700	-0.86198500
H	12.89246000	-1.87900800	-4.06485200
H	10.21964200	-4.47725800	4.12175500
H	9.66938300	-5.06327200	2.55895700

H	8.34751000	-3.51392300	1.03556300
H	9.35603800	-2.37528600	5.04865400
H	6.73845300	-1.63585100	0.90635600
H	7.74812300	-0.48628300	4.90899600
H	5.96229400	0.28029700	1.91927400
H	11.77030800	-2.85358900	2.96886500
H	6.04883300	-1.49050800	-5.20029800
H	6.17828400	-0.97448600	-3.52763600
H	3.86112500	-1.73629800	-6.17875200
H	4.24359600	-1.70557700	-1.90403400
H	1.38904400	-1.83641300	-5.94784200
H	1.80769300	-1.82759500	-1.67454100
H	-0.19749400	-2.04755000	-2.80292800
H	6.01936700	-3.90633800	-4.46071200
H	0.23917400	2.62874300	-8.37232000
H	1.09937300	3.18644900	-6.94613400
H	-2.13680300	1.93770300	-8.22890800
H	-0.02850900	3.20872800	-4.71570400
H	-4.27744200	2.03216300	-6.97387900
H	-2.16162100	3.33349900	-3.47443900
H	-4.42437100	2.76928700	-3.50634200
H	1.99292200	0.97622300	-7.69923400
H	1.91896400	-7.21192500	-4.46308800
H	4.21713600	-8.07742800	-3.99160600
H	4.80312500	-6.41840900	-3.78979800
H	4.20193900	-6.95235700	-5.35959900
H	1.24834200	-6.57952400	-2.23908300
H	2.42484100	-7.88278000	-2.18977200
H	2.86413100	-6.24197600	-0.38078900
H	3.02574100	-4.92778900	-1.54754500
H	4.22120700	-6.22825200	-1.50667200
H	3.07860100	-4.43246600	-3.85747200
H	0.38206000	-7.26290500	1.82384500
H	-1.43462500	-6.59735300	3.40655000
H	-2.59309700	-7.57985400	2.49929400
H	-1.25579000	-8.35809900	3.35831900
H	-0.91212400	-5.19197500	1.30376400
H	-2.06607200	-6.14330800	0.35680900
H	-0.38521300	-5.98850300	-0.17975700
H	-0.48609300	-9.51637400	1.18981900
H	-2.50626900	-5.62086400	-2.35429500

H	-4.20571900	-5.91080100	-2.69481500
H	-2.69384600	-3.33037100	-3.38307100
H	-4.41139400	-3.60682200	-3.67253700
H	-4.76116300	-3.86734900	-1.16862700
H	-3.03939200	-3.58849500	-0.93295300
H	-3.25078100	-1.34778200	-1.69096500
H	-7.24377400	-1.27234100	-1.76053400
H	-6.53102400	-2.70119700	-1.05123300
H	-6.04186300	0.55610500	-2.18320800
H	-4.28560500	0.40748500	-2.40402000
H	-3.74410700	-5.66220500	-5.17415200
H	-6.76513900	3.26658500	-0.27410600
H	-6.09194100	4.88432000	-0.30236100
H	-4.08891400	4.05038400	1.02337800
H	-4.80055400	2.45590500	1.06457400
H	-6.25361700	4.97859800	2.23613600
H	-8.41513000	-1.55120700	3.47903000
H	-8.97355300	-0.36230800	0.71089000
H	-8.33927000	-1.98466700	1.05861400
H	-10.00214900	-1.57344200	1.51055800
H	-6.78897100	0.10742200	3.45850900
H	-10.43111800	-0.09509800	3.58962700
H	-4.56740200	1.72192800	3.81616200
H	-2.35277300	1.48924200	4.92687700
H	-2.76650300	0.00523600	4.06865200
H	-2.94128000	0.07448100	5.82968800
H	-4.98044800	-0.68168200	4.21619000
H	-4.81195600	1.51445700	6.86442800
H	7.01243600	7.16800000	0.41520000
H	8.22580600	6.27371300	1.31427400
H	5.45677500	5.21298500	0.58441400
H	6.86503300	5.56992700	-1.43899100
H	6.67406500	3.83093900	-1.12850100
H	8.18227600	4.64267800	-0.70591900
H	7.87348100	3.72984100	1.75593800
H	6.36145100	4.06680400	2.61147100
H	6.36539100	2.97062300	1.22917200
H	6.71334700	6.22709100	3.33162900
H	-7.69719100	-4.99649900	1.91356300
H	-5.25445100	-5.22233100	2.42240400
H	-6.01222400	-6.80480700	2.18338400

H	-5.01790500	-6.09246400	0.89551500
H	-6.16943700	-3.36880600	1.15510800
H	-8.00296900	-6.99330000	0.47490700
H	-7.86002200	0.04129000	-5.55202700
H	-7.58214900	-1.22994700	-4.37623700
H	-10.19398800	0.36881400	-4.5957920
H	-9.82519100	-0.84978200	-3.36547000
H	-9.47997100	-2.57162300	-5.37526600
H	-6.25553100	3.98399500	-4.02667000
H	-6.18823700	3.88562000	-2.52544000
H	-4.67666900	-1.72428800	1.44986000
H	-6.01497400	-1.31709800	2.14048100
H	-9.75991300	-1.28545900	-6.55605900
H	-8.24676000	-2.20013100	-6.58625200
H	-8.53979700	-5.52634600	-0.37025600
H	-6.97975600	-6.26575200	-0.78118000
H	-2.76437100	-6.95101800	-4.45907300
H	-2.03585100	-5.37855700	-4.81569300
H	-9.54824400	1.20371100	2.75540400
H	-9.05565100	0.69046900	4.38140500
H	-7.82916000	4.71504600	1.47630400
H	-6.97121600	3.36000500	2.22249100
H	5.44868100	7.04393900	2.40741800
H	6.96001600	7.88602900	2.77825000
H	-4.27858400	2.94403800	5.95024400
H	-5.94910900	2.36714100	5.80098500
H	11.43307400	-2.39506400	-3.1972070
H	11.28532600	-1.55443100	-4.7425520
H	11.20945900	-3.43493700	1.39980200
H	12.11252800	-4.52279100	2.47124300
H	0.38354700	0.24975700	-7.52717100
H	1.25645800	0.81046500	-6.09945100
H	1.36510800	-4.79350500	-4.07822300
H	2.49825400	-5.01179100	-5.41527000
H	6.13381800	-3.38707200	-2.77336300
H	7.47627900	-3.07129000	-3.88105100
H	-1.80569500	-8.76177200	0.28288300
H	-0.13157100	-8.54862000	-0.25064300
H	-8.04523600	2.92355900	-3.26056900
H	-9.23839600	2.41620600	-4.41532500
H	-0.09907100	-2.97052600	7.57336800

H	0.35361600	-3.42144800	5.18615800
H	-1.53183100	-3.66288800	3.58038000
H	-3.87309400	-3.46174200	4.39339900
H	-4.32194800	-3.03876300	6.79027900
H	-1.60319300	-2.74286700	9.39534300
H	-3.23687400	-3.36583000	9.22334200
H	-2.38037100	-0.47806800	8.62079700
H	-3.29717300	-1.01199300	10.03842100
H	-4.02084400	-1.10422300	8.42657300
H	3.41668800	-1.98126200	5.68047600
H	2.68046300	-1.45677900	4.15645700
H	4.41766600	-1.26992400	4.39925200
H	-0.23661400	0.56131100	5.79251400
H	0.80358900	1.32974500	4.57987400
H	0.63463700	-0.42869200	4.61976800
H	1.73230900	-0.50006300	6.85263400
H	2.13564600	2.52047600	6.57008000
H	2.74464000	1.50274200	7.88056800
H	1.00047300	1.73988900	7.67935100
H	4.05305900	0.38444500	6.20060400
H	3.34508000	0.93396500	4.69518100
H	0.58887900	4.43477800	5.36904900
H	0.61241300	5.65434700	2.55510000
H	2.76476400	4.53465500	3.05289300
H	1.91996100	3.06551800	3.55253300
H	1.85529000	3.59460600	1.86698000
H	-0.75681500	3.10991400	3.56687600
H	-1.57361700	4.61686500	3.11979000
H	-0.73650600	3.67362700	1.88404800
H	-2.51922300	10.02149300	2.26353300
H	-1.88869700	9.56934600	-0.09007700
H	-3.36022600	8.70795700	0.30843200
H	0.86024700	9.00302800	0.24366000
H	3.05213100	7.95189400	-0.23507400
H	3.20380500	5.56320500	-0.86570000
H	1.16660500	4.16116200	-1.04157300
H	-1.68348300	4.30921900	-0.68039900
H	-3.37275800	6.06225300	-0.15705200
H	1.51311900	5.86064200	4.87954200
H	-0.25659500	5.91005500	4.87567800
H	-0.99913000	9.11296300	2.23237500

H	-2.50459400	8.28596000	2.62740200
H	-2.09859200	0.30529800	1.40918300
O	-2.45917500	0.56065800	-2.32724300
H	-2.00507500	0.74591500	-3.16118400
H	-2.49328800	1.42387700	-1.82843300
C	-1.01516000	0.31464800	1.56702000
C	-0.26780800	0.12929700	0.22909300
C	1.24791800	0.24999700	0.31381100
C	2.00603300	-0.56086800	1.17195800
C	3.38876000	-0.45904000	1.22530400
C	4.10016500	0.46667900	0.41138800
C	3.32132200	1.27015100	-0.46455500
C	1.93731900	1.15267500	-0.50893200
O	5.41008300	0.56105400	0.47016000
H	1.50825700	-1.29532900	1.80317300
H	3.95621900	-1.09616200	1.89466900
H	3.83525000	1.97006100	-1.11615100
H	1.37561800	1.76612100	-1.21184600
H	-0.64326800	0.88443800	-0.46969200
H	-0.75287500	-0.46849100	2.28304700
H	-0.73546000	1.27847800	1.99240200
C	-0.63822500	-1.16051100	-0.37666200
N	-0.89730300	-2.18412300	-0.86730500

TS1' (18.7 kcal/mol)

C	-1.67093200	9.11828900	1.66278900
C	-1.84983500	8.75654100	0.19339100
C	-1.34406100	7.38868700	-0.17000300
C	-2.10354100	6.25813100	-0.35378900
C	0.02509400	6.98217900	-0.37316200
C	0.00746200	5.58768000	-0.67184500
C	1.26143300	7.65383100	-0.35217300
N	-1.30513300	5.17454800	-0.65263000
C	1.17949500	4.86735300	-0.93568200
C	2.42426400	6.94206900	-0.61945700
C	2.38331300	5.56094900	-0.90520400
C	0.67404200	5.28620900	4.57466600
C	0.69072300	4.74049900	3.14580400
C	1.99829500	3.99439800	2.86295300
C	-0.54681700	3.87556700	2.87276300
N	-4.26808700	-1.72968100	-1.82942000
N	-6.62405200	-1.63115000	-1.76972000
N	-5.36732300	0.18432900	-2.46329300
N	-8.67527700	2.46078200	-3.97303600
C	1.69020100	1.63857000	7.22984900
C	1.60364800	0.39378100	6.34538300
C	2.99643400	0.02065000	5.79793300
C	0.55678600	0.57243800	5.24015600
C	3.07409600	-1.33822600	5.09409100
C	-3.56700100	-0.83071000	8.96437800
C	-3.08172100	-2.27336600	8.86323700
C	-2.70864100	-2.65116400	7.44164300
C	-3.69071300	-2.70824800	6.44018100
C	-1.37942400	-2.89717300	7.07852800
C	-3.35342600	-2.97757400	5.11419800
C	-1.03543900	-3.17624600	5.75304800
C	-2.01938800	-3.20673600	4.76457400
C	11.82698500	-2.52657300	-3.18444800
C	11.86966000	-1.05360400	-3.62569500
C	10.54179700	-0.36638800	-3.40268900
C	9.63463300	-0.15579800	-4.44810500
C	10.13897000	-0.00462900	-2.11041300
C	8.36392600	0.36674000	-4.21478500
C	8.87541300	0.51742900	-1.85594200
C	7.96200900	0.68989000	-2.91064900

C	10.95696900	-4.38570000	3.11768300
C	9.66904800	-3.89943100	3.80608700
C	9.15329000	-2.58409700	3.26037200
C	9.61913000	-1.35821600	3.75717600
C	8.21964400	-2.54258500	2.21703900
C	9.17573300	-0.14451400	3.24024300
C	7.76369300	-1.33798600	1.68388000
C	8.23868900	-0.11869200	2.19503000
C	6.27500600	-3.72273700	-3.34763400
C	5.74105400	-2.59122700	-2.44894800
C	4.23491400	-2.58386100	-2.26831100
C	3.38582000	-2.12064600	-3.28615000
C	3.63423400	-3.00430900	-1.07546700
C	2.24947500	-2.96174000	-0.89303900
C	1.42631700	-2.48516100	-1.91707200
C	2.00379300	-2.06970200	-3.12307100
C	1.32364000	0.59181300	-7.05433200
C	0.75197000	2.00001800	-7.30371400
C	-0.59247200	2.23157700	-6.64515600
C	-1.79203300	1.96075400	-7.31903500
C	-0.68637400	2.69803700	-5.32766300
C	-3.03141100	2.12904200	-6.70647600
C	-1.91877100	2.87945200	-4.69633100
C	-3.10892400	2.58300600	-5.38241300
C	2.17878100	-5.52062100	-3.97099700
C	2.14207500	-7.05483800	-3.95358000
C	1.69206200	-7.62171800	-2.58760600
C	3.48299500	-7.64491400	-4.41764800
C	2.60212200	-7.29997800	-1.39504600
C	-1.36451000	-8.64609300	0.97697900
C	-1.35118500	-7.29279100	1.70348600
C	-1.62396700	-6.14065800	0.72600700
C	-2.35028200	-7.27775800	2.86877200
C	-3.16825400	-5.96990000	-4.28786700
C	-3.54541000	-5.34097700	-2.94510800
C	-3.75738600	-3.82761800	-3.04808100
C	-4.10201900	-3.17576700	-1.70349600
C	-5.41891600	-1.06731100	-2.00498400
C	-6.69853800	4.60190000	1.27490600
C	-6.04886900	4.24930800	-0.06278500
C	-4.73100900	3.49225000	0.10759200

C	-4.03089800	3.10233200	-1.19376600
C	-9.59403900	0.88955900	3.08766300
C	-8.66913700	-0.20751000	2.54973000
C	-9.09483700	-0.70477600	1.17238700
C	-5.07063200	2.43149700	5.70822200
C	-4.74469600	1.46030100	4.57446300
C	-3.30724800	0.95473600	4.62346300
C	6.76107100	6.50600100	2.60160100
C	6.67309100	6.22159700	1.09811500
C	7.06104000	4.78957900	0.67850200
C	6.77116100	4.57127500	-0.81310900
C	8.52496800	4.45287200	0.99840600
C	-8.06743800	-5.70723300	0.01217100
C	-7.46394600	-4.79232700	1.07813700
C	-6.44295800	-5.52106200	1.95314300
C	-8.90721000	-1.57322700	-6.14968300
C	-8.31189800	-0.54928300	-5.18771400
C	-9.37754200	0.15864400	-4.32415300
C	-8.69296600	1.21356200	-3.47054200
O	6.70736100	1.15811000	-2.71698300
O	7.83336600	1.08487600	1.73102800
O	0.06187700	-2.40849000	-1.80844000
O	-4.33530100	2.71701800	-4.81676600
O	-2.81910600	2.73928700	-1.12069300
O	-4.71049700	3.12056700	-2.26439500
O	-7.32168800	0.27358600	2.42366800
O	-5.68058000	0.35815700	4.67142900
O	-6.85283900	-3.66807900	0.42771300
O	-6.64769800	4.36505400	-3.69779600
O	-5.60908100	-2.00218100	2.28671500
O	-8.12154600	0.89963700	-2.41080800
H	12.14653500	-0.99728200	-4.68585700
H	12.66290800	-0.53621700	-3.06966200
H	9.92185000	-0.41887000	-5.46468800
H	10.82488300	-0.14448200	-1.27718900
H	7.65983600	0.51819300	-5.02736500
H	8.59069700	0.78642100	-0.84478400
H	6.47227200	1.10888300	-1.74487900
H	12.79129000	-3.02471700	-3.34288000
H	9.85669300	-3.80268800	4.88344600
H	8.89485200	-4.66957100	3.69829000
H	7.83564700	-3.47599300	1.80927500
H	10.34110300	-1.35371900	4.57207500
H	7.04192500	-1.32590200	0.87332200
H	9.53536900	0.80061400	3.63529100
H	7.13003400	0.99117100	1.02244900
H	11.76198800	-3.65230800	3.23356200
H	6.05568000	-1.62651400	-2.86275000
H	6.21880800	-2.66436700	-1.46523400
H	3.81858100	-1.77229300	-4.22111000
H	4.26031100	-3.35358600	-0.25805800
H	1.35750500	-1.69780600	-3.91218300
H	1.81313900	-3.27717500	0.05192600
H	-0.20843500	-2.52591700	-0.88463000
H	5.83400300	-3.67461900	-4.34938400
H	0.66074000	2.16239300	-8.38509100
H	1.47014900	2.74485500	-6.94015600
H	-1.75577000	1.60672100	-8.34746500
H	0.22175900	2.93993000	-4.77977700
H	-3.95347400	1.90936700	-7.23602800
H	-1.96462700	3.26089600	-3.68040800
H	-4.27182400	2.86167000	-3.83001300
H	2.29278300	0.46578800	-7.54909700
H	1.37981500	-7.37530100	-4.67999500
H	3.46365900	-8.74125800	-4.41197500
H	4.30949800	-7.32143900	-3.77531500
H	3.71984000	-7.32003100	-5.43680800
H	0.68126500	-7.24646900	-2.37206700
H	1.59561500	-8.71278500	-2.67979400
H	2.20768100	-7.75104000	-0.47785000
H	2.68183300	-6.22229200	-1.22377800
H	3.61517100	-7.69020400	-1.53909200
H	2.94988800	-5.11914500	-3.30792600
H	-0.34460000	-7.14735600	2.12228500
H	-2.31330200	-6.33005500	3.41624600
H	-3.37672700	-7.40960400	2.50356300
H	-2.14521600	-8.08520600	3.58088000
H	-1.60972600	-5.16773000	1.22842200
H	-2.61189900	-6.25389200	0.26225200
H	-0.87589900	-6.11825200	-0.07572200
H	-1.13466600	-9.47001300	1.66170600
H	-2.75784700	-5.54986300	-2.20843300

H	-4.45935200	-5.81372700	-2.55925400
H	-2.84592900	-3.35905500	-3.44238800
H	-4.55942200	-3.61210100	-3.76685300
H	-5.00283100	-3.61288100	-1.26581300
H	-3.30180500	-3.34262900	-0.97776200
H	-3.43113600	-1.17100400	-2.00459800
H	-7.41194400	-0.99259500	-1.85023900
H	-6.71570800	-2.41032300	-1.11968200
H	-6.18523000	0.78193800	-2.39518100
H	-4.45675000	0.57305600	-2.70539300
H	-3.94651200	-5.79950100	-5.03968000
H	-6.73516800	3.63610400	-0.65640600
H	-5.87427500	5.16378100	-0.64423800
H	-4.01915500	4.05580700	0.72190600
H	-4.90883300	2.55299900	0.65113800
H	-6.03799500	5.22261900	1.89177400
H	-8.68561300	-1.05596500	3.25320800
H	-9.00353600	0.09803700	0.43296500
H	-8.46854700	-1.54342200	0.85412900
H	-10.13592900	-1.04407200	1.19147000
H	-6.96116500	0.45363600	3.31786300
H	-10.61773000	0.51614800	3.19845400
H	-4.90964000	1.97084100	3.61304700
H	-2.60567800	1.79007100	4.53435900
H	-3.10178800	0.26159800	3.80014500
H	-3.11385600	0.43122900	5.56515600
H	-5.28940700	-0.41621300	4.23436400
H	-4.88957000	1.95822400	6.67851700
H	5.64386000	6.41253300	0.76591000
H	7.30664400	6.93675900	0.55213900
H	6.42872200	4.09252000	1.24762300
H	5.72711600	4.80662400	-1.04941700
H	6.94910600	3.53501600	-1.11460600
H	7.40525300	5.21621200	-1.43535000
H	9.20603800	5.10034500	0.42994100
H	8.75298000	4.57722000	2.06185500
H	8.74659100	3.41275500	0.74555200
H	7.78672300	6.42265400	2.97528300
H	-8.28100600	-4.42708800	1.72202800
H	-6.01720300	-4.84056100	2.69627000
H	-6.90840200	-6.35962200	2.48296200
H	-5.62410200	-5.90980200	1.33836400
H	-6.39770200	-3.12434800	1.11766500
H	-8.59437500	-6.54396600	0.48115000
H	-7.75268700	0.20653900	-5.75291300
H	-7.58789600	-1.03258600	-4.52345200
H	-10.14649300	0.60596700	-4.9663480
H	-9.86960700	-0.56152100	-3.66338700
H	-9.45357200	-2.35639200	-5.61174600
H	-6.10742100	4.11635000	-4.46464100
H	-6.06909700	4.07667000	-2.95877500
H	-4.69321200	-1.91697400	1.93895600
H	-6.08128700	-1.15884900	2.12252600
H	-9.60710300	-1.10432700	-6.85099100
H	-8.12387000	-2.06087800	-6.73856600
H	-8.77725100	-5.15774300	-0.61364600
H	-7.28057600	-6.11091400	-0.63425600
H	-3.02372700	-7.05228900	-4.19744000
H	-2.23574100	-5.54288500	-4.67448600
H	-9.60584700	1.74419800	2.40332700
H	-9.25476500	1.24063600	4.06839700
H	-7.63369300	5.15355300	1.13160600
H	-6.93202500	3.69487000	1.84566400
H	6.14402200	5.80153700	3.17131000
H	6.40821400	7.51718200	2.83325500
H	-4.44661700	3.32706700	5.63151400
H	-6.12013800	2.73803800	5.66470800
H	11.57484500	-2.60717800	-2.1224210
H	11.06348300	-3.07592700	-3.7452160
H	10.79487300	-4.52706500	2.04406500
H	11.30066500	-5.33781900	3.53898800
H	0.64455200	-0.17980300	-7.43241100
H	1.46615100	0.41247200	-5.98358900
H	1.22066500	-5.09274400	-3.65653600
H	2.39255700	-5.14726400	-4.97897900
H	6.03550100	-4.70698900	-2.92872100
H	7.36150800	-3.64927500	-3.45402700
H	-2.35215700	-8.84282000	0.54029300
H	-0.63152000	-8.67038600	0.16318300
H	-7.97879800	3.16501800	-3.67039800
H	-9.09607200	2.61933300	-4.87582900
H	-0.60340900	-2.86152800	7.83994500

H	0.00414900	-3.35599800	5.49331200
H	-1.75838200	-3.38295300	3.72611000
H	-4.12305900	-2.99055500	4.34767300
H	-4.73094500	-2.52397900	6.69946300
H	-2.21728000	-2.42256400	9.52043300
H	-3.86881800	-2.94631100	9.22996100
H	-2.78559100	-0.13215600	8.64664100
H	-3.84882300	-0.57519800	9.99223300
H	-4.43960300	-0.65937100	8.32623000
H	2.73997500	-2.14402100	5.75919200
H	2.45520400	-1.37302900	4.19229300
H	4.10099200	-1.56310200	4.78940300
H	-0.41872700	0.82855700	5.66590300
H	0.84523200	1.38283500	4.56110300
H	0.42265500	-0.33779300	4.64876900
H	1.27014400	-0.44244800	6.98072300
H	2.03437800	2.50736600	6.65536800
H	2.39208700	1.49289000	8.05869400
H	0.71425400	1.89388500	7.65871200
H	3.70966700	0.02481100	6.63323000
H	3.33602200	0.81126300	5.11350600
H	0.71240300	4.47088900	5.30893600
H	0.64640700	5.59602200	2.45841000
H	2.86343900	4.65040000	3.00971000
H	2.11831000	3.13817300	3.53800900
H	2.03556600	3.62264800	1.83645300
H	-0.57186000	3.00032600	3.53475300
H	-1.46800300	4.44554900	3.04132500
H	-0.56373800	3.51891900	1.83925600
H	-2.03669600	10.13048500	1.87328400
H	-1.34232900	9.51056500	-0.42508800
H	-2.91338600	8.82582000	-0.06882100
H	1.30676500	8.71789300	-0.13359200
H	3.38342100	7.45234300	-0.60935400
H	3.30877100	5.02875800	-1.10383200
H	1.15147000	3.80311700	-1.15014700
H	-1.65750300	4.22905100	-0.80227200
H	-3.17813100	6.14711700	-0.30506000
H	1.53392700	5.93914400	4.76277700
H	-0.23478900	5.86625500	4.77421900
H	-0.61726000	9.07379500	1.95710400
H	-2.21698900	8.42025900	2.30629000
H	-0.40534400	0.55039100	-0.90283300
O	-2.54577000	0.51429700	-2.58545500
H	-2.08128300	0.72745200	-3.40651500
H	-2.52751500	1.35342800	-2.05073500
C	-0.52305800	0.64468300	0.17495200
C	0.59907500	0.80833600	0.98854700
C	1.97106400	0.74814800	0.65426200
C	2.94707900	0.76732600	1.69325900
C	4.29218600	0.80942600	1.43131000
C	4.77297900	0.85494600	0.08155600
C	3.79231200	0.82616000	-0.96932700
C	2.45238600	0.76910600	-0.68869300
O	6.03239200	0.92620000	-0.18141600
H	2.60235000	0.75518100	2.72365000
H	5.02264200	0.83519800	2.23372100
H	4.14795100	0.85403800	-1.99385200
H	1.73811600	0.75869300	-1.50615600
H	0.39655000	0.97213500	2.04775200
H	-1.43472900	1.16166700	0.47217600
H	-1.03170900	-0.65698500	0.53362700
C	-1.85214900	-1.65938700	0.89578200
N	-2.86741300	-1.97729400	1.38322600

Int' (8.6 kcal/mol)

C	-1.52611600	8.92792700	2.41231000
C	-1.77570400	8.69357400	0.92736500
C	-1.28850400	7.36922000	0.41542300
C	-2.06282800	6.26829800	0.14080600
C	0.06836800	6.98178200	0.11984900
C	0.02764500	5.62725800	-0.32678600
C	1.30923000	7.64305600	0.16748100
N	-1.28591800	5.21973300	-0.30181800
C	1.18529500	4.94022800	-0.71236200
C	2.45747100	6.96171300	-0.21694800
C	2.39569600	5.62066400	-0.65011000
C	0.89299600	4.84076300	4.88305900
C	0.83812500	4.41706600	3.41432700
C	2.13301700	3.70904200	2.99596400
C	-0.39899200	3.55674300	3.12596100
N	-4.47889000	-1.56976000	-1.84516300
N	-6.82645600	-1.46721300	-1.68453800
N	-5.58292400	0.41032100	-2.22254500
N	-8.92214400	2.84884700	-3.44093100
C	1.97585700	0.97263800	7.17464300
C	1.83248900	-0.19262100	6.19450600
C	3.19604100	-0.54322100	5.56366400
C	0.75346700	0.08883600	5.14325500
C	3.23789100	-1.88098500	4.81694700
C	-3.23275500	-1.58036900	8.93537500
C	-2.77201900	-3.01409400	8.69265800
C	-2.46989100	-3.25284700	7.22275600
C	-3.50047400	-3.20951600	6.26992800
C	-1.15906600	-3.43695800	6.76753000
C	-3.22705700	-3.31006100	4.90574700
C	-0.88049300	-3.54982500	5.40204200
C	-1.91022300	-3.47152800	4.46353700
C	11.57689500	-2.40455900	-4.00463200
C	11.97645600	-0.98552000	-3.56295000
C	10.80350900	-0.15254300	-3.09008300
C	10.14828700	0.74181400	-3.94754400
C	10.31125400	-0.27328700	-1.78425200
C	9.04694100	1.48356000	-3.52731600
C	9.21233800	0.46154700	-1.34379700
C	8.56595900	1.34717100	-2.21842200
C	10.96398200	-4.77857800	2.15319400
C	9.56967100	-4.74610100	2.80274900
C	8.96554800	-3.35863600	2.82615800
C	9.10084000	-2.51977000	3.94096500
C	8.29328200	-2.84614600	1.71005500
C	8.59679800	-1.22198500	3.94439400
C	7.77960600	-1.55074500	1.69384800
C	7.93550100	-0.71986500	2.81475700
C	6.00672700	-3.52578100	-4.01281400
C	5.55977000	-2.73736400	-2.76169500
C	4.05939500	-2.65913700	-2.51844600
C	3.20312300	-1.99909000	-3.41588600
C	3.47098100	-3.19355000	-1.36343300
C	2.09958900	-3.08824300	-1.10445000
C	1.25805600	-2.43017400	-2.01482400
C	1.83664700	-1.88633500	-3.17720000
C	0.95543300	1.13546300	-7.11519100
C	0.38955100	2.56322600	-7.23944200
C	-0.92142200	2.76581400	-6.50675400
C	-2.15367300	2.54887100	-7.14062600
C	-0.95149800	3.15134900	-5.16062200
C	-3.36200200	2.69210100	-6.46344000
C	-2.15184700	3.30582700	-4.46344300
C	-3.37601500	3.06413400	-5.11104500
C	1.86202000	-5.22370300	-4.59624600
C	1.82721100	-6.74805700	-4.78152100
C	1.63082600	-7.50197900	-3.44601200
C	3.06080600	-7.24082100	-5.55422600
C	2.73748800	-7.31664400	-2.39993500
C	-1.49946000	-8.71759100	0.23023200
C	-1.42570500	-7.38775400	0.99543600
C	-1.91119700	-6.22387000	0.12157200
C	-2.21293700	-7.46081300	2.31125300
C	-3.49955100	-5.58979500	-4.70449500
C	-3.83936300	-5.07146600	-3.30456500
C	-4.01486400	-3.54993300	-3.26340400
C	-4.31739700	-3.02328200	-1.85479700
C	-5.62514500	-0.88505000	-1.90337500
C	-6.62864200	4.51171800	1.87842700
C	-6.03844200	4.30060200	0.48173200
C	-4.71928700	3.52331200	0.50192100

C	-4.09817700	3.25811700	-0.87104900
C	-9.49185300	0.68983100	3.50537800
C	-8.57547200	-0.34223800	2.83083700
C	-9.09654300	-0.77259300	1.46320500
C	-4.83447800	1.95962000	6.03603900
C	-4.51857200	1.13458400	4.78681300
C	-3.08048500	0.62893400	4.75924400
C	6.90201100	6.16026400	2.74232100
C	6.74987700	6.42192700	1.24011500
C	7.12945900	5.23908500	0.32873800
C	6.80271800	5.55560700	-1.13810800
C	8.60333700	4.82989200	0.46903800
C	-8.19686900	-5.63963700	-0.17772500
C	-7.43896100	-4.86742200	0.90712400
C	-6.31542100	-5.69827000	1.52847400
C	-9.25401900	-0.99375100	-5.92734500
C	-8.62185400	-0.04419900	-4.91430200
C	-9.65126600	0.58440100	-3.95131800
C	-8.93063600	1.56413200	-3.03937700
O	7.49913500	2.10244900	-1.84589300
O	7.46137700	0.55005500	2.86714600
O	-0.07906000	-2.29337300	-1.86116800
O	-4.57334000	3.17549800	-4.48496600
O	-2.88020200	2.90468500	-0.91340200
O	-4.84305000	3.35980400	-1.89183200
O	-7.25721300	0.18286800	2.62885400
O	-5.44839800	0.02625800	4.75524800
O	-6.91236800	-3.66230500	0.33332100
O	-6.85586800	4.71438400	-3.10917100
O	-5.34038200	-2.12285200	2.09887600
O	-8.32584900	1.16408400	-2.02979900
H	12.47360000	-0.47645300	-4.39812400
H	12.72225700	-1.05970700	-2.76105600
H	10.50922500	0.86335300	-4.96699900
H	10.79849100	-0.95221800	-1.08750200
H	8.54703300	2.17668200	-4.19666700
H	8.86071400	0.35669900	-0.32255500
H	7.16973500	1.83623500	-0.95408600
H	12.44907900	-2.97836000	-4.33840300
H	9.63929700	-5.13526900	3.82617200
H	8.90595300	-5.42948400	2.25796300
H	8.16173100	-3.47367400	0.83078700
H	9.61273100	-2.89060100	4.82689400
H	7.25682100	-1.17899800	0.81869100
H	8.70544700	-0.57777600	4.81126600
H	7.09397300	0.82132400	1.99216800
H	11.66176900	-4.13202200	2.69566600
H	5.96580900	-1.71925900	-2.83624300
H	6.03432000	-3.18170300	-1.87842800
H	3.61541700	-1.55492100	-4.31973400
H	4.10022200	-3.70095100	-0.63490100
H	1.18787100	-1.37393900	-3.88157400
H	1.67839700	-3.51410700	-0.19823300
H	-0.43707300	-2.43577600	-0.93192400
H	5.57856600	-3.09993700	-4.92621900
H	0.25281500	2.80082800	-8.30196100
H	1.13300500	3.27476300	-6.85946900
H	-2.16773500	2.25670700	-8.18881400
H	-0.01628700	3.35014900	-4.64126700
H	-4.30936700	2.51382700	-6.96285200
H	-2.14853400	3.61869300	-3.42331500
H	-4.47118200	3.23134400	-3.49139400
H	1.90058500	1.03707500	-7.66025200
H	0.94565800	-6.98593200	-5.39593300
H	3.03915000	-8.32793400	-5.69651600
H	3.99005500	-6.99129700	-5.02990900
H	3.11303600	-6.77509400	-6.54474400
H	0.67565500	-7.18182600	-3.00866800
H	1.52111000	-8.57351700	-3.66513900
H	2.51314900	-7.89697700	-1.49842200
H	2.83954400	-6.26991100	-2.09874800
H	3.71084300	-7.65544200	-2.77107700
H	2.73968900	-4.89921200	-4.03080600
H	-0.37071300	-7.20331700	1.24611400
H	-2.13580900	-6.52537000	2.87502000
H	-3.27761300	-7.64399000	2.11788600
H	-1.84822900	-8.27214900	2.95209900
H	-1.84842300	-5.26084600	0.63726300
H	-2.95816700	-6.37492300	-0.17197900
H	-1.31778500	-6.14568000	-0.79669500
H	-1.12385300	-9.55148400	0.83465200
H	-3.04729400	-5.36358900	-2.60456800

H	-4.75880200	-5.55532100	-2.94609400
H	-3.09842000	-3.06798900	-3.62893600
H	-4.82355400	-3.25049300	-3.94433700
H	-5.21022600	-3.49353500	-1.43683900
H	-3.49701600	-3.24890300	-1.16930200
H	-3.63772500	-1.00745200	-1.96704100
H	-7.60877500	-0.81873300	-1.65888900
H	-6.89214400	-2.29695900	-1.09584300
H	-6.39940500	0.99490200	-2.07616200
H	-4.68720900	0.81415600	-2.49474500
H	-4.28591500	-5.33448800	-5.42468200
H	-6.75700900	3.75720200	-0.14073300
H	-5.88355300	5.27169400	-0.00671100
H	-3.96782800	4.01923500	1.12722600
H	-4.87667200	2.53589800	0.95953200
H	-5.93441700	5.05346700	2.53185500
H	-8.51785000	-1.23096500	3.48069500
H	-9.07629000	0.07317700	0.76717000
H	-8.47728700	-1.57559700	1.05282300
H	-10.12670600	-1.13725000	1.53837300
H	-6.81190600	0.26038500	3.49764600
H	-10.49229500	0.27455100	3.67010500
H	-4.69260100	1.76130700	3.89818100
H	-2.38016500	1.46969600	4.77047200
H	-2.88202700	0.04373500	3.85433200
H	-2.88126000	-0.00874900	5.62628900
H	-5.06988500	-0.67646800	4.20052300
H	-4.64714800	1.36947500	6.93894100
H	5.70602600	6.69538800	1.03530900
H	7.35441700	7.29661300	0.95658700
H	6.51766400	4.37558300	0.62996800
H	5.74965300	5.83378400	-1.26330200
H	7.00616900	4.69021500	-1.77575200
H	7.40978200	6.39562500	-1.50068400
H	9.26707400	5.66431200	0.20615100
H	8.84853200	4.51449700	1.48785100
H	8.83197000	3.99594500	-0.20109100
H	7.94412800	5.97901900	3.02421200
H	-8.15551900	-4.59709900	1.70027800
H	-5.77857300	-5.11798100	2.28477800
H	-6.71363200	-6.59959100	2.00785000

H	-5.59576800	-6.00149100	0.76083400
H	-6.34213800	-3.20551800	1.00143700
H	-8.66134000	-6.53466200	0.24742500
H	-8.09183300	0.75870600	-5.44167100
H	-7.86763200	-0.57249500	-4.32197600
H	-10.44561600	1.08238600	-4.5213380
H	-10.11568100	-0.19059500	-3.33419600
H	-9.76625200	-1.82509200	-5.42969000
H	-6.35858500	4.52286900	-3.91991500
H	-6.24404700	4.36443300	-2.42590200
H	-4.41929200	-2.04762200	1.73225500
H	-5.78920000	-1.26509600	1.99240800
H	-9.99191900	-0.47828400	-6.55334500
H	-8.49616900	-1.42196000	-6.59107500
H	-8.98161000	-5.01745600	-0.61891200
H	-7.51259800	-5.94774900	-0.97554500
H	-3.38248000	-6.67871400	-4.71164500
H	-2.56348700	-5.15256300	-5.06918500
H	-9.58273600	1.58248200	2.87767000
H	-9.08760600	0.99424400	4.47702800
H	-7.55963000	5.08672400	1.83284200
H	-6.85455400	3.54966200	2.35392500
H	6.32009900	5.28401500	3.05037400
H	6.54757900	7.01529200	3.32827400
H	-4.20876300	2.85671500	6.06557900
H	-5.88384700	2.26995600	6.03839700
H	11.10604700	-2.95148300	-3.1812210
H	10.85687300	-2.36849400	-4.8288630
H	10.92015900	-4.42014100	1.11958600
H	11.37711700	-5.79387600	2.14334100
H	0.25226100	0.39826600	-7.51657200
H	1.14062500	0.87735600	-6.06732800
H	0.97806000	-4.86363100	-4.06062400
H	1.89319900	-4.71432700	-5.56600400
H	5.68758500	-4.57177600	-3.95473000
H	7.09699800	-3.50329800	-4.11097100
H	-2.53565700	-8.94930200	-0.04746400
H	-0.91016300	-8.68008100	-0.69284900
H	-8.20824700	3.51987400	-3.10891900
H	-9.36806100	3.08000300	-4.31554600
H	-0.34551800	-3.48036700	7.48874400

H	0.14646800	-3.68361900	5.07290400
H	-1.70345100	-3.50101500	3.39804200
H	-4.03154800	-3.23920700	4.17854600
H	-4.52807400	-3.07220300	6.59991300
H	-1.87914900	-3.22801400	9.29157400
H	-3.55063900	-3.70982200	9.03346000
H	-2.46001800	-0.86653900	8.63148000
H	-3.45643200	-1.40368600	9.99412900
H	-4.13553400	-1.35107200	8.36088400
H	2.98776200	-2.71157800	5.48775500
H	2.53098500	-1.91425000	3.98134900
H	4.23578000	-2.07418300	4.41013400
H	-0.19937400	0.34117300	5.61870000
H	1.04096800	0.93247700	4.50510600
H	0.56841200	-0.77688500	4.50074600
H	1.50510000	-1.07092100	6.77353900
H	2.32280400	1.87916100	6.66269400
H	2.69973100	0.74371300	7.96487100
H	1.01992000	1.21166000	7.65413600
H	3.95550800	-0.56368000	6.35654200
H	3.49456500	0.27053200	4.88704200
H	0.98037100	3.96800700	5.54307600
H	0.75257200	5.32733000	2.80519700
H	3.00563500	4.34967400	3.16520700
H	2.28521600	2.78670800	3.57013100
H	2.11674700	3.44865100	1.93389700
H	-0.37449700	2.62294600	3.70027900
H	-1.32021000	4.08779300	3.39154900
H	-0.46421800	3.29739300	2.06469300
H	-1.88746800	9.91415300	2.72698900
H	-1.30284600	9.50369000	0.35387700
H	-2.85130700	8.77802300	0.72605000
H	1.36933500	8.67689000	0.49827300
H	3.41951300	7.46566100	-0.18699700
H	3.30947100	5.10861000	-0.93725400
H	1.13876300	3.90856100	-1.04758000
H	-1.66423200	4.30113100	-0.53869900
H	-3.13556600	6.15676800	0.21739000
H	1.75382000	5.49004700	5.07907400
H	-0.01069900	5.38714500	5.17739500
H	-0.45890700	8.87014000	2.65103500
H	-2.03535900	8.17154300	3.01887800
H	0.33748700	1.46014300	-1.64600200
O	-2.80534600	0.77917800	-2.57784200
H	-2.41913300	1.04935600	-3.42204900
H	-2.71655500	1.57209200	-1.98813100
C	-0.09141200	0.86590900	-0.83734900
C	0.87815300	0.48680900	0.21255000
C	2.23404800	0.68443300	0.23583000
C	2.98906300	0.29166000	1.40714900
C	4.32648900	0.49755400	1.50605000
C	5.06530500	1.09792600	0.41009300
C	4.32042100	1.46616800	-0.78335300
C	2.97839600	1.27715900	-0.85655000
O	6.31532800	1.28742300	0.48629700
H	2.44510200	-0.16425500	2.22949300
H	4.88313000	0.22541800	2.39636800
H	4.88461100	1.88473200	-1.61051600
H	2.44874600	1.54847100	-1.76309500
H	0.43834400	-0.02626300	1.06763400
H	-0.92671700	1.41894000	-0.39079800
H	-0.53376600	-0.04285300	-1.26521000
C	-1.65556400	-2.29618200	0.51434900
N	-2.64837900	-2.15281800	1.13059400

TS2' (13.1 kcal/mol)

C	-2.02139400	8.99711600	2.19124600
C	-2.26383300	8.70548300	0.71549100
C	-1.69504400	7.40491300	0.22744100
C	-2.41216400	6.28201300	-0.10562400
C	-0.31027900	7.07046600	0.00189800
C	-0.27632000	5.72250700	-0.46501800
C	0.90169300	7.77409900	0.12879700
N	-1.57481500	5.26869800	-0.51852400
C	0.92472100	5.08131000	-0.79492500
C	2.09281400	7.13939700	-0.20094200
C	2.10398800	5.80442100	-0.65659500
C	0.72415400	5.17379600	4.74169700
C	0.69036100	4.70824900	3.28511800
C	1.96685900	3.94255300	2.91378800
C	-0.56908000	3.87718100	3.00924900
N	-4.14058700	-1.70622200	-1.87691300
N	-6.47896000	-1.82920800	-1.59614500
N	-5.46597900	0.11804000	-2.32253400
N	-9.13795400	2.17062500	-3.36997200
C	2.11915600	1.46149600	7.12083700
C	2.05787100	0.26324100	6.17233000
C	3.41308400	0.03393300	5.46927900
C	0.88172200	0.37728300	5.19681300
C	3.63421300	-1.40210400	4.98114700
C	-2.86352700	-1.43547800	8.99847100
C	-2.29667200	-2.83584200	8.78725600
C	-2.01524400	-3.10972800	7.31958100
C	-3.06841700	-3.17630900	6.39312100
C	-0.70615700	-3.23356200	6.83970400
C	-2.82104700	-3.33106400	5.02887500
C	-0.45180300	-3.39836500	5.47516400
C	-1.50677000	-3.43416300	4.56264400
C	11.86179900	-1.46360300	-4.06321300
C	11.76494800	-0.05982600	-3.43498100
C	10.35163600	0.37148500	-3.09499800
C	9.53492800	1.00613400	-4.04275800
C	9.80220000	0.14242500	-1.82694200
C	8.22898300	1.38619500	-3.74567900
C	8.49789800	0.51985200	-1.50792100
C	7.68998300	1.14360100	-2.47166600
C	11.48213800	-3.71476000	2.15916800
C	10.14584100	-3.97164800	2.87779000
C	9.18928500	-2.80506700	2.76288600
C	9.18642300	-1.77045600	3.70900800
C	8.31870400	-2.68604000	1.67253300
C	8.36574200	-0.65552700	3.56905400
C	7.48596400	-1.57986600	1.51543500
C	7.51151000	-0.53948600	2.46067500
C	6.39434500	-3.00794500	-3.98723400
C	5.82237700	-2.09219500	-2.88915400
C	4.32292600	-2.18256700	-2.67202100
C	3.41983100	-1.65525400	-3.60913300
C	3.78207900	-2.72879600	-1.50095200
C	2.40703000	-2.73090500	-1.25383500
C	1.53026800	-2.16527500	-2.18462000
C	2.04633400	-1.64826300	-3.38009000
C	0.97546400	1.16957800	-7.15761200
C	0.24340500	2.50436000	-7.40183300
C	-1.08517600	2.63069000	-6.68050700
C	-2.29093500	2.25736500	-7.29261700
C	-1.15918800	3.11852200	-5.36908700
C	-3.51192600	2.34759800	-6.62749400
C	-2.37380300	3.22481200	-4.68657400
C	-3.56924900	2.82549800	-5.30967900
C	2.38777500	-5.03299500	-4.48568600
C	2.46157300	-6.56340100	-4.59589100
C	2.27506800	-7.26400600	-3.23042800
C	3.74888800	-7.00642300	-5.30888200
C	3.33656000	-6.95565000	-2.16663000
C	-0.65686600	-8.64578000	0.46196900
C	-0.62684800	-7.31061200	1.22138800
C	-1.11753000	-6.16037900	0.33123900
C	-1.44153500	-7.39480500	2.51978900
C	-2.93049400	-5.81123000	-4.53105200
C	-3.22543500	-5.22636600	-3.14597600
C	-3.55073400	-3.72921400	-3.19056000
C	-3.83051800	-3.13374500	-1.80392200
C	-5.35742900	-1.14889700	-1.91685300
C	-6.77366100	4.19071200	1.82162500
C	-6.28611900	4.01541900	0.37847700
C	-4.88725600	3.39817000	0.29971800

C	-4.33377900	3.16244500	-1.10843000
C	-9.32169100	0.20487200	3.57438000
C	-8.35671200	-0.81218600	2.94549100
C	-8.83119800	-1.29315300	1.57794200
C	-4.75576600	1.89368200	6.02514200
C	-4.40789000	1.01921900	4.82092000
C	-2.93466100	0.62980900	4.78044400
C	6.59698400	6.89257300	2.50807700
C	7.04636800	6.53891100	1.08555000
C	6.45356300	5.23444000	0.51587600
C	6.83639800	5.07258700	-0.96323000
C	6.87232900	3.99320200	1.31627200
C	-7.57386900	-6.10149300	0.04214600
C	-6.74884100	-5.29454600	1.04807300
C	-5.47871800	-6.03178100	1.47515300
C	-9.03051300	-1.70310800	-5.81395400
C	-8.49995500	-0.65529600	-4.84094400
C	-9.56205800	-0.17979200	-3.82612200
C	-8.94874400	0.90422500	-2.95552600
O	6.41224200	1.52675000	-2.23967500
O	6.74367900	0.56589300	2.35991700
O	0.17850400	-2.08449400	-1.98694100
O	-4.77944500	2.88246200	-4.69828600
O	-3.09082000	2.93616900	-1.21607600
O	-5.14011500	3.14774400	-2.08652200
O	-7.05021000	-0.24796100	2.75757400
O	-5.24416100	-0.16140100	4.88797600
O	-6.41966300	-4.02409200	0.46501500
O	-7.30526100	4.28713500	-3.20209000
O	-5.00491800	-2.33896700	2.21722000
O	-8.24974400	0.60846900	-1.97041000
H	12.21202700	0.66716000	-4.12559800
H	12.37989200	-0.03561700	-2.52650200
H	9.93112800	1.21127700	-5.03589400
H	10.40662500	-0.33798200	-1.06009300
H	7.60579200	1.87853900	-4.48601900
H	8.10725900	0.34096700	-0.51216200
H	6.07844500	1.22333700	-1.35097400
H	12.90150600	-1.72581100	-4.29144100
H	10.34301000	-4.18727300	3.93580000
H	9.68263000	-4.87470800	2.46015600
H	8.28927500	-3.47709900	0.92550100
H	9.84595700	-1.83774400	4.57253300
H	6.81401300	-1.50598600	0.66612900
H	8.37259800	0.14525100	4.30201900
H	6.24508300	0.58486800	1.49188400
H	11.98299500	-2.83369000	2.57376900
H	6.07737900	-1.05388200	-3.12676500
H	6.33089600	-2.31323400	-1.94379000
H	3.80230200	-1.21256300	-4.52618500
H	4.45072700	-3.13564900	-0.74618800
H	1.35938300	-1.21181800	-4.09886800
H	2.01889000	-3.13404800	-0.32227000
H	-0.03590700	-2.14530800	-1.03731100
H	5.92704400	-2.80632900	-4.95741800
H	0.08109400	2.62790300	-8.48004500
H	0.90027000	3.32775500	-7.09547000
H	-2.27501600	1.88440200	-8.31486400
H	-0.24896500	3.43775500	-4.86598000
H	-4.43627700	2.04719900	-7.11143300
H	-2.40216400	3.61603200	-3.67380900
H	-4.70205300	3.01160400	-3.71270500
H	1.92425400	1.13752600	-7.70379200
H	1.61728100	-6.89048100	-5.22184700
H	3.80303100	-8.09780300	-5.40143100
H	4.64264200	-6.67180900	-4.77064600
H	3.80174800	-6.58232800	-6.31782900
H	1.28758100	-6.98753400	-2.83623000
H	2.24156700	-8.34991000	-3.39788300
H	3.12547400	-7.50689300	-1.24377500
H	3.36423500	-5.89139400	-1.91435800
H	4.33963600	-7.24620400	-2.49652000
H	3.22616100	-4.62118600	-3.91814900
H	0.41825300	-7.10284000	1.49360700
H	-1.39612300	-6.45689900	3.08327400
H	-2.49752800	-7.60094300	2.30379700
H	-1.07397300	-8.19615600	3.17104800
H	-1.09463000	-5.19814300	0.85308500
H	-2.15231000	-6.33540800	0.01062000
H	-0.50181400	-6.06650700	-0.57060900
H	-0.27421400	-9.46666600	1.07904600
H	-2.36228500	-5.39022000	-2.48878600

H	-4.06535300	-5.76873100	-2.68959000	H	-4.84754700	-6.23718100	0.60402500
H	-2.70989200	-3.18683200	-3.64234000	H	-5.89482600	-3.50529000	1.12250400
H	-4.42236300	-3.55746500	-3.83663000	H	-7.88934400	-7.05110800	0.48500100
H	-4.64850800	-3.65980000	-1.30527500	H	-8.12800700	0.21270000	-5.39924400
H	-2.95802200	-3.23561300	-1.15313700	H	-7.64255600	-1.05344400	-4.28848000
H	-3.37612200	-1.06831800	-2.10050800	H	-10.44963700	0.18966100	-4.3547920
H	-7.32530600	-1.26621500	-1.57300700	H	-9.87389900	-1.00994700	-3.18529100
H	-6.44447800	-2.65167400	-0.99727600	H	-9.38536800	-2.59489000	-5.28496600
H	-6.32453800	0.63267400	-2.15287400	H	-6.85474800	4.15558600	-4.04999300
H	-4.62631000	0.59577800	-2.64832400	H	-6.61125600	3.99400600	-2.56939100
H	-3.78534900	-5.68529100	-5.20563300	H	-4.12667100	-2.15224100	1.81749700
H	-6.98632600	3.37838600	-0.17258100	H	-5.55886100	-1.53670500	2.15551700
H	-6.28775300	4.98616200	-0.13404800	H	-9.86829400	-1.31401700	-6.40437000
H	-4.15505300	3.99484200	0.85555500	H	-8.25055800	-2.02096400	-6.51304100
H	-4.89086900	2.41304700	0.78849600	H	-8.46575400	-5.54468500	-0.26044200
H	-6.09195200	4.82362500	2.40178800	H	-6.98194900	-6.31606500	-0.85416200
H	-8.28294800	-1.68180100	3.61908700	H	-2.70598800	-6.88136800	-4.47334800
H	-8.83645100	-0.46203600	0.86433000	H	-2.06928300	-5.31538500	-4.99234200
H	-8.17045900	-2.07668000	1.19474100	H	-9.42929000	1.07805900	2.92261000
H	-9.84526500	-1.70176600	1.64300300	H	-8.95054700	0.54644200	4.54682100
H	-6.63481100	-0.10199100	3.63266200	H	-7.76523300	4.65462300	1.85151400
H	-10.31013100	-0.24180500	3.7273520	H	-6.84464600	3.22130600	2.32931200
H	-4.65769300	1.56942600	3.90045500	H	5.50438800	6.95912200	2.57081700
H	-2.30450300	1.52158400	4.71313200	H	7.00655500	7.85892200	2.82338900
H	-2.71517800	0.00065600	3.91021600	H	-4.20698200	2.83892900	5.97756900
H	-2.65864700	0.07039700	5.67979400	H	-5.82681600	2.11706000	6.04363700
H	-4.81285600	-0.86658700	4.37854600	H	11.46005300	-2.22310100	-3.3844060
H	-4.49404600	1.38086900	6.95616500	H	11.28570100	-1.51625500	-4.9931640
H	6.77859900	7.36674100	0.41406500	H	11.32051000	-3.52857200	1.09239600
H	8.14391100	6.46957900	1.05542800	H	12.16143200	-4.57017800	2.25605200
H	5.35677700	5.31781100	0.57424900	H	0.36488000	0.32114200	-7.48431500
H	6.47696800	5.91617700	-1.56485800	H	1.19367900	1.03071100	-6.09369800
H	6.42768200	4.14986300	-1.38693200	H	1.46709100	-4.71019400	-3.98972900
H	7.92699300	5.02594400	-1.07718800	H	2.40943500	-4.56990900	-5.47846900
H	7.96278900	3.86992900	1.30272800	H	6.22393500	-4.06482400	-3.75271500
H	6.55663700	4.04583300	2.36267300	H	7.47224100	-2.84965600	-4.09450700
H	6.43315100	3.08690000	0.89097000	H	-1.68225900	-8.90206700	0.16674000
H	6.92350300	6.14385600	3.23633800	H	-0.05084400	-8.60055700	-0.44972300
H	-7.37047200	-5.11955200	1.94107500	H	-8.50405100	2.93809600	-3.08895100
H	-4.89863400	-5.42978200	2.18094700	H	-9.65230400	2.32088300	-4.22458300
H	-5.72213700	-6.98413300	1.95945700	H	0.12507900	-3.18956900	7.53994100

H	0.57371900	-3.48400500	5.12660900
H	-1.31770800	-3.51456000	3.49680200
H	-3.64635200	-3.35212900	4.32164500
H	-4.09459300	-3.08775700	6.74304800
H	-1.37409200	-2.95729600	9.36605100
H	-3.00758700	-3.57809300	9.17485900
H	-2.15728400	-0.67292800	8.65356800
H	-3.07422000	-1.24422200	10.0570530
H	-3.79568300	-1.29577000	8.44218600
H	3.61317300	-2.10674400	5.82136200
H	2.86628700	-1.72071700	4.26858600
H	4.60494800	-1.50591700	4.48665200
H	-0.05681300	0.53627900	5.73781600
H	1.02077400	1.21699600	4.50723600
H	0.75702200	-0.53400300	4.60408700
H	1.86871500	-0.62415100	6.79838300
H	2.33329700	2.38955100	6.57578700
H	2.90601100	1.33338500	7.87255000
H	1.16934600	1.60300900	7.64952800
H	4.22082300	0.28542800	6.16894400
H	3.51542600	0.73356400	4.62947200
H	0.76155300	4.31821800	5.42850600
H	0.64213800	5.59990600	2.64540300
H	2.85684200	4.56464400	3.05909600
H	2.08620200	3.04596700	3.53374500
H	1.95266900	3.62629600	1.86692500
H	-0.56121400	2.94722100	3.59218100
H	-1.47671900	4.43176600	3.27414500
H	-0.64335000	3.61204600	1.95123800
H	-2.44705900	9.96521500	2.48048600
H	-1.85027800	9.53210400	0.11934200
H	-3.34387600	8.71291400	0.52022200
H	0.90675600	8.80404100	0.47685400
H	3.03339200	7.67477100	-0.10685600
H	3.05001200	5.33259700	-0.90502100
H	0.93889700	4.05251100	-1.14325000
H	-1.90944100	4.34236100	-0.78619800
H	-3.48301100	6.13278400	-0.09321000
H	1.60405100	5.79550100	4.94316800
H	-0.16519200	5.76256900	4.99563100
H	-0.95169900	9.01864900	2.42390700
H	-2.47461800	8.22563400	2.82291100
H	-1.10702500	0.73829000	-0.87269500
O	-2.74728100	0.75194700	-2.74048400
H	-2.36551300	1.01487300	-3.58953100
H	-2.77158200	1.58753700	-2.20218400
C	-1.10949200	0.98977700	0.18837000
C	-0.11497500	0.17438000	0.96752200
C	1.27094900	0.20587400	0.65900300
C	2.21918400	-0.18758500	1.64602500
C	3.56843500	-0.03036500	1.46853600
C	4.09567700	0.54014100	0.25923200
C	3.14273600	0.90872500	-0.75195600
C	1.79419600	0.73240700	-0.56053000
O	5.36318000	0.71038200	0.10098400
H	1.84695100	-0.60807800	2.57533200
H	4.27007200	-0.31103700	2.24601300
H	3.52611400	1.31840900	-1.68096500
H	1.10885600	1.01349400	-1.35420400
H	-0.33117300	0.06036600	2.02530300
H	-0.85159100	2.04962500	0.28506800
H	-2.12299800	0.85180500	0.56138800
C	-1.18521500	-1.63951100	0.78701500
N	-2.21814500	-2.05281000	1.16793800

Prod' (-14.9 kcal/mol)

C	-1.82791800	9.16163100	1.26774400
C	-2.04410500	8.73205900	-0.17810200
C	-1.49623500	7.37492300	-0.51434500
C	-2.22891500	6.23564900	-0.74034500
C	-0.11276700	6.99101400	-0.65771200
C	-0.09428200	5.59897800	-0.96663800
C	1.11054900	7.68097200	-0.56832900
N	-1.40172400	5.16706300	-1.01499100
C	1.10120900	4.89877300	-1.17484800
C	2.29640200	6.98739300	-0.77468500
C	2.29195200	5.60840300	-1.07198900
C	0.67063500	5.50330100	4.27359600
C	0.65434000	4.89408300	2.87073900
C	1.91547500	4.06414100	2.60612900
C	-0.63104200	4.08414500	2.65511300
N	-4.10249600	-1.86417000	-1.50720900
N	-6.46462700	-1.88586700	-1.49991700
N	-5.29828200	-0.07015000	-2.31949800
N	-8.88019900	2.05556200	-3.69658800
C	1.83018500	1.99598800	7.05582600
C	1.73939900	0.71117800	6.23109200
C	3.12147200	0.31686000	5.66920600
C	0.65742900	0.81590500	5.15071100
C	3.23213900	-1.14300500	5.21556700
C	-3.32821300	-0.47851000	9.05892600
C	-2.82075400	-1.91613500	9.00726900
C	-2.49251200	-2.34035900	7.58583500
C	-3.51625200	-2.52526500	6.64279200
C	-1.16771100	-2.49167600	7.15880300
C	-3.22551800	-2.83170400	5.31272500
C	-0.86955400	-2.80537000	5.83017100
C	-1.89667500	-2.96810200	4.89985500
C	11.72299500	-2.46693900	-3.4690590
C	11.79063900	-0.92982900	-3.4420690
C	10.46283800	-0.27700500	-3.1205360
C	9.66206200	0.29520500	-4.11763900
C	9.97156100	-0.25207800	-1.80767700
C	8.42358900	0.86293500	-3.82506600
C	8.73745400	0.30925500	-1.49416300
C	7.93832100	0.87497000	-2.50626300
C	11.07497800	-4.05501100	2.93313100
C	9.83316300	-3.76504000	3.79450900
C	9.13444800	-2.47881500	3.41006000
C	9.39170500	-1.27618600	4.08207500
C	8.24318700	-2.43469100	2.32932400
C	8.79645700	-0.07962200	3.69226800
C	7.63694400	-1.24912300	1.92269300
C	7.91274800	-0.04490000	2.59954700
C	6.19047600	-3.75957600	-3.40861100
C	5.55727200	-2.36530100	-3.23715600
C	4.06920700	-2.41784600	-2.95971400
C	3.12059700	-2.11496900	-3.94487400
C	3.58922900	-2.79610600	-1.69789400
C	2.22584500	-2.89612100	-1.43241200
C	1.29509200	-2.60026700	-2.43654100
C	1.74983800	-2.19782900	-3.69529000
C	1.05502300	0.30231100	-7.14971500
C	0.40529900	1.65819900	-7.48579700
C	-0.92490100	1.88490300	-6.79615900
C	-2.13420800	1.50472900	-7.39586500
C	-0.99375200	2.46359700	-5.52224700
C	-3.35803000	1.67445800	-6.75220000
C	-2.21083300	2.65006900	-4.86264800
C	-3.40948400	2.24211100	-5.47177500
C	2.10995200	-5.65108800	-3.82560400
C	2.34982300	-7.16668300	-3.74808600
C	2.23120300	-7.71053300	-2.30583800
C	3.68290200	-7.55135800	-4.40907000
C	3.24389400	-7.15741900	-1.29495400
C	-1.22772200	-8.60785800	1.36273800
C	-0.90002000	-7.22136600	1.93517600
C	-1.16218800	-6.12182000	0.89711300
C	-1.68132000	-6.96076200	3.23048900
C	-3.23529200	-6.20107500	-3.95810800
C	-3.46434300	-5.51389100	-2.60654900
C	-3.62904300	-3.99362500	-2.72939400
C	-3.87772000	-3.30829300	-1.37731000
C	-5.29040200	-1.28129000	-1.77008200
C	-6.78391400	4.55084700	1.23500100
C	-6.20328800	4.12847500	-0.11612900
C	-4.81085500	3.50737200	0.00844400

C	-4.18122900	3.03440700	-1.30386700
C	-9.55772700	0.87701700	3.29910500
C	-8.66369000	-0.26838200	2.82349100
C	-9.11042800	-0.84953800	1.48617200
C	-4.98606700	2.60895500	5.70878100
C	-4.62936800	1.60022900	4.62100000
C	-3.18405900	1.12645100	4.71431600
C	6.67346600	6.73256200	2.06253100
C	6.81161100	6.60427200	0.54125700
C	6.61948300	5.17840300	-0.01242600
C	6.60730100	5.18811200	-1.54867500
C	7.67592900	4.19240600	0.50461600
C	-8.00727000	-5.82516700	0.47431600
C	-7.15940200	-4.98924300	1.43228500
C	-5.91101800	-5.73398000	1.90703000
C	-9.10431700	-1.98692300	-5.83646300
C	-8.47592800	-0.92545900	-4.94029500
C	-9.48723800	-0.28191600	-3.96796500
C	-8.77634200	0.81402700	-3.19346900
O	6.74021800	1.43823100	-2.26163900
O	7.36582900	1.13323000	2.25330700
O	-0.05724200	-2.71040300	-2.23807300
O	-4.62155200	2.37290200	-4.87103400
O	-2.95305600	2.72746300	-1.28355500
O	-4.92731000	2.93177600	-2.32411500
O	-7.31006900	0.18844300	2.64635800
O	-5.54252200	0.48137400	4.75299600
O	-6.79814700	-3.76662400	0.76828200
O	-6.94538200	4.06641600	-3.70101100
O	-5.50613600	-1.81653400	2.33075100
O	-8.07605200	0.53583900	-2.20197300
H	12.15078200	-0.56881700	-4.4139810
H	12.54412500	-0.62353500	-2.7037950
H	10.01594400	0.29879500	-5.14759500
H	10.56956600	-0.67736500	-1.0036120
H	7.81186200	1.30757800	-4.60445600
H	8.38901600	0.32804800	-0.46797400
H	6.43123500	1.25700300	-1.30803200
H	12.70034700	-2.90816200	-3.6997490
H	10.13121300	-3.72164600	4.85004800
H	9.13569000	-4.60880800	3.70709800
H	8.01615700	-3.35253900	1.78912600
H	10.07580800	-1.27649600	4.92944800
H	6.95121300	-1.23152300	1.08212800
H	9.00217700	0.84807500	4.21783900
H	6.78299700	1.04783500	1.42215700
H	11.80939700	-3.24800900	3.02605900
H	5.74108900	-1.77159900	-4.13950600
H	6.06471000	-1.83606500	-2.42262000
H	3.45927600	-1.80337300	-4.93046500
H	4.29592700	-3.00557400	-0.89889300
H	1.02341200	-1.96129800	-4.46677400
H	1.88071900	-3.18536600	-0.44456100
H	-0.22689100	-2.78521600	-1.28222700
H	5.73723800	-4.29708400	-4.24904400
H	0.26778100	1.72910500	-8.57217600
H	1.09890500	2.46186400	-7.21072700
H	-2.11824800	1.06232800	-8.38997100
H	-0.07836800	2.78993200	-5.03370400
H	-4.28691700	1.36829300	-7.22370500
H	-2.23852400	3.12006400	-3.88382000
H	-4.52836100	2.58920600	-3.90192400
H	2.00890200	0.18338100	-7.67476900
H	1.55062900	-7.65865200	-4.32372800
H	3.85525400	-8.63370000	-4.36911100
H	4.53103400	-7.05902100	-3.92097400
H	3.69630200	-7.24954300	-5.46237300
H	1.21648200	-7.49749500	-1.94153600
H	2.31843200	-8.80594200	-2.33737500
H	3.08185600	-7.59974200	-0.30596100
H	3.15865500	-6.07199000	-1.18856600
H	4.27486400	-7.38252500	-1.58719900
H	2.89589300	-5.08574200	-3.32079000
H	0.17194600	-7.20373400	2.17945300
H	-1.42972300	-5.98693900	3.66360100
H	-2.76241300	-6.96940700	3.04097500
H	-1.47175400	-7.72692200	3.98557200
H	-0.89119500	-5.12945500	1.27095600
H	-2.22543800	-6.09663400	0.62437400
H	-0.58803500	-6.29685500	-0.01982300
H	-1.01441600	-9.40202300	2.08726500
H	-2.62220900	-5.73434600	-1.93863000

H	-4.35712300	-5.93985800	-2.12685800	H	-5.28496000	-6.01509200	1.05343200
H	-2.72631600	-3.56179700	-3.17929300	H	-6.33369700	-3.18113600	1.40883800
H	-4.46737500	-3.76590800	-3.40152600	H	-8.34553000	-6.74219900	0.96580500
H	-4.72903900	-3.75747000	-0.85772800	H	-8.02792400	-0.13799000	-5.55878600
H	-3.00943500	-3.43425000	-0.72281100	H	-7.65663200	-1.35899900	-4.35687900
H	-3.29848900	-1.29715200	-1.78568200	H	-10.34417800	0.11604600	-4.5251840
H	-7.28690400	-1.30095400	-1.63469100	H	-9.86322700	-1.02773400	-3.26111500
H	-6.53132000	-2.65487300	-0.83637000	H	-9.53665400	-2.80325600	-5.24668000
H	-6.14950500	0.48497900	-2.31114800	H	-6.45644900	3.79863300	-4.49508400
H	-4.40705800	0.33340700	-2.61526100	H	-6.30650200	3.82307200	-2.99451800
H	-4.07489100	-6.02442800	-4.64031500	H	-4.80738400	-1.53556600	1.72364900
H	-6.87151800	3.40635100	-0.59767000	H	-6.17638000	-1.08489000	2.31822400
H	-6.15783700	4.99644600	-0.78604300	H	-9.90583900	-1.56531000	-6.45397500
H	-4.10646300	4.19222400	0.49452700	H	-8.36064700	-2.42345000	-6.51060700
H	-4.85252200	2.62318600	0.66122400	H	-8.88494900	-5.26153900	0.14488100
H	-6.13892000	5.27949200	1.73977100	H	-7.42430800	-6.10250700	-0.41058600
H	-8.67803500	-1.06663200	3.58321800	H	-3.12270300	-7.28359200	-3.83968100
H	-9.02140600	-0.09677000	0.69577400	H	-2.32867900	-5.82238900	-4.44178700
H	-8.49806600	-1.71382300	1.21075500	H	-9.56548100	1.68414900	2.55966700
H	-10.15388700	-1.17636600	1.5411480	H	-9.19873400	1.28591000	4.24986100
H	-6.92701700	0.45785300	3.50993700	H	-7.77267700	5.00714600	1.11862200
H	-10.58565100	0.53024900	3.44881700	H	-6.89404900	3.68631800	1.90105900
H	-4.79170600	2.06577600	3.63662100	H	5.69509200	6.37127600	2.40015500
H	-2.49710800	1.96908900	4.59485600	H	6.77032600	7.77637300	2.38293900
H	-2.95180800	0.39881600	3.92857900	H	-4.37697000	3.51133300	5.60242100
H	-2.99392800	0.65024600	5.68118000	H	-6.03991300	2.89578000	5.64127200
H	-5.12596500	-0.29512900	4.34580600	H	11.39436700	-2.85969300	-2.5013670
H	-4.80835600	2.17893400	6.69957200	H	11.00672700	-2.81135300	-4.2226890
H	6.07882400	7.26936900	0.06227100	H	10.80486800	-4.13108600	1.87483400
H	7.80099800	6.97362900	0.23172100	H	11.55955800	-4.99365600	3.22871200
H	5.63546700	4.82065000	0.32736900	H	0.40138800	-0.52764600	-7.43914400
H	5.81494800	5.83761800	-1.94119600	H	1.24847600	0.21462200	-6.07581200
H	6.45646000	4.17982200	-1.94713800	H	1.15515000	-5.37367600	-3.36800800
H	7.56282800	5.55907800	-1.94257800	H	2.08693100	-5.31504800	-4.86772100
H	8.68439400	4.51532500	0.21309500	H	6.04783000	-4.36899900	-2.50971600
H	7.65465300	4.08505800	1.59267400	H	7.26694400	-3.67859000	-3.59310900
H	7.50439700	3.19820300	0.08694100	H	-2.29017800	-8.67803400	1.09663700
H	7.43715300	6.15279300	2.58988300	H	-0.64520800	-8.81406100	0.45812500
H	-7.77523900	-4.73858000	2.31063000	H	-8.18885900	2.79781900	-3.48603500
H	-5.31385100	-5.10691400	2.57667700	H	-9.40869100	2.18279900	-4.54637300
H	-6.18169800	-6.64553300	2.45092700	H	-0.35852100	-2.35602000	7.87250600

H	0.16615900	-2.91153000	5.52135000
H	-1.66384600	-3.18999400	3.86224100
H	-4.03228000	-2.95229300	4.59387400
H	-4.55347000	-2.41680400	6.95253400
H	-1.92972100	-2.02132900	9.63634000
H	-3.58137100	-2.58698600	9.42797400
H	-2.57095800	0.21670900	8.68173900
H	-3.57923500	-0.17878600	10.0827390
H	-4.22550100	-0.35394400	8.44411200
H	3.05241900	-1.82649300	6.05473800
H	2.51312500	-1.38741400	4.42741000
H	4.22878700	-1.35672000	4.81812700
H	-0.31476500	1.04889600	5.59766300
H	0.89603700	1.60790900	4.43295700
H	0.54341400	-0.12138700	4.59726800
H	1.43144400	-0.09290200	6.91966800
H	2.16839400	2.83832700	6.43977200
H	2.54058400	1.88752900	7.88279200
H	0.85795700	2.26983300	7.48221800
H	3.87817200	0.49635900	6.44430400
H	3.38062500	0.98689400	4.83868200
H	0.66223700	4.72064400	5.04374800
H	0.64440900	5.71924900	2.14587700
H	2.81681900	4.67981800	2.69852900
H	2.00922400	3.23773500	3.32044000
H	1.91470200	3.63392500	1.60135700
H	-0.65960800	3.20715500	3.31521800
H	-1.51882000	4.69154900	2.86844800
H	-0.71591600	3.73455000	1.62345500
H	-2.22778400	10.16625700	1.44953100
H	-1.59331500	9.48404800	-0.84200300
H	-3.11850000	8.74661300	-0.40264700
H	1.12841200	8.74411900	-0.34165100
H	3.24609000	7.51029900	-0.70515000
H	3.23408300	5.09032400	-1.22237300
H	1.10481200	3.83728600	-1.40478600
H	-1.73895700	4.21387900	-1.13986600
H	-3.30279700	6.10923000	-0.74267000
H	1.56729900	6.11225600	4.43553500
H	-0.20288200	6.14385300	4.44374600
H	-0.76377700	9.17225300	1.52468900
H	-2.32084300	8.47024500	1.95958600
H	-0.56907900	0.83730100	-0.95461700
O	-2.56334800	0.42094900	-2.57821200
H	-2.13803300	0.58107200	-3.43230300
H	-2.59334700	1.30903100	-2.12806000
C	-0.58066900	1.14625300	0.09131800
C	0.31271000	0.23386100	0.95488500
C	1.80879000	0.38568300	0.69138800
C	2.70193800	0.37485600	1.77104900
C	4.06474100	0.56394600	1.59624500
C	4.61607200	0.77443100	0.30375400
C	3.70264100	0.77833900	-0.78610100

9. References

[1] a) C. Wuensch, T. Pavkov-Keller, G. Steinkellner, J. Gross, M. Fuchs, A. Hromic, A. Lyskowski, K. Fauland, K. Gruber, S. M. Glueck, K. Faber, *Adv. Synth. Catal.* **2015**, *357*, 1909-1918; b) C. Wuensch, J. Gross, G. Steinkellner, K. Gruber, S. M. Glueck, K. Faber, *Angew. Chem. Int. Ed.* **2013**, *52*, 2293-2297.

[2] Z.-S. Yang, W.-L. Zhou, Y. Sui, J.-X. Wang, J.-M. Wu, Y. Zhou, Y. Zhang, P.-L. He, J.-Y. Han, W. Tang, Y. Li, J.-P. Zuo, *J. Med. Chem.* **2005**, *48*, 4608-4617.

[3] B. A. Gellert, N. Kahlcke, M. Feurer, S. Roth, *Chem. Eur. J.* **2011**, *17*, 12203-12209.

[4] J. S. Yadav, S. Nanda, P. T. Reddy, A. B. Rao, *J. Org. Chem.* **2002**, *67*, 3900-3903.

[5] J. Guin, G. Varseev, B. List, *J. Am. Chem. Soc.* **2013**, *135*, 2100-2103.
